# Comparative Genomic Insights into Endofungal Lifestyles of Two Bacterial Endosymbionts, *Mycoavidus cysteinexigens* and *Burkholderia rhizoxinica*

**DOI:** 10.1264/jsme2.ME17138

**Published:** 2018-03-29

**Authors:** Dilruba Sharmin, Yong Guo, Tomoyasu Nishizawa, Shoko Ohshima, Yoshinori Sato, Yusuke Takashima, Kazuhiko Narisawa, Hiroyuki Ohta

**Affiliations:** 1 Ibaraki University College of Agriculture, Department of Bioresource Science Ibaraki 300–0393 Japan; 2 United Graduate School of Agricultural Science, Tokyo University of Agriculture and Technology Fuchu, Tokyo 183–8509 Japan; 3 Center for Conservation and Restoration Techniques, Tokyo National Research Institute for Cultural Properties Tokyo 110–8713 Japan

**Keywords:** genome, endohyphal symbiont, interaction, *M. cysteinexigens*, *B. rhizoxinica*

## Abstract

Endohyphal bacteria (EHB), dwelling within fungal hyphae, markedly affect the growth and metabolic potential of their hosts. To date, two EHB belonging to the family *Burkholderiaceae* have been isolated and characterized as new taxa, *Burkholderia rhizoxinica* (HKI 454^T^) and *Mycoavidus cysteinexigens* (B1-EB^T^), in Japan. Metagenome sequencing was recently reported for *Mortierella elongata* AG77 together with its endosymbiont *M. cysteinexigens* (Mc-AG77) from a soil/litter sample in the USA. In the present study, we elucidated the complete genome sequence of B1-EB^T^ and compared it with those of Mc-AG77 and HKI 454^T^. The genomes of B1-EB^T^ and Mc-AG77 contained a higher level of prophage sequences and were markedly smaller than that of HKI 454^T^. Although the B1-EB^T^ and Mc-AG77 genomes lacked the chitinolytic enzyme genes responsible for invasion into fungal cells, they contained several predicted toxin-antitoxin systems including an insecticidal toxin complex and PIN domain imposing an addiction-like mechanism essential for endohyphal growth control during host colonization. Despite the different host fungi, the alignment of amino acid sequences showed that the HKI 454^T^ genome consisted of 1,265 (32.6%) and 1,221 (31.5%) orthologous coding sequences (CDSs) with those of B1-EB^T^ and Mc-AG77, respectively. This comparative study of three phylogenetically associated endosymbionts has provided insights into their origin and evolution, and suggests the later bacterial invasion and adaptation of B1-EB^T^ to its host metabolism.

‘The swollen hypha with two characteristic filiform appendages at its tip’ was the first report of the presence of an endobacterium in the cytosol of Glomeromycotina spores (44). Further morphological confirmation was obtained in subsequent studies (6, 7), and the presence of endobacteria then became widely evident in fungal lineages (14, 60). Recent studies revealed that bacteria and fungi interact at different levels in the bacterial-fungal interface, such as the bacterial slack attachment with the hyphal surface or metabolic complementation with associates and alterations to partners of various forms ranging from antibiosis and signaling to genetic exchange (20, 25, 57). However, endohyphal symbionts residing inside fungal cells exemplify the most intimate illustration of congenial interactions. Despite their genetic and functional diversities, bacterial-fungal interactions have become a new interest in microbiology with significance for microbial ecology (20).

Bacterial endohyphal symbionts are best characterized by the family *Burkholderiaceae* (*Burkholderia*-related) in the class *Betaproteobacteria* and frequently occur in the fungal phyla of Mucoromycota (63). Ascomycota and Basidiomycota are also known to host gammaproteobacteria (2) and alphaproteobacteria (61), respectively. Two endohyphal bacterium (EHB) belonging to the family *Burkholderiaceae* were isolated as new taxa, *Burkholderia rhizoxinica* (HKI 454^T^) and *B. endofungorum* (HKI 456^T^); however, only *B. rhizoxinica* HKI 454^T^ has been characterized in detail (53). An interesting multilateral microbial interaction involving *B. rhizoxinica* HKI 454^T^ has been shown to cause rice seedling blight and the causative agent is the Mucoromycotinan fungus *Rhizopus microsporus*. Phytopathogenic *R. microsporus* used an antimitotic agent, rhizoxin, as a virulence factor (59, 67). It later became evident that this virulence factor was not produced by *R. microsporus* itself, but by the EHB, *B. rhizoxinica* HKI 454^T^ (53, 54). *B. rhizoxinica* controls the vegetative reproduction of the host (31). A previous study isolated *B. rhizoxinica* HKI 454^T^ together with its host strain *R. microsporus* van Tieghem var. *chinensis* (ATCC 62417) from rice seedlings in Japan (54). Recent findings have characterized several strains of the non-pathogenic fungus *Mortierella elongata* (Mortierellomycotina) hosting a *Burkholderia*-related obligate endohyphal symbiont named B1-EB^T^ (58), which was recently characterized as *Mycoavidus cysteinexigens*, gen. nov., sp. nov., based on its phenotypic, chemotaxonomic, and phylogenetic features (49). The basic function and characteristics of B1-EB^T^ as a fungal endosymbiont currently remain unknown. An isolate of *M. elongata* (AG77) from a soil/litter sample close to a poplar tree in Duke forest, the USA was revealed to host *M. cysteinexigens* and it was attained through metagenome sequencing of the host *M. elongata* AG77 together with the endobacterium *M. cysteinexigens* (67). A large and growing endobacterial population of *M. cysteinexigens* AG77 has been observed under nitrogen limiting conditions, whereas *M. elongata* AG77 exhibited decreased growth (38).

Bacterial endosymbionts of fungi are widespread, and extremely different microbial populations may co-exist in the same host fungal strain. For example, rod-shaped Gramnegative *‘Candidatus* Glomeribacter gigasporarum’ (*Ca*Gg) and coccoid Gram-positive *Mollicutes*-related endobacteria (Mre) co-exist in a single spore of the arbuscular mycorrhizal fungus *Gigaspora margarita* (13). Diversity in microbes is influenced by many different factors such as vertical and horizontal gene transfer, the activity of mobile genetic elements, the recombination machinery, and mutation rate (46, 47, 66). *B. rhizoxinica* HKI 454^T^ is the rhizoxin-producing EHB of the phytopathogen *R. microsporus* and causes blight symptoms in rice seedlings, whereas *M. cysteinexigens* B1-EB^T^ is the endohyphal symbiont of the non-pathogenic fungus *M. elongata*. Despite diverse host specificity and modes of interaction with respective hosts, *B. rhizoxinica* and *M. cysteinexigens* have a close phylogenetic relationship (49, 67). Although *Ca*Gg is the most phylogenetically related to *M. cysteinexigens*, the *Ca*Gg genome was incompletely sequenced, which may have been due to approximately 7% DNA contamination by the fungal host *G. margarita* (22). In addition, the coexisting endofungal bacteria Mre and other *Ca*Gg species were potentially contained in the spore lysates of *G. margarita* used to concentrate the endobacterial fraction (13, 42), which produced a number of contigs or induced big gaps in the subsequent genome assembly. Although the draft genome of *M. cysteinexigens* B1-EB^T^ was sequenced in our previous study (21), information regarding structure, mobile genetic elements, rRNA operons, and tRNAs was still inconclusive. Therefore, in the present study, we elucidated the complete genome sequences of *M. cysteinexigens* B1-EB^T^ by sequencing the DNA extract from culture collection and comparing it with the complete genome of the uncultured endosymbiont of *M. elongata* AG77 (named here as Mc-AG77), and also with that of the cultured *B. rhizoxinica* HKI 454^T^ as a reference. Comparative genomic analyses of the three phylogenetically associated endosymbionts; *M. cysteinexigens* B1-EB^T^, Mc-AG77, and *B. rhizoxinica* HKI 454^T^, in two different fungal hosts may provide insights into their origin and evolution as well as potential host-bacterium interactions.

## Materials and Methods

### DNA extraction and genome sequencing

Strain B1-EB^T^ was incubated on cysteine-containing buffered charcoal-yeast extract agar (B-CYEα) as described in our previous study (49). Colonies were suspended in sterile water and recovered by centrifuging at 12,000×*g* for 2 min. The DNA of strain B1-EB^T^ was extracted using the lysozyme buffer method (69). Genomic DNA (21.8 μg) was used to prepare a sequencing library with a SMRTbell Template Prep Kit (Pacific Biosciences of California, Menlo Park, CA, USA). Genome sequencing was performed by Macrogen Korea using PacBio RSII single-molecule real-time (SMRT) sequencing technology (Pacific Biosciences of California).

### PacBio read assembly and genome completing

The long single pass reads (122,714 reads with 1,069,198,690 bases in total) obtained from 1 SMRT cell were assembled using a PacBio *de novo* assembler optimized for the speed Hierarchical Genome Assembly Process (HGAP, v3.0) (9). Three credible contigs were generated: contig 1, 1,355,844 bp with 282-fold coverage; contig 2, 1,037,856 bp with 293-fold coverage; and contig 3, 398,059 bp with 102-fold coverage. These three contigs were combined with the previous draft genome contigs sequenced by the Roche 454 Titanium system (21) by aligning them using the sequence assembler ATGC ver. 4.2 (Genetyx Co., Tokyo, Japan), resulting in a completely circular genome sequence. The positions of 454 contigs in the complete genome were visualized using a CIRCOS circular visualization data tool (ver. 0.69) (30).

### Multilocus sequence analysis

Five housekeeping genes (*atpD*, *gyrB*, *lepA*, *recA*, and *rpoB*) and the 16S rRNA gene sequence were selected for a multilocus sequence analysis (MLSA) as described previously (17), but with slight modifications. In the present study, nine major members (*Cupriavidus necator* N-1^T^, *C. pinatubonensis* JMP134, *Pandoraea apista* DSM 16535, *P, pulmonicola* DSM 16583, *B. diffusa* RF2- non-BP9, *B. cepacia* ATCC 25416, *B. psudomallei* K96243, and *B. thailandensis* E264^T^) belonging to the family *Burkholderiaceae* with completely sequenced genomes, and two species (*Ralstonia pickettii* 12D^T^ and *R. solanacearum* GMI1000) within the family *Ralstoniaceae*, were selected as the interspecific references and *Polynucleobacter necessarius* subsp. *necessaries* STIR1 as outgroup, respectively, in the MLSA. Briefly, multiple sequence alignments were inferred for each dataset using MAFFT (ver. 7.222). MEGA (ver. 7) was used to modify the alignment scale and change the PHYLIP multiple sequence alignment format. The multiple alignment file and module selection of nucleotide substitution were combined using Kakusan version 4. Among-site rate variations were modeled by a GAMMA distribution with four rate categories. Tree searches were initiated using Randomized Axelerated Maximum Likelihood (RAxML) based on the concatenated dataset (12,492 and 11,023 positions in total with/without 16S rRNA, respectively) of the five housekeeping genes and 16S rRNA. Only strains with a full set of sequenced genes were compared in this analysis. Multilocus phylogenetic trees were visualized using the program FigTree (ver. 1.4.3) (49).

### Genome annotation and general comparison

The complete genome sequence of strain B1-EB^T^ was annotated automatically using Microbial Genome Annotation Pipeline (MiGAP) including MetaGeneAnnotator (23) for identifying protein coding sequences (CDSs), tRNAscan-SE (39) for tRNAs, and RNAmmer (35) for rRNAs. GC-skew and GC-content were calculated using a custom Perl script. In order to confirm the annotation of CDSs, the predicted protein sequences were annotated manually using BLASTP with the NCBI-nr database and KEGG Orthology and Links Annotation (KOALA) (26). PHAge Search Tool Enhanced Release (PHASTER) was used to identify prophage sequences within the genome (3, 71). Detailed pan-genome analyses were performed at the protein level using a bidirectional best-hit (BDBH) algorithm implemented in GET_HOMOLOGUES (ver. 2.0.23), imposing a minimum pairwise alignment coverage of 75.0% with default parameters (11) and visualized using the CIRCOS circular visualization data tool (ver. 0.69) (30).

### Nucleotide sequence accession number

The genome sequence of *M. cysteinexigens* B1-EB^T^ has been deposited in the DDBJ/EMBL/GenBank databases under the accession number AP018150. The complete genome sequences of *B. rhizoxinica* HKI 454^T^ and *M. cysteinexigens* AG77 were obtained from NCBI RefSeq and GenBank (Supplemental Table 7, Reference 68), respectively.

## Results and Discussion

### General characteristics of the complete *M. cysteinexigens* B1-EB^T^ genome

**Fig. 1:**
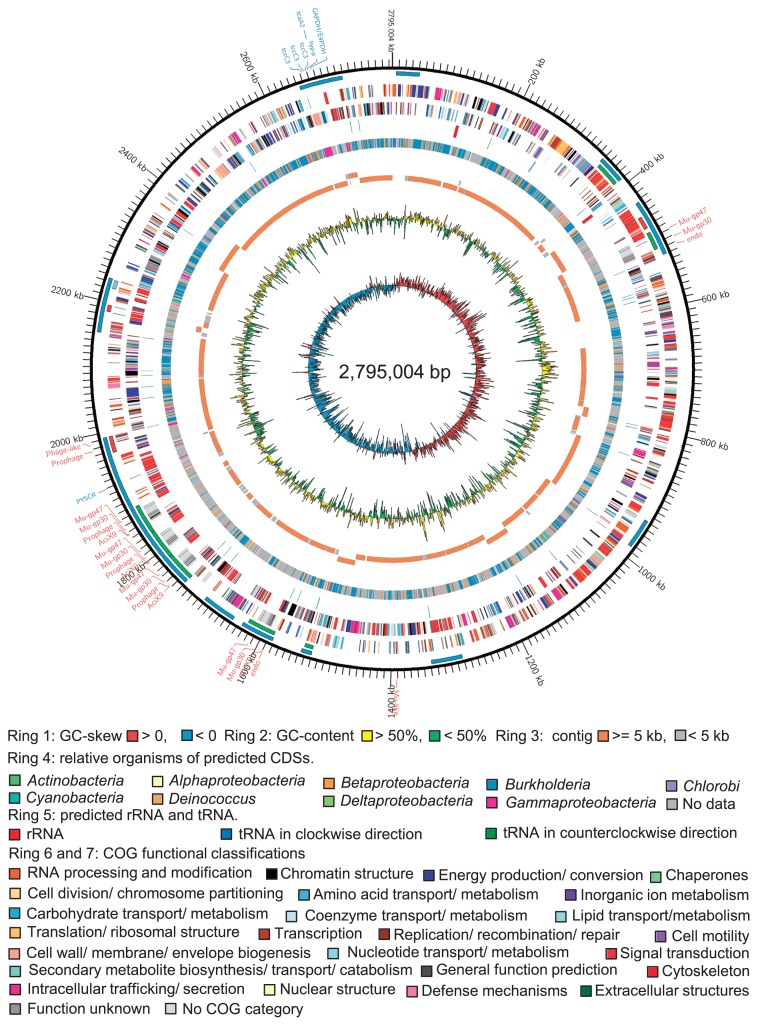
Schematic spherical illustration of the B1-EB^T^ genome. Circles display from the inside to the outside as follows: (ring 1) GC-skew (G-C/G+C ratio) using a 1-kb window; (ring 2) GC-content using a 1-kb window; (ring 3) 454-contigs; (ring 4) relative bacteria of predicted CDSs; (ring 5) rRNAs and tRNAs; (rings 6 and 7) predicted CDSs transcribed in a counterclockwise/clockwise direction; (ring 8) intact, questionable, and incomplete prophage sequences are indicated in blue, green, and red solid bars, respectively; (ring 9) transposons; and (ring 10) scale in kb. Red and blue text outside shows the positions of phage-related genes and insecticidal toxin proteins, respectively.

The complete B1-EB^T^ genome consisted of a 2,795,004-bp circular chromosome with a G+C content of 48.9%, which contained two sets of ribosomal RNA (*rrn*) operons, 41 tRNAs, and 2,317 predicted CDSs (Fig. 1). This result was approximately in accordance with our studied draft genome of strain B1-EB^T^ (21), revealing a genome size of 2,653,566 bp with a G+C content of 46.1%, 1 set of *rrn* operons, 39 tRNAs, and 2,390 CDSs. Most of the CDSs were identified as well conversed with CDSs in bacteria within the genus *Burkholderia* (Fig. 1). Of note, the CDSs encoding insecticidal toxin proteins, which are of potential benefit to the fungal host, were close to those conserved by *Xenorhabdus* (*Gammaproteobacteria*), a genus living symbiotically with soil entomopathogenic nematodes.

**Fig. 2:**
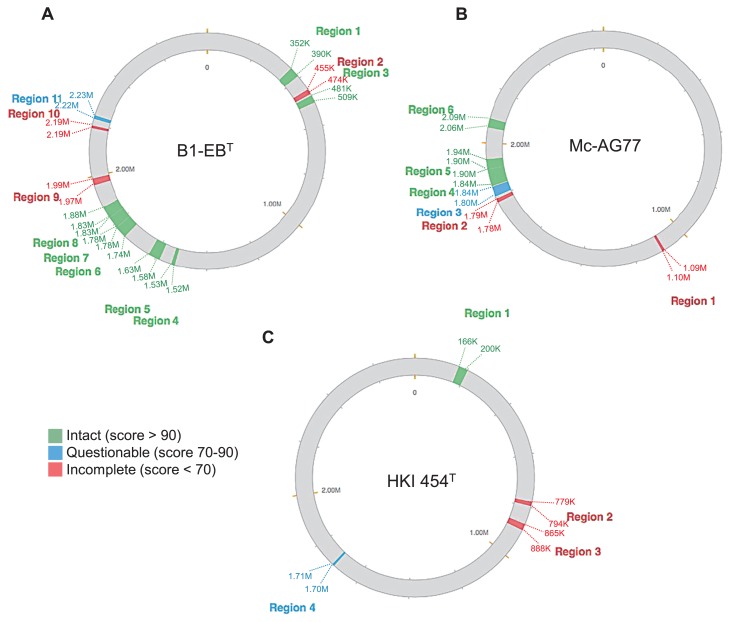
Prophage sequences within B1-EB^T^ (A), Mc-AG77 (B), and HKI 454^T^ (C) genomes. The details of each region are shown in Fig. S1. Position-based identification of prophage region is shown in colors. The scale bar is in bp.

Nevertheless, the annotation of the complete genome indicated that B1-EB^T^ lacks cysteine biosynthetic pathways, as reported previously (21). In addition, the B1-EB^T^ genome had conserved 11 transposons, 7 intact prophages, and 1 questionable and 3 incomplete prophages. Six of these transposons overlapped with the 11 prophage sequences (Fig. 1) encoding prophage-related proteins such as phage integrase and phage transposase (Fig. 2A and S1), suggesting that these regions were acquired by prophage insertion. Besides transposons, there were several retron-type reverse transcriptase and phage proteins. The genetic evidence supporting the introduction to foreign DNA contrasts endosymbionts such as *Buchnera* sp. APS, which was harbored by *Acyrthosiphon pisum* (pea aphid) (43, 62). The B1-EB^T^ genome also contained several predicted toxin-antitoxin systems including the insecticidal toxin complex RelE/StbE, HipA, MazF, and PIN domain proteins encoding genes. These features imposed an addiction-like mechanism on the prokaryotic host genome and arbitrated the stability of mobile elements (1). In addition to their genetic addiction, these factors have been anticipated to facilitate growth retardation under stress conditions (51). Therefore, toxin-antitoxin systems may be essential for endohyphal growth control during host colonization. The B1-EB^T^ genome also included 358 hypothetical proteins. We hypothesized that some strain-specific genomic components of unknown function may correlate with the evolution of the endofungal lifestyle.

### Phylogenetic analysis of *M. cysteinexigens* and related taxa within the family *Burkholderiaceae*

**Fig. 3:**
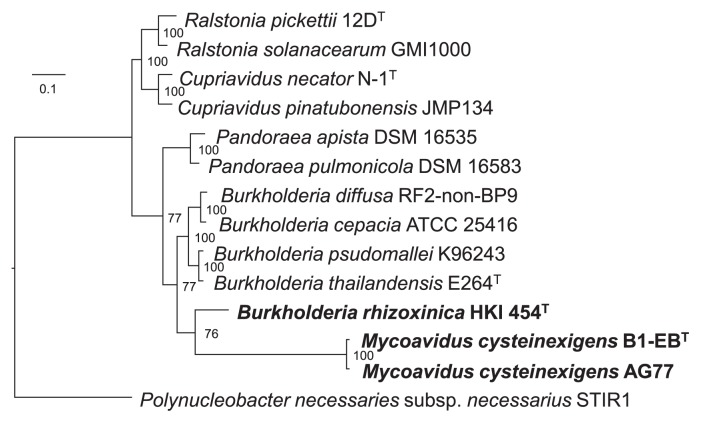
Randomized axelerated maximum likelihood (RAxML) tree based on concatenated sequences (11,023 positions in total) of five housekeeping genes (*atpD*, *gyrB*, *lepA*, *recA*, and *rpoB*) indicating the relative placement of the three endofungal bacteria (bold) and other genera in the families *Burkholderiaceae* and *Ralstoniaceae*. Pairwise distance between B1-EB^T^ and Mc-AG77 is 0.013. The horizontal lines show genetic distances, which are supported by values estimated with 100 bootstrap replicates. The scale bar indicates the number of substitutions per nucleotide position.

In the present study, we generated a multilocus phylogenetic tree of 14 reference strains (including 4 type strains) of *Burkholderiaceae* using the *atpD*, *gyrB*, *lepA*, *recA*, and *rpoB* genes in combination with the 16S rRNA gene sequence and employed RAxML with support values estimated with 100 bootstrap replicates for the evolution of the genus *Mycoavidus* as a sister clade to *Burkholderia* (Fig. 3 and S2). The output of this analysis also showed a distinct clade for *B. rhizoxinica*, which was positioned within the genus *Burkholderia*. Based on phylogenomics and the separation of fungal hosts, two alternative scenarios, ‘early’ and ‘late’ bacterial invasion, were hypothesized (5). Regarding *Burkholderia*-related endobacteria, early bacterial invasion estimated the existence of an ancestral free-living *Burkholderia*-related endobacteria that invaded the common ancestor of Glomeromycotina and Mortierellomycotina (5, 33). As discussed by Bonfante & Desirò (2017) (5), late bacterial invasion may have occurred when the evolutionary lines leading to Glomeromycotina and Mortierellomycotina had already separated. However, *B. rhizoxinica* appeared to have an independent origin shared with the free-living *Burkholderia*-related endobacteria that diverged from the ancestral lineage (34). This hypothetical scenario may lead to further discussions on whether specific bacterial-fungal interactions occurred in the common ancestor only (not separated) and not in separated Glomeromycotina and Mortierellomycotina in which obligate endobacteria essentially lack the ability to grow independently of their fungal hosts.

### Comparative genomics of EHB belonging to *M. cysteinexigens* and *B. rhizoxinica*

**Fig. 4:**
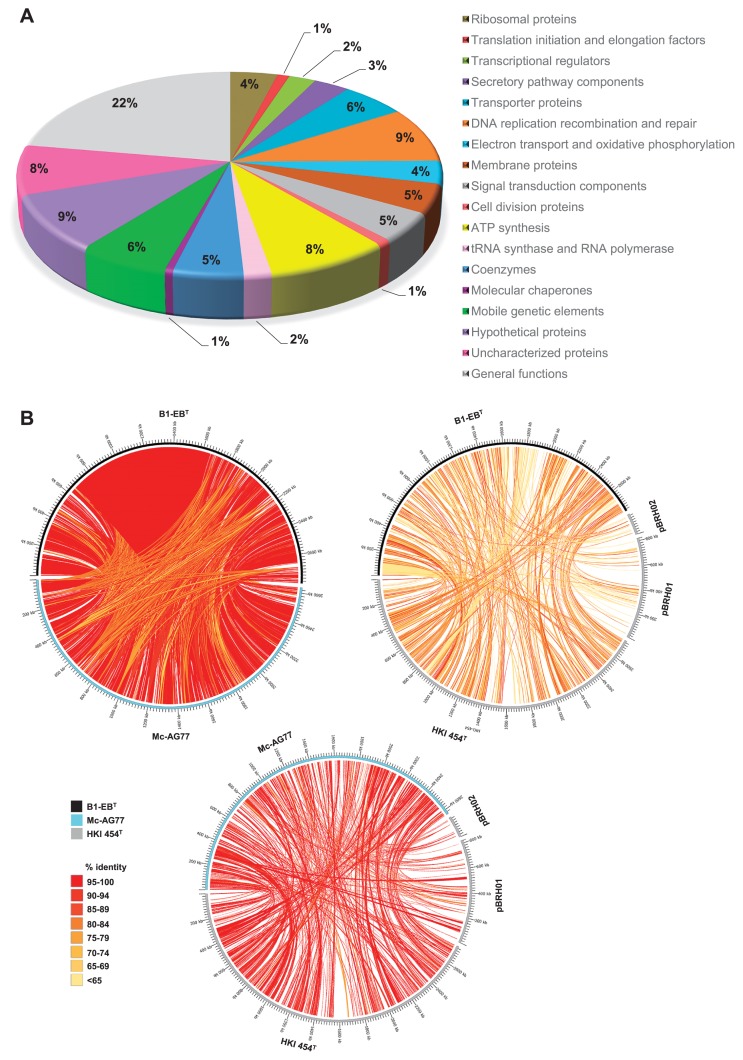
Genome comparison of three beta-proteobacterial endohyphal symbionts. (A) Pie chart showing the distribution (% shared proteomes) of ortholog clusters (BDBH) based on % identity among the proteomes of *Mycoavidus* and *B. rhizoxinica* genomes. (B) Comparative mapping showing links among homologous sequences. The circular exterior displays the genomes of organisms clockwise. Ribbons between genomes indicate regions of shared homology based on the % identity of amino acid pairs between each pair of organisms. Darker colors correspond to higher identity links and the lighter color corresponds to a lower identity (see legends).

In order to obtain insights into the origin and evolution of gene families characteristic of the phylogenetically associated endohyphal symbionts in diverse fungal hosts, we conducted comparative genome analyses on *M. cysteinexigens* B1-EB^T^, Mc-AG77, and *B. rhizoxinica* HKI 454^T^ by the computation of pairwise homologous gene clusters. We excluded *Ca*Gg from this comparative analysis because of its controversial genome sequence, which we clarified earlier in this study. A previous study reported that the total genome of *B. rhizoxinica* HKI 454^T^ is 3,750,139 bp, consisting of the chromosome and two plasmids, pBRH01 and pBRH02 (33). The entire genome comprises 3,878 CDSs, 2,437 of which were estimated to have biological functions, 1,441 had no known function, 897 appeared to be similar to other database entries, and 544 remained without function and similarity (34). The genome of Mc-AG77 is 2,638,116 bp and contained 2,255 CDSs (68). Both bacterial genomes were markedly smaller than that of *B. rhizoxinica* HKI 454^T^ and harbored fewer transcriptional regulator genes (Table 1). Pan-genome GET_HOMOLOGUES analyses at the protein level using the BDBH algorithm resulted in the extraction of 1,266 and 1,221 ortholog clusters with shared homology to the HKI 454^T^ genome based on the % identity of amino acid pairs with B1-EB^T^ and Mc-AG77, respectively, whereas 1,788 homologous regions with high % identity were predicted between B1-EB^T^ and Mc-AG77. We herein summarized the distribution (% shared proteomes) of ortholog clusters based on the % identity among the proteomes of *Mycoavidus* and *B. rhizoxinica* genomes (Fig. 4A). Pairwise comparative mapping of the B1-EB^T^, Mc-AG77, and HKI 454^T^ genomes displayed regions of shared homology (Fig. 4B). Although the MLSA of 14 reference strains of *Burkholderiales* using five housekeeping genes and the 16S rRNA gene revealed that *M. cysteinexigens* B1-EB^T^ and Mc-AG77 both had a similar distance with *B. rhizoxinica* HKI 454^T^ (Fig. 3 and S2), pairwise comparative mapping displayed the high homology of Mc-AG77 with *B. rhizoxinica* HKI 454^T^. The B1-EB^T^ genome was disrupted by a large amount of mobile genetic elements, with 146 CDSs (6.3%) encoding proteins with similarities to transposases or inactivated derivatives, plasmid stability proteins, reverse transcriptases, and phage proteins, which was similar to HKI 454^T^ (Table S1); however, it is currently unclear how many of these were active insertion elements. We also predicted 271 (11.6%) characterized proteins, 137 hypothetical proteins, and 51 transposase and inactivated derivatives in B1-EB^T^, which were non-homologous with Mc-AG77 and HKI 454^T^ (Table S2). Since the annotations of Mc-AG77 CDSs are not yet known, we attempted to predict their functions from homology shared with the B1-EB^T^ genome.

### Prophage sequences, horizontal gene transfer, and genome reductions in *M. cysteinexigens* and *B. rhizoxinica*

Genomes of obligate intracellular bacteria are often categorized by high levels of genetic drift, genome streamlining, and a lack of mobile DNA (plasmid, transposon, and prophage), which emerge with prominent degrees of stability and gene synteny (8, 36). However, a number of studies have showed that the smaller genomes of obligate intracellular species comprise a level of prophage sequences (*e.g.*, the prophage sequence of *Wolbachia pipientis* is less than 2.0% of the total genome) similar to the lower end of the range found in facultative intracellular species (8). We herein identified the prophage sequence in the genomes of EHB *M. cysteinexigens* B1-EB^T^, Mc-AG77, and *B. rhizoxinica* HKI 454^T^ by PHASTER. Of note, the chromosomes of B1-EB^T^, Mc-AG77 comprised a markedly higher level of prophage sequences than those in the HKI 454^T^ chromosome. The prophages and their remnants in B1-EB^T^ and Mc-AG77 accounted for 11.5% (11 regions with 267 phage-related CDSs) and 7.2% (6 regions with 133 phage-related CDSs) of the total lengths of their chromosomes, respectively (Fig. 2A and B), whereas that of HKI 454^T^ was only 2.9% (4 regions with 79 phage-related CDSs, Fig. 2C). A longer association with a host resulted in the endobacterial genomes undergoing a mutational bias toward a lower GC content (12, 40, 43). The GC contents of prophage sequences in B1-EB^T^ and Mc-AG77 chromosomes (prophage GC contents, 49.2% and 48.4%, respectively) were closer to those of their chromosomes (Table 2), whereas an apparently lower GC content (58.6%) was observed for the prophage sequences in the HKI 454^T^ chromosome (chromosome GC content, 61.2%) (Fig. 2C). This result suggests a relatively longer association of prophages with *M. cysteinexigens* hosts than those with *B. rhizoxinica* HKI 454^T^.

As an important means for gene-gain events, prophages horizontally transfer genes from one bacterial genome to another. More than 70% of phage-related CDSs in the genomes of *M. cysteinexigens* were close to those CDSs of the bacteriophages infecting *Gammaproteobacteria* such as *Aggregatibacter actinomycetemcomitans*, *Edwardsiella tarda*, and *Escherichia coli*, followed by those of phage-infecting bacteria of *Betaproteobacteria*, *Alphaproteobacteria*, *Firmicutes*, *Cyanobacteria*, *Bacteroidetes*, *Actinobacteria*, and *Tenericutes* (Mollicutes) (Table 2 and S3). In contrast, most phage-related CDSs in the *B. rhizoxinica* HKI 454^T^ chromosome (72.2%) were similar to those found in the phages infecting betaproteobacteria, particularly *B. cenocepacia*, *B. cepacia*, and *B. thailandensis*, followed by the CDSs related to the phages infecting bacteria of *Gammaproteobacteria* (22.8%) and *Firmicutes* (5.0%) (Table 2 and S3). This phage originated by distinction, suggesting a difference in horizontal gene transformation between the two lineages. However, many researchers have shown that the smaller genomes of obligate intracellular bacteria preferred the inactivation and deletion of prophage sequences over their insertion and duplication (8, 36). An analysis using PHASTER showed that only one prophage region in the *B. rhizoxinica* HKI 454^T^ chromosome was intact, which was consistent with the deletion of the prophage in the intracellular bacterial genome. Nevertheless, *M. cysteinexigens* had more intact prophages in their chromosomes (7 in B1-EB^T^, and 3 in Mc-AG77), indicating that prophages in *M. cysteinexigens* genomes were undergoing deletion and inactivation processes. In consideration of the relatively longer association of prophages with *M. cysteinexigens*, the deletion rate of prophages in the genomes of *M. cysteinexigens* appeared to be slower than those proceeding in the genome of *B. rhizoxinica*, suggesting that the prophages still possess physiological functions in *M. cysteinexigens*. However, the lengths of non-prophage regions in the chromosomes of B1-EB^T^, Mc-AG77, and HKI 454^T^ were 2,469,125 bp, 2,447,528 bp, and 2,674,052 bp, respectively. Therefore, the primordial genetic elements in the *M. cysteinexigens* genome were presumed to be more reduced than those in the HKI 454^T^ chromosome if the two endohyphal bacteria were derived from a common ancestor bacterium.

### Metabolic potential and nutrient uptake in *M. cysteinexigens* and *B. rhizoxinica*

**Fig. 5:**
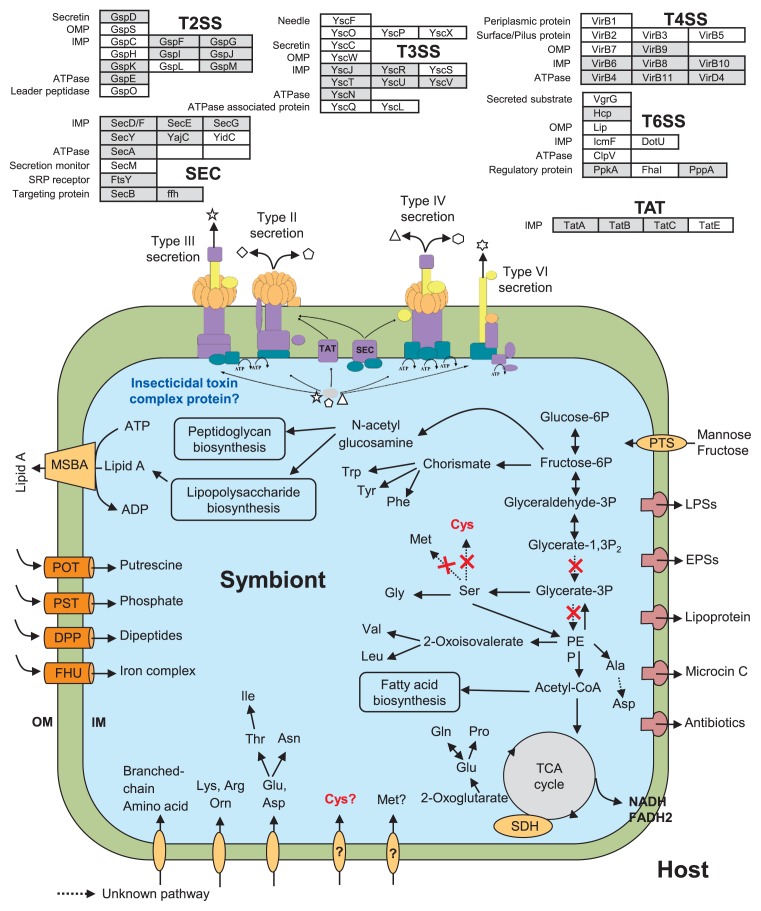
Model representing metabolic processes and the secretion system assumed from the B1-EB^T^ genome. Putative type II, III, IV, and VI secretory proteins are highlighted in grey. POT, polyamine transport system; PST, phosphate-specific transport; DPP, dipeptide permease; FHU, ferrichrome-uptake; EPSs, exopolysaccharides; LPSs, lipopolysaccharides; IM, inner membrane; OM, outer membrane; OMP, outer membrane protein; IMP, inner membrane protein; SEC, general secretion pathway; TAT, twin arginine translocation pathway.

There has always been a primary interest in the metabolic potential of EHB, whether they are nutrient producers or consumers and how they exchange nutrients and cofactors with their fungal hosts. We compared the primary metabolism, cofactor biosynthesis, and membrane transport of B1-EB^T^ and Mc-AG77 with HKI 454^T^ (Table 3) and created a model of metabolic processes in *M. cysteinexigens* (Fig. 5). The deduction of primary metabolism and transporters suggested that *M. cysteinexigens* (B1-EB^T^ and Mc-AG77) possess genes for glucose/maltose/*N*-acetylglucosamine- and mannose/fructose-specific phosphotransferase system components, but do not utilize glucose, arabinose, mannose, mannitol, *N*-acetyl-d-glucosamine, maltose, or gluconate. It was already known that HKI 454^T^ prefers glycerol over glucose as a carbon source (53). HKI 454^T^ may consume organic acids such as citrate, malate, glycerol, and ethanol from *Rhizopus* (41), whereas no genes encoding ethanol assimilation or organic acid importers were detected in the B1-EB^T^ genome (Table 3 and S1), suggesting that B1-EB^T^ cannot utilize these organic acids from its host.

*M. cysteinexigens* and *B. rhizoxinica* both harbor genes responsible for importing branched-chain and aromatic amino acids, histidine, acidic amino acids, and glycine; the biosynthetic routes for all proteinogenic amino acids and efflux systems for basic amino acids (Table S4) (68). Additionally, both endosymbionts had genes for dipeptide and oligopeptide import systems. However, B1-EB^T^ and Mc-AG77 do not possess efflux systems for cysteine. B1-EB^T^ possesses genes encoding biosynthesis enzymes for essential cofactors such as pyridoxine, heme, flavin, biotin, and thiamine, which were similar to those in HKI 454^T^. HKI 454^T^ may provide some amino acids and cofactors to its host (34), which suggests that amino acid metabolism and transportation systems adapted during endobacterium-host symbiosis in B1-EB^T^ and HKI 454^T^.

### Secondary metabolites, lipopolysaccharides, exopolysaccharides, and efflux pumps in *M. cysteinexigens* and *B. rhizoxinica*

The genomic locus for antimitotic rhizoxin biosynthesis in HKI 454^T^ revealed a hybrid system of non-ribosomal peptide synthetase (NRPS) and polyketide synthase (PKS) (52). The NRPS/PKS system architecture in HKI 454^T^ possessed a phosphopantetheinyl transferase to activate the acyl and peptidyl carrier protein domains, 14 NRPS multimodular enzymes, and macrolide-specific ABC-transporters for toxin delivery to the host fungus (34). Since no peptides corresponding to these gene clusters were isolated from HKI 454^T^, it was hypothesized that some NRPS products may function as siderophores or antibiotics (34). *M. cysteinexigens* B1-EB^T^ contained a NRPS module (MCB1EB_1072), which was an ortholog of the adenylation domain in the first NRPS module of bacillibactin present in pBRH01 (RBRH_00588) in *B. rhizoxinica* HKI 454^T^. On the other hand, this NRPS module in B1-EB^T^ shared strong identity (97%) with *Glomeribacter* sp. 1016415. However, its function and biosynthetic potential remain unclear. Virulence factors help bacteria to invade their hosts, which were completely functional in EHB HKI 454^T^. Therefore, the mechanisms by which B1-EB^T^ invaded its host fungus without a complete virulence system have not yet been elucidated. Although bacterial intrusion often depends on genetic characteristics, the invasion mechanism strictly differed with the environment encountered by the bacterium (56) and with different life history policies such as avirulent microorganisms, latent pathogens, and virulent pathogens in the early stages of infection or at a certain phase of their development (28). A single mutation was shown to result in the loss of the virulence factor, transforming the pathogenic fungus, *Colletotrichum magna*, into an endophyte (19). Therefore, virulence-related genes may be lost during bacterial genome reduction in B1-EB^T^. We speculated how *M. cysteinexigens* may invade host fungal tissues, such as when fungal hyphae are damaged by other fungi or by grazing soil fauna. Subsequent invasion occurred through breaks in the fungal cell wall. In contrast, *B. rhizoxinica* HKI 454^T^ invades the host, even under laboratory conditions (34).

Lipopolysaccharides (LPSs) and exopolysaccharides (EPSs) play key roles in the recognition and stability of the symbiosis of *B. rhizoxinica* HKI 454^T^ within its host and a gene cluster involved in LPS biosynthesis in HKI 454^T^ (37). Besides LPS biosynthesis, genome sequencing revealed genes that enabled HKI 454^T^ to also produce EPSs (34). The biosynthesis of the homopolymeric O-antigen LPS depends on an ABC transporter (70). B1-EB^T^ and Mc-AG77 genomes identified an inner membrane ABC transporter, MsbA, similar to the macrolidespecific ABC transporter in HKI 454^T^ and a glycosyltransferase gene (Table S1), which indicates that only these two components of the carbohydrate-derived putative LPS/EPS system of the recognition process are present in *M. cysteinexigens*.

### Secretion systems and insecticidal toxin complexes in *M. cysteinexigens* and *B. rhizoxinica*

Secretory proteins pass through the cell membrane in bacteria, either via the general secretion pathway (4, 15) or twin arginine translocation pathway (50), and regulate host control mechanisms. Therefore, in the present study, we analyzed the genes coding for putative protein secretion systems. The Mc-AG77 genome acquired genes for type II (T2SS), III (T3SS), and IV (T4SS) secretion systems, and these contributed to the exchange of proteins and DNA with its host (68). Additionally, the B1-EB^T^ genome possesses a few components of a type VI secretion system (T6SS).

T2SS is known to be involved in the secretion of toxins and lytic proteins (*e.g.*, chitinolytic enzymes, chitin-binding proteins) outside the cell in various bacteria (10, 18). In addition, microbial chitinases are presumed to degrade hyphal walls and allow bacteria to invade fungi. The betaproteobacterium *Nitrosomonas europea* appears to have significant, but incomplete T2SS core components (10). *B. rhizoxinica* HKI 454^T^ possessed a complete T2SS, whereas B1-EB^T^ possessed a partial T2SS constituent, which is homologous with that in HKI 454^T^ (Table S5). It currently remains unclear whether T2SS homologs in diverse bacteria are expressed and functional. *B. rhizoxinica* and other free-living, pathogenic *Burkholderia* species (*e.g*. *B. thailandensis* and *B. pseudomallei*) contain *hrp* T3SS gene clusters and these are known to play a significant role in symbiosis through the translocation of effector proteins with their hosts (32, 64). B1-EB^T^ possessed a putative T3SS constituent with partial homology with the HKI 454^T^ T3SS pathway (Table S6). In contrast, the T3SS components of Mc-AG77 shared strong homology with *Gammaproteobacteria* such as *Salmonella* and *Yersinia* (68).

The T4SS system was shown to be involved in the plant pathogenicity of *B. cenocepacia* (16). A partial T4SS was found in the B1-EB^T^ genome and in the plasmid pBRH02 of HKI 454^T^ (Table S1). In the B1-EB^T^ genome, we recognized a hypothetical protein homolog type 4 major prepilin protein, pilus assembly pathway ATPase PulE/Tfp homolog type 4 pilus biogenesis protein, a prepilin signal peptidase, and ATPase pilus assembly distributed throughout the chromosome in HKI 454^T^ (Table S1). Adhesive structures, particularly type 4 bacterial pili, are generally major adhesion factors for host surfaces in Gram-negative bacteria (27). However, the pilus structure in B1-EB^T^ lacked several key genes *e.g.*, secretin, motor protein, and other accessory proteins. Moreover, hemolysin gene products are known to be involved in plant pathogenicity (24), and the hemagglutinin-like proteins, putative hemolysin/phospholipase/lecithinase, were also found in the B1-EB^T^ genome and had sequence similarities with RBRH_02330, RBRH_00133, and RBRH_01303 of HKI 454^T^. Additionally, a gene cluster coding for an insecticidal toxin protein (coding genes: CSV86_11535, *tccC3*, PCH70_41490, and *tcaA2*) was found in the homologs of *Xenorhabdus bovienii* SS-2004 and *Photorhabdus asymbiotica* (45, 55). The insecticidal toxin proteins present in the HKI 454^T^ genome were completely different from those present in the B1-EB^T^ genome.

## Conclusions

Compared with animals and plants, the huge genetic diversity of the bacterial biosphere makes it an outstanding resource for studying genetics and evolution. The bacteria that dwell inside eukaryotes contain some of the smallest, most stable, highly repetitive, and recombined bacterial genomes sequences currently known (40, 48). Although bacterial-fungal symbiosis has important implications for agriculture, biotechnology, and food safety, this interaction has long been overlooked (29, 65). The genome of B1-EB^T^ offers an extraordinary picture of an obligate symbiont that has most likely lost its capacity to grow outside of its fungal host (*i.e.*, cannot grow in conventional nutrient media) (12, 21, 49). In the present study, we found that the B1-EB^T^ and Mc-AG77 genomes exhibited amino acid similarities between ~20–84% and ~18–100%, respectively, with HKI 454^T^ (Table S1). Obligate endosymbiotic bacteria were anticipated to have lost many essential genes as a result of genome reduction during evolution (51). In contrast to other fungal endosymbionts in the family *Burkholderiaceae*, both strains of *M. cysteinexigens* lack many genes associated with host invasion such as chitinolytic enzymes (chitinase, chitosanase), chitin-binding proteins, and several T2SS components, combined with sugar importers and the ATP/ADP antiporter, during bacterial genome reduction, suggesting later bacterial invasion and adaptation to its host metabolism.

## Supplementary Material



## Figures and Tables

**Table 1:** Comparative genome analysis of *M. cysteinexigens* B1-EB^T^, Mc-AG77, and *B. rhizoxinica* HKI 454^T^.

Organism	Genome size (bp)	G+C content (%)	Coding sequences (CDSs)	Mobile genetic, elements, (% for all CDSs)	Transcriptional, regulators, (% for all CDSs)	References
***M. cysteinexigens* B1-EB^T^**	2,795,004	48.9	2,317	146 (6.3)	39 (1.68)	This research
***M. cysteinexigens* AG77**	2,638,116	49.0	2,255	71 (3.14)**	39 (1.72)***	Uehling *et al.*, 2017 (68)
***B. rhizoxinica* HKI 454^T^**	3,750,139*	60.7	3,870	255 (6.6)	193 (5.0)	Lackner *et al.*, 2011 (34)

* Includes a 2,755,309-bp chromosome, 822,304-bp mega plasmid, and 172,525-bp plasmid.

** Genes coding for mobile genetic elements are predicted from the homologous sequence of the B1-EB^T^ genome.

*** Genes coding for transcriptional regulators are predicted from the homologous sequence of the B1-EB^T^ genome.

**Table 2:** Phage-related CDSs in genomes of *M. cysteinexigens* B1-EB^T^, Mc-AG77, and *B. rhizoxinica* HKI 454^T^.

Bacterial host of relative phage/prophage in	B1-EB^T^		Mc-AG77		HKI 454^T^	
		
	CDS count	Percentage	CDS count	Percentage	CDS count	Percentage
*Actinobacteria*	2	0.75	1	0.75	0	—
*Bacteroidetes*	4	1.50	2	1.50	0	—
*Cyanobacteria*	2	0.75	4	3.01	0	—
*Firmicutes*	9	3.37	7	5.26	4	5.06
*Alphaproteobacteria*	13	4.87	3	2.26	0	—
*Betaproteobacteria*	39	14.61	21	15.79	57	72.15
*Gammaproteobacteria*	197	73.78	95	71.43	18	22.78
*Tenericutes* (Mollicutes)	1	0.37	0	—	0	—

**Total**	**267**	**100.00**	**133**	**100.00**	**79**	**100.00**

**Table 3:** Comparison of metabolic potentials, nutrient uptake and defense responses in B1-EB^T^, Mc-AG77, and HKI 454^T^.

Attributes	B1-EB^T^	Mc-AG77	HKI 454^T^

Primary metabolism			
Gluconeogenesis*	−	−	+
Ethanol assimilation*	−	−	+
Import of organic acids	−	−	+
Amino acid metabolism	+	+	+
Biosynthetic routes for proteinogenic amino acids*	+	+	+
Dipeptide and oligopeptide import system*	+	+	+

**Cofactor biosynthesis**			

Biosynthesis of pyridoxine*, heme*, flavin*, biotin, and thiamine*	+	+	+

**Membrane transport**			

Putative cobalamin transport system*	+	+	+
Fe^2+^, Mg^2+^, Co^2+^, Zn^2+^, K^+^ uptake system	+	+	+
Na^+^/Ca^2+^ antiporter*	+	+	+
Na^+^/citrate symporter*	+	+	+
NRPS/PKS** system for rhizoxin biosynthesis	−	−	+
Lantibibiotic biosynthesis*	−	−	+
Chitinolytic enzymes and chitin-binding proteins*	−	−	+

**Amino acid metabolism**			

Branched-chain amino acid	+	+	+
Aromatic amino acid	+	+	+
Histidine	+	+	+
Glutamate/aspartate	+	+	+
Glycine	+	+	+
Citrate	+	+	+
Biosynthetic routes for proteinogenic amino acids	+	+	+
Efflux systems for arginine, histidine, lysine	+	+	+
Efflux systems for cysteine	−	−	+

**Polar lipids**			

Phosphatidylethanolamine*	+	+	−
Phosphatidylglycerol*	+	+	−
Diphosphatidylglycerol*	+	+	−
Aminophospholipid*	+	+	−
Aminolipids*	+	+	−

**Fatty acid synthesis**			

Acetyl-CoA carboxylase components	+	+	+
Saturated/unsaturated synthase components*	+	+	+
Peripheral enzymes (ACP synthase and biotin ligase) *	+	+	+
Glycerophospholipid metabolism*	+	+	+

**Lipopolysaccharide gene cluster**			

Lipopolysaccharide	−	−	+
O-antigen*	−	−	+
Inner membrane ABC transporter, MsbA*	+	+	+
Transport complex (LptA, LptAB, LptC, LptD, LptE, LptG) *	−	−	+

**Exopolysaccharide/capsular polysaccharide biosynthesis**			

Antigen flippase and polymerase*	−	−	+
Trans-envelope transport complex (Wza/Wzb/Wzc) *	−	−	+
Glycosyltransferases gene*	+	+	+
Mannosyltransferase gene*	−	−	+

* Genes listed in Mc-AG77 are predicted from the homologous sequence of the B1-EB^T^ genome.

** Non-ribosomal peptide synthetase/polyketide synthase
