# Supplementary material for: Comparative Genomic Insights into Endofungal Lifestyles of Two Bacterial Endosymbionts, Mycoavidus cysteinexigens and Burkholderia rhizoxinica
Source: Microbes Environ. 2018 Mar 29;33(1):66–76. doi: 10.1264/jsme2.ME17138 (PMC5877345; doi:10.1264/jsme2.ME17138)
Supplement: Supplementary file 1 [file 33_66_s1.pdf]

Supplemental Table S1. Comparison of the annotation results for ortholog clusters derived from the B1-EB^T and HKI454^T genomes.

B1-EB^T		HKI454^T	
**Gene**	**Function**	**Gene**	**Function**
dnaA	ATPase involved in DNA replication initiation	RBRH_00964	Chromosomal replication initiator protein dnaA
dnaN	DNA polymerase sliding clamp subunit (PCNA homolog)	RBRH_00962	DNA polymerase III, beta chain
gyrB	Type IIA topoisomerase (DNA gyrase/topo II, topoisomerase IV), B subunit	RBRH_00960	DNA gyrase subunit B
-	Retron-type reverse transcriptase	RBRH_02254	Reverse transcriptase
-	Hypothetical protein	RBRH_03054	Unnamed protein product
-	Uncharacterized conserved protein	RBRH_00791	Hypothetical protein
-	Hypothetical protein	RBRH_00790	Unnamed protein product
gidA	NAD/FAD-utilizing enzyme apparently involved in cell division	RBRH_00815	Glucose inhibited division protein A
gidB	Predicted S-adenosylmethionine-dependent methyltransferase involved in bacterial cell division	RBRH_00816	Methyltransferase gidB
parA2	ATPases involved in chromosome partitioning	RBRH_00817	Chromosome partitioning protein parA
parB2	Predicted transcriptional regulators	RBRH_00818	Chromosome partitioning protein parB
atpF0A	F0F1-type ATP synthase, subunit a	RBRH_00821	ATP synthase A chain
atpF0C	F0F1-type ATP synthase, subunit c/Archaeal/vacuolar-type H^+-ATPase, subunit K	RBRH_00822	ATP synthase C chain
atpF0B	F0F1-type ATP synthase, subunit b	RBRH_00823	ATP synthase B chain
atpF1D	F0F1-type ATP synthase, delta subunit (mitochondrial oligomycin sensitivity protein)	RBRH_00824	ATP synthase delta chain
atpF1G	F0F1-type ATP synthase, gamma subunit	RBRH_00826	ATP synthase gamma chain
atpF1B	F0F1-type ATP synthase, beta subunit	RBRH_00827	ATP synthase beta chain
atpC	F0F1-type ATP synthase, epsilon subunit (mitochondrial delta subunit)	RBRH_00828	ATP synthase epsilon chain
fumC	Fumarase	RBRH_01757	Aldose 1-dehydrogenase
hemE	Uroporphyrinogen-III decarboxylase	RBRH_00831	Uroporphyrinogen decarboxylase
putA	Delta 1-pyrroline-5-carboxylate dehydrogenase	RBRH_00839	Proline dehydrogenase / Delta-1-pyrroline-5-carboxylate dehydrogenase
-	ABC-type branched-chain amino acid transport systems, periplasmic component	RBRH_00840	Leucine-, isoleucine-, valine-, threonine-, and alanine-binding protein
PMI16_02037	Predicted Na+-dependent transporter	RBRH_03087	Transporter, Sodium/bile acid symporter family
bioB	Biotin synthase and related enzymes	RBRH_03099	Biotin synthase
bioA	Adenosylmethionine-8-amino-7-oxononanoate aminotransferase	RBRH_03101	Adenosylmethionine-8-amino-7-oxononanoate aminotransferase
mnmC	Uncharacterized conserved protein	RBRH_00787	tRNA (5-aminomethyl-2-thiouridylate) methyltransferase
-	Bacterial nucleoid DNA-binding protein	RBRH_00786	DNA-binding protein HU
gspD	Type II secretory pathway, component PulD	RBRH_00782	General secretion pathway protein D
gspE	Type II secretory pathway, ATPase PulE/Tfp pilus assembly pathway, ATPase PilB	RBRH_00781	General secretion pathway protein E
gspF	Type II secretory pathway, component PulF	RBRH_00780	General secretion pathway protein F
bglu_1g00350	Hypothetical protein	RBRH_00779	Hypothetical protein
BC1003_3493	Type II secretory pathway, pseudopilin PulG	RBRH_00777	General secretion pathway protein H
gspI	Type II secretory pathway, pseudopilin PulG	RBRH_00776	General secretion pathway protein I
BC1003_3495	Type II secretory pathway, component PulJ	RBRH_00775	General secretion pathway protein J
Bcep1808_0062	Type II secretory pathway, component PulK	RBRH_00774	General secretion pathway protein K
Bxe_A4492	Hypothetical protein	RBRH_00773	General secretion pathway protein L
BC1003_3498	Type II secretory pathway, component PulM	RBRH_00772	General secretion pathway protein M
BCh11DRAFT_04780	Hypothetical protein	RBRH_00771	Unnamed protein product
-	Permeases of the major facilitator superfamily	RBRH_03630	Multidrug resistance protein B
-	Outer membrane protein	RBRH_00769	Type I secretion outer membrane protein
C664_08243	5,10-methylenetetrahydrofolate reductase	RBRH_02728	Methylenetetrahydrofolate reductase
metF	S-adenosylhomocysteine hydrolase	RBRH_02726	Adenosylhomocysteinase
ahcY	Amino acid transporters	RBRH_02715	Amino acid permease
-	S-adenosylmethionine synthetase	RBRH_03427	S-adenosylmethionine synthetase
-	Lauroyl/myristoyl acyltransferase	RBRH_03425	Lipid A biosynthesis lauroyl acyltransferase
-	Diaminopimelate epimerase	RBRH_03424	Diaminopimelate epimerase
dapF	Uncharacterized protein conserved in bacteria	RBRH_03423	Hypothetical protein
Bphy_0075	Site-specific recombinase XerC	RBRH_03422	Integrase/recombinase (XerC/CodV family)
xerC	Predicted membrane protein	RBRH_02737	Permease
-	Predicted membrane protein	RBRH_02738	Permease
-	Uncharacterized proteins, homologs of lactam utilization protein B	RBRH_02739	Lactam utilization protein LAMB
-	Allophanate hydrolase subunit 2	RBRH_02740	Regulator of kinase autophosphorylation inhibitor
-	Allophanate hydrolase subunit 1	RBRH_02742	Kinase autophosphorylation inhibitor kipI
-	5-formyltetrahydrofolate cyclo-ligase	RBRH_02745	5-formyltetrahydrofolate cyclo-ligase
CAGGBEG34_20043	Soluble lytic murein transglycosylase and related regulatory proteins (some contain LysM/invasin domains)	RBRH_02746	Soluble lytic murein transglycosylase
-	tRNA nucleotidyltransferase/poly(A) polymerase	RBRH_02749	tRNA nucleotidyltransferase
cca	Uncharacterized conserved protein	RBRH_01965	Hypothetical protein
-	Dihydroneopterin aldolase	RBRH_01963	Dihydroneopterin aldolase
-	Predicted ATPase of the PP-loop superfamily implicated in cell cycle control	RBRH_01962	tRNA s2C32 biosynthesis protein TtcA
-	N-acetylglucosamine-1-phosphate uridyltransferase	RBRH_01961	Glucosamine-1-phosphate acetyltransferase/ UDP-N-acetylglucosamine pyrophosphorylase
glmU	Glucosamine 6-phosphate synthetase, contains amidotransferase and phosphosugar isomerase domains	RBRH_01960	Glucosamine--fructose-6-phosphate aminotransferase [isomerizing]
glmS	Acetylglutamate semialdehyde dehydrogenase	RBRH_00370	N-acetyl-gamma-glutamyl-phosphate reductase
argC-1	Orotate phosphoribosyltransferase	RBRH_00372	Orotate phosphoribosyltransferase
pyrE	Malic enzyme	RBRH_00373	NADP-dependent malic enzyme
pyrE	Malic enzyme	RBRH_03194	NADP-dependent malic enzyme
oxyR	Transcriptional regulator	RBRH_01664	Hydrogen peroxide-inducible genes activator
ubiA	4-hydroxybenzoate polyprenyltransferase and related prenyltransferases	RBRH_01663	4-hydroxybenzoate polyprenyltransferase
proC	Pyrroline-5-carboxylate reductase	RBRH_01662	Pyrroline-5-carboxylate reductase
-	Predicted enzyme with a TIM-barrel fold	RBRH_01661	Hypothetical cytosolic protein
aroF	3-deoxy-D-arabino-heptulosonate 7-phosphate (DAHP) synthase	RBRH_01654	3-deoxy-7-phosphoheptulonate synthase
tldD	Predicted Zn-dependent proteases and their inactivated homologs	RBRH_01653	Microcin-processing peptidase 2 (TldD)
Bxe_A3970	Predicted membrane protein	RBRH_01651	Hypothetical protein
glnE	Glutamine synthetase adenylyltransferase	RBRH_01650	Glutamate-ammonia-ligase adenylyltransferase
recN	ATPase involved in DNA repair	RBRH_01649	DNA repair protein recN
ppnK	Predicted sugar kinase	RBRH_01648	ATP-NAD kinase
hrcA	Transcriptional regulator of heat shock gene	RBRH_01647	Heat-inducible transcription repressor hrcA
hemH	Protoheme ferro-lyase (ferrochelatase)	RBRH_01646	Ferrochelatase
dnaK	Molecular chaperone	RBRH_01643	Chaperone protein dnaK
dnaJ	DnaJ-class molecular chaperone with C-terminal Zn finger domain	RBRH_01642	Chaperone protein dnaJ
BC1001_0454	Anthranilate/para-aminobenzoate synthases component I	RBRH_01641	Para-aminobenzoate synthetase component I
-	Topoisomerase IA	RBRH_02474	Unnamed protein product
-	Plasmid stabilization system protein	RBRH_02475	DNA damage inducible protein yafQ
cinA	Uncharacterized protein (competence- and mitomycin-induced)	RBRH_03198	Putative competence-damage protein
-	Phosphatidylglycerophosphatase A and related proteins	RBRH_03197	Phosphatidylglycerophosphatase A
thiL	Thiamine monophosphate kinase	RBRH_03196	Thiamine-monophosphate kinase

maeB	Malic enzyme	RBRH_00373	NADP-dependent malic enzyme
maeB	Malic enzyme	RBRH_03194	NADP-dependent malic enzyme
-	Hypothetical protein	RBRH_03190	Ribonuclease inhibitor barstar
-	Uncharacterized protein conserved in bacteria	RBRH_03189	surface protein
-	Hypothetical protein	RBRH_01336	AmpD protein
nth	Predicted EndoIII-related endonuclease	RBRH_01335	Endonuclease III
-	3-polyprenyl-4-hydroxybenzoate decarboxylase and related decarboxylases	RBRH_01423	3-polyprenyl-4-hydroxybenzoate decarboxylase
ribH	Riboflavin synthase beta-chain	RBRH_01419	6,7-dimethyl-8-ribityllumazine synthase
ribA	3,4-dihydroxy-2-butanone 4-phosphate synthase	RBRH_01418	GTP cyclohydrolase II/ 3,4-dihydroxy-2-butanone-4-phosphate synthase
ribE	Riboflavin synthase alpha chain	RBRH_01414	Riboflavin synthase alpha chain
ribD	Pyrimidine reductase, riboflavin biosynthesis	RBRH_01413	Diaminohydroxyphosphoribosylaminopyrimidine deaminase/ 5-amino-6-(5-phosphoribosylamino)uracil reductase
hemL	Glutamate-1-semialdehyde aminotransferase	RBRH_01412	Glutamate-1-semialdehyde 2,1-aminomutase
-	ABC-type histidine transport system, ATPase component	RBRH_01406	Histidine transport ATP-binding protein hisP
-	ABC-type arginine transport system, permease component	RBRH_01405	Histidine transport system permease protein hisQ
-	ABC-type amino acid transport/signal transduction systems, periplasmic component/domain	RBRH_02850	Histidine-binding protein
-	ABC-type nitrate/sulfonate/bicarbonate transport system, ATPase component	RBRH_01397	ABC transporter ATP-binding protein
alaS	Alanyl-tRNA synthetase	RBRH_01067	Alanyl-tRNA synthetase
sirA	Predicted redox protein, regulator of disulfide bond formation	RBRH_01061	Hypothetical transcriptional regulatory protein
-	UDP-glucose pyrophosphorylase	RBRH_01060	UTP--glucose-1-phosphate uridylyltransferase
valS	Valyl-tRNA synthetase	RBRH_01058	Valyl-tRNA synthetase
-	Transcriptional regulator	RBRH_03821	Transcriptional regulator, IclR family
pyrD	Dihydroorotate dehydrogenase	RBRH_03884	Dihydroorotate dehydrogenase
-	ADP-ribose pyrophosphatase	RBRH_03881	Mutator mutT protein (7,8-dihydro-8-oxoguanine-triphosphatase)
aat	Leu/Phe-tRNA-protein transferase	RBRH_03882	Leucyl/phenylalanyl-tRNA--protein transferase
-	Single-stranded DNA-binding protein	RBRH_02121	Single-strand DNA binding protein
-	Permeases of the major facilitator superfamily	RBRH_02122	Transporter, MFS superfamily
uvrA	Excinuclease ATPase subunit	RBRH_02123	Excinuclease ABC subunit A
CAGGBEG34_50005	Predicted permease	RBRH_02124	Hypothetical membrane spanning protein
K788_6823	Hypothetical protein	RBRH_03248	Unnamed protein product
secA	Preprotein translocase subunit SecA (ATPase, RNA helicase)	RBRH_03247	Protein translocase subunit secA
argJ	N-acetylglutamate synthase (N-acetylornithine aminotransferase)	RBRH_03246	Glutamate N-acetyltransferase
-	Predicted ATPase (AAA+ superfamily)	RBRH_03245	ATP-dependent protease subunit
mutT	NTP pyrophosphohydrolases including oxidative damage repair enzymes	RBRH_03244	7,8-dihydro-8-oxoguanine-triphosphatase
yacG	Uncharacterized protein conserved in bacteria	RBRH_03243	non-essential pilus assembly protein
-	Uncharacterized protein conserved in bacteria	RBRH_03242	Hypothetical protein
coaE	Dephospho-CoA kinase	RBRH_03241	Dephospho-CoA kinase
-	Type II secretory pathway, prepilin signal peptidase PulO and related peptidases	RBRH_03240	Type 4 prepilin peptidase pilD
pilC	Type II secretory pathway, component PulF	RBRH_03239	Type IV pilus assembly protein tapC
-	Type II secretory pathway, ATPase PulE/Tfp pilus assembly pathway, ATPase PilB	RBRH_03238	Type 4 pili biogenesis protein pilB (nuleotide-binding protein)
-	Putative Mg^{2+} and Co^{2+} transporter CorB	RBRH_03237	Magnesium and cobalt efflux protein corC
ispB	Geranylgeranyl pyrophosphate synthase	RBRH_03235	Farnesyl pyrophosphate synthetase
-	Predicted membrane protein	RBRH_03659	Hypothetical membrane spanning protein
tufA	GTPases - translation elongation factors	RBRH_04212	Protein Translation Elongation Factor Tu (EF-TU)
tufA	GTPases - translation elongation factors	RBRH_01227	Protein Translation Elongation Factor Tu (EF-TU)
secE	Preprotein translocase subunit SecE	RBRH_01696	Protein translocase subunit secE
nusG	Transcription antiterminator	RBRH_01697	Transcription antitermination protein nusG
rplA	Ribosomal protein L1	RBRH_01699	LSU ribosomal protein L1P
rplJ	Ribosomal protein L10	RBRH_01701	LSU ribosomal protein L10P
rplL	Ribosomal protein L7/L12	RBRH_01702	LSU ribosomal protein L12P (L7/L12)
rpoC	DNA-directed RNA polymerase, beta' subunit/160 kD subunit	RBRH_01706	DNA-directed RNA polymerase beta' chain
CAGGBEG34_190142	Hypothetical protein	RBRH_04009	Phage-related protein
recQ	Superfamily II DNA helicase	RBRH_01709	ATP-dependent DNA helicase recQ
rpsG	Ribosomal protein S7	RBRH_01229	SSU ribosomal protein S7P
fusA1	Translation elongation factors (GTPases)	RBRH_00951	Protein Translation Elongation Factor G (EF-G)
fusA1	Translation elongation factors (GTPases)	RBRH_01228	protein=Protein Translation Elongation Factor G (EF-G)
rpsJ	Ribosomal protein S10	RBRH_04209	SSU ribosomal protein S10P
rplC	Ribosomal protein L3	RBRH_02372	LSU ribosomal protein L3P
rplD	Ribosomal protein L4	RBRH_02371	LSU ribosomal protein L1E (= L4P)
rplW	Ribosomal protein L23	RBRH_02370	LSU ribosomal protein L23P
rplB	Ribosomal protein L2	RBRH_02369	LSU ribosomal protein L2P
rpsS	Ribosomal protein S19	RBRH_02368	SSU ribosomal protein S19P
rplV	Ribosomal protein L22	RBRH_02367	LSU ribosomal protein L22P
rpsC	Ribosomal protein S3	RBRH_02366	SSU ribosomal protein S3P
rplP	Ribosomal protein L16/L10E	RBRH_02365	LSU ribosomal protein L16P
rpmC	Ribosomal protein L29	RBRH_02364	LSU ribosomal protein L29P
rpsQ	Ribosomal protein S17	RBRH_02363	SSU ribosomal protein S17P
-	Ribosomal protein L14	RBRH_02361	LSU ribosomal protein L14P
rplX	Ribosomal protein L24	RBRH_02360	LSU ribosomal protein L24P
rplE	Ribosomal protein L5	RBRH_02359	LSU ribosomal protein L5P
rpsN	Ribosomal protein S14	RBRH_02358	SSU ribosomal protein S14P
rpsH	Ribosomal protein S8	RBRH_02357	SSU ribosomal protein S8P
rplR	Ribosomal protein L18	RBRH_02355	LSU ribosomal protein L18P
rpsE	Ribosomal protein S5	RBRH_02354	SSU ribosomal protein S5P
rpmD	Ribosomal protein L30/L7E	RBRH_02353	LSU ribosomal protein L30P
rplO	Ribosomal protein L15	RBRH_02352	LSU ribosomal protein L15P
secY	Preprotein translocase subunit SecY	RBRH_02351	Protein translocase subunit secY
infA	Translation initiation factor 1 (IF-1)	RBRH_02350	Bacterial Protein Translation Initiation Factor 1 (IF-1)
rpsM	Ribosomal protein S13	RBRH_02347	SSU ribosomal protein S13P
rpsK	Ribosomal protein S11	RBRH_02346	SSU ribosomal protein S11P
rpsD	Ribosomal protein S4 and related proteins	RBRH_02344	SSU ribosomal protein S4P
rpoA	DNA-directed RNA polymerase, alpha subunit/40 kD subunit	RBRH_02343	DNA-directed RNA polymerase alpha chain
rplQ	Ribosomal protein L17	RBRH_02342	LSU ribosomal protein L17P
BamMEX5DRAFT_2889	Thiol:disulfide interchange protein	RBRH_02340	Thiol:disulfide interchange protein DsbD
hemB	Delta-aminolevulinic acid dehydratase	RBRH_02338	Delta-aminolevulinic acid dehydratase
CAGGBEG34_190142	Hypothetical protein	RBRH_04009	Phage-related protein
engB	Predicted GTPase	RBRH_02337	GTP-binding protein YihA
-	Cytochrome c553	RBRH_02336	Cytochrome c4
-	ResB protein required for cytochrome c biosynthesis	RBRH_02335	Heme export protein ResB
-	ABC-type transport system involved in cytochrome c biogenesis, permease component	RBRH_02334	Heme export protein ResC / Apocytochrome heme-lyase
lysA	Diaminopimelate decarboxylase	RBRH_02333	Diaminopimelate decarboxylase
cyaY	Protein implicated in iron transport, frataxin homolog	RBRH_02331	Frataxin family protein
-	Membrane carboxypeptidase/penicillin-binding protein	RBRH_02330	Multimodular transpeptidase-transglycosylase PBP 1A
-	Membrane carboxypeptidase/penicillin-binding protein	RBRH_00133	Multimodular transpeptidase-transglycosylase PBP 1A

BYI23_A024070	Tfp pilus assembly protein, ATPase PilM	RBRH_02329	Pili assembly protein PILM
aroK	Shikimate kinase	RBRH_02325	Shikimate kinase
aroB	3-dehydroquinate synthetase	RBRH_02324	3-dehydroquinate synthase
dgt	dGTP triphosphohydrolase	RBRH_02323	Deoxyguanosinetriphosphate triphosphohydrolase
PMI06_006043	Outer membrane protein W	RBRH_02318	Outer membrane protein
-	ABC-type transport system involved in resistance to organic solvents, ATPase component	RBRH_02304	Toluene transport system ATP-binding protein
-	ABC-type transport system involved in resistance to organic solvents, permease component	RBRH_02303	Toluene transport system permease
-	ABC-type transport system involved in resistance to organic solvents, periplasmic component	RBRH_02302	Toluene transport system Ttg2C protein
BURK_006002	ABC-type transport system involved in resistance to organic solvents, auxiliary component	RBRH_02300	Toluene transport system Ttg2D protein
-	Predicted NTP binding protein (contains STAS domain)	RBRH_02299	Toluene transport system Ttg2E protein
-	ABC-type multidrug transport system, ATPase component	RBRH_02298	ABC transporter ATP-binding protein
-	ABC-type multidrug transport system, permease component	RBRH_02297	ABC transporter permease protein
murA	UDP-N-acetylglucosamine enolpyruvyl transferase	RBRH_02295	UDP-N-acetylglucosamine 1-carboxyvinyltransferase
hisG	ATP phosphoribosyltransferase	RBRH_02294	ATP phosphoribosyltransferase
hisD	Histidinol dehydrogenase	RBRH_02293	Histidinol dehydrogenase
hisC	Histidinol-phosphate/aromatic aminotransferase and cobyric acid decarboxylase	RBRH_02292	Histidinol-phosphate aminotransferase
hisB	Imidazoleglycerol-phosphate dehydratase	RBRH_02291	Imidazoleglycerol-phosphate dehydratase
hisH	Glutamine amidotransferase	RBRH_02289	Imidazole glycerol phosphate synthase,glutamine amidotransferase subunit
hisA	Phosphoribosylformimino-5-aminoimidazole carboxamide ribonucleotide (ProFAR) isomerase	RBRH_02288	1-(5-phosphoribosyl)-5-[(5-phosphoribosylamino)methylideneamino] imidazole-4-carboxamide isomerase
hisF	Imidazoleglycerol-phosphate synthase	RBRH_02287	Imidazole glycerol phosphate synthase, cyclase subunit
hisI	Phosphoribosyl-AMP cyclohydrolase	RBRH_02286	Phosphoribosyl-AMP cyclohydrolase
-	Diadenosine tetraphosphate (Ap4A) hydrolase and other HIT family hydrolases	RBRH_02283	Adenosine 5'-monophosphoramidase/Guanosine 5'-monophosphoramidase
tatA	Sec-independent protein secretion pathway components	RBRH_02282	Sec-independent protein translocase protein tatA
tatB	Sec-independent protein secretion pathway components	RBRH_02281	Sec-independent protein translocase protein tatB
tatC	Sec-independent protein secretion pathway component TatC	RBRH_02280	Sec-independent protein translocase protein tatC
-	Trypsin-like serine proteases, typically periplasmic, contain C-terminal PDZ domain	RBRH_02279	Endopeptidase degP
-	Uncharacterized conserved protein	RBRH_02278	NIF3-related protein
-	Rieske Fe-S protein	RBRH_02276	Ubiquinol-cytochrome c reductase iron-sulfur subunit
petB	Cytochrome b subunit of the bc complex	RBRH_02275	Cytochrome b
-	Cytochrome c1	RBRH_02274	Cytochrome c1
sspA	Glutathione S-transferase	RBRH_02273	Stringent starvation protein A
-	Transposase and inactivated derivatives	RBRH_02476	Transposase
istB	DNA replication protein	RBRH_04199	Transposase
istB	DNA replication protein	RBRH_01553	Transposase
-	Transposase and inactivated derivatives	RBRH_03764	Transposase
-	Predicted GTPase, probable translation factor	RBRH_02396	GTP-binding protein, probable translation factor
ubiH	2-polyprenyl-6-methoxyphenol hydroxylase and related FAD-dependent oxidoreductases	RBRH_02393	Putative monooxygenase VisC
-	Predicted protease with the C-terminal PDZ domain	RBRH_02391	M61 glycyl aminopeptidase
SG1089	Hypothetical protein	RBRH_04009	Phage-related protein
-	Indole-3-glycerol phosphate synthase	RBRH_02387	Indole-3-glycerol phosphate synthase
trpD	Anthranilate phosphoribosyltransferase	RBRH_02386	Anthranilate phosphoribosyltransferase
trpG	Anthranilate/para-aminobenzoate synthases component II	RBRH_02385	Anthranilate synthase component II
trpE	Anthranilate/para-aminobenzoate synthases component I	RBRH_02384	Anthranilate synthase component I
rpe	Pentose-5-phosphate-3-epimerase	RBRH_02382	Ribulose-phosphate 3-epimerase
apaG	Uncharacterized protein affecting Mg2+/Co2+ transport	RBRH_02381	ApaG protein
ubiB	Predicted unusual protein kinase	RBRH_02099	2-polyprenylphenol 6-hydroxylase accessory protein ubiB
-	Uncharacterized conserved protein	RBRH_02096	Hypothetical membrane spanning protein
aspS	Aspartyl-tRNA synthetase	RBRH_02095	Aspartyl-tRNA synthetase
ntpA	NTP pyrophosphohydrolases including oxidative damage repair enzymes	RBRH_02094	dATP pyrophosphohydrolase
-	Acyl-CoA dehydrogenases	RBRH_02090	Acyl-CoA dehydrogenase
fadA	Acetyl-CoA acetyltransferase	RBRH_02087	3-ketoacyl-CoA thiolase
-	Acyl-CoA hydrolase	RBRH_02083	Acyl-CoA hydrolase
gdhA	Glutamate dehydrogenase/leucine dehydrogenase	RBRH_02080	Glutamate dehydrogenase
-	ABC-type amino acid transport/signal transduction systems, periplasmic component/domain	RBRH_02079	Glutamate/aspartate-binding protein
gltJ	ABC-type amino acid transport system, permease component	RBRH_02078	Glutamate/aspartate transport system permease protein gltJ
-	ABC-type amino acid transport system, permease component	RBRH_02077	Glutamate/aspartate transport system permease protein gltK
-	ABC-type polar amino acid transport system, ATPase component	RBRH_02076	Glutamate/aspartate transport ATP-binding protein gltL
argD	Ornithine/acetylornithine aminotransferase	RBRH_00911	Acetylornithine aminotransferase
ascD	2-polyprenylphenol hydroxylase and related flavodoxin oxidoreductases	RBRH_00910	Flavodoxin reductase family protein
BCh11DRAFT_05379	Nucleoside-diphosphate-sugar epimerases	RBRH_00906	Nucleoside-diphosphate-sugar epimerases
-	Glutaredoxin-related protein	RBRH_02408	Glutaredoxin
-	3-polyprenyl-4-hydroxybenzoate decarboxylase	RBRH_02409	3-polyprenyl-4-hydroxybenzoate decarboxylase ubiX
CAGGBEG34_220024	Phosphotransferase system IIC components, glucose/maltose/N-acetylglucosamine-specific	RBRH_01185	Holin
-	Transposase and inactivated derivatives	RBRH_02476	Transposase
Neut_1498	Integrase	RBRH_01051	Unnamed protein product
CAGGBEG34_220024	Permeases of the major facilitator superfamily	RBRH_01185	Holin
-	Hypothetical protein	RBRH_01189	Lysozyme
-	Hypothetical protein	RBRH_01186	Lysozyme
L682_09955	Uncharacterized homolog of phage Mu protein gp47	RBRH_01193	Unnamed protein product
L682_09945	Hypothetical protein	RBRH_01195	phage-related protein
HIB_15810	Hypothetical protein	RBRH_01196	Unnamed protein product
-	Predicted transcriptional regulator	RBRH_01522	Hypothetical cytosolic protein
BC1002_6556	Hypothetical protein	RBRH_01199	Unnamed protein product
L682_09905	Hypothetical protein	RBRH_01202	Unnamed protein product
XFEB_01759	Uncharacterized protein, homolog of phage Mu protein gp30	RBRH_01211	Unnamed protein product
-	Uncharacterized protein conserved in bacteria	RBRH_01213	large terminase subunit
CAGGBEG34_610004	Methyl-accepting chemotaxis protein	RBRH_01048	Unnamed protein product
-	Integrase	RBRH_01051	Unnamed protein product
-	Ferredoxin	RBRH_02590	Ferredoxin
-	Na+/H+ antiporter NhaD and related arsenite permeases	RBRH_02593	Hypothetical membrane spanning protein
-	Uncharacterized protein conserved in bacteria	RBRH_02595	RmuC family protein
-	Hypothetical protein	RBRH_02596	Unnamed protein product
-	Methylase of polypeptide chain release factors	RBRH_02405	Peptide release factor-glutamine N5-methyltransferase
prfA	Protein chain release factor A	RBRH_02404	Bacterial Peptide Chain Release Factor 1
-	Predicted ATPase related to phosphate starvation-inducible protein PhoH	RBRH_02596	PhoH protein

bcp	Peroxiredoxin	RBRH_03685	Thioredoxin peroxidase
-	Predicted xylanase/chitin deacetylase	RBRH_03686	Chitooligosaccharide deacetylase
-	Nucleoside-diphosphate-sugar epimerases	RBRH_03682	UDP-glucuronate 4-dehydrogenase (decarboxylating)
pmrIa	Methionyl-tRNA formyltransferase	RBRH_03684	UDP-4-amino-4-deoxy-L-arabinose N-formyltransferase
pmrF	Glycosyltransferases involved in cell wall biogenesis	RBRH_03680	Undecaprenyl-phosphate alpha-4-amino-L-arabinosyltransferase
pmrH	Predicted pyridoxal phosphate-dependent enzyme apparently involved in regulation of cell wall biogenesis	RBRH_03679	UDP-4-amino-L-arabinose synthase
-	Permeases of the drug/metabolite transporter (DMT) superfamily	RBRH_03678	Quaternary ammonium compound-resistance protein
arnT	4-amino-4-deoxy-L-arabinose transferase and related glycosyltransferases of PMT family	RBRH_03677	Glucosyltransferase
-	Aspartate/tyrosine/aromatic aminotransferase	RBRH_03675	Probable aminotransferase yfbQ
pyrF	Orotidine-5'-phosphate decarboxylase	RBRH_03199	Orotidine 5'-phosphate decarboxylase
C265_18829	Transcriptional regulator	RBRH_02657	Transcriptional regulators, LysR family
BN113_0351	Transcriptional regulator	RBRH_02721	Transcriptional regulators, LysR family
-	ATPase components of ABC transporters with duplicated ATPase domains	RBRH_01375	ABC transporter ATP-binding protein uup
parE	Type IIA topoisomerase (DNA gyrase/topo II, topoisomerase IV), B subunit	RBRH_01374	Topoisomerase IV subunit B
parC	Type IIA topoisomerase (DNA gyrase/topo II, topoisomerase IV), A subunit	RBRH_01373	Topoisomerase IV subunit A
aspC	Aspartate/tyrosine/aromatic aminotransferase	RBRH_02874	Aromatic-amino-acid aminotransferase
-	Helicase subunit of the DNA excision repair complex	RBRH_02875	Excinuclease ABC subunit B
RBRH_00986	Permeases of the drug/metabolite transporter (DMT) superfamily	RBRH_00986	Transporter, drug/metabolite exporter family
greB	Transcription elongation factor	RBRH_01007	Transcription elongation factor greB
spoT	Guanosine polyphosphate pyrophosphohydrolases/synthetases	RBRH_01008	GTP pyrophosphokinase
rpoZ	DNA-directed RNA polymerase, subunit K/omega	RBRH_01009	DNA-directed RNA polymerase omega chain
gmk	Guanylate kinase	RBRH_01010	Guanylate kinase
rph	RNase PH	RBRH_01013	Ribonuclease PH
-	Xanthosine triphosphate pyrophosphatase	RBRH_01014	Xanthosine triphosphate pyrophosphatase
hemN	Coproporphyrinogen III oxidase and related Fe-S oxidoreductases	RBRH_01015	Oxygen-independent coproporphyrinogen-III oxidase
talA	Transaldolase	RBRH_01343	Transaldolase
-	Protein involved in catabolism of external DNA	RBRH_02873	Protein involved in external DNA uptake
surE	Predicted acid phosphatase	RBRH_03744	Acid phosphatase surE
-	Protein-L-isoaspartate carboxylmethyltransferase	RBRH_03743	Protein-L-isoaspartate O-methyltransferase
-	Membrane proteins related to metalloendopeptidases	RBRH_03742	Peptidoglycan-specific endopeptidase, M23 family
-	Transposase and inactivated derivatives, IS5 family	RBRH_03381	Transposase
-	Predicted 3'-5' exonuclease related to the exonuclease domain of PolB	RBRH_03740	Hypothetical cytosolic protein
groES	Co-chaperonin GroES (HSP10)	RBRH_01487	0 kDa chaperonin GROES
groEL	Chaperonin GroEL (HSP60 family)	RBRH_01486	60 kDa chaperonin GROEL
-	Rubredoxin	RBRH_01484	Rubredoxin
-	Putative transcriptional regulator	RBRH_01481	Transcriptional regulator, algH
yqqF	Predicted endonuclease involved in recombination (possible Holliday junction resolvase in Mycoplasmas and B. subtilis)	RBRH_01480	Hypothetical cytosolic protein
-	Pyrimidine operon attenuation protein/uracil phosphoribosyltransferase	RBRH_01479	Uracil phosphoribosyltransferase
pyrB	Aspartate carbamoyltransferase, catalytic chain	RBRH_01477	Aspartate carbamoyltransferase
pyrC	Dihydroorotase and related cyclic amidohydrolases	RBRH_01476	Aspartate carbamoyltransferase non-catalytic chain
K788_7818	1-acyl-sn-glycerol-3-phosphate acyltransferase	RBRH_01475	1-acyl-sn-glycerol-3-phosphate acyltransferase
CAGGBEG34_200100	Uncharacterized protein conserved in bacteria	RBRH_01545	Hypothetical exported protein
-	Uncharacterized protein SCO1/SenC/PrrC, involved in biogenesis of respiratory and photosynthetic systems	RBRH_02431	SCO2 protein precursor
-	ABC-type multidrug transport system, ATPase and permease components	RBRH_02428	Multidrug resistance ABC transporter ATP-binding and permease protein
-	Glycosyltransferase	RBRH_00363	Glycosyltransferase
-	Glycosyltransferase	RBRH_02426	Glycosyltransferase
-	Hypothetical protein	RBRH_02876	34 kDa membrane antigen precursor
-	High-affinity Fe2+/Pb2+ permease	RBRH_02878	High-affinity iron permease
-	Polyferredoxin	RBRH_02879	Nitrogen fixation protein vnfA
-	Membrane-fusion protein	RBRH_01297	Acriflavin resistance periplasmic protein
-	Cation/multidrug efflux pump	RBRH_01296	Acriflavin resistance plasma membrane protein
-	Outer membrane protein	RBRH_01295	Type I secretion outer membrane protein
-	ABC-type Mn2+/Zn2+ transport systems, permease components	RBRH_01292	Manganese transport system membrane protein
-	ABC-type Mn/Zn transport systems, ATPase component	RBRH_01291	Manganese transport system ATP-binding protein
-	ABC-type metal ion transport system, periplasmic component/surface adhesin	RBRH_01290	Manganese-binding protein
CAGGBEG34_200017	Tfp pilus assembly protein PilW	RBRH_01538	Unnamed protein product
Bcep1808_0746	Predicted membrane protein	RBRH_01542	Hypothetical protein
-	Uncharacterized conserved protein	RBRH_01543	ElaB protein
msrA	Peptide methionine sulfoxide reductase	RBRH_01085	Peptide methionine sulfoxide reductase msrA
pdxH	Pyridoxamine-phosphate oxidase	RBRH_01081	Pyridoxamine 5'-phosphate oxidase
glnS	Glutamyl- and glutaminyl-tRNA synthetases	RBRH_01072	Glutaminyl-tRNA synthetase
-	Predicted sugar phosphate isomerase involved in capsule formation	RBRH_02131	Arabinose-5-phosphate isomerase
kdsC	Low specificity phosphatase (HAD superfamily)	RBRH_02132	3-deoxy-manno-octulosonate-8-phosphatase
MYA_2549	Uncharacterized protein conserved in bacteria	RBRH_02133	Unnamed protein product
Bphyt_0598	Uncharacterized protein conserved in bacteria	RBRH_02134	Protein yhbN precursor
-	ABC-type (unclassified) transport system, ATPase component	RBRH_02135	ABC transporter ATP-binding protein
ptsN	Phosphotransferase system mannitol/fructose-specific IIA domain (Ntr-type)	RBRH_02140	Nitrogen regulatory IIA protein
hprK	Serine kinase of the HPr protein, regulates carbohydrate metabolism	RBRH_02141	HPR(Ser) Kinase/Phosphatase
-	Predicted P-loop-containing kinase	RBRH_02142	ATP-binding protein (contains P-loop)
RBRH_00029	Hypothetical protein	RBRH_00029	Unnamed protein product
-	Cytochrome c553	RBRH_01338	Cytochrome c-554
-	Cytochrome c553	RBRH_01337	Cytochrome c-554(548)
hemA	Glutamyl-tRNA reductase	RBRH_02403	Glutamyl-tRNA reductase
	Xaa-Pro aminopeptidase	RBRH_02401	Xaa-Pro aminopeptidase
CAGGBEG34_240007	Response regulator containing a CheY-like receiver domain and an HTH DNA-binding domain	RBRH_01136	Transcriptional regulator, LuxR family
-	Hypothetical protein	RBRH_01137	Hypothetical protein
-	Ribonucleases G and E	RBRH_02601	Ribonuclease E
rluC	Pseudouridylate synthases, 23S RNA-specific	RBRH_02605	Ribosomal large subunit pseudouridine synthase C
-	Periplasmic serine proteases (ClpP class)	RBRH_02608	Signal peptide peptidase sppA
-	Predicted methyltransferases	RBRH_02609	Tetrapyrrole (Corrin/Porphyrin) methylase family protein
rpmF	Ribosomal protein L32	RBRH_02613	LSU ribosomal protein L32P
plsX	Fatty acid/phospholipid biosynthesis enzyme	RBRH_02614	Fatty acid/phospholipid synthesis protein plsX
fabH1	3-oxoacyl-[acyl-carrier-protein] synthase III	RBRH_02615	3-oxoacyl-[acyl-carrier-protein] synthase III
fabD	(acyl-carrier-protein) S-malonyltransferase	RBRH_02616	Malonyl-CoA-[acyl-carrier-protein] transacylase
fabG	Dehydrogenases with different specificities (related to short-chain alcohol dehydrogenases)	RBRH_02617	3-oxoacyl-[acyl-carrier protein] reductase
fabF1	3-oxoacyl-(acyl-carrier-protein) synthase	RBRH_02619	3-oxoacyl-[acyl-carrier-protein] synthase

rpoE1	DNA-directed RNA polymerase specialized sigma subunit, sigma24 homolog	RBRH_02621	RNA polymerase sigma-E factor
-	Trypsin-like serine proteases, typically periplasmic, contain C-terminal PDZ domain	RBRH_02624	Endopeptidase degP
-	Transposase and inactivated derivatives	RBRH_02476	Transposase
lepA	Membrane GTPase LepA	RBRH_02448	GTP-binding protein lepA
spaS	Signal peptidase I	RBRH_02447	Signal peptidase I
era	GTPase	RBRH_02445	GTP-binding protein era
recO	Recombinational DNA repair protein (RecF pathway)	RBRH_02444	DNA repair protein recO
pdxJ	Pyridoxal phosphate biosynthesis protein	RBRH_02443	Pyridoxal phosphate biosynthetic protein pdxJ
nagZ	Beta-glucosidase-related glycosidases	RBRH_02441	Anhydromuramoyl-peptide exo-beta-N-acetylglucosaminidase
efp	Translation elongation factor P (EF-P)/translation initiation factor 5A (eIF-5A)	RBRH_02438	Protein Translation Elongation Factor P
Bxe_A1094	Uncharacterized protein conserved in bacteria	RBRH_02437	Hypothetical cytosolic protein
uvrC	Glutamate synthase domain 2	RBRH_02436	Excinuclease ABC subunit C
pgsA	Nuclease subunit of the excinuclease complex	RBRH_02435	CDP-diacylglycerol--glycerol-3-phosphate 3-phosphatidyltransferase
-	Phosphatidylglycerophosphate synthase	RBRH_03699	ABC transporter ATP-binding protein
rpsF	Ribosomal protein S6	RBRH_03695	SSU ribosomal protein S6P
-	Primosomal replication protein N	RBRH_03694	Primosomal replication protein N
rpsR	Ribosomal protein S18	RBRH_03693	SSU ribosomal protein S18P
rplI	Ribosomal protein L9	RBRH_03692	LSU ribosomal protein L9P
dnaB	Replicative DNA helicase	RBRH_03691	Replicative DNA helicase
-	Phosphate transport regulator (distant homolog of PhoU)	RBRH_03690	Putative pit accessory protein
-	Phosphate/sulphate permeases	RBRH_03689	Low-affinity inorganic phosphate transporter
minE	Septum formation topological specificity factor	RBRH_01023	Cell division inhibitor MinE
minD	Septum formation inhibitor-activating ATPase	RBRH_01024	Cell division inhibitor MinD
minC	Septum formation inhibitor	RBRH_01025	Cell division inhibitor MinC
-	Biopolymer transport protein	RBRH_03935	TolR protein
-	Periplasmic protein TonB, links inner and outer membranes	RBRH_03934	TolQ protein
BURK_023930	Glutamate racemase	RBRH_03932	Bacterioferritin-associated ferredoxin
-	Bacterioferritin (cytochrome b1)	RBRH_03930	Bacterioferritin
-	Predicted membrane protein	RBRH_03928	Hypothetical membrane spanning protein
fbp1	Fructose-1,6-bisphosphatase	RBRH_00086	Fructose-1,6-bisphosphatase
-	Putative translation initiation inhibitor, yjgF family	RBRH_01369	Translation initiation inhibitor
htpG	Molecular chaperone, HSP90 family	RBRH_01367	Chaperone protein htpG
ubiC	4-hydroxybenzoate synthetase (chorismate lyase)	RBRH_01366	Chorismate--pyruvate lyase
-	G:T/U mismatch-specific DNA glycosylase	RBRH_01365	Hypothetical cytosolic protein
-	Spermidine synthase	RBRH_01364	Spermidine synthase
mutY	A/G-specific DNA glycosylase	RBRH_02144	A/G-specific adenine DNA glycosylase
mutM	Formamidopyrimidine-DNA glycosylase	RBRH_02145	Formamidopyrimidine-DNA glycosylase
-	FOG: TPR repeat	RBRH_02146	Tetratricopeptide repeat family protein
ipk	4-diphosphocytidyl-2C-methyl-D-erythritol 2-phosphate synthase	RBRH_02148	4-diphosphocytidyl-2-C-methyl-D-erythritol kinase
prsA	Phosphoribosylpyrophosphate synthetase	RBRH_02149	Ribose-phosphate pyrophosphokinase
rplY	Ribosomal protein L25 (general stress protein Ctc)	RBRH_02151	LSU ribosomal protein L25P
pth1	Peptidyl-tRNA hydrolase	RBRH_02152	Peptidyl-tRNA hydrolase
RBRH_01242	Outer membrane protein (porin)	RBRH_01242	Outer membrane porin protein 32 precursor
-	Integral membrane protein, interacts with FtsH	RBRH_03737	SecY stabilizing membrane protein
ndk	Nucleoside diphosphate kinase	RBRH_03736	Nucleoside diphosphate kinase
-	Predicted Fe-S-cluster redox enzyme	RBRH_03735	Radical SAM family enzyme
BH160DRAFT_6135	Uncharacterized protein conserved in bacteria	RBRH_03733	Integral membrane protein
ispG	Enzyme involved in the deoxyxylulose pathway of isoprenoid biosynthesis	RBRH_03732	1-hydroxy-2-methyl-2-(E)-butenyl 4-diphosphate synthase
hisS	Histidyl-tRNA synthetase	RBRH_03731	Histidyl-tRNA synthetase
BH160DRAFT_6132	Uncharacterized protein conserved in bacteria	RBRH_03730	Hypothetical protein
-	FOG: WD40-like repeat	RBRH_03729	PQQ enzyme repeat family protein
engA	Predicted GTPases	RBRH_03728	GTP-binding protein
hfq	Uncharacterized host factor I protein	RBRH_03727	sRNA chaperone Hfq
BC1003_1211	Membrane protease subunits, stomatin/prohibitin homologs	RBRH_03725	Protease activity modulator HflK
CARN2_3473	Membrane protease subunits, stomatin/prohibitin homologs	RBRH_03724	Protease activity modulator HflC
-	Membrane protease subunits, stomatin/prohibitin homologs	RBRH_03722	Histidyl-tRNA synthetase
purA	ATP phosphoribosyltransferase involved in histidine biosynthesis	RBRH_03721	Adenylosuccinate synthetase
kup2	Adenylosuccinate synthase	RBRH_03719	Kup system potassium uptake protein
-	K+ transporter	RBRH_03718	Transcription accessory protein (S1 RNA binding domain)
dinG	Hypothetical protein	RBRH_03715	ATP-dependent helicase, DinG family
queF	Enzyme related to GTP cyclohydrolase I	RBRH_02109	Queuosine biosynthesis protein QueF
ilvA	Threonine dehydratase	RBRH_02108	Threonine dehydratase
-	FAD/FMN-containing dehydrogenases	RBRH_02105	Anaerobic glycerol-3-phosphate dehydrogenase membrane anchor subunit
-	Diadenosine tetraphosphate (Ap4A) hydrolase and other HIT family hydrolases	RBRH_02104	HIT family hydrolase
ubiE	Methylase involved in ubiquinone/menaquinone biosynthesis	RBRH_02102	Ubiquinone/menaquinone biosynthesis methyltransferase ubiE
BC1002_0370	Uncharacterized protein conserved in bacteria	RBRH_02101	Hypothetical protein
ligA	Response regulator containing a CheY-like receiver domain and an HTH DNA-binding domain	RBRH_03584	NAD-dependent DNA ligase
glnD	NAD-dependent DNA ligase (contains BRCT domain type II)	RBRH_03580	protein-PII- uridylyltransferase
map	UTP:GlnB (protein PII) uridylyltransferase	RBRH_03579	Methionine aminopeptidase
-	Dephospho-CoA kinase	RBRH_00071	CrcB family protein
-	Integral membrane protein possibly involved in chromosome condensation	RBRH_00077	Glutamyl-tRNA(Gln) amidotransferase subunit A
-	Asp-tRNAAsn/Glu-tRNAGln amidotransferase A subunit and related amidases	RBRH_00011	Transcriptional activator protein metR
metE	Transcriptional regulator	RBRH_00010	5-methyltetrahydropteroyltriglutamate--homocysteine methyltransferase
-	Uncharacterized protein related to capsule biosynthesis enzymes	RBRH_00085	Membrane alanine aminopeptidase
-	Hypothetical protein	RBRH_01726	Membrane-bound lytic murein transglycosylase B
-	Predicted NTPase (NACHT family)	RBRH_00082	Integral membrane protein
metN	Predicted membrane protein	RBRH_01728	ABC transporter ATP-binding protein
-	ABC-type metal ion transport system, ATPase component	RBRH_01729	ABC transporter permease protein
-	ABC-type metal ion transport system, permease component	RBRH_01730	ABC transporter substrate-binding protein
-	ABC-type metal ion transport system, periplasmic component	RBRH_02176	ABC transporter substrate-binding protein
-	ABC-type metal ion transport system, periplasmic component/surface antigen	RBRH_01731	Electron transfer flavoprotein beta-subunit
-	Electron transfer flavoprotein, beta subunit	RBRH_01732	Electron transfer flavoprotein alpha-subunit
-	Predicted NTPase (NACHT family)	RBRH_01736	Radical SAM superfamily protein
rpsP	DNA repair photolyase	RBRH_01739	SSU ribosomal protein S16P
rimM	Ribosomal protein S16	RBRH_01740	16S rRNA processing protein rimM
trmD	RimM protein, required for 16S rRNA processing	RBRH_01741	tRNA (Guanine-N(1)-)-methyltransferase
cobD	Ribosomal protein L19	RBRH_01744	Adenosylcobinamide-phosphate synthase
engC	FOG: TPR repeat	RBRH_01746	GTPase

-	Predicted GTPases	RBRH_01747	Zinc metalloprotease
orn	Zn-dependent protease with chaperone function	RBRH_01749	Oligoribonuclease
pmbA	Oligoribonuclease (3'->5' exoribonuclease)	RBRH_01753	Microcin-processing peptidase 1 (PmbA)
folA	Transposase and inactivated derivatives	RBRH_01754	Dihydrofolate reductase
thyA	Dihydrofolate reductase	RBRH_01755	Thymidylate synthase
lexA	tRNA-dihydrouridine synthase	RBRH_00149	LexA repressor
-	SOS-response transcriptional repressors (RecA-mediated autopeptidases)	RBRH_03698	L-asparaginase
rpsB	L-asparaginase/archaeal Glu-tRNAGln amidotransferase subunit D	RBRH_03576	SSU ribosomal protein S2P
tsf	Ribosomal protein S2	RBRH_03574	Protein Translation Elongation Factor Ts (EF-Ts)
pyrH	Translation elongation factor Ts	RBRH_03573	Uridylate kinase
frr	Uridylate kinase	RBRH_03572	Ribosome Recycling Factor (RRF)
uppS	Ribosome recycling factor	RBRH_03571	Undecaprenyl pyrophosphate synthetase
RBRH_03570	Undecaprenyl pyrophosphate synthase	RBRH_03570	Phosphatidate cytidylyltransferase
dxr	CDP-diglyceride synthetase	RBRH_03569	1-deoxy-D-xylulose 5-phosphate reductoisomerase
-	1-deoxy-D-xylulose 5-phosphate reductoisomerase	RBRH_03567	Outer membrane protein assembly factor yaeT
-	Outer membrane protein/protective antigen OMA87	RBRH_03566	Outer membrane protein OmpH
lpxD	Outer membrane protein	RBRH_03565	UDP-3-O-[3-hydroxymyristoyl
fabZ	UDP-3-O-[3-hydroxymyristoyl] glucosamine N-acyltransferase	RBRH_00049	(3R)-hydroxymyristoyl-[acyl carrier protein] dehydratase
lpxA	3-hydroxymyristoyl/3-hydroxydecanoyl-(acyl carrier protein) dehydratases	RBRH_00048	Acyl-[acyl-carrier-protein]--UDP-N-acetylglucosamine O-acyltransferase
lpxB	Acyl-[acyl carrier protein]--UDP-N-acetylglucosamine O-acyltransferase	RBRH_00047	Lipid-A-disaccharide synthase
rnhB	Lipid A disaccharide synthetase	RBRH_00046	Ribonuclease HII
BTH_I2042	Ribonuclease HII	RBRH_00045	23S rRNA methyltransferase
-	Uncharacterized conserved protein	RBRH_00044	ATP/GTP-binding protein
ppsA	Uncharacterized protein conserved in bacteria	RBRH_00043	Phosphoenolpyruvate synthase
BDAG_01288	Phosphoenolpyruvate synthase/pyruvate phosphate dikinase	RBRH_00042	Hypothetical protein
smpB	Histone acetyltransferase HPA2 and related acetyltransferases	RBRH_02709	SsrA-binding protein
WQE_31521	Oligoketide cyclase/lipid transport protein	RBRH_02708	Hypothetical cytosolic protein
guaB	Uncharacterized protein conserved in bacteria	RBRH_02707	Inosine-5'-monophosphate dehydrogenase
guaA	IMP dehydrogenase/GMP reductase	RBRH_02704	GMP synthase [glutamine-hydrolyzing
tnpA	Predicted NTPase (NACHT family)	RBRH_02021	Transposase
-	Transposase and inactivated derivatives	RBRH_02020	Transposase
rep	Predicted NTPase (NACHT family)	RBRH_00848	ATP-dependent DNA helicase rep
A33K_12849	Superfamily I DNA and RNA helicases	RBRH_00849	Cytochrome c555
-	Hypothetical protein	RBRH_02803	Potassium-transporting ATPase A chain
-	Osmosensitive K+ channel histidine kinase	RBRH_02495	Two-component response regulator kdpE
mfd	Hypothetical protein	RBRH_03399	Transcription-repair coupling factor
ispD	Transcription-repair coupling factor (superfamily II helicase)	RBRH_03400	2-C-methyl-D-erythritol 4-phosphate cytidylyltransferase
ispF	4-diphosphocytidyl-2-methyl-D-erithritol synthase	RBRH_03401	2-C-methyl-D-erythritol 2,4-cyclodiphosphate synthase
-	Superfamily II RNA helicase	RBRH_03625	GTP-binding protein TypA/BipA
rhlE2	Predicted membrane GTPase involved in stress response	RBRH_02465	ATP-dependent RNA helicase
-	Superfamily II DNA and RNA helicases	RBRH_02467	Rhodanese-related sulfurtransferases
RPSI07_mp1112	3-methyladenine DNA glycosylase	RBRH_00100	Hemagglutinin-like protein
-	Glucose-6-phosphate isomerase	RBRH_02020	Transposase
-	Transposase and inactivated derivatives	RBRH_02021	Transposase
dnaE	Transposase and inactivated derivatives	RBRH_02469	DNA polymerase III alpha subunit
msbA	Lipid A core - O-antigen ligase and related enzymes	RBRH_02470	Phospholipid-lipopolysaccharide ABC transporter
cafA	ABC-type multidrug transport system, ATPase and permease components	RBRH_02481	Ribonuclease G
-	Ribonucleases G and E	RBRH_02484	iojap protein family
nadD	Uncharacterized homolog of plant Iojap protein	RBRH_02485	Nicotinate-nucleotide adenylyltransferase
hemF	Nicotinic acid mononucleotide adenylyltransferase	RBRH_02486	Coproporphyrinogen III oxidase
purD	Coproporphyrinogen III oxidase	RBRH_02487	Phosphoribosylamine--glycine ligase
-	Phosphoribosylamine-glycine ligase	RBRH_02488	Hypothetical cytosolic protein
-	Uncharacterized conserved protein	RBRH_03644	Ribosomal large subunit pseudouridine synthase B
BURK_000910	Predicted transcriptional regulator containing the HTH domain	RBRH_01550	Peptidoglycan-specific endopeptidase, M23 family
-	Hypothetical protein	RBRH_00852	Transcriptional regulator
SG0735	Uracil-DNA glycosylase	RBRH_03599	Hypothetical cytosolic protein
-	DNA polymerase III, gamma/tau subunits	RBRH_03748	Hypothetical transcriptional regulatory protein
recR	Uncharacterized protein conserved in bacteria	RBRH_03747	Recombination protein recR
iscX	Recombinational DNA repair protein (RecF pathway)	RBRH_03832	Hypothetical cytosolic protein
-	Hypothetical protein	RBRH_01057	DNA helicase II
Bcep18194_A3475	Superfamily I DNA and RNA helicases	RBRH_02341	Periplasmic divalent cation tolerance protein CutA
thrB	Hypothetical protein	RBRH_01055	Homoserine kinase
-	Putative homoserine kinase type II (protein kinase fold)	RBRH_01052	AMP nucleosidase
-	Nucleoside phosphorylase	RBRH_01170	3-isopropylmalate dehydrogenase
polA	Predicted Rossmann fold nucleotide-binding protein	RBRH_01169	DNA polymerase I
-	DNA-directed RNA polymerase, beta subunit/140 kD subunit	RBRH_01168	NADH dehydrogenase
RSp1073	Thymidylate kinase	RBRH_00100	Hemagglutinin-like protein
-	Rhodanese-related sulfurtransferase	RBRH_01165	ALCE
-	ABC-type spermidine/putrescine transport systems, ATPase components	RBRH_03381	Transposase
thiE	Transaldolase	RBRH_02305	Thiamin-phosphate pyrophosphorylase
thiG	Thiamine monophosphate synthase	RBRH_02306	Thiazole biosynthesis protein thiG
Bphyt_3574	Uncharacterized enzyme of thiazole biosynthesis	RBRH_02307	ThiS protein
-	Sulfur transfer protein involved in thiamine biosynthesis	RBRH_02308	Glycine oxidase
ispA	Exonuclease VII small subunit	RBRH_01162	Dimethylallyltransferase/Geranyltranstransferase
dxs	Geranylgeranyl pyrophosphate synthase	RBRH_01161	1-deoxy-D-xylulose 5-phosphate synthase
-	Deoxyxylulose-5-phosphate synthase	RBRH_01160	Hypothetical cytosolic protein
gcp	Uncharacterized conserved protein	RBRH_01159	O-sialoglycoprotein endopeptidase
rpsU	Uncharacterized conserved protein	RBRH_01156	SSU ribosomal protein S21P
-	Ribosomal protein S21	RBRH_01155	GatB/Yqey domain protein
dnaG	Uncharacterized conserved protein	RBRH_01154	DNA primase
rpoD	DNA primase (bacterial type)	RBRH_01153	RNA polymerase sigma factor rpoD
-	Predicted aminomethyltransferase related to GcvT	RBRH_01584	Hypothetical exported protein
tmk	Predicted periplasmic solute-binding protein	RBRH_01583	Thymidylate kinase
holB	Thymidylate kinase	RBRH_01582	DNA polymerase III, delta' subunit
-	Sortase and related acyltransferases	RBRH_01580	DNase, TatD family
GWL_35760	Hypothetical protein	RBRH_02184	Hypothetical protein
istB	Membrane protein involved in the export of O-antigen and teichoic acid	RBRH_03978	Transposase
-	Autotransporter adhesin	RBRH_03585	Cell division protein zipA
-	Hypothetical protein	RBRH_03586	Chromosome partition protein smc
dapC	Permeases of the drug/metabolite transporter (DMT) superfamily	RBRH_03588	N-Succinyl-L,L-DAP Aminotransferase
dapD	Aspartate/tyrosine/aromatic aminotransferase	RBRH_03589	2,3,4,5-tetrahydropyridine-2-carboxylate N-succinyltransferase
-	Tetrahydrodipicolinate N-succinyltransferase	RBRH_03590	Arsenate reductase
dapE	Arsenate reductase and related proteins, glutaredoxin family	RBRH_03591	Succinyl-diaminopimelate desuccinylase
cls	Methylase of polypeptide chain release factors	RBRH_03593	Cardiolipin synthetase
-	Phosphatidylserine/phosphatidylglycerophosphate/cardiolipin synthases and related enzymes	RBRH_03597	ABC transporter ATP-binding protein

RBRH_02472	Uncharacterized protein conserved in bacteria	RBRH_02472	Unnamed protein product
RBRH_02471	ATPases involved in chromosome partitioning	RBRH_02471	Unnamed protein product
-	Transposase and inactivated derivatives	RBRH_01599	Hypothetical cytosolic protein
lysC	Integrase	RBRH_03956	Aspartokinase
CAGGBEG34_30027	Thioesterase domains of type I polyketide synthases or non-ribosomal peptide synthetases	RBRH_02120	Reverse transcriptase
-	Retron-type reverse transcriptase	RBRH_02476	Transposase
tilS	Transposase and inactivated derivatives	RBRH_03957	tRNA(Ile)-lysidine synthetase TilS
accA	Predicted ATPase of the PP-loop superfamily implicated in cell cycle control	RBRH_03958	Acetyl-coenzyme A carboxylase carboxyl transferase subunit alpha
cysS	3-methyladenine DNA glycosylase/8-oxoguanine DNA glycosylase	RBRH_03960	Cysteinyl-tRNA synthetase
ppiB	FOG: TPR repeat	RBRH_03963	Peptidyl-prolyl cis-trans isomerase
-	Peptidyl-prolyl cis-trans isomerase (rotamase) - cyclophilin family	RBRH_03964	UDP-2,3-diacylglucosamine hydrolase
-	L-alanine-DL-glutamate epimerase and related enzymes of enolase superfamily	RBRH_00092	Transposase
-	Predicted NTPase (NACHT family)	RBRH_03966	RRNA methylase, SpoU family
suhB	rRNA methylase	RBRH_03967	Myo-inositol-1(or 4)-monophosphatase
-	Phosphoglycerol transferase and related proteins, alkaline phosphatase superfamily	RBRH_03620	ATPase
-	Predicted ATPase	RBRH_03621	Dihydrolipoamide dehydrogenase
sucB	Pyruvate/2-oxoglutarate dehydrogenase complex, dihydrolipoamide dehydrogenase (E3) component, and related enzymes	RBRH_03623	Dihydrolipoamide succinyltransferase component (E2) of 2-oxoglutarate dehydrogenase complex
sucA	Pyruvate/2-oxoglutarate dehydrogenase complex, dihydrolipoamide acyltransferase (E2) component, and related enzymes	RBRH_03624	2-oxoglutarate dehydrogenase E1 component
fdx-1	2-oxoglutarate dehydrogenase complex, dehydrogenase (E1) component, and related enzymes	RBRH_03831	Ferredoxin, 2Fe-2s
hscA	Ferredoxin	RBRH_03830	Chaperone protein hscA
hscB	Molecular chaperone	RBRH_03829	Chaperone protein hscB
-	DnaJ-domain-containing proteins 1	RBRH_03828	Iron-sulphur cluster assembly protein IscA
-	Uncharacterized conserved protein	RBRH_03827	IscU protein
iscS	NifU homolog involved in Fe-S cluster formation	RBRH_03826	Cysteine desulfurase
-	Cysteine sulfinate desulfinase/cysteine desulfurase and related enzymes	RBRH_03825	Rrf2 family protein
bglu_1g19220	Predicted transcriptional regulator	RBRH_00954	Multidrug resistance protein B
bglu_1g19220	Predicted transcriptional regulator	RBRH_03409	Multidrug resistance protein B
BgramDRAFT_5853	Permeases of the major facilitator superfamily	RBRH_02952	Ribonuclease BN
-	Uncharacterized protein related to capsule biosynthesis enzymes	RBRH_00984	Putative periplasmic protein
-	Uncharacterized protein conserved in bacteria	RBRH_00983	Putative periplasmic protein
-	Uncharacterized protein conserved in bacteria	RBRH_00982	Hypothetical exported protein
leuA	Predicted NTPase (NACHT family)	RBRH_02514	2-isopropylmalate synthase
pssA	Isopropylmalate/homocitrate/citramalate synthases	RBRH_02513	CDP-diacylglycerol--serine O-phosphatidyltransferase
psd	Phosphatidylserine synthase	RBRH_02512	Phosphatidylserine decarboxylase
ilvC	Phosphatidylserine decarboxylase	RBRH_02511	Ketol-acid reductoisomerase
ilvH	Ketol-acid reductoisomerase	RBRH_02510	Acetolactate synthase small subunit
ilvB	Acetolactate synthase, small (regulatory) subunit	RBRH_02508	Acetolactate synthase large subunit
-	Thiamine pyrophosphate-requiring enzymes [acetolactate synthase, pyruvate dehydrogenase (cytochrome), glyoxylate carboligase, phosphonopyruvate decarboxylase]	RBRH_02506	Specialized sigma subunits of RNA polymerase
Bphy_2024	DNA-directed RNA polymerase specialized sigma subunit, sigma24 homolog	RBRH_02505	Unnamed protein product
-	Hypothetical protein	RBRH_02500	Transcriptional regulator, TetR family
BUPH_00305	Transcriptional regulator	RBRH_01384	Hypothetical protein
-	Uncharacterized conserved protein	RBRH_02883	Pirin
RBRH_01044	Pirin-related protein	RBRH_01044	HemY protein
SG0735	Porphobilinogen deaminase	RBRH_03599	Hypothetical cytosolic protein
argH	Uncharacterized conserved protein	RBRH_00570	Citrate/L-malate proton symporter
ldcC	Argininosuccinate lyase	RBRH_03122	Biodegradative arginine decarboxylase
dcd	Arginine/lysine/ornithine decarboxylases	RBRH_03123	Deoxycytidine triphosphate deaminase
-	Predicted NTPase (NACHT family)	RBRH_03778	Multidrug resistance ABC transporter ATP-binding and permease protein
RBRH_00037	ABC-type transport system involved in Fe-S cluster assembly, permease and ATPase components	RBRH_00037	Unnamed protein product
RBRH_00037	ABC-type transport system involved in Fe-S cluster assembly, permease and ATPase components	RBRH_03777	Unnamed protein product
RBRH_03890	Hypothetical protein	RBRH_03890	Unnamed protein product
-	Hypothetical protein	RBRH_03890	Unnamed protein product
CAGGBEG34_550003	Nitrate reductase beta subunit	RBRH_03124	Superoxide dismutase [Cu-Zn]
mrp	Cu/Zn superoxide dismutase	RBRH_03125	Iron-sulfur cluster assembly/repair protein ApbC
-	ATPases involved in chromosome partitioning	RBRH_03126	OmpA family protein
metG	Hypothetical protein	RBRH_03128	Methionyl-tRNA synthetase
Bphyt_1014	Methionyl-tRNA synthetase	RBRH_03129	Hypothetical Outer Membrane Protein
-	Uncharacterized protein conserved in bacteria	RBRH_03130	Outer membrane protein
-	Outer membrane protein	RBRH_03131	Hypothetical protein
-	Hypothetical protein	RBRH_03132	Segregation and condensation protein Scp
panC	Uncharacterized conserved protein	RBRH_03133	Pantoate--beta-alanine ligase
panD	Panthothenate synthetase	RBRH_03134	Aspartate 1-decarboxylase
K788_8069	Aspartate 1-decarboxylase	RBRH_03137	Cobalamin-binding protein
-	Large exoproteins involved in heme utilization or adhesion	RBRH_03381	Transposase
aroC	Large exoproteins involved in heme utilization or adhesion	RBRH_02955	Chorismate synthase
-	Chorismate synthase	RBRH_02954	CBS domain containing protein
pdhD	FOG: CBS domain	RBRH_03818	Dihydrolipoamide dehydrogenase
aceE	Pyruvate/2-oxoglutarate dehydrogenase complex, dihydrolipoamide acyltransferase (E2) component, and related enzymes	RBRH_03816	Pyruvate dehydrogenase E1 component
folD	Pyruvate dehydrogenase complex, dehydrogenase (E1) component	RBRH_03810	Methylenetetrahydrofolate dehydrogenase (NADP+)
prlC	5,10-methylene-tetrahydrofolate dehydrogenase/Methenyl tetrahydrofolate cyclohydrolase	RBRH_03809	Oligopeptidase A
-	ABC-type branched-chain amino acid transport systems, periplasmic component	RBRH_03629	Multidrug resistance protein A
mutS	Uncharacterized conserved small protein	RBRH_03971	DNA mismatch repair protein mutS
slyD	Mismatch repair ATPase (MutS family)	RBRH_03973	Peptidyl-prolyl cis-trans isomerase
dapA	Uncharacterized lipoprotein	RBRH_03860	Dihydrodipicolinate synthase
trpS	Dihydrodipicolinate synthase/N-acetylneuraminate lyase	RBRH_03858	Tryptophanyl-tRNA synthetase
-	Tryptophanyl-tRNA synthetase	RBRH_03857	Membrane endopeptidase, M50 family
-	Zn-dependent proteases	RBRH_03855	PHP domain containing protein (TRPH)
-	Large-conductance mechanosensitive channel	RBRH_03820	D-alanyl-D-alanine-endopeptidase
tnpA2	Transposase and inactivated derivatives	RBRH_00904	Tnp
tnpA2	Transposase and inactivated derivatives	RBRH_03165	Tnp
-	Hypothetical protein	RBRH_02476	Transposase
gltX	DNA replication protein	RBRH_02893	Glutamyl-tRNA synthetase
-	ABC-type Fe3+ transport system, periplasmic component	RBRH_02891	NlpC/P60 family protein

-	Cell wall-associated hydrolases (invasion-associated proteins)	RBRH_02890	Oligopeptide transport ATP-binding protein oppD / Oligopeptide transport ATP-binding protein oppF
-	ABC-type uncharacterized transport system, duplicated ATPase component	RBRH_02889	Oligopeptide transport system permease protein oppC
-	ABC-type uncharacterized transport system, permease component	RBRH_02888	Oligopeptide transport system permease protein oppB
-	ABC-type uncharacterized transport system, permease component	RBRH_02887	Oligopeptide-binding protein oppA
fabI	ABC-type oligopeptide transport system, periplasmic component	RBRH_02885	Enoyl-[acyl-carrier protein] reductase (NADH)
-	Predicted NTPase (NACHT family)	RBRH_03797	Sulfide dehydrogenase precursor
glnA1	Rhodanese-related sulfurtransferase	RBRH_03799	Glutamine synthetase
XSR1_50074	Tellurite resistance protein and related permeases	RBRH_02910	Unnamed protein product
XSR1_50074	Tellurite resistance protein and related permeases	RBRH_00122	Unnamed protein product
XSR1_50074	Tellurite resistance protein and related permeases	RBRH_02048	Putative phage tail protein
-	Hypothetical protein	RBRH_03381	Transposase
-	Transposase and inactivated derivatives, IS5 family	RBRH_01603	BolA protein
-	Stress-induced morphogen (activity unknown)	RBRH_01602	Intracellular septation protein
-	Intracellular septation protein A	RBRH_01601	Peptide methionine sulfoxide reductase msrB
purL	Conserved domain frequently associated with peptide methionine sulfoxide reductase	RBRH_01605	Phosphoribosylformylglycinamidine synthase
pgi	Leucine-rich repeat (LRR) protein	RBRH_01609	Glucose-6-phosphate isomerase
RBRH_01614	Preprotein translocase subunit SecA (ATPase, RNA helicase)	RBRH_01614	Peptidyl-prolyl cis-trans isomerase
lon	Parvulin-like peptidyl-prolyl isomerase	RBRH_01622	ATP-dependent endopeptidase Lon
clpX	ATP-dependent Lon protease, bacterial type	RBRH_01623	ATP-dependent endopeptidase clp ATP-binding subunit clpX
clpP	ATP-dependent protease Clp, ATPase subunit	RBRH_01624	ATP-dependent endopeptidase clp proteolytic subunit clpP
tig	Protease subunit of ATP-dependent Clp proteases	RBRH_01625	Trigger factor, ppiase
trxA	Cytotoxic translational repressor of toxin-antitoxin stability system	RBRH_03750	Thioredoxin
rho	Thiol-disulfide isomerase and thioredoxins	RBRH_03753	Transcription termination factor rho
-	Ribosomal protein L31	RBRH_03758	Hypothetical protein
int	Permeases of the major facilitator superfamily	RBRH_01051	Unnamed protein product
CAGGBEG34_610004	Na+/citrate symporter	RBRH_01048	Unnamed protein product
-	Predicted NAD/FAD-binding protein	RBRH_01213	large terminase subunit
XFEB_01759	Uncharacterized protein conserved in bacteria	RBRH_01211	Unnamed protein product
L682_09905	Hypothetical protein	RBRH_01202	Unnamed protein product
BC1002_6556	Hypothetical protein	RBRH_01199	Unnamed protein product
HIB_15810	Hypothetical protein	RBRH_01196	Unnamed protein product
L682_09945	Hypothetical protein	RBRH_01195	phage-related protein
L682_09955	Hypothetical protein	RBRH_01193	Unnamed protein product
-	Hypothetical protein	RBRH_01189	Lysozyme
-	Hypothetical protein	RBRH_01186	Lysozyme
CAGGBEG34_220024	Hypothetical protein	RBRH_01185	Holin
relA	Predicted permeases	RBRH_02966	GTP pyrophosphokinase
etf	Guanosine polyphosphate pyrophosphohydrolases/synthetases	RBRH_02956	Electron transfer flavoprotein-ubiquinone oxidoreductase
-	Dehydrogenases (flavoproteins)	RBRH_03138	Transporter
-	ABC-type cobalamin/Fe3+-siderophores transport systems, ATPase components	RBRH_03139	Transporter
BURK_025795	ABC-type Fe3+-siderophore transport system, permease component	RBRH_03140	Vitamin B12 receptor
BYI23_A005940	Outer membrane cobalamin receptor protein	RBRH_03141	Unnamed protein product
-	Hypothetical protein	RBRH_03142	Hypothetical protein
lgt	Hypothetical protein	RBRH_03145	Prolipoprotein diacylglyceryl transferase
holC	Dihydroxyacid dehydratase/phosphogluconate dehydratase	RBRH_03149	DNA polymerase III, chi subunit
pepA	DNA polymerase III, chi subunit	RBRH_03150	Cytosol aminopeptidase
-	Leucyl aminopeptidase	RBRH_03151	Hypothetical membrane spanning protein
-	Predicted permeases	RBRH_03152	Hypothetical membrane spanning protein
Bphyt_0969	Uncharacterized conserved protein	RBRH_03160	Cys regulon transcriptional activator
Bphyt_0969	Uncharacterized conserved protein	RBRH_00145	Transcriptional regulator cbl
-	Predicted transcriptional regulators	RBRH_03055	Hydroxyacylglutathione hydrolase
ldcA	Guanosine polyphosphate pyrophosphohydrolases/synthetases	RBRH_03904	Muramoyltetrapeptide carboxypeptidase
-	Uncharacterized proteins, homologs of microcin C7 resistance protein MccF	RBRH_03905	tRNA-specific adenosine deaminase
-	Transposase and inactivated derivatives	RBRH_02476	Transposase
-	Transposase and inactivated derivatives	RBRH_03980	Transposase
-	Peroxiredoxin	RBRH_03402	smolarity sensor protein envZ
ompR	Signal transduction histidine kinase	RBRH_03403	Transcriptional regulatory protein ompR
rbfA	Pseudouridine synthase	RBRH_03638	Ribosome-binding factor A
nusA	Translation initiation factor 2 (IF-2; GTPase)	RBRH_03642	N utilization substance protein A
BUPH_06600	Transcriptional regulator	RBRH_03701	Unnamed protein product
-	Anaerobic dehydrogenases, typically selenocysteine-containing	RBRH_03703	Phosphoserine phosphatase
metC	Phosphoserine phosphatase	RBRH_03704	Cystathionine beta-lyase
rimO	Cystathionine beta-lyases/cystathionine gamma-synthases	RBRH_03707	tRNA 2-methylthioadenosine synthase homolog
rnr	2-methylthioadenine synthetase	RBRH_03602	Ribonuclease R
rlmB	Exoribonuclease R	RBRH_03601	23S rRNA Gm2251 methyltransferase
rpiA	rRNA methylases	RBRH_03598	Ribose 5-phosphate isomerase
-	Ribose 5-phosphate isomerase	RBRH_03714	Lipoprotein, ComL family
-	DNA uptake lipoprotein	RBRH_03713	Ribosomal large subunit pseudouridine synthase D
BgramDRAFT_2322	Pseudouridylate synthases, 23S RNA-specific	RBRH_03712	Hypothetical cytosolic protein
-	Uncharacterized conserved protein	RBRH_03708	Methyl-accepting chemotaxis protein
-	Hypothetical protein	RBRH_01051	Unnamed protein product
RBRH_04010	Transcriptional regulator	RBRH_04010	Phage protein
RBRH_01048	Hypothetical protein	RBRH_01048	Unnamed protein product
QWA_03220	Zn-dependent protease with chaperone function	RBRH_01047	Unnamed protein product
AcdelDRAFT_0859	Predicted transcriptional regulator	RBRH_01226	Regulatory protein CRO (antirepressor)
-	Uncharacterized protein conserved in bacteria	RBRH_03772	Hypothetical cytosolic protein
-	Acetyl-CoA carboxylase, carboxyltransferase component (subunits alpha and beta)	RBRH_01213	Large terminase subunit
BC1002_6544	Uncharacterized protein conserved in bacteria	RBRH_01211	Unnamed protein product
L682_09905	Hypothetical protein	RBRH_01202	Unnamed protein product
BC1002_6556	Hypothetical protein	RBRH_01199	Unnamed protein product
HIB_15810	Hypothetical protein	RBRH_01196	Unnamed protein product
L682_09945	Hypothetical protein	RBRH_01195	phage-related protein
L682_09955	Hypothetical protein	RBRH_01193	Unnamed protein product
-	Hypothetical protein	RBRH_01189	Lysozyme
-	Hypothetical protein	RBRH_01186	Lysozyme
CAGGBEG34_220024	Hypothetical protein	RBRH_01185	Holin
-	Predicted NTPase (NACHT family)	RBRH_01051	Unnamed protein product
tnpA2	Na+/citrate symporter	RBRH_00904	Tnp
tnpA2	Na+/citrate symporter	RBRH_03165	Tnp
CAGGBEG34_610004	Na+/citrate symporter	RBRH_01048	Unnamed protein product
-	Hypothetical protein	RBRH_01213	large terminase subunit
XFEB_01759	Uncharacterized protein conserved in bacteria	RBRH_01211	Unnamed protein product

L682_09905	Hypothetical protein	RBRH_01202	Unnamed protein product
BC1002_6556	Hypothetical protein	RBRH_01199	Unnamed protein product
HMPREF9095_1680	Hypothetical protein	RBRH_01196	Unnamed protein product
L682_09945	Hypothetical protein	RBRH_01195	phage-related protein
L682_09955	Hypothetical protein	RBRH_01193	Unnamed protein product
-	Hypothetical protein	RBRH_01189	Lysozyme
-	Hypothetical protein	RBRH_01186	Lysozyme
CAGGBEG34_220024	Hypothetical protein	RBRH_01185	Holin
-	Predicted GTPases	RBRH_01051	Unnamed protein product
CAGGBEG34_610004	Na+/citrate symporter	RBRH_01048	Unnamed protein product
-	Zn-dependent protease with chaperone function	RBRH_01213	large terminase subunit
-	Uncharacterized protein conserved in bacteria	RBRH_03766	Virulance-associated protein C
BC1002_6544	Uncharacterized protein conserved in bacteria	RBRH_01211	Unnamed protein product
L682_09905	Hypothetical protein	RBRH_01202	Unnamed protein product
BC1002_6556	Hypothetical protein	RBRH_01199	Unnamed protein product
HMPREF9095_1680	Hypothetical protein	RBRH_01196	Unnamed protein product
L682_09945	Hypothetical protein	RBRH_01195	phage-related protein
L682_09955	Hypothetical protein	RBRH_01193	Unnamed protein product
-	Hypothetical protein	RBRH_01189	Lysozyme
-	Hypothetical protein	RBRH_01186	Lysozyme
BC1002_6570	Hypothetical protein	RBRH_01185	Holin
	Predicted NTPase (NACHT family)	RBRH_01051	Unnamed protein product
CAGGBEG34_610004	Na+/citrate symporter	RBRH_01048	Unnamed protein product
-	ATPase components of ABC transporters with duplicated ATPase domains	RBRH_02476	Transposase
-	Transposase and inactivated derivatives	RBRH_03299	Transposase
-	Hypothetical protein	RBRH_02629	Plasmid stability protein stbB
-	Predicted nucleic acid-binding protein, contains PIN domain	RBRH_02630	Hypothetical protein
-	Integrase	RBRH_01549	Hypothetical protein
-	Putative hemolysin	RBRH_02330	Multimodular transpeptidase-transglycosylase PBP 1A
-	Putative hemolysin	RBRH_00133	Multimodular transpeptidase-transglycosylase PBP 1A
eno	Septum formation initiator	RBRH_03848	Enolase
kdsA	Enolase	RBRH_03846	2-dehydro-3-deoxyphosphooctonate aldolase
pyrG	3-deoxy-D-manno-octulosonic acid (KDO) 8-phosphate synthase	RBRH_03845	CTP synthase
ComA	CTP synthase (UTP-ammonia lyase)	RBRH_03842	DNA translocation competence protein ComA
lolD	Hypothetical protein	RBRH_03840	Lipoprotein releasing system ATP-binding protein lolD
-	ABC-type antimicrobial peptide transport system, ATPase component	RBRH_03839	Lipoprotein releasing system transmembrane protein lolC
BYI23_A017700	ABC-type transport system, involved in lipoprotein release, permease component	RBRH_03837	Regulatory protein
recJ	Uncharacterized protein conserved in bacteria	RBRH_03836	Single-stranded-DNA-specific exonuclease recJ
prfB	Single-stranded DNA-specific exonuclease	RBRH_03835	Bacterial Peptide Chain Release Factor 2 (RF-2)
lysS	Protein chain release factor B	RBRH_03834	Lysyl-tRNA synthetase
Dsui_2362	Lysyl-tRNA synthetase (class II)	RBRH_03833	Outer membrane lipoprotein
-	Outer membrane lipoprotein	RBRH_03802	Exodeoxyribonuclease III
BC1001_3356	Transposase and inactivated derivatives	RBRH_02729	Dipeptide-binding protein
serS	Transcriptional regulator	RBRH_01029	Seryl-tRNA synthetase
-	Seryl-tRNA synthetase	RBRH_01030	ATPase, AAA family
-	ATPase related to the helicase subunit of the Holliday junction resolvase	RBRH_01032	Outer-membrane lipoproteins carrier protein
ftsK	Outer membrane lipoprotein-sorting protein	RBRH_01033	Cell division protein ftsK
trxB2	DNA segregation ATPase FtsK/SpoIIIE and related proteins	RBRH_01034	Thioredoxin reductase
nadE	Hypothetical protein	RBRH_01038	Glutamine-dependent NAD(+) synthetase
secG	NAD synthase	RBRH_02523	Protein translocase subunit secG
tpiA	Preprotein translocase subunit SecG	RBRH_02522	Triosephosphate isomerase
Rleg4DRAFT_3636	Triosephosphate isomerase	RBRH_03955	Superfamily I DNA helicase
-	Superfamily I DNA and RNA helicases and helicase subunits	RBRH_02521	Quinone oxidoreductase
pnp	NADPH:quinone reductase and related Zn-dependent oxidoreductases	RBRH_02519	Polyribonucleotide nucleotidyltransferase
rpsO	Polyribonucleotide nucleotidyltransferase (polynucleotide phosphorylase)	RBRH_02518	SSU ribosomal protein S15P
-	Ribosomal protein S15P/S13E	RBRH_02516	Unnamed protein product
-	Predicted NTPase (NACHT family)	RBRH_02565	Hypothetical cytosolic protein
-	FOG: Ankyrin repeat	RBRH_02539	Hypothetical protein
nuoN	Hypothetical protein	RBRH_02538	NADH-quinone oxidoreductase chain N
nuoM	NADH:ubiquinone oxidoreductase subunit 2 (chain N)	RBRH_02537	NADH-quinone oxidoreductase chain M
nuoL	NADH:ubiquinone oxidoreductase subunit 4 (chain M)	RBRH_02536	NADH-quinone oxidoreductase chain L
nuoK	NADH:ubiquinone oxidoreductase subunit 5 (chain L)/Multisubunit Na+/H+ antiporter, MnhA subunit	RBRH_02535	NADH-quinone oxidoreductase chain K
nuoJ	NADH:ubiquinone oxidoreductase subunit 11 or 4L (chain K)	RBRH_02534	NADH-quinone oxidoreductase chain J
nuoI	NADH:ubiquinone oxidoreductase subunit 6 (chain J)	RBRH_02533	NADH-quinone oxidoreductase chain I
nuoH	Formate hydrogenlyase subunit 6/NADH:ubiquinone oxidoreductase 23 kD subunit (chain I)	RBRH_02532	NADH-quinone oxidoreductase chain H
nuoG	NADH:ubiquinone oxidoreductase subunit 1 (chain H)	RBRH_02530	NADH-quinone oxidoreductase chain G
nuoF	NADH dehydrogenase/NADH:ubiquinone oxidoreductase 75 kD subunit (chain G)	RBRH_02529	NADH-quinone oxidoreductase chain F
nuoE	NADH:ubiquinone oxidoreductase, NADH-binding (51 kD) subunit	RBRH_02528	NADH-quinone oxidoreductase chain E
nuoD	NADH:ubiquinone oxidoreductase 24 kD subunit	RBRH_02527	NADH-quinone oxidoreductase chain D
nuoC	NADH:ubiquinone oxidoreductase 49 kD subunit 7	RBRH_02526	NADH-quinone oxidoreductase chain C
nuoB	NADH:ubiquinone oxidoreductase 27 kD subunit	RBRH_02525	NADH-quinone oxidoreductase chain B
nuoA	NADH:ubiquinone oxidoreductase 20 kD subunit and related Fe-S oxidoreductases	RBRH_02524	NADH-quinone oxidoreductase chain A
RBRH_03658	NADH:ubiquinone oxidoreductase subunit 3 (chain A)	RBRH_03658	Hypothetical protein
-	Uncharacterized lipoprotein	RBRH_03796	Acriflavin resistance periplasmic protein
mdtB	Membrane-fusion protein	RBRH_03795	Acriflavin resistance plasma membrane protein
-	Cation/multidrug efflux pump	RBRH_03794	Acriflavin resistance plasma membrane protein
-	Growth inhibitor	RBRH_02835	Transcriptional regulator, MerR family
ihfA	Predicted transcriptional regulators	RBRH_02834	Integration host factor alpha-subunit
pheT	Bacterial nucleoid DNA-binding protein	RBRH_02833	Phenylalanyl-tRNA synthetase beta chain
pheS	Phenylalanyl-tRNA synthetase beta subunit	RBRH_02832	Phenylalanyl-tRNA synthetase alpha chain
rplT	Phenylalanyl-tRNA synthetase alpha subunit	RBRH_02831	LSU ribosomal protein L20P
rpmI	Ribosomal protein L20	RBRH_02830	LSU ribosomal protein L35P
infC	Ribosomal protein L35	RBRH_02829	Bacterial Protein Translation Initiation Factor 3 (IF-3)
thrS	Translation initiation factor 3 (IF-3)	RBRH_02828	Threonyl-tRNA synthetase
-	DNA uptake protein and related DNA-binding proteins	RBRH_01723	ADP-L-glycero-D-manno-heptose-6-epimerase
-	Nucleoside-diphosphate-sugar epimerases	RBRH_01722	D-glycero-D-manno-heptose-7-phosphate 1-kinase
-	ADP-heptose synthase, bifunctional sugar kinase/adenylyltransferase	RBRH_01721	UDP-glucose 6-dehydrogenase
-	Predicted UDP-glucose 6-dehydrogenase	RBRH_01720	Tetratricopeptide repeat family protein
SG0766	Uncharacterized integral membrane protein	RBRH_03599	Hypothetical cytosolic protein
ihfB	Glucoamylase and related glycosyl hydrolases	RBRH_01718	Integration host factor beta-subunit

rpsA	Bacterial nucleoid DNA-binding protein	RBRH_01717	SSU ribosomal protein S1P
cmk	Ribosomal protein S1	RBRH_01716	Cytidylate kinase
aroA	Cytidylate kinase	RBRH_01715	3-phosphoshikimate 1-carboxyvinyltransferase
tyrA	5-enolpyruvylshikimate-3-phosphate synthase	RBRH_01714	Prephenate dehydrogenase
pheA	Prephenate dehydrogenase	RBRH_01713	Chorismate mutase / Prephenate dehydratase
serC	Prephenate dehydratase	RBRH_01712	Phosphoserine aminotransferase
-	Phosphoserine aminotransferase	RBRH_01711	Unnamed protein product
gyrA	Hypothetical protein	RBRH_01710	DNA gyrase subunit A
-	Type IIA topoisomerase (DNA gyrase/topo II, topoisomerase IV), A subunit	RBRH_00055	Outer membrane protein
ubiG	Outer membrane protein and related peptidoglycan-associated (lipo)proteins	RBRH_00056	3-demethylubiquinone 3-methyltransferase
gph	2-polyprenyl-3-methyl-5-hydroxy-6-metoxy-1,4-benzoquinol methylase	RBRH_00057	Phosphoglycolate phosphatase
KYC_15302	Cell division protein FtsI/penicillin-binding protein 2	RBRH_00944	Outer membrane protein NOSA precursor
upk	Predicted S-adenosylmethionine-dependent methyltransferase	RBRH_01430	Undecaprenyl-diphosphatase
-	Hypothetical protein	RBRH_01434	Protein-L-isoaspartate O-methyltransferase
kdtA	Protein-L-isoaspartate carboxylmethyltransferase	waaA	3-deoxy-D-manno-octulosonic-acid transferase
rfaC	3-deoxy-D-manno-octulosonic-acid transferase	waaC	lipopolysaccharide heptosyltransferase-1
-	ADP-heptose:LPS heptosyltransferase	manB	phosphomannomutase
-	Phosphomannomutase	galE	UDP-glucose 4-epimerase
CAGGBEG34_200099	UDP-glucose 4-epimerase	wbiF	glycosyltransferase
-	SAM-dependent methyltransferases	wzt	polysaccharide export ATP-binding protein
Rta_08890	ABC-type polysaccharide/polyol phosphate transport system, ATPase component	wzm	polysaccharide export ABC transporter permease protein
wbpM	ABC-type polysaccharide/polyol phosphate export systems, permease component	wbiI	nucleotide sugar epimerase/dehydratase
CAGGBEG34_260027	Predicted nucleoside-diphosphate sugar epimerases	wbiH	UDP-N-acetylhexosamine-1-phosphate transferase
CAGGBEG34_190057	UDP-N-acetylmuramyl pentapeptide phosphotransferase/UDP-N-acetylglucosamine-1-phosphate transferase	wbiG	nucleotide sugar epimerase/dehydratase
nrdR	Nucleoside-diphosphate-sugar epimerases	RBRH_01535	Putative regulatory protein
glyA	Predicted transcriptional regulator, consists of a Zn-ribbon and ATP-cone domains	RBRH_01534	Serine hydroxymethyltransferase
-	Glycine/serine hydroxymethyltransferase	RBRH_01952	3-oxoacyl-[acyl-carrier protein] reductase
-	Short-chain alcohol dehydrogenase of unknown specificity	RBRH_01531	TolQ protein
tolR	Biopolymer transport proteins	RBRH_01530	TolR protein
Bphy_2454	Biopolymer transport protein	RBRH_01529	TolA protein
tolB	Hypothetical protein	RBRH_01528	TolB protein
pal	Periplasmic component of the Tol biopolymer transport system	RBRH_01527	Peptidoglycan-associated lipoprotein
-	Outer membrane protein and related peptidoglycan-associated (lipo)proteins	RBRH_01525	Tol system periplasmic component YbgF
-	Uncharacterized conserved protein	RBRH_01621	DNA-binding protein HU
-	Hypothetical protein	RBRH_01189	Lysozyme
-	Hypothetical protein	RBRH_01186	Lysozyme
CAGGBEG34_220024	Hypothetical protein	RBRH_01185	Holin
metB	Methyl-accepting chemotaxis protein	RBRH_00193	O-acetylhomoserine sulfhydrolase
purF	Cystathionine beta-lyases/cystathionine gamma-synthases	RBRH_00192	Amidophosphoribosyltransferase
-	Glutamine phosphoribosylpyrophosphate amidotransferase	RBRH_00191	Colicin V production protein
P355_3030	Uncharacterized membrane protein, required for colicin V production	RBRH_00190	Unnamed protein product
folC	Uncharacterized protein conserved in bacteria	RBRH_00189	Folylpolyglutamate synthase
accD	Folylpolyglutamate synthase	RBRH_00188	Acetyl-coenzyme A carboxylase carboxyl transferase subunit beta
trpA	Acetyl-CoA carboxylase beta subunit	RBRH_00187	Tryptophan synthase alpha chain
trpB	Tryptophan synthase alpha chain	RBRH_00185	Tryptophan synthase beta chain
-	Tryptophan synthase beta chain	RBRH_00184	N-(5'-phosphoribosyl)anthranilate isomerase
truA	Phosphoribosylanthranilate isomerase	RBRH_00183	tRNA pseudouridine synthase A
asd	Pseudouridylate synthase	RBRH_00180	Aspartate-semialdehyde dehydrogenase
leuB1	Aspartate-semialdehyde dehydrogenase	RBRH_00179	3-isopropylmalate dehydrogenase
gltA	Isocitrate/isopropylmalate dehydrogenase	RBRH_00175	Citrate synthase
-	Citrate synthase	RBRH_00174	Tetratricopeptide repeat family protein
sdhA	Succinate dehydrogenase/fumarate reductase, Fe-S protein subunit	RBRH_00172	Succinate dehydrogenase flavoprotein subunit
-	Succinate dehydrogenase/fumarate reductase, flavoprotein subunit	RBRH_00171	Succinate dehydrogenase membrane anchor subunit
SG0766	Uncharacterized conserved protein	RBRH_03599	Hypothetical cytosolic protein
-	Uncharacterized conserved protein	RBRH_00169	Transcriptional regulator, GntR family
mdh	Transcriptional regulators	RBRH_00167	Malate dehydrogenase
acnA	Hypothetical protein	RBRH_00163	Aconitate hydratase
thrC	Aconitase A	RBRH_03673	Threonine synthase
thrA	Threonine synthase	RBRH_03674	Homoserine dehydrogenase
aroE	Na+/citrate symporter	RBRH_03200	Shikimate 5-dehydrogenase
-	Shikimate 5-dehydrogenase	RBRH_03201	Exoribonuclease II
mpl	Exoribonuclease R	RBRH_03203	UDP-N-acetylmuramate:L-alanyl-gamma-D-glutamyl-meso-diaminopimelate ligase
aroQ	Thiol-disulfide isomerase and thioredoxins	RBRH_03207	3-dehydroquinate dehydratase
bccP	3-dehydroquinate dehydratase II	RBRH_03208	Biotin carboxyl carrier protein of acetyl-CoA carboxylase
accC	Biotin carboxyl carrier protein	RBRH_03209	Biotin carboxylase
prmA	Biotin carboxylase	RBRH_03210	Ribosomal protein L11 methyltransferase
tpx	Ribosomal protein L11 methylase	RBRH_03212	Thioredoxin peroxidase
-	Peroxiredoxin	RBRH_03213	Adenosine kinase
-	Sugar kinases, ribokinase family	RBRH_03214	Outer membrane lipoprotein
nrdB	Hypothetical protein	RBRH_03217	Ribonucleoside-diphosphate reductase beta chain
nrdA	Ribonucleotide reductase, beta subunit	RBRH_03219	Ribonucleoside-diphosphate reductase alpha chain
ampD	Ribonucleotide reductase, alpha subunit	RBRH_03220	Anhydro-N-acetylmuramyl-tripeptide amidase
-	Negative regulator of beta-lactamase expression	RBRH_03222	CcsA-related protein
ffh	ABC-type uncharacterized transport system, permease component	RBRH_03223	Signal recognition particle, subunit FFH/SRP54
-	Predicted NTPase (NACHT family)	RBRH_03225	MarC family integral membrane protein
proS	Multiple antibiotic transporter	RBRH_03226	Prolyl-tRNA synthetase
proB	NTP pyrophosphohydrolases including oxidative damage repair enzymes	RBRH_03230	Glutamate 5-kinase
obgE	Glutamate 5-kinase	RBRH_03231	GTP-binding protein CgtA (probably involved in DNA repair)
rpmA	Predicted GTPase	RBRH_03233	LSU ribosomal protein L27P
rplU	Ribosomal protein L27	RBRH_03234	LSU ribosomal protein L21P
-	Ribosomal protein L21	RBRH_01990	Cytochrome c4
-	Cytochrome c553	RBRH_01991	Cytochrome c
-	Cytochrome c, mono- and diheme variants	RBRH_01994	Hypothetical protein
-	2-methylthioadenine synthetase	RBRH_02006	Hypothetical metal-binding protein
-	Predicted metal-dependent hydrolase	RBRH_02007	Cation transport protein chaC
-	Uncharacterized protein involved in cation transport	RBRH_02008	Magnesium and cobalt efflux protein corC
lnt	Putative Mg2+ and Co2+ transporter CorC	RBRH_02009	Apolipoprotein N-acyltransferase
glyS	Glycyl-tRNA synthetase, alpha subunit	RBRH_02011	Glycyl-tRNA synthetase beta chain
ksgA	1-acyl-sn-glycerol-3-phosphate acyltransferase	RBRH_02051	Dimethyladenosine transferase
-	Parvulin-like peptidyl-prolyl isomerase	RBRH_02054	Organic solvent tolerance protein

A33K_14291	Nucleoside-diphosphate-sugar pyrophosphorylase involved in lipopolysaccharide biosynthesis/translation initiation factor 2B, gamma/epsilon subunits (eIF-2Bgamma/eIF-2Bepsilon)	RBRH_02057	2-polyprenyl-6-methoxyphenol hydroxylase
fis	2-polyprenyl-6-methoxyphenol hydroxylase and related FAD-dependent oxidoreductases	RBRH_02059	DNA-binding protein fis
purH	Factor for inversion stimulation Fis, transcriptional activator	RBRH_02060	Phosphoribosylaminoimidazolecarboxamide formyltransferase
ruvC	AICAR transformylase/IMP cyclohydrolase PurH (only IMP cyclohydrolase domain in Aful)	RBRH_02061	Crossover junction endodeoxyribonuclease ruvC
ruvA	Holliday junction resolvasome, endonuclease subunit	RBRH_02062	Holliday junction DNA helicase ruvA
ruvB	Holliday junction resolvasome, DNA-binding subunit	RBRH_02063	Holliday junction DNA helicase ruvB
tyrS	Holliday junction resolvasome, helicase subunit	RBRH_02065	Tyrosyl-tRNA synthetase
gcvP	Predicted molecular chaperone distantly related to HSP70-fold metalloproteases	RBRH_00844	Glycine dehydrogenase [decarboxylating]
-	Glycine cleavage system protein P (pyridoxal-binding), C-terminal domain	RBRH_00845	Glycine cleavage system H protein
gcvT	Glycine cleavage system H protein (lipoate-binding)	RBRH_00846	Aminomethyltransferase
-	Putative NADP-dependent oxidoreductases	RBRH_00075	Glutamine amidotransferase, class I
BURMUCGD1_0587	Uncharacterized protein conserved in bacteria	RBRH_01305	Esterase
-	Phospholipase/lecithinase/hemolysin	RBRH_01303	D-alanyl-meso-diaminopimelate endopeptidase
-	D-alanyl-D-alanine carboxypeptidase (penicillin-binding protein 4)	RBRH_01300	Hypothetical protein
SG0735	Hypothetical protein	RBRH_01306	Sua5/YciO/YrdC/YwlC family protein
purK	ATPases with chaperone activity, ATP-binding subunit	RBRH_03599	Hypothetical cytosolic protein
purE	Uncharacterized conserved protein	RBRH_01307	Phosphoribosylaminoimidazole carboxylase NCAIR mutase subunit
purC	Phosphoribosylaminoimidazole carboxylase (NCAIR synthetase)	RBRH_01308	Phosphoribosylaminoimidazole carboxylase carboxyltransferase subunit
fbaA	Phosphoribosylcarboxyaminoimidazole (NCAIR) mutase	RBRH_01309	Phosphoribosylamidoimidazole-succinocarboxamide synthase
pgk	Phosphoribosylaminoimidazolesuccinocarboxamide (SAICAR) synthase	RBRH_01310	Fructose-bisphosphate aldolase
-	Fructose/tagatose bisphosphate aldolase	RBRH_01314	Phosphoglycerate kinase
rfaF	3-phosphoglycerate kinase	RBRH_01318	Branched-chain amino acid aminotransferase
BRPE64_ACDS04830	Branched-chain amino acid aminotransferase/4-amino-4-deoxychorismate lyase	RBRH_01321	ADP-heptose--LPS heptosyltransferase II
-	ADP-heptose:LPS heptosyltransferase	RBRH_01322	Unnamed protein product
sucD	Hypothetical protein	RBRH_01328	Type 4 major prepilin protein pilA
Bcep1808_2762	Membrane protein TerC, possibly involved in tellurium resistance	RBRH_01237	Succinyl-CoA synthetase alpha chain
recA	Succinyl-CoA synthetase, beta subunit	RBRH_01976	Regulatory protein recX
-	Uncharacterized protein conserved in bacteria	RBRH_01977	RecA protein
-	RecA/RadA recombinase	RBRH_00353	Cytochrome b561
CAGGBEG34_290002	Uncharacterized paraquat-inducible protein A	RBRH_01670	Paraquat-inducible protein B
-	Paraquat-inducible protein B	RBRH_01669	Hypothetical protein
mnmA	Hypothetical protein	RBRH_01681	Adenylosuccinate lyase
-	Adenylosuccinate lyase	RBRH_01680	tRNA (5-methylaminomethyl-2-thiouridylate)-methyltransferase
proA	Lysine 2,3-aminomutase	RBRH_03176	Hypothetical membrane spanning protein
-	Predicted membrane protein	RBRH_03177	Gamma-glutamyl phosphate reductase
holA	Gamma-glutamyl phosphate reductase	RBRH_03178	DNA polymerase III, delta subunit
BYI23_A022000	DNA polymerase III, delta subunit	RBRH_03179	Rare lipoprotein B precursor
leuS	Rare lipoprotein B	RBRH_03180	Leucyl-tRNA synthetase
dapB	Leucyl-tRNA synthetase	RBRH_03182	Dihydrodipicolinate reductase
fur1	Small protein A (tmRNA-binding)	RBRH_03184	Ferric uptake regulation protein
tktA	Glyceraldehyde-3-phosphate dehydrogenase/erythrose-4-phosphate dehydrogenase	RBRH_03187	Transketolase
-	Transketolase	RBRH_02069	HesB protein family
rpsI	Uncharacterized conserved protein	RBRH_02070	SSU ribosomal protein S9P
rplM	Ribosomal protein S9	RBRH_02071	LSU ribosomal protein L13P
recG	Ribosomal protein L13	RBRH_01665	ATP-dependent DNA helicase recG
queA	RecG-like helicase	RBRH_01667	protein=S-adenosylmethionine:tRNA ribosyltransferase-isomerase
tgt	S-adenosylmethionine:tRNA-ribosyltransferase-isomerase (queuine synthetase)	RBRH_01668	Queuine tRNA-ribosyltransferase
secD	Preprotein translocase subunit YajC	RBRH_01676	Protein translocase subunit secD
secF	Preprotein translocase subunit SecD	RBRH_01675	Protein translocase subunit secF
A33K_15475	Preprotein translocase subunit SecF	RBRH_02721	Transcriptional regulators, LysR family
RSc2452	ABC-type dipeptide transport system, periplasmic component	RBRH_00366	Endonuclease
-	Phosphatidylserine/phosphatidylglycerophosphate/cardiolipin synthases and related enzymes	RBRH_00932	16S rRNA m(5)C 967 methyltransferase
purN	tRNA and rRNA cytosine-C5-methylases	RBRH_00933	Phosphoribosylglycinamide formyltransferase
ribF	Folate-dependent phosphoribosylglycinamide formyltransferase PurN	RBRH_00935	Riboflavin kinase
ileS	FAD synthase	RBRH_00936	Isoleucyl-tRNA synthetase
lspA	Isoleucyl-tRNA synthetase	RBRH_00937	Lipoprotein signal peptidase
dfp	Lipoprotein signal peptidase	RBRH_00938	Phosphopantothenoylcysteine decarboxylase
dut	Phosphopantothenoylcysteine synthetase/decarboxylase	RBRH_00940	Deoxyuridine 5'-triphosphate nucleotidohydrolase
lpxC	Uncharacterized conserved protein	RBRH_00949	UDP-3-O-[3-hydroxymyristoyl] N-acetylglucosamine deacetylase
ftsZ	UDP-3-O-acyl-N-acetylglucosamine deacetylase	RBRH_00951	Cell division protein ftsZ
ftsA	Cell division GTPase	RBRH_00952	Cell division protein ftsA
-	Actin-like ATPase involved in cell division	RBRH_00953	Cell division protein ftsQ
-	Cell division septal protein	RBRH_00954	D-alanine--D-alanine ligase
murC	D-alanine-D-alanine ligase and related ATP-grasp enzymes	RBRH_00955	UDP-N-acetylmuramate--alanine ligase
murG	UDP-N-acetylmuramate-alanine ligase	RBRH_00956	UDP-N-acetylglucosamine--N-acetylmuramyl-(pentapeptide) pyrophosphoryl-undecaprenol N-acetylglucosamine transferase
ftsW	UDP-N-acetylglucosamine:LPS N-acetylglucosamine transferase	RBRH_00957	Cell division protein ftsW
murD	Bacterial cell division membrane protein	RBRH_00958	UDP-N-acetylmuramoylalanine--D-glutamate ligase
mraY	UDP-N-acetylmuramoylalanine-D-glutamate ligase	RBRH_00959	Phospho-N-acetylmuramoyl-pentapeptide-transferase
-	UDP-N-acetylmuramyl pentapeptide phosphotransferase/UDP-N-acetylglucosamine-1-phosphate transferase	RBRH_00960	UDP-N-acetylmuramoyl-tripeptide--D-alanyl-D-alanine ligase
murE	UDP-N-acetylmuramyl pentapeptide synthase	RBRH_00961	UDP-N-acetylmuramoylalanyl-D-glutamate--2,6-diaminopimelate ligase
ftsI	UDP-N-acetylmuramyl tripeptide synthase	RBRH_00962	Division specific D,D-transpeptidase/Cell division protein ftsI
mraW	Cell division protein	RBRH_03264	S-adenosyl-methyltransferase mraW
mraZ	Predicted S-adenosylmethionine-dependent methyltransferase involved in cell envelope biogenesis	RBRH_03265	Cell division protein mraZ
-	Hypothetical protein	RBRH_03267	2-polyprenyl-3-methyl-6-methoxy-1,4-benzoquinol hydroxylase
RBRH_03270	Ubiquinone biosynthesis protein COQ7	RBRH_03270	Outer membrane porin protein 32 precursor
fadD1	Outer membrane protein (porin)	RBRH_03271	Long-chain-fatty-acid--CoA ligase
phoU	Acyl-CoA synthetases (AMP-forming)/AMP-acid ligases II	RBRH_01093	Phosphate transport system protein phoU
pstB	Phosphate uptake regulator	RBRH_01094	Phosphate transport ATP-binding protein pstB
pstA	ABC-type phosphate transport system, ATPase component	RBRH_01095	Phosphate transport system permease protein pstA
pstC	ABC-type phosphate transport system, permease component	RBRH_01096	Phosphate transport system permease protein pstC
-	ABC-type phosphate transport system, permease component	RBRH_01097	Phosphate-binding protein
glmM	FOG: WD40 repeat	RBRH_01098	Phosphoglucosamine mutase
folP	Phosphomannomutase	RBRH_01099	Dihydropteroate synthase
ftsH	Dihydropteroate synthase and related enzymes	RBRH_01100	Cell division protein ftsH
rrmJ	ATP-dependent Zn proteases	RBRH_01102	23S rRNA Um2552 2'-O-methyltransferase
-	23S rRNA methylase	RBRH_01103	Hypothetical RNA binding protein

greA	Predicted RNA-binding protein containing KH domain, possibly ribosomal protein	RBRH_01105	Transcription elongation factor greA
carA	Carbamoylphosphate synthase large subunit (split gene in MJ)	RBRH_01107	Carbamoyl-phosphate synthase small chain
-	Carbamoylphosphate synthase small subunit	RBRH_01113	Ribonuclease HI
-	Ribonuclease HI	RBRH_01112	Methyltransferase
dnaQ	Soluble lytic murein transglycosylase and related regulatory proteins (some contain LysM/invasin domains)	RBRH_01114	DNA polymerase III, epsilon chain
pyrC	Type IV secretory pathway, VirB11 components, and related ATPases involved in archaeal flagella biosynthesis	RBRH_02074	Dihydroorotase
-	Dihydroorotase	RBRH_00912	Branched-chain amino acid transport ATP-binding protein livF
-	ABC-type branched-chain amino acid transport systems, ATPase component	RBRH_00913	Branched-chain amino acid transport ATP-binding protein livG
-	ABC-type branched-chain amino acid transport systems, ATPase component	RBRH_00914	Branched-chain amino acid transport system permease protein livM
-	ABC-type branched-chain amino acid transport system, permease component	RBRH_00915	Branched-chain amino acid transport system permease protein livH
ispH	Branched-chain amino acid ABC-type transport system, permease components	RBRH_00920	4-hydroxy-3-methylbut-2-enyl diphosphate reductase
fkpB	Penicillin tolerance protein	RBRH_00921	Peptidyl-prolyl cis-trans isomerase
-	FKBP-type peptidyl-prolyl cis-trans isomerases 2	RBRH_00922	DNA repair protein radC
-	DNA repair proteins	RBRH_00923	LSU ribosomal protein L28P
rpmG	Ribosomal protein L28	RBRH_00925	LSU ribosomal protein L33P
nadB	Ribosomal protein L33	RBRH_00926	L-aspartate oxidase
rfbD	Predicted glycosyltransferases	rmlD	dTDP-4-dehydrorhamnose reductase
-	dTDP-4-dehydrorhamnose reductase	rmlC	dTDP-4-dehydrorhamnose 3,5-epimerase
-	dTDP-4-dehydrorhamnose 3,5-epimerase and related enzymes	rmlA	glucose-1-phosphate thymidylyltransferase
-	dTDP-glucose pyrophosphorylase	rmlB	dTDP-glucose 4,6-dehydratase
nadC	Glycosyltransferases involved in cell wall biogenesis	RBRH_00927	Nicotinate-nucleotide pyrophosphorylase
nadA	Nicotinate-nucleotide pyrophosphorylase	RBRH_00928	Quinolinate synthetase A
-	Quinolinate synthase	RBRH_00929	Acyl-CoA desaturase
-	Fatty-acid desaturase	RBRH_02153	Ferredoxin
coaD	Ferredoxin	RBRH_02154	Phosphopantetheine adenylyltransferase
-	Phosphopantetheine adenylyltransferase	RBRH_02155	Methyltransferase
-	Signal recognition particle GTPase	RBRH_02159	Fumarylpyruvate hydrolase
rpoH	Molecular chaperone (small heat shock protein)	RBRH_02167	RNA polymerase sigma-32 factor
clpA	Hypothetical protein	RBRH_00941	ATP-dependent clp protease ATP-binding subunit clpA
clpS	ATPases with chaperone activity, ATP-binding subunit	RBRH_00942	ATP-dependent Clp protease adaptor protein ClpS
cyoE	Uncharacterized protein SCO1/SenC/PrrC, involved in biogenesis of respiratory and photosynthetic systems	RBRH_02178	Protoheme IX farnesyltransferase
ctaA	Polyprenyltransferase (cytochrome oxidase assembly factor)	RBRH_02179	Heme O monooxygenase (ferredoxin)
Bphyt_0552	Uncharacterized protein required for cytochrome oxidase assembly	RBRH_02180	Hypothetical protein
-	Hypothetical protein	RBRH_02181	Cytochrome c oxidase assembly protein Surf1
coxC	Hypothetical protein	RBRH_02183	Cytochrome c oxidase polypeptide III
-	Heme/copper-type cytochrome/quinol oxidase, subunit 3	RBRH_02186	Cytochrome c oxidase polypeptide I
coxB	Heme/copper-type cytochrome/quinol oxidases, subunit 1	RBRH_02187	Cytochrome c oxidase polypeptide II
bioC	Heme/copper-type cytochrome/quinol oxidases, subunit 2	RBRH_04087	Biotin synthesis protein bioC
yibK	Predicted amidophosphoribosyltransferases	RBRH_02190	23S rRNA methyltransferase
gpsA	Predicted rRNA methylase (SpoU class)	RBRH_02191	Glycerol-3-phosphate dehydrogenase [NAD(P)+
secB	Glycerol-3-phosphate dehydrogenase	RBRH_02192	Protein translocase subunit secB
grxC	Preprotein translocase subunit SecB	RBRH_02193	Glutaredoxin
gpmA	Rhodanese-related sulfurtransferase	RBRH_02197	Phosphoglycerate mutase
thiF	Periplasmic protease	RBRH_02200	Molybdopterin biosynthesis MoeB protein
icd	Cold shock proteins	RBRH_00946	socitrate dehydrogenase [NADP]
sodB	Uncharacterized conserved protein	RBRH_04037	Superoxide dismutase
xseA	Superoxide dismutase	RBRH_03275	Exodeoxyribonuclease VII large subunit
-	Tetraacyldisaccharide-1-P 4'-kinase	RBRH_03277	conserved cytosolic protein
kdsB	Uncharacterized conserved protein	RBRH_03278	3-deoxy-manno-octulosonate cytidylyltransferase
adk	CMP-2-keto-3-deoxyoctulosonic acid synthetase	RBRH_03279	Adenylate kinase / Nucleoside-diphosphate kinase
mviN	Adenylate kinase and related kinases	RBRH_03318	Virulence factor mviN
rpsN	Uncharacterized membrane protein, putative virulence factor	RBRH_03319	SSU ribosomal protein S20P
argF	Hypothetical protein	RBRH_03322	Ornithine carbamoyltransferase
-	Ornithine carbamoyltransferase	RBRH_00290	Argininosuccinate synthase
murB	Argininosuccinate synthase	RBRH_03323	UDP-N-acetylenolpyruvoylglucosamine reductase
-	UDP-N-acetylmuramate dehydrogenase	RBRH_03324	Protein yajQ
h16_A0588	Uncharacterized protein conserved in bacteria	RBRH_03325	Hypothetical membrane spanning protein
ppa	Acyl-CoA synthetases (AMP-forming)/AMP-acid ligases II	RBRH_01042	Inorganic pyrophosphatase
xerD	Inorganic pyrophosphatase	RBRH_03335	Integrase/recombinase (XerD/RipX family)
ogt	Site-specific recombinase XerD	RBRH_03336	O6-methylguanine-DNA methyltransferase
-	Hypothetical protein	RBRH_03337	iron-sulfur cluster-binding protein
bgla_1g32700	Uncharacterized Fe-S protein	RBRH_03338	ATP/GTP hydrolase
amiA	Predicted ATPase or kinase	RBRH_03339	N-acetylmuramoyl-L-alanine amidase
dedA	N-acetylmuramoyl-L-alanine amidase	RBRH_03340	DedA family protein
mutL	Uncharacterized membrane-associated protein	RBRH_01632	DNA mismatch repair protein mutL
miaA	DNA mismatch repair enzyme (predicted ATPase)	RBRH_01633	tRNA delta(2)-isopentenylpyrophosphate transferase
purM	tRNA delta(2)-isopentenylpyrophosphate transferase	RBRH_01634	Phosphoribosylformylglycinamidine cyclo-ligase
-	Phosphoribosylaminoimidazole (AIR) synthetase	RBRH_01635	DnaA-related protein
-	ATPase involved in DNA replication initiation	RBRH_01636	Phosphoserine phosphatase
pcnB	Phosphoserine phosphatase	RBRH_01637	Poly(A) polymerase
-	DNA-binding protein H-NS	RBRH_02753	Leucine-, isoleucine-, valine-, threonine-, and alanine-binding protein
-	Hypothetical protein	RBRH_01969	Endonuclease/Exonuclease/phosphatase family protein]
-	Metal-dependent hydrolase	RBRH_00365	Ferredoxin--NADP reductase
-	Hypothetical protein	RBRH_00766	Hypophosphite dioxygenase
serA	DNA-binding protein H-NS	RBRH_01278	D-3-phosphoglycerate dehydrogenase
leuD	FOG: Ankyrin repeat	RBRH_00178	3-isopropylmalate dehydratase small subunit
leuC	3-isopropylmalate dehydratase small subunit	RBRH_00176	3-isopropylmalate dehydratase large subunit
ptsI	3-isopropylmalate dehydratase large subunit	RBRH_02201	Phosphoenolpyruvate-protein phosphotransferase
pstH	Phosphoenolpyruvate-protein kinase (PTS system EI component in bacteria)	RBRH_02202	Phosphocarrier protein HPr
-	Phosphotransferase system, HPr-related proteins	RBRH_02203	PTS system, IIA component
-	Phosphotransferase system, mannose/fructose-specific component IIA	RBRH_02207	Nitrogen regulatory protein GlnK
-	Nitrogen regulatory protein PII	RBRH_02209	Hypothetical protein
-	Uncharacterized protein conserved in bacteria	RBRH_02210	ATPase related to magnesium chelatase subunit ChlI
-	Uncharacterized conserved protein	RBRH_02229	Hypothetical protein
-	small molecule metabolism	RBRH_02238	Unnamed protein product
metHa	Hypothetical protein	RBRH_02239	5-methyltetrahydrofolate--homocysteine methyltransferase
metHb	Methionine synthase I (cobalamin-dependent), methyltransferase domain	RBRH_01971	5-methyltetrahydrofolate--homocysteine methyltransferase
-	Carbonic anhydrase	RBRH_03113	Dipeptide-binding protein
BgramDRAFT_1585	ABC-type dipeptide transport system, periplasmic component	RBRH_03115	Thiol:disulfide interchange protein dsbA

K788_4134	Thiol-disulfide isomerase and thioredoxins	RBRH_03116	Cell division protein ftsN
argS	Cell division protein	RBRH_01973	Arginyl-tRNA synthetase
WQE_03647	Arginyl-tRNA synthetase	RBRH_01972	Unnamed protein product
-	Transposase and inactivated derivatives	RBRH_02628	Transposase
trmE	Integrase	RBRH_00972	tRNA (5-carboxymethylaminomethyl-2-thiouridylate) synthase
phrB	Predicted GTPase	RBRH_03918	Deoxyribodipyrimidine photolyase
-	FOG: Ankyrin repeat	RBRH_03381	Transposase
-	FOG: Ankyrin repeat	RBRH_03381	Transposase
-	Transposase and inactivated derivatives, IS5 family	RBRH_02228	D-glycero-D-manno-heptose-1-phosphate adenylyltransferase
-	ADP-heptose synthase, bifunctional sugar kinase/adenylyltransferase	RBRH_02227	pantothenate kinase
BC1001_0218	Putative transcriptional regulator, homolog of Bvg accessory factor	RBRH_02226	Biotin operon repressor / Biotin--[acetyl-CoA-carboxylase
dppB	Biotin-(acetyl-CoA carboxylase) ligase	RBRH_02730	Dipeptide transport system permease protein dppB
-	ABC-type dipeptide/oligopeptide/nickel transport systems, permease components	RBRH_02731	Dipeptide transport system permease protein dppC
-	ABC-type dipeptide/oligopeptide/nickel transport systems, permease components	RBRH_02732	Dipeptide transport ATP-binding protein dppD
dppF	ABC-type dipeptide/oligopeptide/nickel transport system, ATPase component	RBRH_02733	Dipeptide transport ATP-binding protein dppF
-	Beta-lactamase class D	RBRH_02248	Queuosine biosynthesis protein QueC
-	Predicted PP-loop superfamily ATPase	RBRH_02247	Queuosine biosynthesis protein QueE
-	Organic radical activating enzymes	RBRH_02246	6-pyruvoyl tetrahydropterin synthase
mdrB	6-pyruvoyl-tetrahydropterin synthase	RBRH_02245	Rod shape-determining protein rodA
mrdA	Bacterial cell division membrane protein	RBRH_02244	Cell elongation specific D,D-transpeptidase
mreD	Cell division protein FtsI/penicillin-binding protein 2	RBRH_02243	Rod shape-determining protein mreD
mreC	Cell shape-determining protein	RBRH_02242	Rod shape-determining protein mreC
gatC	Actin-like ATPase involved in cell morphogenesis	RBRH_01001	Aspartyl/glutamyl-tRNA(Asn/Gln) amidotransferase subunit C
gatA	Asp-tRNAAsn/Glu-tRNAGln amidotransferase C subunit	RBRH_01000	Aspartyl/glutamyl-tRNA(Asn/Gln) amidotransferase subunit A
gatB	Asp-tRNAAsn/Glu-tRNAGln amidotransferase A subunit and related amidases	RBRH_00999	Aspartyl/glutamyl-tRNA(Asn/Gln) amidotransferase subunit B
-	Asp-tRNAAsn/Glu-tRNAGln amidotransferase B subunit (PET112 homolog)	RBRH_00998	Exodeoxyribonuclease III
BCh11DRAFT_04361	Exonuclease III	RBRH_00997	Zinc metalloprotease
-	Zn-dependent protease with chaperone function	RBRH_00996	AmpG protein
metW	Permeases of the major facilitator superfamily	RBRH_00995	Methyltransferase
argB	Homoserine acetyltransferase	RBRH_00991	Acetylglutamate kinase
hslU	Acetylglutamate kinase	RBRH_03413	ATP-dependent endopeptidase hsl ATP-binding subunit hslU
clpQ	ATP-dependent protease HslVU (ClpYQ), ATPase subunit	RBRH_03414	ATP-dependent endopeptidase hsl proteolytic subunit hslV
dksA	ATP-dependent protease HslVU (ClpYQ), peptidase subunit	RBRH_03416	DnaK suppressor protein
-	Cytochrome c551/c552	RBRH_03061	21 kDa hemolysin precursor
-	Predicted periplasmic or secreted lipoprotein	RBRH_03060	Phosphoheptose isomerase
-	Predicted endonuclease distantly related to archaeal Holliday junction resolvase	RBRH_03058	Tetrapyrrole (Corrin/Porphyrin) methylase family protein
-	Transposase and inactivated derivatives	RBRH_00882	Transposase
-	Transposase and inactivated derivatives	RBRH_01683	Transposase
-	Transposase and inactivated derivatives	RBRH_03170	Transposase
-	Transposase and inactivated derivatives	RBRH_01145	Transposase
lipA	18 : Unknown function	RBRH_02213	Lipoic acid synthetase
-	Lipoate-protein ligase B	RBRH_02217	Hypothetical protein
-	Branched-chain amino acid aminotransferase/4-amino-4-deoxychorismate lyase	RBRH_02218	D-alanyl-D-alanine serine-type carboxypeptidase
-	D-alanyl-D-alanine carboxypeptidase	RBRH_02220	Alpha/beta hydrolase
-	Ferredoxin	RBRH_01263	Unnamed protein product
-	Response regulator containing CheY-like receiver, AAA-type ATPase, and DNA-binding domains	RBRH_01264	Nitrogen regulation protein ntrY
-	Signal transduction histidine kinase involved in nitrogen fixation and metabolism regulation	RBRH_01265	Hypothetical protein
-	Hypothetical protein	RBRH_01266	16S rRNA m(5)C 967 methyltransferase
fmt	tRNA and rRNA cytosine-C5-methylases	RBRH_01268	Methionyl-tRNA formyltransferase
htpX	Methionyl-tRNA formyltransferase	RBRH_01267	Endopeptidase htpX
def1	4-amino-4-deoxy-L-arabinose transferase and related glycosyltransferases of PMT family	RBRH_01269	Peptide deformylase
-	Predicted Rossmann fold nucleotide-binding protein involved in DNA uptake	RBRH_01271	Thiol-disulfide isomerase and thioredoxins
topB	Thiol-disulfide isomerase and thioredoxins	RBRH_01273	DNA topoisomerase III
-	DNA-damage-inducible protein J	RBRH_00968	60 kDa inner membrane protein YIDC
		**pBRH01**	
-	FOG: HEAT repeat	RBRH_04258	Reverse transcriptase
Dehly_0278	Retron-type reverse transcriptase	RBRH_00621	Transposase
BamMC406_2351	Transposase and inactivated derivatives	RBRH_00447	Transposase
-	Transposase and inactivated derivatives	RBRH_00446	Transposase
-	Transposase and inactivated derivatives	RBRH_00621	Transposase
CAGGBEG34_210015	ABC-type branched-chain amino acid transport systems, periplasmic component	RBRH_03040	Transporter, drug/metabolite exporter family
CAGGBEG34_210015	ABC-type branched-chain amino acid transport systems, periplasmic component	RBRH_00303	Transporter, drug/metabolite exporter family
-	Valyl-tRNA synthetase	RBRH_00327	Hypothetical cytosolic protein
argA	Fe-S cluster protector protein	RBRH_00328	Amino-acid acetyltransferase
hrpA	Acetylglutamate kinase	RBRH_00329	ATP-dependent helicase hrpA
-	InterPro IPR001584 COGs COG2801	RBRH_04249	Transposase
-	Hypothetical protein	RBRH_00621	Transposase
RBRH_00570	Methyl-accepting chemotaxis protein	RBRH_00570	Citrate/L-malate proton symporter
-	Orotidine-5'-phosphate decarboxylase	RBRH_00570	Citrate/L-malate proton symporter
tnpA	Helicase subunit of the DNA excision repair complex	RBRH_00621	Transposase
L810_8411	Spermidine/putrescine-binding periplasmic protein	RBRH_00295	Transcriptional regulators, LysR family
BgramDRAFT_0582	DNA-binding HTH domain-containing proteins	hrpB	regulatory protein HrpB
BgramDRAFT_0573	Hypothetical protein	RBRH_03540	transcriptional regulatory protein
hpaB	Mannose-1-phosphate guanylyltransferase	hpaB	type III secretion protein HpaB
bgla_2g02110	Trypsin-like serine proteases, typically periplasmic, contain C-terminal PDZ domain	sctE	type III secretion protein SctE
bgla_2g02120	FOG: CBS domain	sctD	type III secretion protein SctD
BgramDRAFT_0557	Fe2+ transport system protein B	sctS	type III secretion inner membrane protein SctS
yscR	Type III secretory pathway, component EscS	sctR	type III secretion inner membrane protein SctR
PMI06_000610	Type III secretory pathway, component EscR	sctQ	type III secretion apparatus protein SctQ]
-	Hypothetical protein	sctV	type III secretion inner membrane protein SctV
sctU	Type III secretory pathway, component EscV	RBRH_00570	Citrate/L-malate proton symporter
BamMEX5DRAFT_4566	Uncharacterized protein conserved in bacteria	sctC	type III secretion outer membrane pore SctC
-	Uncharacterized conserved protein	RBRH_00334	Integral membrane protein
BCh11DRAFT_07142	Putative cation transport regulator	RBRH_00335	Exodeoxyribonuclease V gamma chain
WQE_32336	Exonuclease V gamma subunit	RBRH_03044	Exodeoxyribonuclease V beta chain

recD	ATP-dependent exoDNAse (exonuclease V) beta subunit (contains helicase and exonuclease domains)	RBRH_03043	Exodeoxyribonuclease V alpha chain
-	Electron transfer flavoprotein, alpha subunit	RBRH_03995	Acyl-CoA dehydrogenase
-	Uncharacterized protein conserved in bacteria	RBRH_02803	Potassium-transporting ATPase A chain
-	K+-transporting ATPase, A chain	RBRH_02804	Potassium-transporting ATPase B chain
-	High-affinity K+ transport system, ATPase chain B	RBRH_02805	Potassium-transporting ATPase C chain
RBRH_00570	DNA polymerase III, alpha subunit	RBRH_00570	Citrate/L-malate proton symporter
SG0735	Uracil-DNA glycosylase	RBRH_02783	Hypothetical cytosolic protein
thiD	Uncharacterized conserved protein	RBRH_00322	Phosphomethylpyrimidine kinase/ Hydroxymethylpyrimidine kinase
lpiT	Hydroxymethylpyrimidine/phosphomethylpyrimidine kinase	RBRH_00323	Lysophospholipid transporter family protein
-	Permeases of the major facilitator superfamily	RBRH_00324	Alanine racemase
sms	Alanine racemase	RBRH_00325	DNA repair protein RadA
RBRH_00570	Phenylpropionate dioxygenase and related ring-hydroxylating dioxygenases, large terminal subunit	RBRH_00570	Citrate/L-malate proton symporter
-	ABC-type spermidine/putrescine transport system, permease component II	RBRH_03496	Transporter
PACG_03516	ABC-type spermidine/putrescine transport system, permease component II	RBRH_03496	Transporter
dxs	Geranylgeranyl pyrophosphate synthase	RBRH_03430	1-deoxy-D-xylulose 5-phosphate synthase
RBRH_03529	Diacylglycerol kinase	RBRH_03529	Phosphoglycerol transferase MdoB and related proteins, alkaline phosphatase superfamily
SG0735	Porphobilinogen deaminase	RBRH_02783	Hypothetical cytosolic protein
RALTA_B0823	Spermidine/putrescine-binding periplasmic protein	RBRH_00295	Transcriptional regulators, LysR family
L810_8411	Spermidine/putrescine-binding periplasmic protein	RBRH_00295	Transcriptional regulators, LysR family
D769_12681	Transcriptional regulator	RBRH_00570	Citrate/L-malate proton symporter
hutH	Acyl-coenzyme A synthetases/AMP-(fatty) acid ligases	RBRH_02789	Histidine ammonia-lyase
mscL	Hypothetical protein	RBRH_00583	Large-conductance mechanosensitive channel
-	Dihydroorotate dehydrogenase	RBRH_00602	Dihydropyrimidine dehydrogenase [NADP+] alpha subunit
RBRH_00570	Low specificity phosphatase (HAD superfamily)	RBRH_00570	Citrate/L-malate proton symporter
-	Transposase and inactivated derivatives	RBRH_01902	Transposase
-	Transposase and inactivated derivatives	RBRH_01838	Transposase
ahpC1	Uncharacterized conserved protein	RBRH_03492	Peroxiredoxin
thiC	Uncharacterized protein conserved in bacteria	RBRH_03038	Thiamine biosynthesis protein thiC
-	Thiamine biosynthesis protein ThiC	RBRH_03039	Osmotically inducible lipoprotein B
C266_24825	Spermidine/putrescine-binding periplasmic protein	RBRH_00295	Transcriptional regulators, LysR family
RBRH_00570	Serine/threonine protein kinase	RBRH_00570	Citrate/L-malate proton symporter
RBRH_00570	Serine/threonine protein kinase	RBRH_00570	Citrate/L-malate proton symporter
RBRH_00570	Predicted exonuclease of the beta-lactamase fold involved in RNA processing	RBRH_00570	Citrate/L-malate proton symporter
-	Hypothetical protein	RBRH_00621	Transposase
-	Transposase and inactivated derivatives	RBRH_00621	Transposase
-	Hypothetical protein	RBRH_01691	Plasmid stability protein stbB
-	Predicted nucleic acid-binding protein, contains PIN domain	RBRH_01692	Hypothetical protein
AXXA_21803	Exonuclease III	RBRH_00295	Transcriptional regulators, LysR family
-	Transposase and inactivated derivatives	RBRH_00621	Transposase
KYC_09711	ABC-type dipeptide transport system, periplasmic component	RBRH_00295	Transcriptional regulators, LysR family
-	Transposase and inactivated derivatives	RBRH_00621	Transposase
BamMC406_2351	Transposase and inactivated derivatives	RBRH_00447	Transposase
-	Transposase and inactivated derivatives	RBRH_00446	Transposase
BgramDRAFT_0571	Threonyl-tRNA synthetase	sctT	type III secretion apparatus protein SctT
sctO	Type III secretory pathway, component EscT	sctO	type III secretion protein SctO
-	Hypothetical protein	sctN	type III secretion cytoplasmic ATPase SctN
A33K_17794	Flagellar biosynthesis/type III secretory pathway protein	sctK	type III secretion protein SctK
SG0766	Uncharacterized integral membrane protein	RBRH_02783	Hypothetical cytosolic protein
L810_8411	Hypothetical protein	RBRH_00295	Transcriptional regulators, LysR family
-	Hypothetical protein	RBRH_04249	Transposase
-	Hypothetical protein	RBRH_00446	Transposase
BamMC406_2351	Transposase and inactivated derivatives	RBRH_00447	Transposase
-	Transposase and inactivated derivatives	RBRH_01902	Transposase
-	Transposase and inactivated derivatives	RBRH_01838	Transposase
sdhB	Uncharacterized conserved protein	RBRH_02777	Succinate dehydrogenase iron-sulfur protein
SG0766	Uncharacterized conserved protein	RBRH_02783	Hypothetical cytosolic protein
-	Homoserine dehydrogenase	RBRH_00570	Citrate/L-malate proton symporter
SG0735	ATPases with chaperone activity, ATP-binding subunit	RBRH_02783	Hypothetical cytosolic protein
-	Predicted N6-adenine-specific DNA methylase	RBRH_00491	Aromatic amino acid transport protein aroP
-	Gamma-aminobutyrate permease and related permeases	RBRH_00422	Lysine 2,3-aminomutase
Bcep18194_B0308	Transcriptional regulator	RBRH_00570	Citrate/L-malate proton symporter
-	Di- and tricarboxylate transporters	RBRH_03031	Cyanide-insensitive cytochrome bd-type quinol oxidase subunit I
appB	Cytochrome bd-type quinol oxidase, subunit 1	RBRH_03032	Cyanide-insensitive cytochrome bd-type quinol oxidase subunit II
-	Rhs family protein	RBRH_00570	Citrate/L-malate proton symporter
tcaA2	Glyceraldehyde-3-phosphate dehydrogenase/erythrose-4-phosphate dehydrogenase	RBRH_00596	Insecticidal toxin complex protein TccA
-	Preprotein translocase subunit SecF	RBRH_01897	MazF protein
L810_8411	Undecaprenyl pyrophosphate synthase	RBRH_00295	Transcriptional regulators, LysR family
OR16_12515	Spermidine/putrescine-binding periplasmic protein	RBRH_00295	Transcriptional regulators, LysR family
-	Type IV secretory pathway, component VirB8	RBRH_01929	Transposase
C266_09527	Zn-dependent protease with chaperone function	RBRH_02758	Undecaprenyl-phosphate-4-amino-L-arabinose--lipidA 4-amino-L-arabinosyltransferase
		**pBRH02**	
Dehly_0278	Retron-type reverse transcriptase	RBRH_00722	Transposase
-	Transposase and inactivated derivatives	RBRH_00722	Transposase
-	FOG: HEAT repeat	RBRH_01694	Reverse transcriptase
-	InterPro IPR001584 COGs COG2801	RBRH_00630	Transposase
istB	Transposase and inactivated derivatives	RBRH_04297	Transposase
-	Hypothetical protein	RBRH_00722	Transposase
-	Uncharacterized protein conserved in bacteria	RBRH_00750	Hypothetical cytosolic protein
RBRH_00570	Methyl-accepting chemotaxis protein	RBRH_01258	Citrate/L-malate proton symporter
-	Orotidine-5'-phosphate decarboxylase	RBRH_01258	Citrate/L-malate proton symporter
tnpA	Helicase subunit of the DNA excision repair complex	RBRH_00722	Transposase
-	DNA-directed RNA polymerase, sigma subunit (sigma70/sigma32)	RBRH_01252	Transposase
-	Signal transduction histidine kinase	RBRH_00667	Unnamed protein product
tnpA	Predicted NTPase (NACHT family)	RBRH_00685	Transposase
-	Transposase and inactivated derivatives	RBRH_04292	Transposase
-	Glucose-6-phosphate isomerase	RBRH_04292	Transposase
-	Transposase and inactivated derivatives	RBRH_00685	Transposase
RBRH_00570	DNA polymerase III, alpha subunit	RBRH_01258	Citrate/L-malate proton symporter
SG0735	Uracil-DNA glycosylase	RBRH_00687	Hypothetical cytosolic protein
RBRH_00570	Phenylpropionate dioxygenase and related ring-hydroxylating dioxygenases, large terminal subunit	RBRH_01258	Citrate/L-malate proton symporter

-	ABC-type spermidine/putrescine transport systems, ATPase components	RBRH_01252	Transposase
-	Hypothetical protein	RBRH_01787	Hypothetical cytosolic protein
-	Nucleotidyltransferase/DNA polymerase involved in DNA repair	RBRH_01786	DNA polymerase III alpha subunit
SG0735	Porphobilinogen deaminase	RBRH_00687	Hypothetical cytosolic protein
D769_12681	Transcriptional regulator	RBRH_01258	Citrate/L-malate proton symporter
-	Large exoproteins involved in heme utilization or adhesion	RBRH_01252	Transposase
-	Hypothetical protein	RBRH_01252	Transposase
RBRH_00570	Low specificity phosphatase (HAD superfamily)	RBRH_01258	Citrate/L-malate proton symporter
-	Transposase and inactivated derivatives	RBRH_00741	Transposase
RBRH_00570	Serine/threonine protein kinase	RBRH_01258	Citrate/L-malate proton symporter
-	Uncharacterized protein conserved in bacteria	RBRH_00676	Hypothetical cytosolic protein
-	Predicted transcriptional regulator	RBRH_00677	Hypothetical cytosolic protein
RBRH_00570	Serine/threonine protein kinase	RBRH_01258	Citrate/L-malate proton symporter
RBRH_00570	Predicted exonuclease of the beta-lactamase fold involved in RNA processing	RBRH_01258	Citrate/L-malate proton symporter
-	Hypothetical protein	RBRH_00722	Transposase
-	Transposase and inactivated derivatives	RBRH_00722	Transposase
-	Transposase and inactivated derivatives	RBRH_00722	Transposase
-	Transposase and inactivated derivatives	RBRH_00722	Transposase
SG0766	Uncharacterized integral membrane protein	RBRH_00687	Hypothetical cytosolic protein
-	Hypothetical protein	RBRH_00630	Transposase
istB	Transposase and inactivated derivatives	RBRH_04297	Transposase
-	Transposase and inactivated derivatives	RBRH_00741	Transposase
SG0766	Uncharacterized conserved protein	RBRH_00687	Hypothetical cytosolic protein
-	Homoserine dehydrogenase	RBRH_01258	Citrate/L-malate proton symporter
SG0735	ATPases with chaperone activity, ATP-binding subunit	RBRH_00687	Hypothetical cytosolic protein
Bcep18194_B0308	Transcriptional regulator	RBRH_01258	Citrate/L-malate proton symporter
-	Preprotein translocase subunit SecF	RBRH_03361	MazF protein
-	UDP-N-acetylmuramate-alanine ligase	RBRH_01252	Transposase
-	Bacterial cell division membrane protein	RBRH_01252	Transposase
-	Type IV secretory pathway, component VirB8	RBRH_00717	Transposase

Supplemental Table S2. Comparison of B1-EB^T, Mc-AG77, and HKI 454^T genomes. Specific proteins encoded by genes in the B1-EB^T genome are colored (legends for the colored rows).

B1-EB^T			% identity		
**Genes**	**Name**	**Annotation**	**B1-EB^T - Mc-AG77**	**B1-EB^T - HKI454^T**	**Mc-AG77 - HKI454^T**
MCB1EB_1	dnaA	ATPase involved in DNA replication initiation	99	77	100
MCB1EB_2	dnaN	DNA polymerase sliding clamp subunit (PCNA homolog)	100	76	100
MCB1EB_2	gyrB	DNA polymerase sliding clamp subunit (PCNA homolog)	100	76	100
MCB1EB_3	Sinac_2044	Type IIA topoisomerase (DNA gyrase/topo II, topoisomerase IV), B subunit	99	80	100
MCB1EB_4	-	Response regulator of citrate/malate metabolism	-	-	-
MCB1EB_5	CICLE_v10014636mg	Site-specific recombinases, DNA invertase Pin homologs	-	-	-
MCB1EB_6	HCAG_07455	Hypothetical protein	-	-	-
MCB1EB_7	-	FOG: HEAT repeat	-	-	-
MCB1EB_8	Dehly_0278	Retron-type reverse transcriptase	-	53	-
MCB1EB_9	-	Transposase and inactivated derivatives	-	27-35	-
MCB1EB_10	-	Hypothetical protein	99	81	93
MCB1EB_11	-	Proteins containing SET domain	97	-	100
MCB1EB_12	-	Uncharacterized conserved protein	99	74	99
MCB1EB_13	gidA	Hypothetical protein	99	67	100
MCB1EB_14	gidB	NAD/FAD-utilizing enzyme apparently involved in cell division	99	80	98
MCB1EB_15	parA2	Predicted S-adenosylmethionine-dependent methyltransferase involved in bacterial cell division	99	59	97
MCB1EB_16	parB2	ATPases involved in chromosome partitioning	100	80	100
MCB1EB_17	-	Predicted transcriptional regulators	99	74	98
MCB1EB_18	-	Type II secretory pathway, component ExeA (predicted ATPase)	-	-	-
MCB1EB_19	BamMC406_2351	Transposase and inactivated derivatives	-	-	-
MCB1EB_20	-	Transposase and inactivated derivatives	99	44	98
MCB1EB_21	-	Transposase and inactivated derivatives	95	66	-
MCB1EB_22	-	Transposase and inactivated derivatives	-	26-47	-
MCB1EB_23	-	Transposase and inactivated derivatives	91-100	-	-
MCB1EB_24	HCAG_07455	Site-specific recombinases, DNA invertase Pin homologs	94	-	-
MCB1EB_25	N47_J00140	FOG: HEAT repeat	61-77	-	-
MCB1EB_26	-	Hypothetical protein	-	-	-
MCB1EB_27	MTR_7g052740	Transposase and inactivated derivatives	-	-	-
MCB1EB_28	M23134_05452	Fe-S oxidoreductase	97	-	-
MCB1EB_29	HacjB3_01980	Leucine-rich repeat (LRR) protein	97	-	-
MCB1EB_30	BURK_029400	Branched-chain amino acid aminotransferase/4-amino-4-deoxychorismate lyase	94	-	-
MCB1EB_31	atpF0A	Hypothetical protein	99	-	-
MCB1EB_32	atpF0C	F0F1-type ATP synthase, subunit a	100	74	99
MCB1EB_33	atpF0B	F0F1-type ATP synthase, subunit c/Archaeal/vacuolar-type H+-ATPase, subunit K	100	97	98
MCB1EB_34	atpF1D	F0F1-type ATP synthase, subunit b	99	70	100
MCB1EB_35	atpF1A	F0F1-type ATP synthase, delta subunit (mitochondrial oligomycin sensitivity protein)	99	58	100
MCB1EB_36	atpF1G	F0F1-type ATP synthase, alpha subunit	100	-	-
MCB1EB_37	atpF1B	F0F1-type ATP synthase, gamma subunit	99	79	100
MCB1EB_38	atpC	F0F1-type ATP synthase, beta subunit	99	94	100
MCB1EB_39	fumC	F0F1-type ATP synthase, epsilon subunit (mitochondrial delta subunit)	100	83	99
MCB1EB_40	hemE	Fumarase	99	82	98
MCB1EB_41	priA	Uroporphyrinogen-III decarboxylase	99	78	99
MCB1EB_42	putA	Primosomal protein N' (replication factor Y) - superfamily II helicase	99	-	-
MCB1EB_43	-	Delta 1-pyrroline-5-carboxylate dehydrogenase	99	68	99
MCB1EB_44	CAGGBEG34_210015	ABC-type branched-chain amino acid transport systems, periplasmic component	99	70	100
MCB1EB_45	PMI16_02037	Permeases of the drug/metabolite transporter (DMT) superfamily	99	44-48	-
MCB1EB_46	-	Predicted Na+-dependent transporter	99	52	91
MCB1EB_47	bioB	Dioxygenases related to 2-nitropropane dioxygenase	99	-	-
MCB1EB_48	bioD	Biotin synthase and related enzymes	100	66	98
MCB1EB_49	bioF	Dethiobiotin synthetase	100	-	-
MCB1EB_50	bioA	7-keto-8-aminopelargonate synthetase and related enzymes	100	-	-
MCB1EB_51	mnmC	Adenosylmethionine-8-amino-7-oxononanoate aminotransferase	100	73	99
MCB1EB_52	-	Uncharacterized conserved protein	100	52	95
MCB1EB_53	-	Bacterial nucleoid DNA-binding protein	100	72	91
MCB1EB_54	gspD	Soluble lytic murein transglycosylase and related regulatory proteins (some contain LysM/invasin domains)	100	-	-
MCB1EB_55	gspE	Type II secretory pathway, component PulD	100	60	100
MCB1EB_56	gspF	Type II secretory pathway, ATPase PulE/Tfp pilus assembly pathway, ATPase PilB	100	80	100
MCB1EB_57	bglu_1g00350	Type II secretory pathway, component PulF	100	78	100
MCB1EB_58	gspG	Hypothetical protein	98	45	100
MCB1EB_59	BC1003_3493	Type II secretory pathway, pseudopilin PulG	100	-	-
MCB1EB_60	gspI	Type II secretory pathway, pseudopilin PulG	100	59	83
MCB1EB_61	BC1003_3495	Type II secretory pathway, pseudopilin PulG	90	53	100
MCB1EB_62	Bcep1808_0062	Type II secretory pathway, component PulJ	100	51	100
MCB1EB_63	Bxe_A4492	Type II secretory pathway, component PulK	100	49	97
MCB1EB_64	BC1003_3498	Hypothetical protein	100	43	97
MCB1EB_65	BCh11DRAFT_04780	Type II secretory pathway, component PulM	100	50	99
MCB1EB_66	-	Hypothetical protein	100	49	95
MCB1EB_67	-	Permeases of the major facilitator superfamily	100	59	95
MCB1EB_68	C664_08243	Outer membrane protein	100	76	90
MCB1EB_69	metF	Predicted ATPase (AAA+ superfamily)	100	-	-
MCB1EB_70	ahcY	5,10-methylenetetrahydrofolate reductase	100	84	99
MCB1EB_71	-	S-adenosylhomocysteine hydrolase	100	85	97
MCB1EB_72	-	Amino acid transporters	100	78	74
MCB1EB_73	-	S-adenosylmethionine synthetase	100	63	100
MCB1EB_74	dapF	Lauroyl/myristoyl acyltransferase	100	71	92
MCB1EB_75	Bphy_0075	Diaminopimelate epimerase	100	63	96
MCB1EB_76	xerC	Uncharacterized protein conserved in bacteria	100	45	97
MCB1EB_77	-	Site-specific recombinase XerC	100	67	97
MCB1EB_78	-	Predicted membrane protein	100	72	100
MCB1EB_79	-	Predicted membrane protein	100	76	95
MCB1EB_80	-	Uncharacterized proteins, homologs of lactam utilization protein B	100	66	97
MCB1EB_81	-	Allophanate hydrolase subunit 2	100	61	97
MCB1EB_82	CAGGBEG34_20043	Allophanate hydrolase subunit 1	100	63	97
MCB1EB_83	-	5-formyltetrahydrofolate cyclo-ligase	100	45	95
MCB1EB_84	cca	Soluble lytic murein transglycosylase and related regulatory proteins (some contain LysM/invasin domains)	100	57	96
MCB1EB_85	-	tRNA nucleotidyltransferase/poly(A) polymerase	100	72	40
MCB1EB_86	-	Uncharacterized conserved protein	100	62	100
MCB1EB_87	-	Dihydroneopterin aldolase	100	61	98
MCB1EB_88	glmU	Predicted ATPase of the PP-loop superfamily implicated in cell cycle control	98	80	92

MCB1EB_89	glmS	N-acetylglucosamine-1-phosphate uridyltransferase (contains nucleotidyltransferase and I-patch acetyltransferase domains)	100	67	100
MCB1EB_90	argC-1	Glucosamine 6-phosphate synthetase, contains amidotransferase and phosphosugar isomerase domains	100	82	100
MCB1EB_91	pyrE	Acetylglutamate semialdehyde dehydrogenase	99	68	99
MCB1EB_92	maeB	Orotate phosphoribosyltransferase	100	78	99
MCB1EB_93	-	Malic enzyme	98-100	63-83	-
MCB1EB_94	-	Hypothetical protein	100	-	-
MCB1EB_95	Bpet0683	Uncharacterized protein conserved in archaea	-	-	-
MCB1EB_96	oxyR	Hypothetical protein	-	-	-
MCB1EB_97	-	Transcriptional regulator	100	82	99
MCB1EB_98	ubiA	DNA-binding ferritin-like protein (oxidative damage protectant)	100	-	-
MCB1EB_99	PMI40_00445	4-hydroxybenzoate polyprenyltransferase and related prenyltransferases	100	67	99
MCB1EB_100	Bbu156a_Z31	Hypothetical protein	100	-	-
MCB1EB_101	proC	Hypothetical protein	-	-	-
MCB1EB_102	-	Pyrroline-5-carboxylate reductase	100	65	96
MCB1EB_103	aroF	Predicted enzyme with a TIM-barrel fold	100	68	97
MCB1EB_104	tldD	3-deoxy-D-arabino-heptulosonate 7-phosphate (DAHP) synthase	100	81	100
MCB1EB_105	Bxe_A3970	Predicted Zn-dependent proteases and their inactivated homologs	100	78	100
MCB1EB_106	glnE	Predicted membrane protein	98	37	98
MCB1EB_107	recN	Glutamine synthetase adenylyltransferase	100	63	99
MCB1EB_108	ppnK	ATPase involved in DNA repair	100	68	100
MCB1EB_109	hrcA	Predicted sugar kinase	100	72	89
MCB1EB_110	-	Transcriptional regulator of heat shock gene	100	82	100
MCB1EB_111	hemH	Hypothetical protein	28	-	-
MCB1EB_112	grpE	Protoheme ferro-lyase (ferrochelatase)	100	65	99
MCB1EB_113	dnaK	Molecular chaperone GrpE (heat shock protein)	100	-	-
MCB1EB_114	dnaJ	Molecular chaperone	100	78	100
MCB1EB_115	BC1001_0454	DnaJ-class molecular chaperone with C-terminal Zn finger domain	100	77	100
MCB1EB_116	SMUL_0469	Anthranilate/para-aminobenzoate synthases component I	100	53	99
MCB1EB_117	-	Isoleucyl-tRNA synthetase	100	-	-
MCB1EB_118	-	Topoisomerase IA	-	38	-
MCB1EB_119	HCAG_07455	Plasmid stabilization system protein	-	54	-
MCB1EB_120	CAOG_00447	Hypothetical protein	-	-	-
MCB1EB_121	PBF_15929	DNA segregation ATPase FtsK/SpoIIIE and related proteins	-	-	-
MCB1EB_122	TSTA_111040	DnaJ-class molecular chaperone	-	-	-
MCB1EB_123	-	FOG: HEAT repeat	-	-	-
MCB1EB_124	-	Retron-type reverse transcriptase	-	60	-
MCB1EB_125	HNWD1	Uncharacterized protein conserved in bacteria	-	-	-
MCB1EB_126	F750_6439	Predicted NTPase (NACHT family)	-	-	-
MCB1EB_127	FGSG_13479	Predicted NTPase (NACHT family)	92	-	-
MCB1EB_128	N234_15805	FOG: HEAT repeat	68-81	-	-
MCB1EB_129	Bcep1808_2462	Transposase and inactivated derivatives	-	-	-
MCB1EB_130	ACD_45C00610G0002	Hypothetical protein	-	-	-
MCB1EB_131	K788_3283	Hypothetical protein	-	-	-
MCB1EB_132	Rpic12D_3451	Uncharacterized protein conserved in bacteria	-	-	-
MCB1EB_133	Bphy_1935	Hypothetical protein	-	-	-
MCB1EB_134	RSc1870	Predicted transcriptional regulator	-	-	-
MCB1EB_135	-	Hypothetical protein	-	-	-
MCB1EB_136	cinA	Integrase	97	-	-
MCB1EB_137	-	Uncharacterized protein (competence- and mitomycin-induced)	100	75	99
MCB1EB_138	thiL	Phosphatidylglycerophosphatase A and related proteins	-	60	-
MCB1EB_139	maeB	Thiamine monophosphate kinase	-	61	-
MCB1EB_140	-	Malic enzyme	98-100	82	-
MCB1EB_141	-	Hypothetical protein	100	61	95
MCB1EB_142	-	Uncharacterized protein conserved in bacteria	100	61	98
MCB1EB_143	nth	Hypothetical protein	100	68	100
MCB1EB_144	rnfB	Predicted EndoIII-related endonuclease	100	84	93
MCB1EB_145	-	Predicted NADH:ubiquinone oxidoreductase, subunit RnfB	100	-	-
MCB1EB_146	-	3-polyprenyl-4-hydroxybenzoate decarboxylase and related decarboxylases	100	79	98
MCB1EB_147	nusB	Soluble lytic murein transglycosylase and related regulatory proteins (some contain LysM/invasin domains)	83	-	-
MCB1EB_148	ribH	Transcription termination factor	100	-	-
MCB1EB_149	ribA	Riboflavin synthase beta-chain	100	73	90
MCB1EB_150	ribE	3,4-dihydroxy-2-butanone 4-phosphate synthase	100	75	98
MCB1EB_151	ribD	Riboflavin synthase alpha chain	100	65	98
MCB1EB_152	hemL	Pyrimidine reductase, riboflavin biosynthesis	100	71	96
MCB1EB_153	-	Glutamate-1-semialdehyde aminotransferase	100	75	100
MCB1EB_154	-	ABC-type histidine transport system, ATPase component	100	79	100
MCB1EB_155	-	ABC-type arginine/histidine transport system, permease component	100	-	-
MCB1EB_156	-	ABC-type arginine transport system, permease component	100	76	100
MCB1EB_157	ssuC	ABC-type amino acid transport/signal transduction systems, periplasmic component/domain	100	70	99
MCB1EB_158	-	ABC-type anion transport system, duplicated permease component	94	-	-
MCB1EB_159	alaS	ABC-type nitrate/sulfonate/bicarbonate transport system, ATPase component	100	56	99
MCB1EB_160	sirA	Alanyl-tRNA synthetase	97	82	97
MCB1EB_161	-	Predicted redox protein, regulator of disulfide bond formation	100	73	95
MCB1EB_162	valS	UDP-glucose pyrophosphorylase	100	79	98
MCB1EB_163		Valyl-tRNA synthetase	100	82	99
MCB1EB_164	argA	Fe-S cluster protector protein	100	84	100
MCB1EB_165	hrpA	Acetylglutamate kinase	100	75	100
MCB1EB_166	-	HrpA-like helicases	100	68	97
MCB1EB_167	N9414_23838	Transcriptional regulator	100	79	97
MCB1EB_168	MldDRAFT_4528	Hypothetical protein	100	-	-
MCB1EB_169	pyrD	Hypothetical protein	100	-	-
MCB1EB_170	-	Dihydroorotate dehydrogenase	100	77	94
MCB1EB_171	aat	ADP-ribose pyrophosphatase	100	72	98
MCB1EB_172	-	Leu/Phe-tRNA-protein transferase	100	67	98
MCB1EB_173	-	Single-stranded DNA-binding protein	100	58	100
MCB1EB_174	uvrA	Permeases of the major facilitator superfamily	100	65	96
MCB1EB_175	CAGGBEG34_50005	Excinuclease ATPase subunit	100	83	99
MCB1EB_176	HBHAL_1466	Predicted permease	100	52	94
MCB1EB_177	dsat_1931	Methyl-accepting chemotaxis protein	100	-	-
MCB1EB_178	SsaD	Predicted hydrolases or acyltransferases (alpha/beta hydrolase superfamily)	100	-	-
MCB1EB_179	BN873_490019	Hypothetical protein	100	-	-
MCB1EB_180	escC	5-enolpyruvylshikimate-3-phosphate synthase	100	-	-
MCB1EB_181	CAGGBEG34_210108	Type II secretory pathway, component PulD	100	-	-
MCB1EB_182	SsaH	Predicted Fe-S oxidoreductase	100	-	-
MCB1EB_183	CAGGBEG34_210106	Hypothetical protein	100	-	-
MCB1EB_184	ssaJ	Peptide chain release factor RF-3	100	-	-

MCB1EB_185	CAGGBEG34_210103	Type III secretory pathway, lipoprotein EscJ	100	-	-
MCB1EB_186	SsaK	Hypothetical protein	88	-	-
MCB1EB_187	-	Hypothetical protein	100	-	-
MCB1EB_188	fliI	Type III secretory pathway, component EscV	100	-	-
MCB1EB_189	CAGGBEG34_210094	Flagellar biosynthesis/type III secretory pathway ATPase	100	-	-
MCB1EB_190	CAGGBEG34_210093	Hypothetical protein	100	-	-
MCB1EB_191	PscQ	Phenylalanyl-tRNA synthetase beta subunit	100	-	-
MCB1EB_192	escR	Flagellar motor switch/type III secretory pathway protein	100	-	-
MCB1EB_193	SsaS	Type III secretory pathway, component EscR	100	-	-
MCB1EB_194	escT	Type III secretory pathway, component EscS	100	-	-
MCB1EB_195	SsaU	Type III secretory pathway, component EscT	100	-	-
MCB1EB_196	GSI01S_33_00230	Type III secretory pathway, component EscU	100	-	-
MCB1EB_197	-	Transcriptional regulators of sugar metabolism	83	-	-
MCB1EB_198	ETSY1_29215	Transposase and inactivated derivatives	-	-	-
MCB1EB_199	-	Cation transport ATPase	-	-	-
MCB1EB_200	HMPREF1541_09163	Hypothetical protein	-	-	-
MCB1EB_201	PMI35_05042	Hypothetical protein	100	-	-
MCB1EB_202	BURK_027400	Transcriptional regulators	100	-	-
MCB1EB_203	K788_6823	Transcriptional regulator	100	-	-
MCB1EB_204	RSc3174	Hypothetical protein	98	44	91
MCB1EB_205	secA	Hypothetical protein	100	-	-
MCB1EB_206	argJ	Preprotein translocase subunit SecA (ATPase, RNA helicase)	100	78	100
MCB1EB_207	-	N-acetylglutamate synthase (N-acetylornithine aminotransferase)	100	71	100
MCB1EB_208	mutT	Predicted ATPase (AAA+ superfamily)	100	79	99
MCB1EB_209	yacG	NTP pyrophosphohydrolases including oxidative damage repair enzymes	100	74	91
MCB1EB_210	-	Uncharacterized protein conserved in bacteria	100	57	90
MCB1EB_211	coaE	Uncharacterized protein conserved in bacteria	100	76	99
MCB1EB_212	-	Dephospho-CoA kinase	100	58	98
MCB1EB_213	pilC	Type II secretory pathway, prepilin signal peptidase PulO and related peptidases	100	50	97
MCB1EB_214	-	Type II secretory pathway, component PulF	100	42	96
MCB1EB_215	-	Type II secretory pathway, ATPase PulE/Tfp pilus assembly pathway, ATPase PilB	100	58	87
MCB1EB_216	ispB	Putative Mg^{2+} and Co^{2+} transporter CorB	100	69	98
MCB1EB_217	-	Geranylgeranyl pyrophosphate synthase	100	75	100
MCB1EB_218	tufA	Predicted membrane protein	86	70	97
MCB1EB_219	secE	GTPases - translation elongation factors	100	89	100
MCB1EB_220	nusG	Preprotein translocase subunit SecE	87	63	100
MCB1EB_221	rplK	Transcription antiterminator	100	90	100
MCB1EB_222	rplA	Ribosomal protein L11	100	-	-
MCB1EB_223	rplJ	Ribosomal protein L1	100	85	100
MCB1EB_224	rplL	Ribosomal protein L10	100	77	93
MCB1EB_225	rpoB	Ribosomal protein L7/L12	100	87	100
MCB1EB_226	rpoC	DNA-directed RNA polymerase, beta subunit/140 kD subunit	100	-	-
MCB1EB_227	CAGGBEG34_190142	DNA-directed RNA polymerase, beta' subunit/160 kD subunit	100	89	100
MCB1EB_228	recQ	Hypothetical protein	-	33	-
MCB1EB_229	rpsL	Superfamily II DNA helicase	100	74	100
MCB1EB_230	rpsG	Ribosomal protein S12	100	-	-
MCB1EB_231	fusA1	Ribosomal protein S7	100	88	100
MCB1EB_232	rpsJ	Translation elongation factors (GTPases)	100	81-87	100
MCB1EB_233	rplC	Ribosomal protein S10	100	76	98
MCB1EB_234	rplD	Ribosomal protein L3	100	73	100
MCB1EB_235	rplW	Ribosomal protein L4	100	85	100
MCB1EB_236	rplB	Ribosomal protein L23	100	69	100
MCB1EB_237	rpsS	Ribosomal protein L2	100	85	100
MCB1EB_238	rplV	Ribosomal protein S19	100	86	100
MCB1EB_239	rpsC	Ribosomal protein L22	100	82	100
MCB1EB_240	rplP	Ribosomal protein S3	100	89	85
MCB1EB_241	rpmC	Ribosomal protein L16/L10E	99	95	100
MCB1EB_242	rpsQ	Ribosomal protein L29	100	75	100
MCB1EB_243	-	Ribosomal protein S17	100	83	100
MCB1EB_244	rplX	Ribosomal protein L14	100	89	81
MCB1EB_245	rplE	Ribosomal protein L24	99	75	100
MCB1EB_246	rpsN	Ribosomal protein L5	100	84	100
MCB1EB_247	rpsH	Ribosomal protein S14	100	89	83
MCB1EB_248	rplF	Ribosomal protein S8	100	82	100
MCB1EB_249	rplR	Ribosomal protein L6P/L9E	100	-	-
MCB1EB_250	rpsE	Ribosomal protein L18	100	83	100
MCB1EB_251	rpmD	Ribosomal protein S5	100	81	99
MCB1EB_252	rplO	Ribosomal protein L30/L7E	100	82	100
MCB1EB_253	secY	Ribosomal protein L15	100	77	99
MCB1EB_254	infA	Preprotein translocase subunit SecY	100	87	100
MCB1EB_255	rpsM	Translation initiation factor 1 (IF-1)	100	94	100
MCB1EB_256	rpsK	Ribosomal protein S13	100	74	100
MCB1EB_257	rpsD	Ribosomal protein S11	100	84	100
MCB1EB_258	rpoA	Ribosomal protein S4 and related proteins	100	84	100
MCB1EB_259	rplQ	DNA-directed RNA polymerase, alpha subunit/40 kD subunit	100	87	100
MCB1EB_260	BamMEX5DRAFT_2889	Ribosomal protein L17	100	78	93
MCB1EB_261	hemB	Thiol:disulfide interchange protein	100	43	100
MCB1EB_262	CAGGBEG34_190142	Delta-aminolevulinic acid dehydratase	100	77	99
MCB1EB_263	engB	Hypothetical protein	100	33	83
MCB1EB_264	-	Predicted GTPase	100	71	98
MCB1EB_265	DOK_03213	Cytochrome c553	100	55	86
MCB1EB_266	-	Hypothetical protein	100	-	-
MCB1EB_267	-	ResB protein required for cytochrome c biosynthesis	97	61	97
MCB1EB_268	NFKB1	ABC-type transport system involved in cytochrome c biogenesis, permease component	100	74	91
MCB1EB_269	lysA	FOG: Ankyrin repeat	100	-	-
MCB1EB_270	cyaY	Diaminopimelate decarboxylase	100	72	99
MCB1EB_271	-	Protein implicated in iron transport, frataxin homolog	100	56	94
MCB1EB_272	BYI23_A024070	Membrane carboxypeptidase/penicillin-binding protein	100	66-68	-
MCB1EB_273	CAGGBEG34_210075	Tfp pilus assembly protein, ATPase PilM	100	34	99
MCB1EB_274	-	Tfp pilus assembly protein PilP	100	-	-
MCB1EB_275	aroK	Type II secretory pathway, component HofQ	100	-	-
MCB1EB_276	aroB	Shikimate kinase	100	70	99
MCB1EB_277	dgt	3-dehydroquinate synthetase	100	69	97
MCB1EB_278	PMI06_006043	dGTP triphosphohydrolase	100	77	99
MCB1EB_279	-	Outer membrane protein W	100	46	100
MCB1EB_280	-	ABC-type transport system involved in resistance to organic solvents, ATPase component	100	77	96
MCB1EB_281	-	ABC-type transport system involved in resistance to organic solvents, permease component	100	84	100
MCB1EB_282	-	ABC-type transport system involved in resistance to organic solvents, periplasmic component	100	77	90
MCB1EB_283	BURK_006002	Surface lipoprotein	100	-	-

MCB1EB_284	-	ABC-type transport system involved in resistance to organic solvents, auxiliary component	100	56	94
MCB1EB_285	-	Predicted NTP binding protein (contains STAS domain)	100	47	99
MCB1EB_286	-	ABC-type multidrug transport system, ATPase component	67	75	99
MCB1EB_287	murA	ABC-type multidrug transport system, permease component	100	74	100
MCB1EB_288	hisG	UDP-N-acetylglucosamine enolpyruvyl transferase	100	74	99
MCB1EB_289	hisD	ATP phosphoribosyltransferase	100	82	96
MCB1EB_290	hisC	Histidinol dehydrogenase	100	75	100
MCB1EB_291	hisB	Histidinol-phosphate/aromatic aminotransferase and cobyric acid decarboxylase	100	69	99
MCB1EB_292	hisH	Imidazoleglycerol-phosphate dehydratase	100	64	97
MCB1EB_293	hisA	Glutamine amidotransferase	100	72	100
MCB1EB_294	hisF	Phosphoribosylformimino-5-aminoimidazole carboxamide ribonucleotide (ProFAR) isomerase	82	64	100
MCB1EB_295	hisI	Imidazoleglycerol-phosphate synthase	81	78	93
MCB1EB_296	hisE	Phosphoribosyl-AMP cyclohydrolase	100	71	94
MCB1EB_297	C266_22308	Phosphoribosyl-ATP pyrophosphohydrolase	100	-	-
MCB1EB_298	-	Predicted membrane protein	100	-	88
MCB1EB_299	tatA	Diadenosine tetraphosphate (Ap4A) hydrolase and other HIT family hydrolases	100	73	100
MCB1EB_300	tatB	Sec-independent protein secretion pathway components	100	58	98
MCB1EB_301	tatC	Sec-independent protein secretion pathway components	100	48	99
MCB1EB_302	-	Sec-independent protein secretion pathway component TatC	100	70	99
MCB1EB_303	-	Trypsin-like serine proteases, typically periplasmic, contain C-terminal PDZ domain	100	77	99
MCB1EB_304	-	Uncharacterized conserved protein	100	73	100
MCB1EB_305	petB	Rieske Fe-S protein	100	74	100
MCB1EB_306	-	Cytochrome b subunit of the bc complex	100	78	97
MCB1EB_307	sspA	Cytochrome c1	100	63	92
MCB1EB_308	-	Glutathione S-transferase	100	90	100
MCB1EB_309	SM8_04219	Stringent starvation protein B	100	-	-
MCB1EB_310	Dacsa_3325	FOG: TPR repeat	100	-	-
MCB1EB_311	LYNGBM3L_24300	Hypothetical protein	100	-	-
MCB1EB_312	CAGGBEG34_190029	Predicted NTPase (NACHT family)	66-91	-	-
MCB1EB_313	HNWD1	Hypothetical protein	-	-	-
MCB1EB_314	CAGGBEG34_330054	Predicted NTPase (NACHT family)	78-96	-	-
MCB1EB_315	RoseRS_4025	Transposase and inactivated derivatives	-	-	-
MCB1EB_316	-	Predicted NTPase (NACHT family)	66-91	-	-
MCB1EB_317	-	Transposase and inactivated derivatives	-	-	-
MCB1EB_318	istB	Transposase and inactivated derivatives	100	65	-
MCB1EB_319	istA	DNA replication protein	-	56	-
MCB1EB_320	-	Transposase and inactivated derivatives	-	-	-
MCB1EB_321	-	InterPro IPR001584 COGs COG2801	-	-	-
MCB1EB_322	istB	Transposase and inactivated derivatives	-	54-55	-
MCB1EB_323	BCh11DRAFT_03152	DNA replication protein	-	66	-
MCB1EB_324	-	NAD-dependent aldehyde dehydrogenases	-	-	-
MCB1EB_325	istA	Transposase and inactivated derivatives	-	62	-
MCB1EB_326	Npun_F1419	Transposase and inactivated derivatives	-	-	-
MCB1EB_327	HMPREF9711_02659	Predicted NTPase (NACHT family)	-	-	-
MCB1EB_328	RoseRS_4025	Orotidine-5'-phosphate decarboxylase	-	-	-
MCB1EB_329	CAGGBEG34_330054	Predicted NTPase (NACHT family)	66-91	-	-
MCB1EB_330	-	Hypothetical protein	-	-	-
MCB1EB_331	CARN7_2937	DNA replication protein	-	-	-
MCB1EB_332	-	Transposase and inactivated derivatives	-	-	-
MCB1EB_333	BN683_02092	Transposase and inactivated derivatives	-	-	-
MCB1EB_334	-	Hypothetical protein	-	-	-
MCB1EB_335	-	Transposase and inactivated derivatives	34-100	27	-
MCB1EB_336	-	Transposase and inactivated derivatives	91-100	-	-
MCB1EB_337	HMPREF1487_04394	Transposase and inactivated derivatives	100	-	-
MCB1EB_338	LHK_00908	Integrase	-	-	-
MCB1EB_339	BN508_00740	Integrase	-	-	-
MCB1EB_340	-	Transcriptional regulators	89	-	-
MCB1EB_341	ubiH	Predicted GTPase, probable translation factor	99	82	100
MCB1EB_342	-	2-polyprenyl-6-methoxyphenol hydroxylase and related FAD-dependent oxidoreductases	100	64	99
MCB1EB_343	SG1089	Predicted protease with the C-terminal PDZ domain	100	67	100
MCB1EB_344	ung	Hypothetical protein	100	38	-
MCB1EB_345	-	Uracil DNA glycosylase	100	-	-
MCB1EB_346	trpD	Indole-3-glycerol phosphate synthase	100	65	100
MCB1EB_347	trpG	Anthranilate phosphoribosyltransferase	100	75	100
MCB1EB_348	trpE	Anthranilate/para-aminobenzoate synthases component II	100	73	99
MCB1EB_349	rpe	Anthranilate/para-aminobenzoate synthases component I	100	79	100
MCB1EB_350	apaG	Pentose-5-phosphate-3-epimerase	100	82	98
MCB1EB_351	mltA	Uncharacterized protein affecting Mg^{2+}/Co^{2+} transport	100	77	100
MCB1EB_352	-	Membrane-bound lytic murein transglycosylase	85	-	93
MCB1EB_353	-	Hypothetical protein	100	-	-
MCB1EB_354	Bpet0683	Uncharacterized protein conserved in archaea	-	-	-
MCB1EB_355	BCh11DRAFT_04003	Hypothetical protein	-	-	-
MCB1EB_356	ubiB	Uncharacterized protein conserved in bacteria	80	-	94
MCB1EB_357	-	Predicted unusual protein kinase	100	76	100
MCB1EB_358	-	Uncharacterized protein conserved in bacteria	100	-	-
MCB1EB_359	aspS	Uncharacterized conserved protein	98	79	94
MCB1EB_360	ntpA	Aspartyl-tRNA synthetase	100	82	100
MCB1EB_361	-	NTP pyrophosphohydrolases including oxidative damage repair enzymes	100	70	94
MCB1EB_362	THITH_00215	Acyl-CoA dehydrogenases	100	81	100
MCB1EB_363	fadB	Hypothetical protein	-	-	-
MCB1EB_364	fadA	3-hydroxyacyl-CoA dehydrogenase	100	-	-
MCB1EB_365	-	Acetyl-CoA acetyltransferase	100	78	100
MCB1EB_366	gdhA	Acyl-CoA hydrolase	100	73	98
MCB1EB_367	-	Glutamate dehydrogenase/leucine dehydrogenase	100	85	99
MCB1EB_368	gltJ	ABC-type amino acid transport/signal transduction systems, periplasmic component/domain	100	72	92
MCB1EB_369	-	ABC-type amino acid transport system, permease component	100	76	100
MCB1EB_370	-	ABC-type amino acid transport system, permease component	100	74	100
MCB1EB_371	argD	ABC-type polar amino acid transport system, ATPase component	100	87	100
MCB1EB_372	ascD	Ornithine/acetylornithine aminotransferase	100	74	100
MCB1EB_373	BCh11DRAFT_05379	2-polyprenylphenol hydroxylase and related flavodoxin oxidoreductases	100	73	100
MCB1EB_374	-	Nucleoside-diphosphate-sugar epimerases	100	58	61
MCB1EB_375	-	Glutaredoxin-related protein	100	76	100
MCB1EB_376	-	3-polyprenyl-4-hydroxybenzoate decarboxylase	100	66	92
MCB1EB_377	RSK60_1520072	Amino acid transporters	100	-	-
MCB1EB_378	BRADI1G69720	SEC7 domain proteins	100	-	-
MCB1EB_379	MC7420_7550	DNA polymerase III, alpha subunit	-	-	-
MCB1EB_380	CAGGBEG34_330054	Predicted NTPase (NACHT family)	-	-	-
MCB1EB_381	Mic7113_0456	Methyl-accepting chemotaxis protein	-	-	-

MCB1EB_382	matK	Predicted NTPase (NACHT family)	66-91	-	-
MCB1EB_383	HNWD1	Retron-type reverse transcriptase	-	-	-
MCB1EB_384	Npun_F1419	Predicted NTPase (NACHT family)	66-91	-	-
MCB1EB_385	wzxE	Predicted NTPase (NACHT family)	66-91	-	-
MCB1EB_386	Thi970DRAFT_00599	Membrane protein involved in the export of O-antigen and teichoic acid	94	-	-
MCB1EB_387	-	Thiol-disulfide isomerase and thioredoxins	-	-	-
MCB1EB_388	Cyan7822_2631	Transposase and inactivated derivatives	-	-	-
MCB1EB_389	Cyan7822_2631	Predicted NTPase (NACHT family)	78-96	-	-
MCB1EB_390	CAGGBEG34_330054	Predicted NTPase (NACHT family)	78-96	-	-
MCB1EB_391	CAGGBEG34_220024	Hypothetical protein	-	-	-
MCB1EB_392	-	Phosphotransferase system IIC components, glucose/maltose/N-acetylglucosamine-specific	-	54	-
MCB1EB_393	-	Transposase and inactivated derivatives	100	-	-
MCB1EB_394	CARN7_2937	Transposase and inactivated derivatives	100	65	-
MCB1EB_395	-	Transposase and inactivated derivatives	-	-	-
MCB1EB_396	CAGGBEG34_420004	DNA replication protein	-	-	-
MCB1EB_397	Neut_1498	Hypothetical protein	97	-	-
MCB1EB_398	ANA_C12029	Integrase	100	25	97
MCB1EB_399	CAGGBEG34_330054	Predicted NTPase (NACHT family)	-	-	-
MCB1EB_400	ACD_44C00098G0001	Hypothetical protein	-	-	-
MCB1EB_401	CAGGBEG34_330054	Predicted NTPase (NACHT family)	-	-	-
MCB1EB_402	CAGGBEG34_220024	Hypothetical protein	-	-	-
MCB1EB_403	-	Permeases of the major facilitator superfamily	-	54	-
MCB1EB_404	SFV_0255	Hypothetical protein	100	72	-
MCB1EB_405	L682_09965	Hypothetical protein	-	-	-
MCB1EB_406	L682_09960	Hypothetical protein	100	-	-
MCB1EB_407	L682_09955	Hypothetical protein	100	-	96
MCB1EB_408	L682_09950	Uncharacterized homolog of phage Mu protein gp47	-	26	-
MCB1EB_409	L682_09945	Hypothetical protein	-	-	-
MCB1EB_410	HIB_15810	Hypothetical protein	-	28	-
MCB1EB_411	-	Hypothetical protein	100	25	-
MCB1EB_412		Uncharacterized protein conserved in bacteria	-	-	-
MCB1EB_413	L682_09935	Predicted transcriptional regulator	-	42-48	-
MCB1EB_414	HMPREF9095_1683	Hypothetical protein	-	-	-
MCB1EB_415	BC1002_6556	Hypothetical protein	-	-	-
MCB1EB_416	-	Hypothetical protein	-	22	-
MCB1EB_417	L682_09915	Hypothetical protein	-	-	-
MCB1EB_418	B398_05775	Hypothetical protein	-	-	-
MCB1EB_419	L682_09905	Hypothetical protein	-	-	-
MCB1EB_420	BC1002_6552	Hypothetical protein	100	21	-
MCB1EB_421	PD_0974	Hypothetical protein	-	-	-
MCB1EB_422	gene0107	Hypothetical protein	-	-	-
MCB1EB_423	BC1002_6549	Hypothetical protein	-	-	-
MCB1EB_424	BC1002_6547	Hypothetical protein	-	-	-
MCB1EB_425	XF_1576	Uncharacterized protein conserved in bacteria	100	-	-
MCB1EB_426	BC1002_6545	Hypothetical protein	96	-	-
MCB1EB_427	XFEB_01759	Uncharacterized protein conserved in bacteria	100	-	-
MCB1EB_428	BC1002_6543	Uncharacterized protein, homolog of phage Mu protein gp30	100	30	-
MCB1EB_429	-	Uncharacterized protein conserved in bacteria	100	-	-
MCB1EB_430	PA2G_00805	Uncharacterized protein conserved in bacteria	96-100	32	-
MCB1EB_431	HMPREF0005_02877	Glycosyltransferase	-	-	-
MCB1EB_432	CAGGBEG34_330022	Hypothetical protein	97	-	-
MCB1EB_433	CAGGBEG34_330013	Pyruvate-formate lyase-activating enzyme	-	-	-
MCB1EB_434	C75L2_00370035	Acyl-CoA dehydrogenases	-	-	-
MCB1EB_435	Shewmr7_3068	Hypothetical protein	-	-	-
MCB1EB_436	HMPREF9701_05463	FOG: Ankyrin repeat	-	-	-
MCB1EB_437	Alide_1665	Hypothetical protein	-	-	-
MCB1EB_438	O991_02545	ABC-type multidrug transport system, permease component	-	-	-
MCB1EB_439	NH8B_1955	Phosphatidylinositol kinase and protein kinases of the PI-3 kinase family	-	-	-
MCB1EB_440	Halhy_5407	Predicted transcriptional regulator	-	-	-
MCB1EB_441	Veis_0670	Predicted membrane protein	-	-	-
MCB1EB_442	D623_10006357	Hypothetical protein	-	-	-
MCB1EB_443	ST1_0045	cAMP-binding proteins - catabolite gene activator and regulatory subunit of cAMP-dependent protein kinases	100	-	-
MCB1EB_444	CAGGBEG34_190021	Hypothetical protein	100	-	-
MCB1EB_445	-	Phage-related protein, predicted endonuclease	-	-	-
MCB1EB_446	BDB_30064	Recombinational DNA repair protein (RecE pathway)	-	-	-
MCB1EB_447	CAGGBEG34_610004	ATPase involved in DNA repair	-	-	-
MCB1EB_448	RBRH_00570	Methyl-accepting chemotaxis protein	-	42	-
MCB1EB_449	PGSC0003DMG40001771	Na+/citrate symporter	99-100	49-53	-
MCB1EB_450	B738_22335	FKBP-type peptidyl-prolyl cis-trans isomerases 2	100	-	-
MCB1EB_451	CAGGBEG34_420004	Hypothetical protein	100	-	-
MCB1EB_452	-	Hypothetical protein	-	-	-
MCB1EB_453	-	Integrase	-	30	-
MCB1EB_454	-	Ferredoxin	100	85	100
MCB1EB_455	-	Na+/H+ antiporter NhaD and related arsenite permeases	100	66	98
MCB1EB_456	-	Uncharacterized protein conserved in bacteria	97	72	93
MCB1EB_457	-	Hypothetical protein	100	64	100
MCB1EB_458	-	Co/Zn/Cd efflux system component	100	-	-
MCB1EB_459	prfA	Methylase of polypeptide chain release factors	100	59	100
MCB1EB_460	-	Protein chain release factor A	100	82	99
MCB1EB_461	bcp	Predicted ATPase related to phosphate starvation-inducible protein PhoH	100	76	100
MCB1EB_462	-	Peroxiredoxin	100	67	98
MCB1EB_463	-	Predicted xylanase/chitin deacetylase	100	75	100
MCB1EB_464	pmrIa	Nucleoside-diphosphate-sugar epimerases	100	84	99
MCB1EB_465	pmrF	Methionyl-tRNA formyltransferase	100	70	100
MCB1EB_466	pmrH	Glycosyltransferases involved in cell wall biogenesis	100	80	96
MCB1EB_467	-	Predicted pyridoxal phosphate-dependent enzyme apparently involved in regulation of cell wall biogenesis	100	74	98
MCB1EB_468	LOTGIDRAFT_118875	Permeases of the drug/metabolite transporter (DMT) superfamily	100	77	99
MCB1EB_469	ROI_15060	Hypothetical protein	99	-	-
MCB1EB_470	ATXN3	Phosphatidylserine/phosphatidylglycerophosphate/cardiolipin synthases and related enzymes	93	-	-
MCB1EB_471	arnT	Spermidine/putrescine-binding periplasmic protein	100	-	-
MCB1EB_472	-	4-amino-4-deoxy-L-arabinose transferase and related glycosyltransferases of PMT family	100	64	95
MCB1EB_473	-	Uncharacterized conserved protein	-	-	-
MCB1EB_474	pyrF	Aspartate/tyrosine/aromatic aminotransferase	100	75	99
MCB1EB_475	-	Orotidine-5'-phosphate decarboxylase	99	76	98
MCB1EB_476	C265_18829	Na+/citrate symporter	88-99	54-56	-
MCB1EB_477	BN113_0351	Transcriptional regulator	100	31	97
MCB1EB_478	-	Transcriptional regulator	100	27	-
MCB1EB_479	-	Permeases of the major facilitator superfamily	100	-	-
MCB1EB_480	parE	ATPase components of ABC transporters with duplicated ATPase domains	100	71	100

MCB1EB_481	LACBIDRAFT_297160	Type IIA topoisomerase (DNA gyrase/topo II, topoisomerase IV), B subunit	100	83	100
MCB1EB_482	IGC_05496	Serine phosphatase RsbU, regulator of sigma subunit	-	-	-
MCB1EB_483	parC	Hypothetical protein	100	-	-
MCB1EB_484	FMAG_00195	Type IIA topoisomerase (DNA gyrase/topo II, topoisomerase IV), A subunit	-	-	-
MCB1EB_485	parC	ATPase related to the helicase subunit of the Holliday junction resolvase	-	-	-
MCB1EB_486	aspC	Type IIA topoisomerase (DNA gyrase/topo II, topoisomerase IV), A subunit	100	77	100
MCB1EB_487	-	Aspartate/tyrosine/aromatic aminotransferase	100	75	99
MCB1EB_488	tnpA	Helicase subunit of the DNA excision repair complex	100	83	98
MCB1EB_489	kdsB	Transposase and inactivated derivatives	96	35-36	-
MCB1EB_490	HMPREF9710_00354	CMP-2-keto-3-deoxyoctulosonic acid synthetase	-	-	-
MCB1EB_491	RBRH_00986	Predicted sugar phosphate isomerase involved in capsule formation	-	-	-
MCB1EB_492	greB	Permeases of the drug/metabolite transporter (DMT) superfamily	-	59	-
MCB1EB_493	spoT	Transcription elongation factor	100	64	98
MCB1EB_494	rpoZ	Guanosine polyphosphate pyrophosphohydrolases/synthetases	100	77	99
MCB1EB_495	gmk	DNA-directed RNA polymerase, subunit K/omega	100	64	100
MCB1EB_496	-	Guanylate kinase	100	72	90
MCB1EB_497	rph	Uncharacterized stress-induced protein	100	-	-
MCB1EB_498	-	RNase PH	100	71	98
MCB1EB_499	hemN	Xanthosine triphosphate pyrophosphatase	100	65	90
MCB1EB_500	-	Coproporphyrinogen III oxidase and related Fe-S oxidoreductases	100	71	97
MCB1EB_501	Piso0_004142	Integrase	-	-	-
MCB1EB_502	Bm1_43535	ABC-type sugar transport system, periplasmic component	100	-	-
MCB1EB_503	talA	Hypothetical protein	-	-	-
MCB1EB_504	-	Transaldolase	100	68	100
MCB1EB_505	surE	Protein involved in catabolism of external DNA	100	63	99
MCB1EB_506	-	Predicted acid phosphatase	100	75	100
MCB1EB_507	-	Protein-L-isoaspartate carboxylmethyltransferase	100	62	99
MCB1EB_508	-	Membrane proteins related to metalloendopeptidases	85	65	97
MCB1EB_509	-	DNA-directed RNA polymerase, sigma subunit (sigma70/sigma32)	-	-	-
MCB1EB_510	rpoS	Transposase and inactivated derivatives, IS5 family	-	82	-
MCB1EB_511	-	DNA-directed RNA polymerase, sigma subunit (sigma70/sigma32)	-	-	-
MCB1EB_512	TRIATDRAFT_285415	Predicted 3'-5' exonuclease related to the exonuclease domain of PolB	100	74	100
MCB1EB_513	groES	Integrase	-	-	-
MCB1EB_514	groEL	Co-chaperonin GroES (HSP10)	100	85	96
MCB1EB_515	-	Chaperonin GroEL (HSP60 family)	100	87	96
MCB1EB_516	-	Rubredoxin	100	93	100
MCB1EB_517	yqqF	Putative transcriptional regulator	100	77	87
MCB1EB_518	-	Predicted endonuclease involved in recombination (possible Holliday junction resolvase in Mycoplasmas and B. subtilis)	100	65	93
MCB1EB_519	pyrB	Pyrimidine operon attenuation protein/uracil phosphoribosyltransferase	100	69	93
MCB1EB_520	pyrC	Aspartate carbamoyltransferase, catalytic chain	100	83	98
MCB1EB_521	K788_7818	Dihydroorotase and related cyclic amidohydrolases	100	73	100
MCB1EB_522	apaH	1-acyl-sn-glycerol-3-phosphate acyltransferase	100	56	93
MCB1EB_523	SRU_1425	Diadenosine tetraphosphatase and related serine/threonine protein phosphatases	-	-	-
MCB1EB_524	-	Hydrogenase maturation factor	-	-	-
MCB1EB_525	polC	Hypothetical protein	-	-	-
MCB1EB_526	-	Hypothetical protein	-	-	-
MCB1EB_527	BCE_G9241_2121	Transposase and inactivated derivatives, IS5 family	-	-	-
MCB1EB_528	CLOHYLEM_04803	ABC-type nitrate/sulfonate/bicarbonate transport systems, periplasmic components	-	-	-
MCB1EB_529	secA	Hypothetical protein	-	-	-
MCB1EB_530	Anacy_0920	Hypothetical protein	-	-	-
MCB1EB_531	LDG_7312	FOG: Ankyrin repeat	-	-	-
MCB1EB_532	rfbB	Preprotein translocase subunit SecA (ATPase, RNA helicase)	-	-	-
MCB1EB_533	-	dTDP-D-glucose 4,6-dehydratase	-	-	-
MCB1EB_534	rfbC	dTDP-glucose pyrophosphorylase	100	-	-
MCB1EB_535	CAGGBEG34_200100	dTDP-4-dehydrorhamnose 3,5-epimerase and related enzymes	-	-	-
MCB1EB_536	-	Uncharacterized protein conserved in bacteria	100	47	78
MCB1EB_537	A1I_06320	Uncharacterized protein SCO1/SenC/PrrC, involved in biogenesis of respiratory and photosynthetic systems	100	54	100
MCB1EB_538	-	Arylsulfatase A and related enzymes	100	-	-
MCB1EB_539	-	ABC-type multidrug transport system, ATPase and permease components	100	74	81
MCB1EB_540	-	Glycosyltransferase	100	79	100
MCB1EB_541	-	Uncharacterized protein probably involved in high-affinity Fe2+ transport	100	72	96
MCB1EB_542	-	Hypothetical protein	100	-	-
MCB1EB_543	-	High-affinity Fe2+/Pb2+ permease	100	66	99
MCB1EB_544	-	Polyferredoxin	100	69	98
MCB1EB_545	-	Membrane-fusion protein	100	64	90
MCB1EB_546	-	Cation/multidrug efflux pump	100	74	99
MCB1EB_547	MARPU_13085	Outer membrane protein	100	64	99
MCB1EB_548	Agau_P200259	Uncharacterized protein related to capsule biosynthesis enzymes	100	-	-
MCB1EB_549	-	Predicted transcriptional regulators	100	-	-
MCB1EB_550	-	ABC-type Mn^{2+}/Zn^{2+} transport systems, permease components	100	77	100
MCB1EB_551	-	ABC-type Mn/Zn transport systems, ATPase component	100	69	95
MCB1EB_552	Bxe_A3912	ABC-type metal ion transport system, periplasmic component/surface adhesin	100	61	84
MCB1EB_553	CAGGBEG34_200018	Tfp pilus assembly protein FimT	100	-	-
MCB1EB_554	CAGGBEG34_200017	Tfp pilus assembly protein PilV	100	-	-
MCB1EB_555	CAGGBEG34_200016	Tfp pilus assembly protein PilW	100	36	90
MCB1EB_556	CAGGBEG34_200015	Tfp pilus assembly protein PilX	100	-	-
MCB1EB_557	Bamb_0690	Tfp pilus assembly protein PilE	100	-	-
MCB1EB_558	Bcep1808_0746	Methyl-accepting chemotaxis protein	100	-	-
MCB1EB_559	-	Predicted membrane protein	100	52	99
MCB1EB_560	abcT4	Uncharacterized conserved protein	100	75	100
MCB1EB_561	msrA	ABC-type multidrug transport system, ATPase and permease components	100	-	-
MCB1EB_562	Halhy_2265	Peptide methionine sulfoxide reductase	100	62	97
MCB1EB_563	Cyan7822_4279	Predicted NTPase (NACHT family)	78-96	-	-
MCB1EB_564	pdxH	FOG: HEAT repeat	-	-	-
MCB1EB_565	glnS	Pyridoxamine-phosphate oxidase	100	64	99
MCB1EB_566	HA1_08492	Glutamyl- and glutaminyl-tRNA synthetases	100	78	99
MCB1EB_567	-	Predicted NTPase (NACHT family)	100	-	-
MCB1EB_568	kdsC	Predicted sugar phosphate isomerase involved in capsule formation	100	71	100
MCB1EB_569	MYA_2549	Low specificity phosphatase (HAD superfamily)	100	61	92
MCB1EB_570	Bphyt_0598	Uncharacterized protein conserved in bacteria	100	59	90
MCB1EB_571	-	Uncharacterized protein conserved in bacteria	100	48	88
MCB1EB_572	-	ABC-type (unclassified) transport system, ATPase component	100	80	100
MCB1EB_573	ptsN	Ribosome-associated protein Y (PSrp-1)	100	-	-
MCB1EB_574	hprK	Phosphotransferase system mannitol/fructose-specific IIA domain (Ntr-type)	100	77	96
MCB1EB_575	-	Serine kinase of the HPr protein, regulates carbohydrate metabolism	100	86	98
MCB1EB_576	Aasi_1766	Predicted P-loop-containing kinase	100	70	99
MCB1EB_577	O166_23020	Predicted alternative thymidylate synthase	100	-	-

MCB1EB_578	RBRH_00033	Hypothetical protein	-	-	-
MCB1EB_579	RBRH_00029	Glycerophosphoryl diester phosphodiesterase	-	-	-
MCB1EB_580	A33K_15832	Hypothetical protein	100	70	78
MCB1EB_581	A33K_15831	Hypothetical protein	100	-	-
MCB1EB_582	CV_2589	Hypothetical protein	100	-	-
MCB1EB_583	ssaJ	Hypothetical protein	100	-	-
MCB1EB_584	BTI_1930	Type III secretory pathway, lipoprotein EscJ	100	-	-
MCB1EB_585	CV_2586	Hypothetical protein	100	-	-
MCB1EB_586	eprI	Hypothetical protein	100	-	-
MCB1EB_587	A33K_15825	Hypothetical protein	100	-	-
MCB1EB_588	CV_2581	AraC-type DNA-binding domain-containing proteins	94	-	-
MCB1EB_589	A33K_15824	Acyl-CoA dehydrogenases	100	-	-
MCB1EB_590	BTI_1937	Hypothetical protein	99	-	-
MCB1EB_591	A33K_15820	FOG: TPR repeat	100	-	-
MCB1EB_592	BTI_1939	Uncharacterized conserved protein	100	-	-
MCB1EB_593	BTI_1940	Cobalamin biosynthesis protein CobN and related Mg-chelatases	100	-	-
MCB1EB_594	BTI_1907	Hypothetical protein	92	-	-
MCB1EB_595	escU	Soluble lytic murein transglycosylase and related regulatory proteins (some contain LysM/invasin domains)	90	-	-
MCB1EB_596	escT	Type III secretory pathway, component EscU	99	-	-
MCB1EB_597	ssaS	Type III secretory pathway, component EscT	100	-	-
MCB1EB_598	epaP	Type III secretory pathway, component EscS	100	-	-
MCB1EB_599	PTE_02619	Type III secretory pathway, component EscR	100	-	-
MCB1EB_600	epaO	Uncharacterized ABC-type transport system, periplasmic component/surface lipoprotein	-	-	-
MCB1EB_601	CV_2605	Flagellar motor switch/type III secretory pathway protein	100	-	-
MCB1EB_602	CV_2604	3-phosphoglycerate kinase	100	-	-
MCB1EB_603	-	Flagellar hook protein FlgE	100	-	-
MCB1EB_604	escV	Flagellar biosynthesis/type III secretory pathway ATPase	100	-	-
MCB1EB_605	ssaM	Type III secretory pathway, component EscV	98-100	-	-
MCB1EB_606	BTI_1918	Hypothetical protein	100	-	-
MCB1EB_607	-	Signal transduction histidine kinase	98	-	-
MCB1EB_608	CV_2598	Response regulator containing a CheY-like receiver domain and an HTH DNA-binding domain	100	28	96
MCB1EB_609	escC	ABC-type sugar transport system, ATPase component	-	-	-
MCB1EB_610	CV_2596	Type II secretory pathway, component PulD	100	-	-
MCB1EB_611	CV_2595	Hypothetical protein	100	-	-
MCB1EB_612	-	Hexokinase	100	-	-
MCB1EB_613	-	Cytochrome c553	100	58	100
MCB1EB_614	hemA	Cytochrome c553	100	64	84
MCB1EB_615	-	Glutamyl-tRNA reductase	100	73	98
MCB1EB_616	CAGGBEG34_380006	Xaa-Pro aminopeptidase	100	65	98
MCB1EB_617	CAGGBEG34_380007	Hypothetical protein	100	-	
MCB1EB_618	HDN1F_23160	Hypothetical protein	99	-	
MCB1EB_619	M622_15490	Predicted hydrolases or acyltransferases (alpha/beta hydrolase superfamily)	84	-	
MCB1EB_620	MIM_c07780	Predicted hydrolase (metallo-beta-lactamase superfamily)	28	-	
MCB1EB_621	-	Hypothetical protein	100	-	
MCB1EB_622	CAGGBEG34_240007	Transposase and inactivated derivatives	100	-	
MCB1EB_623	-	Response regulator containing a CheY-like receiver domain and an HTH DNA-binding domain	100	55	77
MCB1EB_624	-	Hypothetical protein	100	64	100
MCB1EB_625	rluC	Ribonucleases G and E	100	76	
MCB1EB_626	SG1089	Pseudouridylate synthases, 23S RNA-specific	100	64	
MCB1EB_627	-	Hypothetical protein	100	-	99
MCB1EB_628	-	Periplasmic serine proteases (ClpP class)	100	59	95
MCB1EB_629	-	Predicted methyltransferases	100	68	99
MCB1EB_630	A33K_14804	Nucleotide-binding protein implicated in inhibition of septum formation	100	-	
MCB1EB_631	RoseRS_4025	Predicted metal-binding, possibly nucleic acid-binding protein	100	-	
MCB1EB_632	PVAR5_8935	Predicted NTPase (NACHT family)	78-96	-	
MCB1EB_633	rpmF	Hypothetical protein	-	-	-
MCB1EB_634	plsX	Ribosomal protein L32	100	78	100
MCB1EB_635	fabH1	Fatty acid/phospholipid biosynthesis enzyme	100	77	87
MCB1EB_636	fabD	3-oxoacyl-[acyl-carrier-protein] synthase III	100	75	100
MCB1EB_637	fabG	(acyl-carrier-protein) S-malonyltransferase	100	72	100
MCB1EB_638	acpP	Dehydrogenases with different specificities (related to short-chain alcohol dehydrogenases)	100	74	100
MCB1EB_639	fabF1	Acyl carrier protein	100	-	
MCB1EB_640	BCh11DRAFT_05733	3-oxoacyl-(acyl-carrier-protein) synthase	100	79	100
MCB1EB_641	rpoE1	Uncharacterized protein conserved in bacteria	100	-	
MCB1EB_642	-	DNA-directed RNA polymerase specialized sigma subunit, sigma24 homolog	100	75	100
MCB1EB_643	Cal6303_2739	Trypsin-like serine proteases, typically periplasmic, contain C-terminal PDZ domain	100	65	91
MCB1EB_644	-	Predicted NTPase (NACHT family)	66-91	-	
MCB1EB_645	pagP	Transposase and inactivated derivatives	100	65	
MCB1EB_646	Cenrod_0254	Hypothetical protein	100	-	
MCB1EB_647	HNWD1	Predicted NTPase (NACHT family)	99-100	-	
MCB1EB_648	lepA	Predicted NTPase (NACHT family)	66-91	-	
MCB1EB_649	spaS	Membrane GTPase LepA	100	87	96
MCB1EB_650	rnc	Signal peptidase I	100	66	100
MCB1EB_651	era	dsRNA-specific ribonuclease	91	-	
MCB1EB_652	recO	GTPase	100	71	99
MCB1EB_653	pdxJ	Recombinational DNA repair protein (RecF pathway)	100	69	90
MCB1EB_654	acpS	Pyridoxal phosphate biosynthesis protein	100	72	95
MCB1EB_655	nagZ	Phosphopantetheinyl transferase (holo-ACP synthase)	100	-	
MCB1EB_656	efp	Beta-glucosidase-related glycosidases	96	64	95
MCB1EB_657	Bxe_A1094	Translation elongation factor P (EF-P)/translation initiation factor 5A (eIF-5A)	100	87	100
MCB1EB_658	B1144G04.29	Uncharacterized protein conserved in bacteria	100	53	99
MCB1EB_659	TRIADDRAFT_62950	Biotin-(acetyl-CoA carboxylase) ligase	-	-	
MCB1EB_660	uvrC	Glutamate synthase domain 2	-	-	
MCB1EB_661	pgsA	Nuclease subunit of the excinuclease complex	100	75	96
MCB1EB_662	-	Phosphatidylglycerophosphate synthase	100	78	96
MCB1EB_663	CAGGBEG34_320030	ATPase components of ABC transporters with duplicated ATPase domains	100	80	100
MCB1EB_664	Cal6303_2739	Transposase and inactivated derivatives	-	-	-
MCB1EB_665	Dmoj\GI11313	Predicted NTPase (NACHT family)	66-91	-	
MCB1EB_666	RoseRS_4025	DNA-directed RNA polymerase, beta' subunit/160 kD subunit	-	-	
MCB1EB_667	LYNGBM3L_24300	Predicted NTPase (NACHT family)	66-91	-	
MCB1EB_668	rpsF	Predicted NTPase (NACHT family)	-	-	
MCB1EB_669	-	Ribosomal protein S6	100	86	83
MCB1EB_670	rpsR	Primosomal replication protein N	100	61	98
MCB1EB_671	rplI	Ribosomal protein S18	100	82	100
MCB1EB_672	dnaB	Ribosomal protein L9	100	79	96
MCB1EB_673	-	Replicative DNA helicase	100	85	99

MCB1EB_674	-	Phosphate transport regulator (distant homolog of PhoU)	100	76	100
MCB1EB_675	minE	Phosphate/sulphate permeases	100	84	94
MCB1EB_676	minD	Septum formation topological specificity factor	100	77	99
MCB1EB_677	minC	Septum formation inhibitor-activating ATPase	100	86	100
MCB1EB_678	Vapar_5848	Septum formation inhibitor	100	56	100
MCB1EB_679	BurJV3_3298	Hypothetical protein	-	-	-
MCB1EB_680	-	Predicted transcriptional regulators	-	-	
MCB1EB_681	-	Biopolymer transport protein	98	57	99
MCB1EB_682	RBRH_03933	Biopolymer transport proteins	100	65	97
MCB1EB_683	BURK_023930	Periplasmic protein TonB, links inner and outer membranes	100	-	
MCB1EB_684	-	Bacterioferritin-associated ferredoxin	-	48	
MCB1EB_685	-	Glutamate racemase	-	-	
MCB1EB_686	-	Bacterioferritin (cytochrome b1)	100	81	98
MCB1EB_687	-	Predicted membrane protein	100	75	91
MCB1EB_688	MC7420_7550	Integrase	-	-	
MCB1EB_689	-	Predicted NTPase (NACHT family)	-	-	
MCB1EB_690	LYNGBM3L_24300	Transposase and inactivated derivatives	-	-	-
MCB1EB_691	OR16_03807	Predicted NTPase (NACHT family)	66-91	-	
MCB1EB_692	L810_8411	Spermidine/putrescine-binding periplasmic protein	100	-	
MCB1EB_693	-	Transcriptional regulator	100	33	87
MCB1EB_694	fbp1	Cu/Zn superoxide dismutase	100	-	
MCB1EB_695	-	Fructose-1,6-bisphosphatase	100	80	99
MCB1EB_696	-	ABC-type spermidine/putrescine transport system, permease component I	100	-	
MCB1EB_697	PMI24_02397	ABC-type spermidine/putrescine transport systems, ATPase components	100	-	
MCB1EB_698	PMO01_28020	Hypothetical protein	100	-	
MCB1EB_699	CARN4_0352	Hypothetical protein	100	-	
MCB1EB_700	WALBB_1140015	Hypothetical protein	100	-	
MCB1EB_701	-	FOG: Ankyrin repeat	100	-	
MCB1EB_702	-	Putative translation initiation inhibitor, yjgF family	100	72	99
MCB1EB_703	htpG	Permeases of the drug/metabolite transporter (DMT) superfamily	100	-	
MCB1EB_704	ubiC	Molecular chaperone, HSP90 family	100	79	99
MCB1EB_705	-	4-hydroxybenzoate synthetase (chorismate lyase)	100	49	92
MCB1EB_706	-	G:T/U mismatch-specific DNA glycosylase	100	71	97
MCB1EB_707	-	Spermidine synthase	100	66	95
MCB1EB_708	Cenrod_0254	Transposase and inactivated derivatives	-	-	-
MCB1EB_709	-	Predicted NTPase (NACHT family)	66-91	-	
MCB1EB_710	CAGGBEG34_330054	Transposase and inactivated derivatives	-	-	-
MCB1EB_711	SES60163_16622	Hypothetical protein	-	-	-
MCB1EB_712	PSPTOT1_4093	Transcriptional regulator, contains sigma factor-related N-terminal domain	100	-	
MCB1EB_713	SES60163_16627	NAD-dependent aldehyde dehydrogenases	-	-	
MCB1EB_714	SM39_2078	Large exoproteins involved in heme utilization or adhesion	-	-	
MCB1EB_715	RSPO_m00925	Hypothetical protein	100	-	
MCB1EB_716	MPL1_08392	Large exoproteins involved in heme utilization or adhesion	-	-	
MCB1EB_717	mutY	Large exoproteins involved in heme utilization or adhesion	-	-	
MCB1EB_718	mutM	A/G-specific DNA glycosylase	100	59	90
MCB1EB_719	-	Formamidopyrimidine-DNA glycosylase	100	70	100
MCB1EB_720	lolB	FOG: TPR repeat	100	57	88
MCB1EB_721	ipk	Outer membrane lipoprotein involved in outer membrane biogenesis	100	-	
MCB1EB_722	prsA	4-diphosphocytidyl-2C-methyl-D-erythritol 2-phosphate synthase	100	67	82
MCB1EB_723	rplY	Phosphoribosylpyrophosphate synthetase	100	89	99
MCB1EB_724	pth1	Ribosomal protein L25 (general stress protein Ctc)	100	73	98
MCB1EB_725	-	Peptidyl-tRNA hydrolase	100	69	98
MCB1EB_726	RBRH_01242	Hypothetical protein	100	-	
MCB1EB_727	-	Outer membrane protein (porin)	100	-	100
MCB1EB_728	ndk	Integral membrane protein, interacts with FtsH	100	-	99
MCB1EB_729	-	Nucleoside diphosphate kinase	100	-	100
MCB1EB_730	CAGGBEG34_80006	Predicted Fe-S-cluster redox enzyme	100	-	96
MCB1EB_731	BH160DRAFT_6135	Tfp pilus assembly protein PilF	100	-	
MCB1EB_732	ispG	Uncharacterized protein conserved in bacteria	100	37	89
MCB1EB_733	hisS	Enzyme involved in the deoxyxylulose pathway of isoprenoid biosynthesis	100	74	96
MCB1EB_734	BH160DRAFT_6132	Histidyl-tRNA synthetase	100	76	100
MCB1EB_735	-	Uncharacterized protein conserved in bacteria	100	56	100
MCB1EB_736	engA	FOG: WD40-like repeat	90	61	100
MCB1EB_737	hfq	Predicted GTPases	100	80	100
MCB1EB_738	BC1003_1211	Uncharacterized host factor I protein	100	91	100
MCB1EB_739	CARN2_3473	Membrane protease subunits, stomatin/prohibitin homologs	100	45	91
MCB1EB_740	-	Membrane protease subunits, stomatin/prohibitin homologs	100	43	87
MCB1EB_741	purA	ATP phosphoribosyltransferase involved in histidine biosynthesis	100	72	100
MCB1EB_742	kup2	Adenylosuccinate synthase	100	77	100
MCB1EB_743	-	K+ transporter	100	76	98
MCB1EB_744	-	Transcriptional accessory protein	100	79	95
MCB1EB_745	BCh11DRAFT_06143	Hypothetical protein	100	-	-
MCB1EB_746	dinG	Hypothetical protein	100	-	-
MCB1EB_747	C438_14856	Rad3-related DNA helicases	98	62	93
MCB1EB_748	BgramDRAFT_0582	DNA-binding HTH domain-containing proteins	100	-	-
MCB1EB_749	CAPGI0001_1457	AraC-type DNA-binding domain-containing proteins	100	43	100
MCB1EB_750	CC1_07920	Microtubule-binding protein involved in cell cycle control	100	-	-
MCB1EB_751	BgramDRAFT_0573	Hypothetical protein	100	-	-
MCB1EB_752	BMAJHU_I0812	Response regulators consisting of a CheY-like receiver domain and a winged-helix DNA-binding domain	100	46	96
MCB1EB_753	BMAA1618	Acetyltransferases, including N-acetylases of ribosomal proteins	100	-	-
MCB1EB_754	HMPREF1195_01277	Acetyltransferases, including N-acetylases of ribosomal proteins	100	-	-
MCB1EB_755	EUTSA_v10020525mg	Acetyl-CoA acetyltransferase	80	-	-
MCB1EB_756	hpaB	Mannose-1-phosphate guanylyltransferase	94	-	-
MCB1EB_757	-	Hypothetical protein	100	71	100
MCB1EB_758	bgla_2g02110	Trypsin-like serine proteases, typically periplasmic, contain C-terminal PDZ domain	100	-	-
MCB1EB_759	bgla_2g02120	FOG: CBS domain	100	30	89
MCB1EB_760	METH_21655	Hypothetical protein	94	29	86
MCB1EB_761	BgramDRAFT_0557	Fe2+ transport system protein B	100	-	-
MCB1EB_762	yscR	Type III secretory pathway, component EscS	100	56	100
MCB1EB_763	PMI06_000610	Type III secretory pathway, component EscR	100	67	100
MCB1EB_764	BgramDRAFT_0561	Flagellar motor switch/type III secretory pathway protein	100	31	93
MCB1EB_765	-	Hypothetical protein	100	-	-
MCB1EB_766	sctU	Type III secretory pathway, component EscV	100	66	45
MCB1EB_767	queF	Flagellar biosynthesis pathway, component FlhB	100	48	100
MCB1EB_768	ilvA	Enzyme related to GTP cyclohydrolase I	100	67	100
MCB1EB_769	CGI_10022907	Threonine dehydratase	100	75	100
MCB1EB_770	-	Membrane carboxypeptidase/penicillin-binding protein	-	-	-
MCB1EB_771	-	FAD/FMN-containing dehydrogenases	100	77	100
MCB1EB_772	ubiE	Diadenosine tetraphosphate (Ap4A) hydrolase and other HIT family hydrolases	100	55	76
MCB1EB_773	BC1002_0370	Methylase involved in ubiquinone/menaquinone biosynthesis	100	76	100

MCB1EB_774	BamMEX5DRAFT_4566	Uncharacterized protein conserved in bacteria	100	47	90
MCB1EB_775	TBLA0B04730	Type II secretory pathway, component PulD	100	46	100
MCB1EB_776	TMO_c0168	FOG: WD40 repeat	100	-	-
MCB1EB_777	Jab_2c00820	Hypothetical protein	100	-	-
MCB1EB_778	ARALYDRAFT_663337	Uncharacterized conserved protein	100	-	-
MCB1EB_779	M595_1342	Hypothetical protein	85	-	-
MCB1EB_780	Halhy_2265	Leucine-rich repeat (LRR) protein	100	-	-
MCB1EB_781	Halhy_2265	Predicted NTPase (NACHT family)	66-91	-	-
MCB1EB_782	PMI16_02582	Predicted NTPase (NACHT family)	66-92	-	-
MCB1EB_783	ligA	Response regulator containing a CheY-like receiver domain and an HTH DNA-binding domain	100	-	-
MCB1EB_784	glnD	NAD-dependent DNA ligase (contains BRCT domain type II)	100	70	99
MCB1EB_785	map	UTP:GlnB (protein PII) uridylyltransferase	100	67	98
MCB1EB_786		Methionine aminopeptidase	86	81	86
MCB1EB_787	DSC_04030	Integrase	-	-	-
MCB1EB_788	-	Response regulators consisting of a CheY-like receiver domain and a winged-helix DNA-binding domain	-	-	-
MCB1EB_789	-	Dehydrogenases with different specificities (related to short-chain alcohol dehydrogenases)	-	-	-
MCB1EB_790	O166_08720	Transposase and inactivated derivatives	-	-	-
MCB1EB_791	RRP5-2	Transposase and inactivated derivatives	-	-	-
MCB1EB_792	UU7_15460	Hypothetical protein	-	-	-
MCB1EB_793	AOL_s00004g332	Zn-dependent hydrolases, including glyoxylases	-	-	-
MCB1EB_794	-	Small-conductance mechanosensitive channel	-	-	-
MCB1EB_795	-	InterPro IPR001584 COGs COG2801	100	-	-
MCB1EB_796	GMDG_00079	InterPro IPR001584 COGs COG2801	-	-	-
MCB1EB_797	KM1_230160	Glutathione synthase/Ribosomal protein S6 modification enzyme (glutaminyl transferase)	75	-	-
MCB1EB_798	CCALI_00757	Pyruvate/2-oxoglutarate dehydrogenase complex, dihydrolipoamide dehydrogenase (E3) component, and related enzymes	98	-	-
MCB1EB_799	-	Dephospho-CoA kinase	-	-	-
MCB1EB_800	-	Integral membrane protein possibly involved in chromosome condensation	100	58	98
MCB1EB_801	-	Asp-tRNAAsn/Glu-tRNAGln amidotransferase A subunit and related amidases	100	49	95
MCB1EB_802	metE	Transcriptional regulator	100	34	95
MCB1EB_803	Reut_C6143	Methionine synthase II (cobalamin-independent)	100	54	99
MCB1EB_804	hipA	Predicted transcriptional regulators	92	-	-
MCB1EB_805	-	Uncharacterized protein related to capsule biosynthesis enzymes	-	-	-
MCB1EB_806	Paz_35	Aminopeptidase N	100	65	99
MCB1EB_807	PCC21_027950	Leucyl-tRNA synthetase	-	-	-
MCB1EB_808	Sant_P0287	Hypothetical protein	-	-	-
MCB1EB_809	-	Uncharacterized conserved protein	-	-	-
MCB1EB_810	chaB	Predicted membrane protein	-	57	-
MCB1EB_811	BCh11DRAFT_07142	Putative cation transport regulator	-	-	-
MCB1EB_812	WQE_32336	Exonuclease V gamma subunit	100	50	100
MCB1EB_813	recD	ATP-dependent exoDNAse (exonuclease V) beta subunit (contains helicase and exonuclease domains)	99	51	97
MCB1EB_814	X636_03970	ATP-dependent exoDNAse (exonuclease V), alpha subunit - helicase superfamily I member	100	52	93
MCB1EB_815	THITH_00215	Chemotaxis protein histidine kinase and related kinases	100	-	-
MCB1EB_816	-	Hypothetical protein	100	-	-
MCB1EB_817	-	Membrane-bound lytic murein transglycosylase B	100	57	94
MCB1EB_818	LYNGBM3L_24300	Uncharacterized conserved protein	-	-	-
MCB1EB_819	-	Predicted NTPase (NACHT family)	78-96	-	-
MCB1EB_820	metN	Predicted membrane protein	100	-	97
MCB1EB_821	-	ABC-type metal ion transport system, ATPase component	100	73	100
MCB1EB_822	-	ABC-type metal ion transport system, permease component	100	77	100
MCB1EB_823	-	ABC-type metal ion transport system, periplasmic component/surface antigen	100	58-67	100
MCB1EB_824	-	Electron transfer flavoprotein, beta subunit	100	80	100
MCB1EB_825	-	Electron transfer flavoprotein, alpha subunit	100	72	99
MCB1EB_826	PT7_0544	Acyl-CoA dehydrogenases	100	64	97
MCB1EB_827	CAGGBEG34_220029	Predicted transcriptional regulators	100	-	-
MCB1EB_828	SS1G_05485	Uncharacterized protein related to capsule biosynthesis enzymes	100	-	-
MCB1EB_829	-	Predicted NTPase (NACHT family)	-	-	-
MCB1EB_830	rpsP	DNA repair photolyase	99	66	97
MCB1EB_831	rimM	Ribosomal protein S16	100	75	93
MCB1EB_832	trmD	RimM protein, required for 16S rRNA processing	100	49	95
MCB1EB_833	rplS	tRNA-(guanine-N1)-methyltransferase	100	72	97
MCB1EB_834	cobD	Ribosomal protein L19	100	-	-
MCB1EB_835	A986_18323	Cobalamin biosynthesis protein CobD/CbiB	100	70	100
MCB1EB_836	engC	FOG: TPR repeat	98	-	-
MCB1EB_837	-	Predicted GTPases	100	65	100
MCB1EB_838	orn	Zn-dependent protease with chaperone function	100	70	100
MCB1EB_839	pmbA	Oligoribonuclease (3'->5' exoribonuclease)	100	77	91
MCB1EB_840	rpoC	Predicted Zn-dependent proteases and their inactivated homologs	100	73	100
MCB1EB_841	BcDW1_3665	Precorrin-3B methylase	-	-	-
MCB1EB_842	Halhy_2265	Predicted NTPase (NACHT family)	78-96	-	-
MCB1EB_843	-	Predicted NTPase (NACHT family)	78-96	-	-
MCB1EB_844	folA	Transposase and inactivated derivatives	-	-	-
MCB1EB_845	thyA	Dihydrofolate reductase	100	70	99
MCB1EB_846	dusA	Thymidylate synthase	100	56	99
MCB1EB_847	lexA	tRNA-dihydrouridine synthase	83	-	-
MCB1EB_848	-	SOS-response transcriptional repressors (RecA-mediated autopeptidases)	100	70	100
MCB1EB_849	rpsB	L-asparaginase/archaeal Glu-tRNAGln amidotransferase subunit D	100	64	95
MCB1EB_850	tsf	Ribosomal protein S2	100	81	100
MCB1EB_851	pyrH	Translation elongation factor Ts	100	76	95
MCB1EB_852	frr	Uridylate kinase	100	92	97
MCB1EB_853	uppS	Ribosome recycling factor	100	78	100
MCB1EB_854	RBRH_03570	Undecaprenyl pyrophosphate synthase	100	69	96
MCB1EB_855	dxr	CDP-diglyceride synthetase	100	49	96
MCB1EB_856	-	1-deoxy-D-xylulose 5-phosphate reductoisomerase	100	68	95
MCB1EB_857	-	Outer membrane protein/protective antigen OMA87	100	72	100
MCB1EB_858	lpxD	Outer membrane protein	100	64	94
MCB1EB_859	fabZ	UDP-3-O-[3-hydroxymyristoyl] glucosamine N-acyltransferase	100	68	94
MCB1EB_860	lpxA	3-hydroxymyristoyl/3-hydroxydecanoyl-(acyl carrier protein) dehydratases	100	80	96
MCB1EB_861	lpxB	Acyl-[acyl carrier protein]--UDP-N-acetylglucosamine O-acyltransferase	100	74	100
MCB1EB_862	rnhB	Lipid A disaccharide synthetase	100	65	96
MCB1EB_863	BTH_I2042	Ribonuclease HII	100	69	95
MCB1EB_864	BrE312_2629	rRNA methylases	100	56	100
MCB1EB_865	-	Uncharacterized conserved protein	100	-	98
MCB1EB_866	ppsA	Uncharacterized protein conserved in bacteria	100	82	100
MCB1EB_867	BDAG_01288	Phosphoenolpyruvate synthase/pyruvate phosphate dikinase	100	84	100
MCB1EB_868	-	Hypothetical protein	100	48	99
MCB1EB_869	BN131_3651	Membrane protease subunits, stomatin/prohibitin homologs	100	-	-

MCB1EB_870	DOTSEDRAFT_70016	Nucleoid-associated protein	100	-	-
MCB1EB_871	smpB	Histone acetyltransferase HPA2 and related acetyltransferases	100	-	-
MCB1EB_872	-	tmRNA-binding protein	100	79	98
MCB1EB_873	WQE_31521	Oligoketide cyclase/lipid transport protein	100	-	-
MCB1EB_874	guaB	Uncharacterized protein conserved in bacteria	100	60	95
MCB1EB_875	guaA	IMP dehydrogenase/GMP reductase	100	86	100
MCB1EB_876	-	GMP synthase, PP-ATPase domain/subunit	100	80	100
MCB1EB_877	Halhy_2265	Transposase and inactivated derivatives	-	-	-
MCB1EB_878	tnpA	Predicted NTPase (NACHT family)	-	-	-
MCB1EB_879	-	Transposase and inactivated derivatives	-	53	-
MCB1EB_880	KGM_22366	Transposase and inactivated derivatives	-	64	-
MCB1EB_881	N234_15805	FOG: HEAT repeat	-	-	-
MCB1EB_882	plu1110	Transposase and inactivated derivatives	-	-	-
MCB1EB_883	-	FOG: WD40 repeat	-	-	-
MCB1EB_884	-	Retron-type reverse transcriptase	100	-	-
MCB1EB_885	HNWD1	Transposase and inactivated derivatives	-	-	-
MCB1EB_886	-	Predicted NTPase (NACHT family)	66-91	-	-
MCB1EB_887	rep	Predicted NTPase (NACHT family)	66-91	-	-
MCB1EB_888	A33K_12849	Superfamily I DNA and RNA helicases	100	77	99
MCB1EB_889	RCFBP_10023	Cytochrome c5	100	51	100
MCB1EB_890	-	Uncharacterized protein conserved in bacteria	90	-	-
MCB1EB_891	-	K+-transporting ATPase, A chain	100	66	100
MCB1EB_892	-	High-affinity K+ transport system, ATPase chain B	100	79	99
MCB1EB_893	PMI14_06932	K+-transporting ATPase, c chain	100	62	99
MCB1EB_894	-	Hypothetical protein	100	-	-
MCB1EB_895	-	Osmosensitive K+ channel histidine kinase	100	63	98
MCB1EB_896	Veis_0806	Response regulators consisting of a CheY-like receiver domain and a winged-helix DNA-binding domain	100	75	100
MCB1EB_897	-	Uncharacterized conserved protein	-	-	-
MCB1EB_898	NAS141_02841	Superfamily II DNA/RNA helicases, SNF2 family	-	-	-
MCB1EB_899	mfd	Hypothetical protein	-	-	-
MCB1EB_900	ispD	Transcription-repair coupling factor (superfamily II helicase)	100	77	99
MCB1EB_901	ispF	4-diphosphocytidyl-2-methyl-D-erithritol synthase	100	69	94
MCB1EB_902	clpB	2C-methyl-D-erythritol 2,4-cyclodiphosphate synthase	100	72	99
MCB1EB_903	M472_06040	ATPases with chaperone activity, ATP-binding subunit	100	-	-
MCB1EB_904	-	Negative regulator of beta-lactamase expression	100	-	-
MCB1EB_905	-	Superfamily II RNA helicase	100	-	-
MCB1EB_906	rhlE2	Predicted membrane GTPase involved in stress response	100	84	98
MCB1EB_907	-	Superfamily II DNA and RNA helicases	100	71	96
MCB1EB_908	Hsero_1294	Predicted sulfurtransferase	-	72	97
MCB1EB_909	v1g109364	Large exoproteins involved in heme utilization or adhesion	-	-	-
MCB1EB_910	THAPSDRAFT_3777	Dihydrodipicolinate synthase/N-acetylneuraminate lyase	99	-	-
MCB1EB_911	RPSI07_mp1112	3-methyladenine DNA glycosylase	100	-	-
MCB1EB_912	C266_17566	Large exoproteins involved in heme utilization or adhesion	90-97	38	-
MCB1EB_913	PTE_00017	ATPase related to the helicase subunit of the Holliday junction resolvase	-	-	-
MCB1EB_914	HNWD1	Large exoproteins involved in heme utilization or adhesion	-	-	-
MCB1EB_915	BDB_mp40104	Predicted NTPase (NACHT family)	81-100	-	-
MCB1EB_916	-	Predicted glutamine amidotransferase involved in pyridoxine biosynthesis	-	-	-
MCB1EB_917	-	Glucose-6-phosphate isomerase	-	-	-
MCB1EB_918	-	Transposase and inactivated derivatives	100	65	-
MCB1EB_919	dnaE	Transposase and inactivated derivatives	100	58	-
MCB1EB_920	RBRH_00570	DNA polymerase III, alpha subunit	100	74	100
MCB1EB_921	-	Na+/citrate symporter	88-100	57-58	-
MCB1EB_922	-	InterPro IPR001584 COGs COG2801	-	-	-
MCB1EB_923	AN3547.2	Glycerophosphoryl diester phosphodiesterase	-	-	-
MCB1EB_924	HCAG_07455	FOG: TPR repeat	-	-	-
MCB1EB_925	Rpic12D_2004	FOG: HEAT repeat	-	-	-
MCB1EB_926	-	Glycosyltransferases involved in cell wall biogenesis	100	-	-
MCB1EB_927	CAGGBEG34_210069	Glycosyltransferases involved in cell wall biogenesis	100	-	-
MCB1EB_928	msbA	Lipid A core - O-antigen ligase and related enzymes	100	-	-
MCB1EB_929	cafA	ABC-type multidrug transport system, ATPase and permease components	100	73	98
MCB1EB_930	-	Ribonucleases G and E	100	77	100
MCB1EB_931	nadD	Uncharacterized homolog of plant Iojap protein	100	73	100
MCB1EB_932	hemF	Nicotinic acid mononucleotide adenylyltransferase	100	54	98
MCB1EB_933	purD	Coproporphyrinogen III oxidase	100	67	98
MCB1EB_934	-	Phosphoribosylamine-glycine ligase	100	79	99
MCB1EB_935	-	Uncharacterized conserved protein	100	82	99
MCB1EB_936	scpB	16S rRNA uridine-516 pseudouridylate synthase and related pseudouridylate synthases	100	59	87
MCB1EB_937	BURK_000910	Predicted transcriptional regulator containing the HTH domain	100	-	-
MCB1EB_938	Calla_1546	Membrane proteins related to metalloendopeptidases	100	54	92
MCB1EB_939	CAGGBEG34_330054	ATP-dependent protease HslVU (ClpYQ), ATPase subunit	100	-	-
MCB1EB_940	R0011_03965	Hypothetical protein	-	-	-
MCB1EB_941	F886_02160	Hypothetical protein	-	-	-
MCB1EB_942	-	Hypothetical protein	-	-	-
MCB1EB_943	-	Uncharacterized conserved small protein	-	76	-
MCB1EB_944	BgramDRAFT_6427	Inactive homolog of metal-dependent proteases, putative molecular chaperone	75	-	-
MCB1EB_945	-	Acetyltransferases	100	-	-
MCB1EB_946	SG0735	Uracil-DNA glycosylase	100	-	-
MCB1EB_947	thiD	Uncharacterized conserved protein	100	35-36	-
MCB1EB_948	lpiT	Hydroxymethylpyrimidine/phosphomethylpyrimidine kinase	100	71	100
MCB1EB_949	-	Permeases of the major facilitator superfamily	100	71	100
MCB1EB_950	sms	Alanine racemase	100	68	99
MCB1EB_951	HCAG_07455	Predicted ATP-dependent serine protease	100	84	98
MCB1EB_952	dnaX	FOG: HEAT repeat	94	-	95
MCB1EB_953	dnaX	DNA polymerase III, gamma/tau subunits	100	-	-
MCB1EB_954	-	DNA polymerase III, gamma/tau subunits	100	-	-
MCB1EB_955	recR	Uncharacterized protein conserved in bacteria	100	77	100
MCB1EB_956	iscX	Recombinational DNA repair protein (RecF pathway)	84	78	98
MCB1EB_957	-	Uncharacterized protein conserved in bacteria	100	82	100
MCB1EB_958	-	Universal stress protein UspA and related nucleotide-binding proteins	100	-	-
MCB1EB_959	cobN	Superfamily II DNA and RNA helicases	100	-	-
MCB1EB_960	-	Hypothetical protein	86	-	-
MCB1EB_961	Bcep18194_A3475	Superfamily I DNA and RNA helicases	100	69	100
MCB1EB_962	RBRH_00037	Uncharacterized protein involved in tolerance to divalent cations	90	57	80
MCB1EB_963	HMPREF1482_00429	Hypothetical protein	-	-	-
MCB1EB_964	RBRH_03890	Hypothetical protein	-	-	-
MCB1EB_965	HMPREF0497_0173	Hypothetical protein	100	-	-
MCB1EB_966	thrB	Hypothetical protein	-	-	-
MCB1EB_967	-	Putative homoserine kinase type II (protein kinase fold)	100	64	100
MCB1EB_968	-	Nucleoside phosphorylase	100	75	100
MCB1EB_969	polA	Predicted Rossmann fold nucleotide-binding protein	97	77	98
MCB1EB_970	rpoB	DNA polymerase I - 3'-5' exonuclease and polymerase domains	100	70	100
MCB1EB_971	-	DNA-directed RNA polymerase, beta subunit/140 kD subunit	-	-	-
MCB1EB_972	LEP1GSC108_1530	NADH dehydrogenase, FAD-containing subunit	100	61	97

MCB1EB_973	Dtox_3121	Leucine-rich repeat (LRR) protein	100	-	-
MCB1EB_974	rpoC2	Hypothetical protein	99	-	-
MCB1EB_975	RSp1073	Thymidylate kinase	97	-	-
MCB1EB_976	Entcl_4309	Large exoproteins involved in heme utilization or adhesion		39	-
MCB1EB_977	sseA	NAD-dependent aldehyde dehydrogenases	100	-	-
MCB1EB_978	-	Rhodanese-related sulfurtransferase	-	-	-
MCB1EB_979	-	Rhodanese-related sulfurtransferase	-	-	-
MCB1EB_980	RBRH_00570	Phenylpropionate dioxygenase and related ring-hydroxylating dioxygenases, large terminal subunit	100	72	98
MCB1EB_981	A33K_13045	Na+/citrate symporter	94-100	55	-
MCB1EB_982	PA6_003_01060	Spermidine/putrescine-binding periplasmic protein	100	-	-
MCB1EB_983	-	ABC-type spermidine/putrescine transport system, permease component II	99	-	-
MCB1EB_984	-	ABC-type spermidine/putrescine transport system, permease component I	90	27	-
MCB1EB_985	-	ABC-type spermidine/putrescine transport systems, ATPase components	100	-	-
MCB1EB_986	BTH_II0086	Transposase and inactivated derivatives, IS5 family	-	82	-
MCB1EB_987	PA6_003_01060	Spermidine/putrescine-binding periplasmic protein	96	-	-
MCB1EB_988	PACG_03516	ABC-type spermidine/putrescine transport system, permease component II	-	-	-
MCB1EB_989	BP1026A_1768	ABC-type spermidine/putrescine transport system, permease component I	-	30	-
MCB1EB_990	BTI_4604	AraC-type DNA-binding domain-containing proteins	-	-	-
MCB1EB_991	O166_14525	Histone acetyltransferase HPA2 and related acetyltransferases	-	-	-
MCB1EB_992	thiE	Transaldolase	100	-	-
MCB1EB_993	thiG	Thiamine monophosphate synthase	87	56	95
MCB1EB_994	Bphyt_3574	Uncharacterized enzyme of thiazole biosynthesis	100	75	99
MCB1EB_995	-	Sulfur transfer protein involved in thiamine biosynthesis	100	51	100
MCB1EB_996	xseB	Glycine/D-amino acid oxidases (deaminating)	94	62	96
MCB1EB_997	ispA	Exonuclease VII small subunit	100	-	-
MCB1EB_998	dxs	Geranylgeranyl pyrophosphate synthase	100	66	99
MCB1EB_999	-	Deoxyxylulose-5-phosphate synthase	100	75-79	96
MCB1EB_1000	gcp	Uncharacterized conserved protein	100	72	98
MCB1EB_1001	LEP1GSC043_2144	Metal-dependent proteases with possible chaperone activity	100	79	100
MCB1EB_1002	rpsU	Uncharacterized conserved protein	100	-	-
MCB1EB_1003	-	Ribosomal protein S21	100	96	100
MCB1EB_1004	dnaG	Uncharacterized conserved protein	100	68	96
MCB1EB_1005	rpoD	DNA primase (bacterial type)	100	66	99
MCB1EB_1006	-	DNA-directed RNA polymerase, sigma subunit (sigma70/sigma32)	100	79	94
MCB1EB_1007	-	Uncharacterized protein, possibly involved in aromatic compounds catabolism	100	-	-
MCB1EB_1008	-	Predicted aminomethyltransferase related to GcvT	100	-	-
MCB1EB_1009	tmk	Predicted periplasmic solute-binding protein	100	56	88
MCB1EB_1010	holB	Thymidylate kinase	100	67	99
MCB1EB_1011	BC1002_1487	ATPase involved in DNA replication	100	56	98
MCB1EB_1012	-	Sortase and related acyltransferases	100	-	-
MCB1EB_1013	I634_08040	Mg-dependent DNase	100	70	96
MCB1EB_1014	-	Predicted acyltransferases	100	-	-
MCB1EB_1015	GWL_35760	Hypothetical protein	-	-	-
MCB1EB_1016	KYG_08880	Hypothetical protein	100	36	89
MCB1EB_1017	-	Predicted transcriptional regulators	-	-	-
MCB1EB_1018	Jab_2c03390	Hypothetical protein	100	-	-
MCB1EB_1019	PMI35_04561	Transcriptional regulator	100	-	-
MCB1EB_1020	Nit79A3_1107	Anti-sigma factor	100	-	-
MCB1EB_1021	istB	Membrane protein involved in the export of O-antigen and teichoic acid	-	-	-
MCB1EB_1022	-	Transposase and inactivated derivatives	-	42	-
MCB1EB_1023	Halhy_2265	Transposase and inactivated derivatives	100	-	-
MCB1EB_1024	nusG	Predicted NTPase (NACHT family)	66-91	-	-
MCB1EB_1025	-	Transcription antiterminator	-	-	-
MCB1EB_1026	-	Hypothetical protein	-	-	-
MCB1EB_1027	-	Nucleotidyltransferase/DNA polymerase involved in DNA repair	-	69	-
MCB1EB_1028	traC	DNA polymerase III, alpha subunit	-	78	-
MCB1EB_1029	CC1G_08648	Uncharacterized protein conserved in bacteria	-	-	-
MCB1EB_1030	TVY486_1109920	ABC-type multidrug transport system, ATPase and permease components	-	-	-
MCB1EB_1031	PMI16_02591	Hypothetical protein	100	-	-
MCB1EB_1032	CELE_F35C5.3	Signal transduction histidine kinase	100	-	-
MCB1EB_1033	-	Autotransporter adhesin	100	-	-
MCB1EB_1034	-	Hypothetical protein	100	53	100
MCB1EB_1035	M942_23135	Chromosome segregation ATPases	100	68	100
MCB1EB_1036	PMI06_003799	Uncharacterized conserved small protein	100	-	-
MCB1EB_1037	dapC	Permeases of the drug/metabolite transporter (DMT) superfamily	100	-	-
MCB1EB_1038	dapD	Aspartate/tyrosine/aromatic aminotransferase	100	69	99
MCB1EB_1039	-	Tetrahydrodipicolinate N-succinyltransferase	100	78	100
MCB1EB_1040	dapE	Arsenate reductase and related proteins, glutaredoxin family	100	58	96
MCB1EB_1041	yfcB	Acetylornithine deacetylase/Succinyl-diaminopimelate desuccinylase and related deacylases	98	70	97
MCB1EB_1042	cls	Methylase of polypeptide chain release factors	100	-	-
MCB1EB_1043	-	Phosphatidylserine/phosphatidylglycerophosphate/cardiolipin synthases and related enzymes	100	63	100
MCB1EB_1044	BURMUCGD2M_1311	ATPase components of ABC transporters with duplicated ATPase domains	100	68	100
MCB1EB_1045	CFU_3359	Hypothetical protein	100	-	-
MCB1EB_1046	PMI24_05069	Hypothetical protein	100	-	-
MCB1EB_1047	RBRH_02472	Uncharacterized protein conserved in bacteria	100	-	-
MCB1EB_1048	RBRH_02471	ATPases involved in chromosome partitioning	-	70	-
MCB1EB_1049	HCH_03142	Uncharacterized conserved protein	-	65	-
MCB1EB_1050	Npun_F1222	Histidyl-tRNA synthetase	-	-	-
MCB1EB_1051	-	Predicted NTPase (NACHT family)	78-96	-	-
MCB1EB_1052	RBRH_01189	Transposase and inactivated derivatives	-	-	-
MCB1EB_1053	RoseRS_4025	Superfamily II helicase, archaea-specific	-	-	-
MCB1EB_1054	-	Predicted NTPase (NACHT family)	66-91	-	-
MCB1EB_1055	-	Predicted nucleoside-diphosphate sugar epimerase	100	-	-
MCB1EB_1056	PSE_3910	Dehydrogenases with different specificities (related to short-chain alcohol dehydrogenases)	100	-	-
MCB1EB_1057	-	Predicted ATPase	-	-	-
MCB1EB_1058	-	Bacterial lipocalin	100	-	-
MCB1EB_1059	C380_16345	Predicted membrane protein	-	-	-
MCB1EB_1060	Halhy_2265	Predicted NAD/FAD-dependent oxidoreductase	86	-	-
MCB1EB_1061	-	Predicted NTPase (NACHT family)	99-100	-	-
MCB1EB_1062	-	Transposase and inactivated derivatives	97	-	-
MCB1EB_1063	-	Transposase and inactivated derivatives	-	-	-
MCB1EB_1064	LYNGBM3L_24300	Uncharacterized conserved protein	100	61	96
MCB1EB_1065	tnpA	Predicted NTPase (NACHT family)	70-100	-	-
MCB1EB_1066	VCHENC01_3983	ABC-type Fe3+-siderophore transport system, permease component	100	-	-

MCB1EB_1067	GSPATT00025202001	Predicted unusual protein kinase	-	-	-
MCB1EB_1068	-	Predicted NTPase (NACHT family)	-	-	-
MCB1EB_1069	lysC	Integrase	-	-	-
MCB1EB_1070	BUPH_02948	Aspartokinases	97	82	99
MCB1EB_1071	RBRH_00578	Permeases of the drug/metabolite transporter (DMT) superfamily	89	-	93
MCB1EB_1072	-	Non-ribosomal peptide synthetase modules and related proteins	-	-	-
MCB1EB_1073	CAGGBEG34_30027	Thioesterase domains of type I polyketide synthases or non-ribosomal peptide synthetases	-	-	-
MCB1EB_1074	-	Retron-type reverse transcriptase	96	31	-
MCB1EB_1075	tilS	Transposase and inactivated derivatives	100	65	-
MCB1EB_1076	accA	Predicted ATPase of the PP-loop superfamily implicated in cell cycle control	100	47	99
MCB1EB_1077	-	Acetyl-CoA carboxylase alpha subunit	100	88	97
MCB1EB_1078	cysS	3-methyladenine DNA glycosylase/8-oxoguanine DNA glycosylase	100	-	-
MCB1EB_1079	RBRH_03961	Cysteinyl-tRNA synthetase	100	70	100
MCB1EB_1080	ppiB	FOG: TPR repeat	100	-	-
MCB1EB_1081	-	Peptidyl-prolyl cis-trans isomerase (rotamase) - cyclophilin family	100	75	99
MCB1EB_1082	BN668_01709	Uncharacterized protein conserved in bacteria	99	54	97
MCB1EB_1083	PROVALCAL_01421	Glycosyltransferase	-	-	-
MCB1EB_1084	-	L-alanine-DL-glutamate epimerase and related enzymes of enolase superfamily	-	-	-
MCB1EB_1085	Halhy_2265	Transposase and inactivated derivatives	100	60	75
MCB1EB_1086	-	Predicted NTPase (NACHT family)	99	-	-
MCB1EB_1087	suhB	rRNA methylase	99	66	91
MCB1EB_1088	U875_08940	Archaeal fructose-1,6-bisphosphatase and related enzymes of inositol monophosphatase family	100	76	98
MCB1EB_1089	RBRH_03529	Diacylglycerol kinase	100	-	-
MCB1EB_1090	-	Phosphoglycerol transferase and related proteins, alkaline phosphatase superfamily	100	-	98
MCB1EB_1091	-	Predicted ATPase	100	73	100
MCB1EB_1092	sucB	Pyruvate/2-oxoglutarate dehydrogenase complex, dihydrolipoamide dehydrogenase (E3) component, and related enzymes	100	79	100
MCB1EB_1093	sucA	Pyruvate/2-oxoglutarate dehydrogenase complex, dihydrolipoamide acyltransferase (E2) component, and related enzymes	100	73	100
MCB1EB_1094	fdx-1	2-oxoglutarate dehydrogenase complex, dehydrogenase (E1) component, and related enzymes	100	87	86
MCB1EB_1095	hscA	Ferredoxin	100	70	100
MCB1EB_1096	hscB	Molecular chaperone	100	70	100
MCB1EB_1097	-	DnaJ-domain-containing proteins 1	100	57	100
MCB1EB_1098	-	Uncharacterized conserved protein	100	86	100
MCB1EB_1099	iscS	NifU homolog involved in Fe-S cluster formation	100	88	96
MCB1EB_1100	-	Cysteine sulfinate desulfinase/cysteine desulfurase and related enzymes	100	83	100
MCB1EB_1101	bglu_1g19220	Predicted transcriptional regulator	100	74	98
MCB1EB_1102	BgramDRAFT_5853	Permeases of the major facilitator superfamily	100	31-32	-
MCB1EB_1103	-	Predicted membrane protein	98	49	94
MCB1EB_1104	-	Predicted transcriptional regulators	100	-	-
MCB1EB_1105	-	Uncharacterized protein related to capsule biosynthesis enzymes	100	-	-
MCB1EB_1106	-	Uncharacterized protein conserved in bacteria	100	59	86
MCB1EB_1107	-	Uncharacterized protein conserved in bacteria	100	50	82
MCB1EB_1108	HNWD1	Uncharacterized protein involved in formation of curli polymers	91	73	85
MCB1EB_1109	leuA	Predicted NTPase (NACHT family)	99-100	-	-
MCB1EB_1110	pssA	Isopropylmalate/homocitrate/citramalate synthases	100	79	100
MCB1EB_1111	psd	Phosphatidylserine synthase	100	75	98
MCB1EB_1112	ilvC	Phosphatidylserine decarboxylase	100	72	100
MCB1EB_1113	ilvH	Ketol-acid reductoisomerase	100	85	100
MCB1EB_1114	ilvB	Acetolactate synthase, small (regulatory) subunit	100	82	100
MCB1EB_1115	-	Thiamine pyrophosphate-requiring enzymes [acetolactate synthase, pyruvate dehydrogenase (cytochrome), glyoxylate carboligase, phosphonopyruvate decarboxylase]	100	82	99
MCB1EB_1116	Bphy_2024	DNA-directed RNA polymerase specialized sigma subunit, sigma24 homolog	100	72	100
MCB1EB_1117	BCh11DRAFT_00132	Hypothetical protein	100	50	98
MCB1EB_1118	-	Hypothetical protein	100	-	-
MCB1EB_1119	BUPH_00305	Transcriptional regulator	100	79	97
MCB1EB_1120	-	Mu-like prophage protein	100	34	99
MCB1EB_1121	N234_09080	Hypothetical protein	100	-	-
MCB1EB_1122	-	Uncharacterized conserved protein	-	-	-
MCB1EB_1123	RBRH_01044	Pirin-related protein	100	42	96
MCB1EB_1124	RBRH_01045	Uncharacterized enzyme of heme biosynthesis	100	57	99
MCB1EB_1125	BgramDRAFT_1913	Uncharacterized enzyme of heme biosynthesis	100	-	-
MCB1EB_1126	hemC	Uroporphyrinogen-III synthase	100	-	-
MCB1EB_1127	SG0735	Porphobilinogen deaminase	100	-	-
MCB1EB_1128	argH	Uncharacterized conserved protein	100	33-35	-
MCB1EB_1129	ldcC	Argininosuccinate lyase	100	78	86
MCB1EB_1130	dcd	Arginine/lysine/ornithine decarboxylases	100	86	100
MCB1EB_1131	RoseRS_4025	Deoxycytidine deaminase	100	90	99
MCB1EB_1132	-	Predicted NTPase (NACHT family)	-	-	-
MCB1EB_1133	RBRH_00037	ABC-type transport system involved in Fe-S cluster assembly, permease and ATPase components	100	66	94
MCB1EB_1134	RBRH_03890	Hypothetical protein	100	59-65	-
MCB1EB_1135	-	Hypothetical protein	100	59	100
MCB1EB_1136	RBRH_03890	Hypothetical protein	-	63	-
MCB1EB_1137	CAGGBEG34_550003	Nitrate reductase beta subunit	100	-	-
MCB1EB_1138	mrp	Cu/Zn superoxide dismutase	100	46	91
MCB1EB_1139	-	ATPases involved in chromosome partitioning	100	72	85
MCB1EB_1140	OR16_03807	Outer membrane protein and related peptidoglycan-associated (lipo)proteins	100	64	96
MCB1EB_1141	RALTA_B0823	Spermidine/putrescine-binding periplasmic protein	-	-	-
MCB1EB_1142	OR16_03807	Transcriptional regulator	-	42	-
MCB1EB_1143	L810_8411	Spermidine/putrescine-binding periplasmic protein	100	-	-
MCB1EB_1144	D769_12681	Transcriptional regulator	93	32	86
MCB1EB_1145	Cagg_1814	Na+/citrate symporter	84-86	53-54	-
MCB1EB_1146	Tc00.1047053511557.50	Predicted NTPase (NACHT family)	-	-	-
MCB1EB_1147	MetexDRAFT_1696	Predicted transcriptional regulators	-	-	-
MCB1EB_1148	metG	Hypothetical protein	-	-	-
MCB1EB_1149	Bphyt_1014	Methionyl-tRNA synthetase	100	75	99
MCB1EB_1150	-	Uncharacterized protein conserved in bacteria	100	47	95
MCB1EB_1151	-	Outer membrane protein	100	59	96
MCB1EB_1152	-	Hypothetical protein	100	74	93
MCB1EB_1153	panC	Uncharacterized conserved protein	100	75	90
MCB1EB_1154	panD	Panthothenate synthetase	100	69	100
MCB1EB_1155	K788_8069	Aspartate 1-decarboxylase	100	77	100
MCB1EB_1156	OR16_03807	ABC-type Fe^{3+}-hydroxamate transport system, periplasmic component	100	50	93
MCB1EB_1157	WQE_37849	Spermidine/putrescine-binding periplasmic protein	99-100	-	-
MCB1EB_1158	-	Transcriptional regulator	-	-	-
MCB1EB_1159	U875_17425	Transcriptional regulator	-	-	-
MCB1EB_1160	Tery_1841	Spermidine/putrescine-binding periplasmic protein	-	-	-
MCB1EB_1161	Entcl_4309	FOG: HEAT repeat	-	-	-

MCB1EB_1162	D781_0052	Response regulator containing CheY-like receiver domain and AraC-type DNA-binding domain	-	-	-
MCB1EB_1163	F504_865	Hypothetical protein	-	-	-
MCB1EB_1164	-	Large exoproteins involved in heme utilization or adhesion	-	-	-
MCB1EB_1165	RSPO_m00925	18 : Unknown function	100	-	-
MCB1EB_1166	PTE_02404	Large exoproteins involved in heme utilization or adhesion	-	-	-
MCB1EB_1167	RSPO_m00925	Hypothetical protein	-	-	-
MCB1EB_1168		Large exoproteins involved in heme utilization or adhesion	-	-	-
MCB1EB_1169	H045_00290	Transposase and inactivated derivatives, IS5 family	-	82	-
MCB1EB_1170	TKWG_21470	Large exoproteins involved in heme utilization or adhesion	-	-	-
MCB1EB_1171	aroC	Large exoproteins involved in heme utilization or adhesion	-	-	-
MCB1EB_1172	-	Chorismate synthase	100	82	100
MCB1EB_1173	pdhD	FOG: CBS domain	100	75	99
MCB1EB_1174	-	Pyruvate/2-oxoglutarate dehydrogenase complex, dihydrolipoamide dehydrogenase (E3) component, and related enzymes	100	78	97
MCB1EB_1175	aceE	Pyruvate/2-oxoglutarate dehydrogenase complex, dihydrolipoamide acyltransferase (E2) component, and related enzymes	100	-	-
MCB1EB_1176	folD	Pyruvate dehydrogenase complex, dehydrogenase (E1) component	100	83	100
MCB1EB_1177	prlC	5,10-methylene-tetrahydrofolate dehydrogenase/Methenyl tetrahydrofolate cyclohydrolase	100	78	97
MCB1EB_1178	SRU_2653	Zn-dependent oligopeptidases	100	74	99
MCB1EB_1179	TNMX_08450	Putative dehydrogenase domain of multifunctional non-ribosomal peptide synthetases and related enzymes	100	-	-
MCB1EB_1180	TSTA_059840	Antitoxin of toxin-antitoxin stability system	100	-	-
MCB1EB_1181	SERLA73DRAFT_77359	Predicted NTPase (NACHT family)	92-93	-	-
MCB1EB_1182	GGTG_12522	Thiamine pyrophosphate-requiring enzymes [acetolactate synthase, pyruvate dehydrogenase (cytochrome), glyoxylate carboligase, phosphonopyruvate decarboxylase]	-	-	-
MCB1EB_1183	BRAFLDRAFT_98235	Hypothetical protein	-	-	-
MCB1EB_1184	BgramDRAFT_3627	Hypothetical protein	-	-	-
MCB1EB_1185	-	Hypothetical protein	100	-	-
MCB1EB_1186	BC1003_2657	Acyl carrier protein	100	-	-
MCB1EB_1187	hutH	Acyl-coenzyme A synthetases/AMP-(fatty) acid ligases	100	-	-
MCB1EB_1188	-	Histidine ammonia-lyase	100	34	88
MCB1EB_1189	-	Predicted thioesterase	100	-	-
MCB1EB_1190	K788_3336	3-oxoacyl-(acyl-carrier-protein) synthase	100	91	-
MCB1EB_1191	fabG	Predicted 3-hydroxylacyl-(acyl carrier protein) dehydratase	100	-	-
MCB1EB_1192	RoseRS_4025	Dehydrogenases with different specificities (related to short-chain alcohol dehydrogenases)	100	-	-
MCB1EB_1193	-	Predicted NTPase (NACHT family)	66-91	-	-
MCB1EB_1194	-	ABC-type branched-chain amino acid transport systems, periplasmic component	96	-	-
MCB1EB_1195	N878_22445	Multidrug resistance efflux pump	100	63	99
MCB1EB_1196	mutS	Uncharacterized conserved small protein	100	-	-
MCB1EB_1197	slyD	Mismatch repair ATPase (MutS family)	97	72	97
MCB1EB_1198	CAGGBEG34_200032	FKBP-type peptidyl-prolyl cis-trans isomerases 2	100	65	92
MCB1EB_1199	MYA_1877	Predicted small secreted protein	-	-	-
MCB1EB_1200	dapA	Uncharacterized lipoprotein	100	-	-
MCB1EB_1201	trpS	Dihydrodipicolinate synthase/N-acetylneuraminate lyase	100	72	96
MCB1EB_1202	-	Tryptophanyl-tRNA synthetase	100	86	100
MCB1EB_1203	-	Zn-dependent proteases	100	66	99
MCB1EB_1204	bgla_1g27740	Predicted metal-dependent phosphoesterases (PHP family)	100	65	100
MCB1EB_1205	mscL	Hypothetical protein	100	-	-
MCB1EB_1206	-	Large-conductance mechanosensitive channel	100	71	99
MCB1EB_1207	-	D-alanyl-D-alanine carboxypeptidase	100	67	76
MCB1EB_1208	-	ABC-type spermidine/putrescine transport system, permease component II	96	-	-
MCB1EB_1209	-	ABC-type spermidine/putrescine transport system, permease component I	100	-	-
MCB1EB_1210	-	ABC-type spermidine/putrescine transport systems, ATPase components	100	-	-
MCB1EB_1211	HMPREF0731_1372	Spermidine/putrescine-binding periplasmic protein	93	-	-
MCB1EB_1212	STRIC_0577	Predicted flavoprotein	100	-	-
MCB1EB_1213	pyrD	Glutathione S-transferase	100	-	-
MCB1EB_1214	pyrD	Dihydroorotate dehydrogenase	100	-	96
MCB1EB_1215	-	Dihydroorotate dehydrogenase	-	-	-
MCB1EB_1216	-	NADPH-dependent glutamate synthase beta chain and related oxidoreductases	100	78	100
MCB1EB_1217	-	Transposase and inactivated derivatives	-	-	-
MCB1EB_1218	-	Transposase and inactivated derivatives	-	-	-
MCB1EB_1219	tnpA2	Transposase and inactivated derivatives	-	-	-
MCB1EB_1220	-	Hypothetical protein	-	64	-
MCB1EB_1221	CNE_BB1p04810	Transposase and inactivated derivatives	-	-	-
MCB1EB_1222	-	Transposase and inactivated derivatives	-	-	-
MCB1EB_1223	-	Hypothetical protein	-	-	-
MCB1EB_1224	PMI24_02838	Transposase and inactivated derivatives	-	-	-
MCB1EB_1225	-	Hypothetical protein	-	-	-
MCB1EB_1226	CARN7_2937	Integrase	95	-	-
MCB1EB_1227	-	Transposase and inactivated derivatives	-	-	-
MCB1EB_1228	CARN7_2937	Transposase and inactivated derivatives	-	-	-
MCB1EB_1229	-	Hypothetical protein	-	-	-
MCB1EB_1230	CARN7_2937	Transposase and inactivated derivatives	100	65	-
MCB1EB_1231	-	Predicted glutamine amidotransferase	-	-	-
MCB1EB_1232	gltX	DNA replication protein	-	-	-
MCB1EB_1233	-	Glutamyl- and glutaminyl-tRNA synthetases	94	75	84
MCB1EB_1234	-	Transposase and inactivated derivatives, IS5 family	-	-	-
MCB1EB_1235	-	ABC-type Fe3+ transport system, periplasmic component	-	-	-
MCB1EB_1236	-	Cell wall-associated hydrolases (invasion-associated proteins)	100	61	96
MCB1EB_1237	-	ABC-type uncharacterized transport system, duplicated ATPase component	100	66	99
MCB1EB_1238	-	ABC-type uncharacterized transport system, permease component	100	70	95
MCB1EB_1239	-	ABC-type uncharacterized transport system, permease component	100	76	100
MCB1EB_1240	fabI	ABC-type oligopeptide transport system, periplasmic component	95	63	92
MCB1EB_1241	N9414_09841	Enoyl-[acyl-carrier-protein] reductase (NADH)	100	80	100
MCB1EB_1242	-	Predicted NTPase (NACHT family)	100	-	-
MCB1EB_1243	glnA1	Rhodanese-related sulfurtransferase	100	59	99
MCB1EB_1244	-	Glutamine synthetase	100	88	100
MCB1EB_1245	XSR1_50074	Tellurite resistance protein and related permeases	100	-	-
MCB1EB_1246	-	Hypothetical protein	-	30	-
MCB1EB_1247	-	Transposase and inactivated derivatives, IS5 family	-	82	-
MCB1EB_1248	-	Stress-induced morphogen (activity unknown)	100	61	89
MCB1EB_1249	-	Intracellular septation protein A	100	60	100
MCB1EB_1250	purL	Conserved domain frequently associated with peptide methionine sulfoxide reductase	100	66	96
MCB1EB_1251	Mfla_0907	Phosphoribosylformylglycinamidine (FGAM) synthase, synthetase domain	100	72	100
MCB1EB_1252	M23134_05452	Predicted transcriptional regulator	-	-	-
MCB1EB_1253	-	Leucine-rich repeat (LRR) protein	-	-	-
MCB1EB_1254	NB231_14001	Transposase and inactivated derivatives	-	-	-

MCB1EB_1255	S7335_4732	Transposase and inactivated derivatives	-	-	-
MCB1EB_1256	pgi	Leucine-rich repeat (LRR) protein	-	-	-
MCB1EB_1257	BamMC406_1833	Glucose-6-phosphate isomerase	100	67	99
MCB1EB_1258	A1I_05390	Glycine/D-amino acid oxidases (deaminating)	100	-	-
MCB1EB_1259	RBRH_01614	Preprotein translocase subunit SecA (ATPase, RNA helicase)	-	-	-
MCB1EB_1260	lon	Parvulin-like peptidyl-prolyl isomerase	100	-	99
MCB1EB_1261	clpX	ATP-dependent Lon protease, bacterial type	100	85	100
MCB1EB_1262	clpP	ATP-dependent protease Clp, ATPase subunit	100	86	93
MCB1EB_1263	tig	Protease subunit of ATP-dependent Clp proteases	100	83	100
MCB1EB_1264	gyrB	FKBP-type peptidyl-prolyl cis-trans isomerase (trigger factor)	100	56	99
MCB1EB_1265	HMPREF1015_00064	Type IIA topoisomerase (DNA gyrase/topo II, topoisomerase IV), B subunit	-	-	-
MCB1EB_1266	trxA	Cytotoxic translational repressor of toxin-antitoxin stability system	-	-	-
MCB1EB_1267	rho	Thiol-disulfide isomerase and thioredoxins	100	70	100
MCB1EB_1268	rpmE	Transcription termination factor	100	94	100
MCB1EB_1269	-	Ribosomal protein L31	100	-	-
MCB1EB_1270	MYA_3532	4-amino-4-deoxy-L-arabinose transferase and related glycosyltransferases of PMT family	100	64	99
MCB1EB_1271	int	Permeases of the major facilitator superfamily	100	-	-
MCB1EB_1272	bglu_1g03560	Integrase	-	27	-
MCB1EB_1273	uvrB	Hypothetical protein	-	-	-
MCB1EB_1274	RBRH_00570	Low specificity phosphatase (HAD superfamily)	-	-	-
MCB1EB_1275	CAGGBEG34_610004	Na+/citrate symporter	99-100	49-53	-
MCB1EB_1276	L332_13090	Lhr-like helicases	-	41	-
MCB1EB_1277	CAGGBEG34_190021	Glycyl-tRNA synthetase, beta subunit	-	-	-
MCB1EB_1278	atpA	Phage-related protein, predicted endonuclease	-	-	-
MCB1EB_1279	CAGGBEG34_190142	Excinuclease ATPase subunit	-	-	-
MCB1EB_1280	AciX9_3324	Hypothetical protein	100	-	-
MCB1EB_1281	BURPS668_A2366	Phage anti-repressor protein	99	-	-
MCB1EB_1282	O991_02545	Predicted transcriptional regulator	-	-	-
MCB1EB_1283	V757_00985	Phosphatidylinositol kinase and protein kinases of the PI-3 kinase family	-	-	-
MCB1EB_1284	B1A_21845	Hypothetical protein	-	-	-
MCB1EB_1285	CAGGBEG34_330013	Uncharacterized protein conserved in bacteria	-	-	-
MCB1EB_1286	BDB_30040	Acyl-CoA dehydrogenases	-	-	-
MCB1EB_1287	CAGGBEG34_330022	Holliday junction resolvase	99	-	-
MCB1EB_1288	KYC_12668	Pyruvate-formate lyase-activating enzyme	-	-	-
MCB1EB_1289	N234_18785	Hypothetical protein	99	-	-
MCB1EB_1290	-	Predicted NAD/FAD-binding protein	100	-	-
MCB1EB_1291	-	Uncharacterized protein conserved in bacteria	89-100	31	-
MCB1EB_1292	XFEB_01759	Uncharacterized protein conserved in bacteria	100	-	-
MCB1EB_1293	BC1002_6545	Uncharacterized protein, homolog of phage Mu protein gp30	100	29	-
MCB1EB_1294	XF_1576	Uncharacterized protein conserved in bacteria	100	-	-
MCB1EB_1295	BC1002_6547	Hypothetical protein	-	-	-
MCB1EB_1296	BC1002_6549	Uncharacterized protein conserved in bacteria	99	-	-
MCB1EB_1297	gene0107	Hypothetical protein	99	-	-
MCB1EB_1298	PD_0974	Hypothetical protein	-	-	-
MCB1EB_1299	BC1002_6552	Hypothetical protein	-	-	-
MCB1EB_1300	L682_09905	Hypothetical protein	-	-	-
MCB1EB_1301	B398_05775	Hypothetical protein	100	20	-
MCB1EB_1302	L682_09915	Hypothetical protein	-	-	-
MCB1EB_1303	-	Hypothetical protein	-	-	-
MCB1EB_1304	BC1002_6556	Hypothetical protein	-	-	-
MCB1EB_1305	HMPREF0178_03708	Hypothetical protein	100	22	-
MCB1EB_1306	L682_09935	Hypothetical protein	-	-	-
MCB1EB_1307	HIB_15810	Hypothetical protein	-	-	-
MCB1EB_1308	L682_09945	Hypothetical protein	100	26	-
MCB1EB_1309	L682_09950	Hypothetical protein	-	28	-
MCB1EB_1310	L682_09955	Hypothetical protein	100	-	-
MCB1EB_1311	L682_09960	Uncharacterized homolog of phage Mu protein gp47	100	26	-
MCB1EB_1312	L682_09965	Hypothetical protein	-	-	-
MCB1EB_1313	SfIV_21	Hypothetical protein	100	-	-
MCB1EB_1314	-	Hypothetical protein	99	-	-
MCB1EB_1315	CAGGBEG34_220024	Hypothetical protein	-	71	-
MCB1EB_1316	CAGGBEG34_330054	Permeases of the major facilitator superfamily	100	51	94-98
MCB1EB_1317	LYNGBM3L_10820	Hypothetical protein	-	-	-
MCB1EB_1318	-	Predicted NTPase (NACHT family)	66-91	-	-
MCB1EB_1319	MAE_58050	Hypothetical protein	100	-	-
MCB1EB_1320	Tint_1965	FOG: TPR repeat	-	-	-
MCB1EB_1321	Tint_1966	Uncharacterized protein conserved in bacteria	-	-	-
MCB1EB_1322	int	Uncharacterized protein conserved in bacteria	-	-	-
MCB1EB_1323	Aasi_1766	Integrase	-	-	-
MCB1EB_1324	Npun_F1419	DNA segregation ATPase FtsK/SpoIIIE and related proteins	-	-	-
MCB1EB_1325	L810_7852	Predicted NTPase (NACHT family)	99-100	-	-
MCB1EB_1326	M942_10535	Transposase and inactivated derivatives	-	-	-
MCB1EB_1327	RBRH_02548	FOG: TPR repeat	98	-	-
MCB1EB_1328	EPM1_0524	DNA uptake protein and related DNA-binding proteins	-	-	-
MCB1EB_1329	relA	Predicted permeases	100	-	-
MCB1EB_1330	etf	Guanosine polyphosphate pyrophosphohydrolases/synthetases	100	66	98
MCB1EB_1331	-	Dehydrogenases (flavoproteins)	100	77	98
MCB1EB_1332	-	ABC-type cobalamin/Fe3+-siderophores transport systems, ATPase components	100	58	91
MCB1EB_1333	BURK_025795	ABC-type Fe3+-siderophore transport system, permease component	99	56	99
MCB1EB_1334	BYI23_A005940	Outer membrane cobalamin receptor protein	100	46	94
MCB1EB_1335	-	Hypothetical protein	100	46	93
MCB1EB_1336	lgt	Hypothetical protein	100	69	90
MCB1EB_1337	ilvD	Prolipoprotein diacylglyceryltransferase	100	33	96
MCB1EB_1338	holC	Dihydroxyacid dehydratase/phosphogluconate dehydratase	100	-	-
MCB1EB_1339	pepA	DNA polymerase III, chi subunit	100	63	100
MCB1EB_1340	-	Leucyl aminopeptidase	100	65	100
MCB1EB_1341	-	Predicted permeases	100	72	97
MCB1EB_1342	BUPH_06468	Predicted permeases	100	72	97
MCB1EB_1343	Bphyt_0969	Uncharacterized conserved protein	100	-	-
MCB1EB_1344	BUPH_04618	Transcriptional regulator	100	44-59	94
MCB1EB_1345	QWC_02034	Uncharacterized conserved protein	100	-	-
MCB1EB_1346	HMPREF1004_04590	Flp pilus assembly protein, ATPase CpaF	100	-	-
MCB1EB_1347	BUPH_02420	Transposase and inactivated derivatives	100	-	-
MCB1EB_1348	HMPREF1004_04592	Type IV secretory pathway, VirB4 components	100	-	-
MCB1EB_1349	BC1001_1963	Conjugal transfer/entry exclusion protein	100	-	-
MCB1EB_1350	HMPREF1004_04595	Type IV secretory pathway, VirB6 components	100	-	-
MCB1EB_1351	BC1001_1966	ABC-type polar amino acid transport system, ATPase component	100	-	-
MCB1EB_1352	BUPH_02427	Type IV secretory pathway, VirB9 components	100	-	-
MCB1EB_1353	HMPREF0004_2499	Type IV secretory pathway, VirB10 components	100	-	-
MCB1EB_1354	OR214_02188	Type IV secretory pathway, VirB9 components	100	-	-
MCB1EB_1355	EDWATA_03480	Predicted Zn-dependent peptidases	100	-	-
MCB1EB_1356	Rmet_6292	FOG: PAS/PAC domain	100	-	-

MCB1EB_1357	OR214_02191	Dinucleotide-utilizing enzymes involved in molybdopterin and thiamine biosynthesis family 1	100	-	-
MCB1EB_1358	OR214_02192	Type II secretory pathway, ATPase PulE/Tfp pilus assembly pathway, ATPase PilB	100	-	-
MCB1EB_1359	L810_0422	Type II secretory pathway, component PulF	100	-	-
MCB1EB_1360	L810_0421	Transcriptional regulators	100	-	-
MCB1EB_1361	BUPH_06486	Phosphomannomutase	83	-	-
MCB1EB_1362	RSPO_c02513	Hypothetical protein	94	-	-
MCB1EB_1363	xcc-b100_3058	Importin, protein involved in nuclear import	100	-	-
MCB1EB_1364	RSPO_c02513	Hypothetical protein	70	-	-
MCB1EB_1365	CAGGBEG34_500001	Di- and tripeptidases	100	-	-
MCB1EB_1366	Bphy_5667	SAM-dependent methyltransferases	100	-	-
MCB1EB_1367	QWC_13492	Na+/citrate symporter	-	-	-
MCB1EB_1368	Bcep1808_1159	Rad3-related DNA helicases	100	-	-
MCB1EB_1369	-	Predicted transcriptional regulators	100	-	-
MCB1EB_1370	-	Zn-dependent hydrolases, including glyoxylases	100	68	98
MCB1EB_1371	-	Transposase and inactivated derivatives	-	-	-
MCB1EB_1372	RoseRS_4025	Transposase and inactivated derivatives	100	56-58	90
MCB1EB_1373	-	Predicted NTPase (NACHT family)	66-91	-	-
MCB1EB_1374	O162_07910	Predicted NTPase (NACHT family)	-	-	-
MCB1EB_1375	ldcA	Guanosine polyphosphate pyrophosphohydrolases/synthetases	-	-	-
MCB1EB_1376	-	Uncharacterized proteins, homologs of microcin C7 resistance protein MccF	100	62	98
MCB1EB_1377	-	Cytosine/adenosine deaminases	100	71	98
MCB1EB_1378	Nit79A3_0051	Transposase and inactivated derivatives	-	-	-
MCB1EB_1379	-	Predicted oxidoreductases (related to aryl-alcohol dehydrogenases)	-	-	-
MCB1EB_1380	-	Transposase and inactivated derivatives	88	-	-
MCB1EB_1381	-	Transposase and inactivated derivatives	100	65	-
MCB1EB_1382	RoseRS_4025	Transposase and inactivated derivatives	93	49	86
MCB1EB_1383	Bcep1808_1184	Predicted NTPase (NACHT family)	66-91	-	-
MCB1EB_1384	ahpD	Serine/threonine protein kinase	-	-	-
MCB1EB_1385	ahpC1	Uncharacterized conserved protein	100	-	-
MCB1EB_1386	-	Peroxiredoxin	100	34	84
MCB1EB_1387	ompR	Signal transduction histidine kinase	92	73	90
MCB1EB_1388	truB	Response regulators consisting of a CheY-like receiver domain and a winged-helix DNA-binding domain	100	91	100
MCB1EB_1389	rbfA	Pseudouridine synthase	100	-	-
MCB1EB_1390	infB	Ribosome-binding factor A	100	76	100
MCB1EB_1391	nusA	Translation initiation factor 2 (IF-2; GTPase)	100	-	-
MCB1EB_1392	-	Transcription elongation factor	100	83	100
MCB1EB_1393	thiC	Uncharacterized protein conserved in bacteria	100	-	-
MCB1EB_1394	-	Thiamine biosynthesis protein ThiC	100	84	99
MCB1EB_1395	PPSC2_c3662	Hypothetical protein	100	67	67
MCB1EB_1396	NH44784_027271	Hypothetical protein	100	-	-
MCB1EB_1397	C266_24825	Spermidine/putrescine-binding periplasmic protein	100	-	-
MCB1EB_1398	BUPH_06600	Transcriptional regulator	100	32	93
MCB1EB_1399	-	Anaerobic dehydrogenases, typically selenocysteine-containing	100	41	82
MCB1EB_1400	metC	Phosphoserine phosphatase	100	63	100
MCB1EB_1401	rimO	Cystathionine beta-lyases/cystathionine gamma-synthases	100	70	98
MCB1EB_1402	rnr	2-methylthioadenine synthetase	100	79	97
MCB1EB_1403	rlmB	Exoribonuclease R	100	72	95
MCB1EB_1404	rpiA	rRNA methylases	100	75	98
MCB1EB_1405	-	Ribose 5-phosphate isomerase	100	63	97
MCB1EB_1406	-	DNA uptake lipoprotein	100	71	95
MCB1EB_1407	BgramDRAFT_2322	Pseudouridylate synthases, 23S RNA-specific	100	65	83
MCB1EB_1408	-	Uncharacterized conserved protein	100	54	96
MCB1EB_1409	SS1G_07990	Uncharacterized protein conserved in bacteria	100	67	91
MCB1EB_1410	-	Predicted NTPase (NACHT family)	-	-	-
MCB1EB_1411	-	Hypothetical protein	-	-	-
MCB1EB_1412	CAGGBEG34_420004	Integrase	100	27	-
MCB1EB_1413	B738_22335	Hypothetical protein	100	-	-
MCB1EB_1414	L485_22335	Mismatch repair ATPase (MutS family)	100	-	-
MCB1EB_1415	uvrC	Hypothetical protein	-	-	-
MCB1EB_1416	RBRH_00570	Serine/threonine protein kinase	-	-	-
MCB1EB_1417	BURCENK562V_C3062	Na+/citrate symporter	92-100	51-54	-
MCB1EB_1418	RBRH_04010	Transcriptional regulator	100	-	-
MCB1EB_1419	RBRH_01048	Hypothetical protein	100	47	98
MCB1EB_1420	QWA_03220	Zn-dependent protease with chaperone function	100	49	100
MCB1EB_1421	APP7_0464	Methyl-accepting chemotaxis protein	89	56	84
MCB1EB_1422	PLO_0633	Hypothetical protein	-	-	-
MCB1EB_1423	CAGGBEG34_210001	Excinuclease ATPase subunit	-	-	-
MCB1EB_1424	AciX9_3324	Hypothetical protein	-	-	-
MCB1EB_1425	CAGGBEG34_350021	Phage anti-repressor protein	100	-	-
MCB1EB_1426	AcdelDRAFT_0859	Predicted transcriptional regulator	-	-	-
MCB1EB_1427	MYCTH_23848	Uncharacterized protein conserved in bacteria, prophage-related	-	44	-
MCB1EB_1428	V757_00985	Uncharacterized conserved protein	-	-	-
MCB1EB_1429	CAGGBEG34_330012	Bacterial cell division membrane protein	-	-	-
MCB1EB_1430	CAGGBEG34_330013	Hypothetical protein	100	-	-
MCB1EB_1431	BDB_30040	Acyl-CoA dehydrogenases	100	-	-
MCB1EB_1432	CAGGBEG34_330022	Holliday junction resolvase	-	-	-
MCB1EB_1433	CAGGBEG34_120004	Pyruvate-formate lyase-activating enzyme	100	-	-
MCB1EB_1434	-	Uncharacterized protein conserved in bacteria	-	-	-
MCB1EB_1435	-	Predicted transcriptional regulator	-	42-45	-
MCB1EB_1436	PA2G_00805	Uncharacterized protein conserved in bacteria	-	60	-
MCB1EB_1437	-	Acetyl-CoA carboxylase, carboxyltransferase component (subunits alpha and beta)	-	-	-
MCB1EB_1438	-	Uncharacterized protein conserved in bacteria	95-100	32	-
MCB1EB_1439	BC1002_6544	Uncharacterized protein conserved in bacteria	100	-	-
MCB1EB_1440	BC1002_6545	Uncharacterized protein, homolog of phage Mu protein gp30	100	30	-
MCB1EB_1441	XF_1576	Uncharacterized protein conserved in bacteria	100	-	-
MCB1EB_1442	BC1002_6547	Hypothetical protein	-	-	-
MCB1EB_1443	BC1002_6549	Uncharacterized protein conserved in bacteria	99	-	-
MCB1EB_1444	gene0107	Hypothetical protein	99-100	-	-
MCB1EB_1445	PD_0974	Hypothetical protein	-	-	-
MCB1EB_1446	BC1002_6552	Hypothetical protein	-	-	-
MCB1EB_1447	L682_09905	Hypothetical protein	100	-	-
MCB1EB_1448	B398_05775	Hypothetical protein	100	20	-
MCB1EB_1449	P910_003116	Hypothetical protein	100	-	-
MCB1EB_1450	-	Hypothetical protein	-	-	-
MCB1EB_1451	BC1002_6556	Hypothetical protein	-	-	-
MCB1EB_1452	-	Hypothetical protein	100	22	-
MCB1EB_1453	L682_09935	Hypothetical protein	96	-	-
MCB1EB_1454	HIB_15810	Hypothetical protein	-	-	-
MCB1EB_1455	L682_09945	Hypothetical protein	100	25	77
MCB1EB_1456	L682_09950	Hypothetical protein	100	32	-

MCB1EB_1457	L682_09955	Hypothetical protein	-	-	-
MCB1EB_1458	L682_09960	Uncharacterized homolog of phage Mu protein gp47	100	26	-
MCB1EB_1459	L682_09965	Hypothetical protein	-	-	-
MCB1EB_1460	SFV_0255	Hypothetical protein	100	-	-
MCB1EB_1461	-	Hypothetical protein	99	-	-
MCB1EB_1462	CAGGBEG34_220024	Hypothetical protein	100	72	99
MCB1EB_1463	CAGGBEG34_330054	Permeases of the major facilitator superfamily	-	53	-
MCB1EB_1464	Halhy_2265	Hypothetical protein	-	-	-
MCB1EB_1465	BGP_4141	Predicted NTPase (NACHT family)	70-100	-	-
MCB1EB_1466	-	Predicted NTPase (NACHT family)	99-100	-	-
MCB1EB_1467	CAGGBEG34_420004	Integrase	100	29	-
MCB1EB_1468	B738_22335	Hypothetical protein	100	-	-
MCB1EB_1469	L485_22335	Mismatch repair ATPase (MutS family)	100	-	-
MCB1EB_1470	-	Hypothetical protein	-	-	-
MCB1EB_1471	RBRH_00570	Serine/threonine protein kinase	-	-	-
MCB1EB_1472	tnpA2	Na+/citrate symporter	-	-	-
MCB1EB_1473	RBRH_00570	Hypothetical protein	-	64	-
MCB1EB_1474	CAGGBEG34_610004	Na+/citrate symporter	-	-	-
MCB1EB_1475	BURCENK562V_C3071	Transglutaminase-like enzymes, putative cysteine proteases	100	44	100
MCB1EB_1476	BURCENK562V_C3072	Hypothetical protein	100	-	-
MCB1EB_1477	-	Signal transduction histidine kinase	-	-	-
MCB1EB_1478	-	Hypothetical protein	-	-	-
MCB1EB_1479	SDRG_14715	Hypothetical protein	100	-	-
MCB1EB_1480	Veis_0670	Excinuclease ATPase subunit	-	-	-
MCB1EB_1481	AciX9_3324	Hypothetical protein	-	-	-
MCB1EB_1482	mEp043_050	Phage anti-repressor protein	100	-	-
MCB1EB_1483	G838_04777	Predicted transcriptional regulator	-	-	-
MCB1EB_1484	-	Uncharacterized protein conserved in bacteria, prophage-related	-	-	-
MCB1EB_1485	V757_00985	Predicted dehydrogenase	100	-	-
MCB1EB_1486	CAGGBEG34_330012	Bacterial cell division membrane protein	-	-	-
MCB1EB_1487	CAGGBEG34_330013	Hypothetical protein	100	-	-
MCB1EB_1488	BDB_30040	Acyl-CoA dehydrogenases	-	-	-
MCB1EB_1489	CAGGBEG34_330022	Holliday junction resolvase	-	-	-
MCB1EB_1490	HMPREF0005_02877	Pyruvate-formate lyase-activating enzyme	100	-	-
MCB1EB_1491	N234_18785	Hypothetical protein	97	-	-
MCB1EB_1492	-	Hypothetical protein	-	-	-
MCB1EB_1493	-	Uncharacterized protein conserved in bacteria	95-100	31	-
MCB1EB_1494	XFEB_01759	Uncharacterized protein conserved in bacteria	100	-	-
MCB1EB_1495	BC1002_6545	Uncharacterized protein, homolog of phage Mu protein gp30	100	30	-
MCB1EB_1496	XF_1576	Uncharacterized protein conserved in bacteria	100	-	-
MCB1EB_1497	BC1002_6547	Hypothetical protein	-	-	-
MCB1EB_1498	BC1002_6549	Uncharacterized protein conserved in bacteria	99	-	-
MCB1EB_1499	BC1002_6550	Hypothetical protein	99-100	-	-
MCB1EB_1500	B398_05790	Hypothetical protein	100	-	-
MCB1EB_1501	BC1002_6552	Hypothetical protein	100	-	-
MCB1EB_1502	L682_09905	Hypothetical protein	100	-	-
MCB1EB_1503	B398_05775	Hypothetical protein	100	21	-
MCB1EB_1504	B375_09910	Hypothetical protein	-	-	-
MCB1EB_1505	-	Hypothetical protein	-	-	-
MCB1EB_1506	BC1002_6556	Hypothetical protein	-	-	-
MCB1EB_1507	HMPREF9095_1683	Hypothetical protein	100	23	-
MCB1EB_1508	L682_09935	Hypothetical protein	-	-	-
MCB1EB_1509	HMPREF9095_1680	Hypothetical protein	100	-	-
MCB1EB_1510	L682_09945	Hypothetical protein	100	25	77
MCB1EB_1511	L682_09950	Hypothetical protein	-	27	-
MCB1EB_1512	L682_09955	Hypothetical protein	-	-	-
MCB1EB_1513	L682_09960	Uncharacterized homolog of phage Mu protein gp47	100	27	-
MCB1EB_1514	L682_09965	Hypothetical protein	100	-	-
MCB1EB_1515	SFV_0255	Hypothetical protein	100	-	-
MCB1EB_1516	-	Hypothetical protein	-	-	-
MCB1EB_1517	CAGGBEG34_220024	Hypothetical protein	100	71	-
MCB1EB_1518	CAGGBEG34_330054	Permeases of the major facilitator superfamily	100	53	94
MCB1EB_1519	RoseRS_4025	Hypothetical protein	100	-	-
MCB1EB_1520	Calni_0847	Predicted NTPase (NACHT family)	66-91	-	-
MCB1EB_1521	ANA_C12029	Predicted NTPase (NACHT family)	66-91	-	-
MCB1EB_1522	NECAME_13904	Predicted NTPase (NACHT family)	66-91	-	-
MCB1EB_1523	-	Predicted GTPases	-	-	-
MCB1EB_1524	CAGGBEG34_420004	Integrase	100	28	-
MCB1EB_1525	B738_22335	Hypothetical protein	100	-	-
MCB1EB_1526	L485_22335	Hypothetical protein	100	-	-
MCB1EB_1527	-	Hypothetical protein	-	-	-
MCB1EB_1528	RBRH_00570	Serine/threonine protein kinase	-	-	-
MCB1EB_1529	CAGGBEG34_610004	Na+/citrate symporter	99-100	49-52	-
MCB1EB_1530	BDB_30064	Acetate kinase	-	41	-
MCB1EB_1531	Reut_A2376	ATPase involved in DNA repair	-	-	-
MCB1EB_1532	BDB_30060	Hypothetical protein	-	-	-
MCB1EB_1533	SCHCODRAFT_110303	Hypothetical protein	-	-	-
MCB1EB_1534	CAGGBEG34_910002	Transcription termination factor	-	-	-
MCB1EB_1535	AciX9_3324	Hypothetical protein	-	-	-
MCB1EB_1536	G419_09581	Phage anti-repressor protein	100	-	-
MCB1EB_1537	HMPREF9530_00525	ABC-type multidrug transport system, ATPase and permease components	-	-	-
MCB1EB_1538	BAnh1_02300	SOS-response transcriptional repressors (RecA-mediated autopeptidases)	-	-	-
MCB1EB_1539	C817_04332	Uncharacterized protein conserved in bacteria, prophage-related	-	-	-
MCB1EB_1540	V757_00985	Cell division protein FtsI/penicillin-binding protein 2	-	-	-
MCB1EB_1541	CAGGBEG34_330012	Cdc6-related protein, AAA superfamily ATPase	-	-	-
MCB1EB_1542	CAGGBEG34_330013	Hypothetical protein	98	-	-
MCB1EB_1543	BDB_30040	Protein involved in catabolism of external DNA	-	-	-
MCB1EB_1544	CAGGBEG34_330022	Holliday junction resolvase	98	-	-
MCB1EB_1545	KYC_12668	Pyruvate-formate lyase-activating enzyme	-	-	-
MCB1EB_1546	PA2G_00805	Hypothetical protein	99	-	-
MCB1EB_1547	-	Zn-dependent protease with chaperone function	-	-	-
MCB1EB_1548	-	Uncharacterized protein conserved in bacteria	90-100	32	-
MCB1EB_1549	-	Predicted nucleic acid-binding protein, contains PIN domain	100	45	-
MCB1EB_1550	BC1002_6543	Virulence-associated protein and related proteins	100	-	-
MCB1EB_1551	BC1002_6544	Uncharacterized protein conserved in bacteria	99-100	-	-
MCB1EB_1552	BC1002_6545	Uncharacterized protein, homolog of phage Mu protein gp30	100	29	-
MCB1EB_1553	XF_1576	Uncharacterized protein conserved in bacteria	99-100	-	-
MCB1EB_1554	BC1002_6547	Hypothetical protein	-	-	-
MCB1EB_1555	BC1002_6549	Uncharacterized protein conserved in bacteria	99	-	-
MCB1EB_1556	gene0107	Hypothetical protein	99-100	-	-
MCB1EB_1557	PD_0974	Hypothetical protein	-	-	-
MCB1EB_1558	BC1002_6552	Hypothetical protein	100	-	-
MCB1EB_1559	L682_09905	Hypothetical protein	-	-	-
MCB1EB_1560	B398_05775	Hypothetical protein	100	21	-
MCB1EB_1561	B375_09910	Hypothetical protein	-	-	-

MCB1EB_1562	-	Hypothetical protein	100	-	-
MCB1EB_1563	BC1002_6556	Hypothetical protein	-	-	-
MCB1EB_1564	HIBPF_09270	Hypothetical protein	100	22	-
MCB1EB_1565	L682_09935	Hypothetical protein	-	-	-
MCB1EB_1566	HMPREF9095_1680	Hypothetical protein	-	-	-
MCB1EB_1567	L682_09945	Hypothetical protein	100	25	-
MCB1EB_1568	L682_09950	Hypothetical protein	-	28	-
MCB1EB_1569	L682_09955	Hypothetical protein	100	-	-
MCB1EB_1570	L682_09960	Uncharacterized homolog of phage Mu protein gp47	100	26	-
MCB1EB_1571	L682_09965	Hypothetical protein	-	-	-
MCB1EB_1572	SFV_0255	Hypothetical protein	100	-	-
MCB1EB_1573	-	Hypothetical protein	99	-	-
MCB1EB_1574	BC1002_6570	Hypothetical protein	100	66	99
MCB1EB_1575	CAGGBEG34_330054	Phosphotransferase system IIC components, glucose/maltose/N-acetylglucosamine-specific	70-100	53	-
MCB1EB_1576	Halhy_2265	Hypothetical protein	100	-	-
MCB1EB_1577	Halhy_2265	Predicted NTPase (NACHT family)	-	-	-
MCB1EB_1578	-	Predicted NTPase (NACHT family)	99-100	-	-
MCB1EB_1579	CAGGBEG34_420004	Integrase	100	26	-
MCB1EB_1580	B738_22335	Hypothetical protein	99-100	-	-
MCB1EB_1581	uvrC	Hypothetical protein	75-100	-	-
MCB1EB_1582	RBRH_00570	Predicted exonuclease of the beta-lactamase fold involved in RNA processing	-	-	-
MCB1EB_1583	CAGGBEG34_610004	Na+/citrate symporter	99-100	49-53	-
MCB1EB_1584	BDB_30064	Acetate kinase	-	42	-
MCB1EB_1585	-	Hypothetical protein	-	-	-
MCB1EB_1586	-	Transposase and inactivated derivatives	100	27-34	-
MCB1EB_1587	CAGGBEG34_330054	Transposase and inactivated derivatives	91-100	-	-
MCB1EB_1588	RoseRS_4025	Transposase and inactivated derivatives	95	-	-
MCB1EB_1589	-	Predicted NTPase (NACHT family)	78-96	-	-
MCB1EB_1590	-	Transposase and inactivated derivatives	-	-	-
MCB1EB_1591	J07HX64_02175	Transposase and inactivated derivatives	74-94	-	-
MCB1EB_1592	-	ATPase components of ABC transporters with duplicated ATPase domains	100	-	-
MCB1EB_1593	-	Transposase and inactivated derivatives	100	65	-
MCB1EB_1594	-	Transposase and inactivated derivatives	-	-	-
MCB1EB_1595	CAGGBEG34_330054	InterPro IPR001584 COGs COG2801	-	-	-
MCB1EB_1596	PODANS_4_1190	Purine-cytosine permease and related proteins	-	-	-
MCB1EB_1597	Npun_F1419	Predicted NTPase (NACHT family)	66-91	-	-
MCB1EB_1598	-	Predicted NTPase (NACHT family)	55-58	-	-
MCB1EB_1599	Halhy_2265	Transposase and inactivated derivatives	100	-	-
MCB1EB_1600	-	Predicted NTPase (NACHT family)	66-91	-	-
MCB1EB_1601	SSFG_07278	Hypothetical protein	-	-	-
MCB1EB_1602	HMPREF0476_1272	Ferritin-like protein	100	-	-
MCB1EB_1603	-	Hypothetical protein	100	-	-
MCB1EB_1604	CSV86_11535	Predicted transcriptional regulator	-	-	-
MCB1EB_1605	-	Pyrroline-5-carboxylate reductase	-	-	-
MCB1EB_1606	-	Uncharacterized protein conserved in bacteria	-	-	-
MCB1EB_1607	Cri9333_3089	Hypothetical protein	-	-	-
MCB1EB_1608	HNWD1	Predicted NTPase (NACHT family)	-	-	-
MCB1EB_1609	CAGGBEG34_420005	Predicted NTPase (NACHT family)	99-100	-	-
MCB1EB_1610	CAGGBEG34_420004	Integrase	-	-	-
MCB1EB_1611	RoseRS_4025	Hypothetical protein	-	-	-
MCB1EB_1612	RoseRS_4025	Predicted NTPase (NACHT family)	66-91	-	-
MCB1EB_1613	LYNGBM3L_24300	Predicted NTPase (NACHT family)	70-100	-	-
MCB1EB_1614	CAGGBEG34_330054	Predicted NTPase (NACHT family)	66-91	-	-
MCB1EB_1615	RSK60_1520039	Hypothetical protein	99	-	-
MCB1EB_1616	-	Hypothetical protein	100	-	-
MCB1EB_1617	-	Transposase and inactivated derivatives	100	-	-
MCB1EB_1618	-	Transposase and inactivated derivatives	100	62	99
MCB1EB_1619	CAGGBEG34_330054	Transposase and inactivated derivatives	-	-	-
MCB1EB_1620	Halhy_2265	Hypothetical protein	-	-	-
MCB1EB_1621	-	Predicted NTPase (NACHT family)	-	-	-
MCB1EB_1622	-	Transposase and inactivated derivatives	100	-	-
MCB1EB_1623	-	Transposase and inactivated derivatives	100	27-34	-
MCB1EB_1624	-	Transposase and inactivated derivatives	91	-	-
MCB1EB_1625	CAGGBEG34_330054	Predicted NTPase (NACHT family)	-	-	-
MCB1EB_1626	-	Hypothetical protein	-	-	-
MCB1EB_1627	Bcep1808_1184	Hypothetical protein	100	-	-
MCB1EB_1628	Closa_1473	Anaerobic dehydrogenases, typically selenocysteine-containing	99-100	-	-
MCB1EB_1629	HEAR2269	NTP pyrophosphohydrolases including oxidative damage repair enzymes	93-94	-	-
MCB1EB_1630	HEAR2270	Polyketide synthase modules and related proteins	100	-	-
MCB1EB_1631	HEAR2272	Hemolysin activation/secretion protein	100	-	-
MCB1EB_1632	HEAR2274	ABC-type multidrug transport system, ATPase and permease components	100	-	-
MCB1EB_1633	LPO_2922	16S rRNA uridine-516 pseudouridylate synthase and related pseudouridylate synthases	99	-	-
MCB1EB_1634	JBD24_022	Hypothetical protein	100	-	-
MCB1EB_1635	XCR1_4160004	Formate/nitrite family of transporters	100	-	-
MCB1EB_1636	HEAR2279	Hypothetical protein	100	-	-
MCB1EB_1637	Pnuc_1121	Xanthine/uracil permeases	100	-	-
MCB1EB_1638	-	Hypothetical protein	100	-	-
MCB1EB_1639	-	Predicted nucleic acid-binding protein, contains PIN domain	100	83	-
MCB1EB_1640	HEAR2281	Hypothetical protein	100	75-76	100
MCB1EB_1641	Pnuc_1125	K^+ transport systems, NAD-binding component	100	-	-
MCB1EB_1642	-	Dissimilatory sulfite reductase (desulfoviridin), alpha and beta subunits	100	-	-
MCB1EB_1643	PMI42_04850	8.1 : Prophage genes and phage related functions	100	-	-
MCB1EB_1644	DaAHT2_1258	Hypothetical protein	100	-	-
MCB1EB_1645	bbp31	Predicted ATPase	100	-	-
MCB1EB_1646	G701_02790	Predicted transcriptional regulators	100	-	-
MCB1EB_1647	Xfasm12_1180	Predicted transcriptional regulator	100	-	-
MCB1EB_1648	bbp38	NADH:flavin oxidoreductases, Old Yellow Enzyme family	100	-	-
MCB1EB_1649	bbp40	Hypothetical protein	100	-	-
MCB1EB_1650	-	Hypothetical protein	100	-	-
MCB1EB_1651	-	Uncharacterized phage-encoded protein	100	-	-
MCB1EB_1652	-	Hypothetical protein	100	-	-
MCB1EB_1653	-	Hypothetical protein	100	-	-
MCB1EB_1654	RBRH_01258	Superfamily II DNA/RNA helicases, SNF2 family	97	-	-
MCB1EB_1655	RBRH_00570	Na^+/citrate symporter	-	-	-
MCB1EB_1656	C266_17531	Na^+/citrate symporter	-	-	-
MCB1EB_1657	C266_17536	Hypothetical protein	100	-	-
MCB1EB_1658	-	Integrase	82	-	-
MCB1EB_1659	-	Putative hemolysin	100	68	87
MCB1EB_1660	ftsB	Membrane carboxypeptidase/penicillin-binding protein	100	64	-
MCB1EB_1661	eno	Septum formation initiator	76	-	-
MCB1EB_1662	kdsA	Enolase	100	88	100
MCB1EB_1663	pyrG	3-deoxy-D-manno-octulosonic acid (KDO) 8-phosphate synthase	100	82	100

MCB1EB_1664	ComA	CTP synthase (UTP-ammonia lyase)	100	84	98
MCB1EB_1665	SG1089	Predicted membrane metal-binding protein	93	44	100
MCB1EB_1666	lolD	Hypothetical protein	100	-	-
MCB1EB_1667	-	ABC-type antimicrobial peptide transport system, ATPase component	100	68	95
MCB1EB_1668	BYI23_A017700	ABC-type transport system, involved in lipoprotein release, permease component	100	74	100
MCB1EB_1669	recJ	Uncharacterized protein conserved in bacteria	100	51	96
MCB1EB_1670	prfB	Single-stranded DNA-specific exonuclease	100	76	100
MCB1EB_1671	lysS	Protein chain release factor B	100	83	92
MCB1EB_1672	Dsui_2362	Lysyl-tRNA synthetase (class II)	100	77	98
MCB1EB_1673	-	Outer membrane lipoprotein	100	45	92
MCB1EB_1674	AXXA_21803	Exonuclease III	100	70	99
MCB1EB_1675	dppA	Transcriptional regulator	100	38	100
MCB1EB_1676	-	ABC-type dipeptide transport system, periplasmic component	100	-	-
MCB1EB_1677	-	Hypothetical protein	100	-	-
MCB1EB_1678	AAI_13445	Predicted signal-transduction protein containing cAMP-binding and CBS domains	100	-	-
MCB1EB_1679	-	DNA polymerase III, epsilon subunit and related 3'-5' exonucleases	100	-	-
MCB1EB_1680	-	Choline-glycine betaine transporter	100	-	-
MCB1EB_1681	-	Transposase and inactivated derivatives	99	-	-
MCB1EB_1682	BC1001_3356	Transposase and inactivated derivatives	95	30-32	77-78
MCB1EB_1683	KYC_09711	ABC-type dipeptide transport system, periplasmic component	100	53	98
MCB1EB_1684	serS	Transcriptional regulator	100	36	96
MCB1EB_1685	-	Seryl-tRNA synthetase	100	64	99
MCB1EB_1686	-	ATPase related to the helicase subunit of the Holliday junction resolvase	100	73	97
MCB1EB_1687	ftsK	Outer membrane lipoprotein-sorting protein	100	53	91
MCB1EB_1688	trxB2	DNA segregation ATPase FtsK/SpoIIIE and related proteins	100	76	100
MCB1EB_1689	N9414_23838	Thioredoxin reductase	100	82	99
MCB1EB_1690	COXBURSA334_0199	Hypothetical protein	100	-	-
MCB1EB_1691	nadE	Hypothetical protein	100	-	-
MCB1EB_1692	secG	NAD synthase	100	72	100
MCB1EB_1693	tpiA	Preprotein translocase subunit SecG	100	60	100
MCB1EB_1694	Rleg4DRAFT_3636	Triosephosphate isomerase	100	30	95
MCB1EB_1695	-	Superfamily I DNA and RNA helicases and helicase subunits	100	59	75
MCB1EB_1696	pnp	NADPH:quinone reductase and related Zn-dependent oxidoreductases	100	71	100
MCB1EB_1697	rpsO	Polyribonucleotide nucleotidyltransferase (polynucleotide phosphorylase)	100	81	97
MCB1EB_1698	-	Ribosomal protein S15P/S13E	100	76	100
MCB1EB_1699	bglu_1g25640	Hypothetical protein	88	64	87
MCB1EB_1700	Lepto7376_1210	Hypothetical protein	98	-	84
MCB1EB_1701	-	Predicted NTPase (NACHT family)	100	-	-
MCB1EB_1702	YN1551_2013	Uncharacterized protein conserved in bacteria	100	63	100
MCB1EB_1703	-	FOG: Ankyrin repeat	92	-	-
MCB1EB_1704	nuoN	Hypothetical protein	100	59	100
MCB1EB_1705	nuoM	NADH:ubiquinone oxidoreductase subunit 2 (chain N)	100	72	100
MCB1EB_1706	nuoL	NADH:ubiquinone oxidoreductase subunit 4 (chain M)	100	77	99
MCB1EB_1707	nuoK	NADH:ubiquinone oxidoreductase subunit 5 (chain L)/Multisubunit Na+/H+ antiporter, MnhA subunit	100	72	100
MCB1EB_1708	nuoJ	NADH:ubiquinone oxidoreductase subunit 11 or 4L (chain K)	100	91	98
MCB1EB_1709	nuoI	NADH:ubiquinone oxidoreductase subunit 6 (chain J)	100	61	100
MCB1EB_1710	nuoH	Formate hydrogenlyase subunit 6/NADH:ubiquinone oxidoreductase 23 kD subunit (chain I)	100	90	99
MCB1EB_1711	nuoG	NADH:ubiquinone oxidoreductase subunit 1 (chain H)	100	82	100
MCB1EB_1712	nuoF	NADH dehydrogenase/NADH:ubiquinone oxidoreductase 75 kD subunit (chain G)	100	70	100
MCB1EB_1713	nuoE	NADH:ubiquinone oxidoreductase, NADH-binding (51 kD) subunit	100	87	98
MCB1EB_1714	nuoD	NADH:ubiquinone oxidoreductase 24 kD subunit	100	79	100
MCB1EB_1715	nuoC	NADH:ubiquinone oxidoreductase 49 kD subunit 7	100	88	100
MCB1EB_1716	nuoB	NADH:ubiquinone oxidoreductase 27 kD subunit	100	63	98
MCB1EB_1717	nuoA	NADH:ubiquinone oxidoreductase 20 kD subunit and related Fe-S oxidoreductases	100	95	100
MCB1EB_1718	RBRH_03658	NADH:ubiquinone oxidoreductase subunit 3 (chain A)	100	82	100
MCB1EB_1719	-	Uncharacterized lipoprotein	87	48	100
MCB1EB_1720	mdtB	Membrane-fusion protein	100	62	83
MCB1EB_1721	-	Cation/multidrug efflux pump	98	72	-
MCB1EB_1722	hsdM	Cation/multidrug efflux pump	100	68	-
MCB1EB_1723	-	Type I restriction-modification system methyltransferase subunit	-	-	-
MCB1EB_1724	-	Transposase and inactivated derivatives	91-100	-	-
MCB1EB_1725	BamMC406_2351	Transposase and inactivated derivatives	100	27-34	-
MCB1EB_1726	-	Transposase and inactivated derivatives	99	44	98
MCB1EB_1727	-	Transposase and inactivated derivatives	100	66	-
MCB1EB_1728	Halhy_2265	Retron-type reverse transcriptase	-	-	-
MCB1EB_1729	ACD_29C00348G0002	Predicted NTPase (NACHT family)	66-91	-	-
MCB1EB_1730	Dthio_PD2881	Uncharacterized conserved protein	-	-	-
MCB1EB_1731	-	Hypothetical protein	-	-	-
MCB1EB_1732	-	Growth inhibitor	-	-	-
MCB1EB_1733	ihfA	Predicted transcriptional regulators	100	71	98
MCB1EB_1734	pheT	Bacterial nucleoid DNA-binding protein	100	65	95
MCB1EB_1735	pheS	Phenylalanyl-tRNA synthetase beta subunit	100	65	100
MCB1EB_1736	rplT	Phenylalanyl-tRNA synthetase alpha subunit	100	82	94
MCB1EB_1737	rpmI	Ribosomal protein L20	100	85	97
MCB1EB_1738	infC	Ribosomal protein L35	100	83	100
MCB1EB_1739	thrS	Translation initiation factor 3 (IF-3)	100	88	93
MCB1EB_1740	BgramDRAFT_0571	Threonyl-tRNA synthetase	100	76	99
MCB1EB_1741	sctO	Type III secretory pathway, component EscT	100	51	95
MCB1EB_1742	-	Hypothetical protein	100	34	80
MCB1EB_1743	BSV1_D13	Flagellar biosynthesis/type III secretory pathway ATPase	100	72	98
MCB1EB_1744	A33K_17794	Flagellar biosynthesis/type III secretory pathway protein	-	-	91
MCB1EB_1745	-	Catalase	100	27	-
MCB1EB_1746	bglu_2g02400	Type III secretory pathway, lipoprotein EscJ	100	-	-
MCB1EB_1747	BUPH_00152	Hypothetical protein	100	-	-
MCB1EB_1748	BURK_021220	Hypothetical protein	100	-	-
MCB1EB_1749	-	DNA uptake protein and related DNA-binding proteins	100	-	99
MCB1EB_1750	-	Nucleoside-diphosphate-sugar epimerases	100	76	91
MCB1EB_1751	-	ADP-heptose synthase, bifunctional sugar kinase/adenylyltransferase	100	68	99
MCB1EB_1752	-	Predicted UDP-glucose 6-dehydrogenase	100	73	100
MCB1EB_1753	-	Predicted N-acetylglucosaminyl transferase	100	68	-
MCB1EB_1754	SG0766	Uncharacterized integral membrane protein	100	-	-
MCB1EB_1755	BATDEDRAFT_91794	Uncharacterized conserved protein	100	38	-
MCB1EB_1756	ihfB	Glucoamylase and related glycosyl hydrolases	100	-	-
MCB1EB_1757	rpsA	Bacterial nucleoid DNA-binding protein	100	76	99
MCB1EB_1758	cmk	Ribosomal protein S1	100	85	100
MCB1EB_1759	aroA	Cytidylate kinase	100	67	88
MCB1EB_1760	tyrA	5-enolpyruvylshikimate-3-phosphate synthase	100	65	100
MCB1EB_1761	pheA	Prephenate dehydrogenase	100	57	93

MCB1EB_1762	serC	Prephenate dehydratase	100	67	100
MCB1EB_1763	-	Phosphoserine aminotransferase	100	71	100
MCB1EB_1764	gyrA	Hypothetical protein	100	66	92
MCB1EB_1765	-	Type IIA topoisomerase (DNA gyrase/topo II, topoisomerase IV), A subunit	100	85	99
MCB1EB_1766	ubiG	Outer membrane protein and related peptidoglycan-associated (lipo)proteins	100	69	99
MCB1EB_1767	gph	2-polyprenyl-3-methyl-5-hydroxy-6-metoxy-1,4-benzoquinol methylase	100	72	99
MCB1EB_1768	smc	Predicted phosphatases	100	61	91
MCB1EB_1769	KYC_15302	Cell division protein FtsI/penicillin-binding protein 2	100	-	-
MCB1EB_1770	-	Outer membrane receptor proteins, mostly Fe transport	98	55	94
MCB1EB_1771	LipB	Predicted acetyltransferases and hydrolases with the alpha/beta hydrolase fold	100	-	-
MCB1EB_1772	trmB	Lipase chaperone	85	-	-
MCB1EB_1773	upk	Predicted S-adenosylmethionine-dependent methyltransferase	100	-	-
MCB1EB_1774	BgramDRAFT_3578	Uncharacterized bacitracin resistance protein	100	67	91
MCB1EB_1775	-	Hypothetical protein	100	-	-
MCB1EB_1776	kdtA	Protein-L-isoaspartate carboxylmethyltransferase	100	65	100
MCB1EB_1777	rfaC	3-deoxy-D-manno-octulosonic-acid transferase	100	67	99
MCB1EB_1778	-	ADP-heptose:LPS heptosyltransferase	100	65	100
MCB1EB_1779	-	Phosphomannomutase	100	80	100
MCB1EB_1780	CAGGBEG34_200099	UDP-glucose 4-epimerase	100	72	98
MCB1EB_1781	PanABDRAFT_2554	Predicted glycosyltransferases	100	52	98
MCB1EB_1782		SAM-dependent methyltransferases	100	-	-
MCB1EB_1783	Rta_08890	ABC-type polysaccharide/polyol phosphate transport system, ATPase component	100	41	95
MCB1EB_1784	wbpM	ABC-type polysaccharide/polyol phosphate export systems, permease component	93	33	81
MCB1EB_1785	CAGGBEG34_260027	Predicted nucleoside-diphosphate sugar epimerases	100	71	98
MCB1EB_1786	CAGGBEG34_190057	UDP-N-acetylmuramyl pentapeptide phosphotransferase/UDP-N-acetylglucosamine-1-phosphate transferase	100	54	96
MCB1EB_1787	nrdR	Nucleoside-diphosphate-sugar epimerases	100	53	97
MCB1EB_1788	glyA	Predicted transcriptional regulator, consists of a Zn-ribbon and ATP-cone domains	92	76	98
MCB1EB_1789	-	Glycine/serine hydroxymethyltransferase	100	88	100
MCB1EB_1790	-	Short-chain alcohol dehydrogenase of unknown specificity	100	31	95
MCB1EB_1791	tolR	Biopolymer transport proteins	100	82	97
MCB1EB_1792	Bphy_2454	Biopolymer transport protein	100	52	98
MCB1EB_1793	tolB	Hypothetical protein	100	41	99
MCB1EB_1794	pal	Periplasmic component of the Tol biopolymer transport system	100	67	100
MCB1EB_1795	-	Outer membrane protein and related peptidoglycan-associated (lipo)proteins	100	71	99
MCB1EB_1796	NITGR_280108	Uncharacterized protein conserved in bacteria	100	60	86
MCB1EB_1797	HMPREF9701_05507	Integrase	-	-	-
MCB1EB_1798	NITGR_280106	Hypothetical protein	-	-	-
MCB1EB_1799	-	Hypothetical protein	-	-	-
MCB1EB_1800	Nhal_0976	Superfamily II helicase and inactivated derivatives	-	-	-
MCB1EB_1801	MGMO_44c00340	ABC-type antimicrobial peptide transport system, permease component	-	-	-
MCB1EB_1802	RSc2474	Hypothetical protein	-	-	-
MCB1EB_1803	L810_8411	Hypothetical protein	-	-	-
MCB1EB_1804	OR16_03807	Transcriptional regulator	98	32	87
MCB1EB_1805	LYNGBM3L_24300	Spermidine/putrescine-binding periplasmic protein	99-100	-	-
MCB1EB_1806	-	Predicted NTPase (NACHT family)	66-91	-	-
MCB1EB_1807	-	Hypothetical protein	-	-	-
MCB1EB_1808	istB	Transposase and inactivated derivatives	-	54-55	-
MCB1EB_1809	BCh11DRAFT_03152	DNA replication protein	-	66	-
MCB1EB_1810	-	NAD-dependent aldehyde dehydrogenases	-	-	-
MCB1EB_1811	CAGGBEG34_800003	Hypothetical protein	-	-	-
MCB1EB_1812	-	Uncharacterized conserved protein	-	-	-
MCB1EB_1813	IPF_2632	Bacterial nucleoid DNA-binding protein	-	54	97
MCB1EB_1814	-	FOG: HEAT repeat	-	-	-
MCB1EB_1815	MS53_0328	Predicted NTPase (NACHT family)	66-91	-	-
MCB1EB_1816	-	Permeases of the major facilitator superfamily	-	-	-
MCB1EB_1817	RoseRS_4025	Integrase	-	-	-
MCB1EB_1818	Acife_0758	Predicted NTPase (NACHT family)	44-65	-	-
MCB1EB_1819	-	Hypothetical protein	-	-	-
MCB1EB_1820	BamMC406_2351	Transposase and inactivated derivatives	100	66	-
MCB1EB_1821	SFV_0255	Transposase and inactivated derivatives	-	39	-
MCB1EB_1822	-	Hypothetical protein	99		-
MCB1EB_1823	CAGGBEG34_220024	Hypothetical protein	100	73	99
MCB1EB_1824	CAGGBEG34_330054	Permeases of the major facilitator superfamily	100	54	95
MCB1EB_1825	MC7420_7550	Hypothetical protein	-	-	-
MCB1EB_1826	HMPREF9419_1228	Predicted NTPase (NACHT family)	66-91	-	-
MCB1EB_1827	Hoch_4507	Uncharacterized Fe-S oxidoreductase	98	-	-
MCB1EB_1828	SSDG_00272	NaMN:DMB phosphoribosyltransferase	-	-	-
MCB1EB_1829	-	Hypothetical protein	-	-	-
MCB1EB_1830	-	Transposase and inactivated derivatives	-	-	-
MCB1EB_1831	CARN7_2937	Transposase and inactivated derivatives	100	-	-
MCB1EB_1832	-	Transposase and inactivated derivatives	100	-	-
MCB1EB_1833	FGSG_13479	DNA replication protein	-	-	-
MCB1EB_1834	-	FOG: HEAT repeat	-	-	-
MCB1EB_1835	-	Transposase and inactivated derivatives	-	-	-
MCB1EB_1836	Pse7429DRAFT_2799	Transposase and inactivated derivatives	100	56-66	97
MCB1EB_1837	STIAU_8367	Predicted NTPase (NACHT family)	-	-	-
MCB1EB_1838	-	Hypothetical protein	-	-	-
MCB1EB_1839	CMQ_3643	Transposase and inactivated derivatives	-	-	-
MCB1EB_1840	O203_19195	Hypothetical protein	-	-	-
MCB1EB_1841	O203_19190	Hypothetical protein	75	-	-
MCB1EB_1842	-	Uncharacterized conserved protein	100	-	-
MCB1EB_1843	-	Transposase and inactivated derivatives	97	-	-
MCB1EB_1844	-	Transposase and inactivated derivatives	-	-	-
MCB1EB_1845	-	Transposase and inactivated derivatives	-	-	-
MCB1EB_1846	-	Transposase and inactivated derivatives	-	-	-
MCB1EB_1847	-	Transposase and inactivated derivatives	-	-	-
MCB1EB_1848	LYNGBM3L_24300	DNA replication protein	-	-	-
MCB1EB_1849	-	Predicted NTPase (NACHT family)	66-91	-	-
MCB1EB_1850	-	Predicted NTPase (NACHT family)	66-91	-	-
MCB1EB_1851	P9215_14271	Transposase and inactivated derivatives	100	-	-
MCB1EB_1852	metB	Methyl-accepting chemotaxis protein	79	-	-
MCB1EB_1853	purF	Cystathionine beta-lyases/cystathionine gamma-synthases	100	73	98
MCB1EB_1854	-	Glutamine phosphoribosylpyrophosphate amidotransferase	100	79	100
MCB1EB_1855	P355_3030	Uncharacterized membrane protein, required for colicin V production	100	52	97
MCB1EB_1856	folC	Uncharacterized protein conserved in bacteria	100	54	18
MCB1EB_1857	accD	Folylpolyglutamate synthase	100	71	100
MCB1EB_1858	trpA	Acetyl-CoA carboxylase beta subunit	100	86	99
MCB1EB_1859	trpB	Tryptophan synthase alpha chain	92	71	97
MCB1EB_1860	-	Tryptophan synthase beta chain	100	83	100
MCB1EB_1861	truA	Phosphoribosylanthranilate isomerase	100	59	93

MCB1EB_1862	asd	Pseudouridylate synthase	100	70	100
MCB1EB_1863	leuB1	Aspartate-semialdehyde dehydrogenase	100	72	98
MCB1EB_1864	gltA	Isocitrate/isopropylmalate dehydrogenase	100	79	99
MCB1EB_1865	-	Citrate synthase	100	88	100
MCB1EB_1866	sdhB	Uncharacterized conserved protein	100	71	97
MCB1EB_1867	sdhA	Succinate dehydrogenase/fumarate reductase, Fe-S protein subunit	100	75	50
MCB1EB_1868	-	Succinate dehydrogenase/fumarate reductase, flavoprotein subunit	100	86	100
MCB1EB_1869	EUOG_04987	Succinate dehydrogenase, hydrophobic anchor subunit	100	73	100
MCB1EB_1870	SG0766	Uncharacterized conserved protein	-	-	-
MCB1EB_1871	-	Uncharacterized conserved protein	98-100	38-39	-
MCB1EB_1872	mdh	Transcriptional regulators	100	76	89
MCB1EB_1873	RBRH_00165	Malate/lactate dehydrogenases	100	79	100
MCB1EB_1874	acnA	Hypothetical protein	100	-	-
MCB1EB_1875	thrC	Aconitase A	100	77	100
MCB1EB_1876	thrA	Threonine synthase	100	71	100
MCB1EB_1877	-	Homoserine dehydrogenase	100	70	99
MCB1EB_1878	aroE	Na^+/citrate symporter	91-94	54-56	-
MCB1EB_1879	-	Shikimate 5-dehydrogenase	100	64	96
MCB1EB_1880	mpl	Exoribonuclease R	100	66	100
MCB1EB_1881	pYS10087	UDP-N-acetylmuramate-alanine ligase	100	73	97
MCB1EB_1882	Y69_0155	Cation transport ATPase	-	-	-
MCB1EB_1883	K788_3090	NhaP-type Na^+/H^+ and K^+/H^+ antiporters	-	-	-
MCB1EB_1884	K788_3091	Uncharacterized protein conserved in bacteria	100	-	-
MCB1EB_1885	-	Uncharacterized protein conserved in bacteria	100	-	-
MCB1EB_1886	rnc	Uncharacterized protein conserved in bacteria	100	-	-
MCB1EB_1887	PMI06_000449	Integrase	100	-	-
MCB1EB_1888	-	Uncharacterized protein conserved in bacteria	100	-	-
MCB1EB_1889	-	Hypothetical protein	100	-	-
MCB1EB_1890	WQE_10019	ATPases with chaperone activity, ATP-binding subunit	100	-	-
MCB1EB_1891	WQE_10014	Uncharacterized protein conserved in bacteria	100	-	-
MCB1EB_1892	WQE_10009	Uncharacterized protein conserved in bacteria	100	-	-
MCB1EB_1893	CFII68_20828	Uncharacterized protein conserved in bacteria	100	-	-
MCB1EB_1894	tssC-2	Hemolysin-coregulated protein (uncharacterized)	100	-	-
MCB1EB_1895	-	Uncharacterized protein conserved in bacteria	100	-	-
MCB1EB_1896	K788_3104	Uncharacterized protein conserved in bacteria	100	-	-
MCB1EB_1897	BC1002_2680	Uncharacterized protein conserved in bacteria	100	-	-
MCB1EB_1898	aroQ	Thiol-disulfide isomerase and thioredoxins	100	-	-
MCB1EB_1899	bccP	3-dehydroquinate dehydratase II	100	62	98
MCB1EB_1900	accC	Biotin carboxyl carrier protein	100	74	100
MCB1EB_1901	prmA	Biotin carboxylase	100	89	100
MCB1EB_1902	tpx	Ribosomal protein L11 methylase	100	62	97
MCB1EB_1903	-	Peroxiredoxin	100	72	100
MCB1EB_1904	-	Sugar kinases, ribokinase family	100	66	100
MCB1EB_1905	LOC431817	Outer membrane lipoprotein	100	64	97
MCB1EB_1906	nrdB	Hypothetical protein	100	-	-
MCB1EB_1907	nrdA	Ribonucleotide reductase, beta subunit	100	82	100
MCB1EB_1908	ampD	Ribonucleotide reductase, alpha subunit	100	82	96
MCB1EB_1909	-	Negative regulator of beta-lactamase expression	100	61	-
MCB1EB_1910	ffh	ABC-type uncharacterized transport system, permease component	100	60	100
MCB1EB_1911	RoseRS_4025	Signal recognition particle GTPase	100	78	100
MCB1EB_1912	-	Predicted NTPase (NACHT family)	-	-	-
MCB1EB_1913	proS	Multiple antibiotic transporter	100	70	99
MCB1EB_1914	nudH	Prolyl-tRNA synthetase	100	74	100
MCB1EB_1915	proB	NTP pyrophosphohydrolases including oxidative damage repair enzymes	100	-	-
MCB1EB_1916	obgE	Glutamate 5-kinase	100	81	96
MCB1EB_1917	rpmA	Predicted GTPase	100	81	99
MCB1EB_1918	rplU	Ribosomal protein L27	100	86	99
MCB1EB_1919	-	Ribosomal protein L21	100	88	99
MCB1EB_1920	-	Cytochrome c553	98	72	84
MCB1EB_1921	-	Cytochrome c, mono- and diheme variants	100	77	90
MCB1EB_1922	miaB	Hypothetical protein	100	70	99
MCB1EB_1923	-	2-methylthioadenine synthetase	100	-	-
MCB1EB_1924	-	Predicted metal-dependent hydrolase	100	62	99
MCB1EB_1925	-	Uncharacterized protein involved in cation transport	100	61	94
MCB1EB_1926	lnt	Putative Mg^{2+} and Co^{2+} transporter CorC	100	80	100
MCB1EB_1927	COXBURSA334_1537	Apolipoprotein N-acyltransferase	100	58	93
MCB1EB_1928	glyQ	Hypothetical protein	100	-	-
MCB1EB_1929	glyS	Glycyl-tRNA synthetase, alpha subunit	100	-	-
MCB1EB_1930	gmhB	Glycyl-tRNA synthetase, beta subunit	100	65	97
MCB1EB_1931	-	Histidinol phosphatase and related phosphatases	100	-	-
MCB1EB_1932	ksgA	1-acyl-sn-glycerol-3-phosphate acyltransferase	82	-	94
MCB1EB_1933	pdxA	Dimethyladenosine transferase (rRNA methylation)	100	69	97
MCB1EB_1934	surA	Pyridoxal phosphate biosynthesis protein	100	-	-
MCB1EB_1935	-	Parvulin-like peptidyl-prolyl isomerase	100	-	-
MCB1EB_1936	-	Organic solvent tolerance protein OstA	100	59	100
MCB1EB_1937	-	Predicted phosphotransferase related to Ser/Thr protein kinases	100	-	-
MCB1EB_1938	-	Predicted acetyltransferase	-	-	-
MCB1EB_1939	A33K_14291	Nucleoside-diphosphate-sugar pyrophosphorylase involved in lipopolysaccharide biosynthesis/translation initiation factor 2B, gamma/epsilon subunits (eIF-2Bgamma/eIF-2Bepsilon)	100	-	98
MCB1EB_1940	fis	2-polyprenyl-6-methoxyphenol hydroxylase and related FAD-dependent oxidoreductases	100	54	100
MCB1EB_1941	purH	Factor for inversion stimulation Fis, transcriptional activator	100	65	100
MCB1EB_1942	ruvC	AICAR transformylase/IMP cyclohydrolase PurH (only IMP cyclohydrolase domain in Aful)	100	75	99
MCB1EB_1943	ruvA	Holliday junction resolvasome, endonuclease subunit	100	75	99
MCB1EB_1944	ruvB	Holliday junction resolvasome, DNA-binding subunit	100	79	99
MCB1EB_1945	tyrS	Holliday junction resolvasome, helicase subunit	100	84	97
MCB1EB_1946	anmK	Tyrosyl-tRNA synthetase	100	83	-
MCB1EB_1947	gcvP	Predicted molecular chaperone distantly related to HSP70-fold metalloproteases	100	-	98
MCB1EB_1948	-	Glycine cleavage system protein P (pyridoxal-binding), C-terminal domain	100	72	100
MCB1EB_1949	gcvT	Glycine cleavage system H protein (lipoate-binding)	99	62	99
MCB1EB_1950	AXYL_06795	Glycine cleavage system T protein (aminomethyltransferase)	93	66	88
MCB1EB_1951	-	Putative NADP-dependent oxidoreductases	-	-	-
MCB1EB_1952	-	Predicted glutamine amidotransferases	100	77	-
MCB1EB_1953	RoseRS_4025	Kef-type K+ transport systems, membrane components	100	-	-
MCB1EB_1954	elbB	Predicted NTPase (NACHT family)	70-100	-	-
MCB1EB_1955	-	Uncharacterized protein involved in an early stage of isoprenoid biosynthesis	100	-	-
MCB1EB_1956	BURMUCGD1_0587	Uncharacterized protein conserved in bacteria	100	-	-
MCB1EB_1957	-	Phospholipase/lecithinase/hemolysin	85	47	88
MCB1EB_1958	WQE_13621	D-alanyl-D-alanine carboxypeptidase (penicillin-binding protein 4)	96	56	91
MCB1EB_1959	-	Hypothetical protein	100	42	95
MCB1EB_1960	Haur_3755	Putative translation factor (SUA5)	100	61	90
MCB1EB_1961	-	3-methyladenine DNA glycosylase/8-oxoguanine DNA glycosylase	100	-	-

MCB1EB_1962	RoseRS_4025	Methylated DNA-protein cysteine methyltransferase	100	-	-
MCB1EB_1963	adk	Predicted NTPase (NACHT family)	-	-	-
MCB1EB_1964	SG0735	ATPases with chaperone activity, ATP-binding subunit	100	-	-
MCB1EB_1965	purK	Uncharacterized conserved protein	96	33-34	94
MCB1EB_1966	purE	Phosphoribosylaminoimidazole carboxylase (NCAIR synthetase)	100	66	96
MCB1EB_1967	purC	Phosphoribosylcarboxyaminoimidazole (NCAIR) mutase	100	73	96
MCB1EB_1968	fbaA	Phosphoribosylaminoimidazolesuccinocarboxamide (SAICAR) synthase	100	77	99
MCB1EB_1969	pgk	Fructose/tagatose bisphosphate aldolase	100	85	100
MCB1EB_1970	-	3-phosphoglycerate kinase	100	74	99
MCB1EB_1971	rfaF	Branched-chain amino acid aminotransferase/4-amino-4-deoxychorismate lyase	100	78	100
MCB1EB_1972	BRPE64_ACDS04830	ADP-heptose:LPS heptosyltransferase	100	68	100
MCB1EB_1973	-	Hypothetical protein	100	48	100
MCB1EB_1974	RoseRS_4025	Uncharacterized protein conserved in bacteria	100	-	-
MCB1EB_1975	CPAR2_208840	Predicted NTPase (NACHT family)	70-100	-	-
MCB1EB_1976	-	Hypothetical protein	76	-	-
MCB1EB_1977	-	Hypothetical protein	-	-	-
MCB1EB_1978	-	Tfp pilus assembly protein, major pilin PilA	100	62	85
MCB1EB_1979	sucD	Membrane protein TerC, possibly involved in tellurium resistance	100	-	-
MCB1EB_1980	sucC	Succinyl-CoA synthetase, alpha subunit	100	89	100
MCB1EB_1981	Bcep1808_2762	Succinyl-CoA synthetase, beta subunit	100	-	-
MCB1EB_1982	recA	Uncharacterized protein conserved in bacteria	100	51	97
MCB1EB_1983	-	RecA/RadA recombinase	100	91	97
MCB1EB_1984	BURMUCGD1_2804	Cytochrome B561	100	33	95
MCB1EB_1985	-	Uncharacterized paraquat-inducible protein A	80	-	-
MCB1EB_1986	-	Uncharacterized paraquat-inducible protein A	100	-	-
MCB1EB_1987	CAGGBEG34_290002	Paraquat-inducible protein B	100	64	96
MCB1EB_1988	Moth_0730	Uncharacterized protein conserved in bacteria	100	43	68
MCB1EB_1989	-	Hypothetical protein	100	-	-
MCB1EB_1990	mnmA	Adenylosuccinate lyase	100	82	98
MCB1EB_1991	BDB_160006	Predicted tRNA(5-methylaminomethyl-2-thiouridylate) methyltransferase, contains the PP-loop ATPase domain	100	81	99
MCB1EB_1992	-	Hypothetical protein	100	-	-
MCB1EB_1993	-	NAD/NADP transhydrogenase alpha subunit	100	-	-
MCB1EB_1994	-	NAD/NADP transhydrogenase alpha subunit	100	-	-
MCB1EB_1995	-	NAD/NADP transhydrogenase beta subunit	100	-	-
MCB1EB_1996	-	Predicted N6-adenine-specific DNA methylase	100	-	-
MCB1EB_1997	-	Gamma-aminobutyrate permease and related permeases	92	35	-
MCB1EB_1998	-	Lysine 2,3-aminomutase	100	79	94
MCB1EB_1999	proA	Predicted membrane protein	100	70	-
MCB1EB_2000	holA	Gamma-glutamyl phosphate reductase	100	73	100
MCB1EB_2001	BYI23_A022000	DNA polymerase III, delta subunit	100	67	100
MCB1EB_2002	leuS	Rare lipoprotein B	100	49	99
MCB1EB_2003	dapB	Leucyl-tRNA synthetase	100	72	100
MCB1EB_2004	-	Dihydrodipicolinate reductase	100	70	99
MCB1EB_2005	fur1	Small protein A (tmRNA-binding)	100	-	-
MCB1EB_2006	xopK	Fe2+/Zn2+ uptake regulation proteins	100	82	99
MCB1EB_2007	cbbG2	Hypothetical protein	92	-	-
MCB1EB_2008	tktA	Glyceraldehyde-3-phosphate dehydrogenase/erythrose-4-phosphate dehydrogenase	100	-	-
MCB1EB_2009	-	Transketolase	100	77	99
MCB1EB_2010	rpsI	Uncharacterized conserved protein	100	80	98
MCB1EB_2011	rplM	Ribosomal protein S9	100	88	100
MCB1EB_2012	recG	Ribosomal protein L13	100	85	100
MCB1EB_2013	queA	RecG-like helicase	100	71	98
MCB1EB_2014	tgt	S-adenosylmethionine:tRNA-ribosyltransferase-isomerase (queuine synthetase)	100	71	99
MCB1EB_2015	yajC	Queuine/archaeosine tRNA-ribosyltransferase	100	79	100
MCB1EB_2016	secD	Preprotein translocase subunit YajC	100	-	-
MCB1EB_2017	secF	Preprotein translocase subunit SecD	100	78	98
MCB1EB_2018	A33K_15475	Preprotein translocase subunit SecF	100	83	100
MCB1EB_2019	Bcep18194_B0308	Transcriptional regulator	-	27	-
MCB1EB_2020	K788_4302	Na+/citrate symporter	92-97	46-55	-
MCB1EB_2021	H480_14512	Na+/citrate symporter	-	-	-
MCB1EB_2022	secF	Mg/Co/Ni transporter MgtE (contains CBS domain)	-	-	-
MCB1EB_2023	GUITHDRAFT_53559	Preprotein translocase subunit SecF	-	-	-
MCB1EB_2024	RSc2452	ABC-type dipeptide transport system, periplasmic component	99	-	-
MCB1EB_2025	-	Phosphatidylserine/phosphatidylglycerophosphate/cardiolipin synthases and related enzymes	100	33	96
MCB1EB_2026	purN	tRNA and rRNA cytosine-C5-methylases	100	72	99
MCB1EB_2027	ribF	Folate-dependent phosphoribosylglycinamide formyltransferase PurN	100	65	100
MCB1EB_2028	ileS	FAD synthase	100	66	96
MCB1EB_2029	lspA	Isoleucyl-tRNA synthetase	97	76	99
MCB1EB_2030	dfp	Lipoprotein signal peptidase	100	70	99
MCB1EB_2031	dut	Phosphopantothenoylcysteine synthetase/decarboxylase	100	75	99
MCB1EB_2032	N9414_09841	dUTPase	100	83	100
MCB1EB_2033	Cenrod_0254	FOG: WD40 repeat	100	-	-
MCB1EB_2034	Gura_2104	Predicted NTPase (NACHT family)	100	-	-
MCB1EB_2035	lpxC	Uncharacterized conserved protein	100	-	-
MCB1EB_2036	ftsZ	UDP-3-O-acyl-N-acetylglucosamine deacetylase	100	86	100
MCB1EB_2037	ftsA	Cell division GTPase	100	86	100
MCB1EB_2038	-	Actin-like ATPase involved in cell division	100	91	100
MCB1EB_2039	-	Cell division septal protein	100	61	99
MCB1EB_2040	murC	D-alanine-D-alanine ligase and related ATP-grasp enzymes	100	64	100
MCB1EB_2041	murG	UDP-N-acetylmuramate-alanine ligase	100	74	99
MCB1EB_2042	ftsW	UDP-N-acetylglucosamine:LPS N-acetylglucosamine transferase	100	67	98
MCB1EB_2043	murD	Bacterial cell division membrane protein	100	79	100
MCB1EB_2044	mraY	UDP-N-acetylmuramoylalanine-D-glutamate ligase	100	62	100
MCB1EB_2045	-	UDP-N-acetylmuramyl pentapeptide phosphotransferase/UDP-N-acetylglucosamine-1-phosphate transferase	100	85	100
MCB1EB_2046	murE	UDP-N-acetylmuramyl pentapeptide synthase	100	64	97
MCB1EB_2047	ftsI	UDP-N-acetylmuramyl tripeptide synthase	100	58	96
MCB1EB_2048	-	Cell division protein FtsI/penicillin-binding protein 2	100	70	96
MCB1EB_2049	mraW	Cell division protein	100	-	-
MCB1EB_2050	mraZ	Predicted S-adenosylmethionine-dependent methyltransferase involved in cell envelope biogenesis	100	69	98
MCB1EB_2051	PHAVU_006G124200g	Uncharacterized protein conserved in bacteria	100	79	100
MCB1EB_2052	Psta_3938	Site-specific DNA methylase	-	-	-
MCB1EB_2053	CLOSYM_00639	Cobyric acid synthase	-	-	-
MCB1EB_2054	-	Hypothetical protein	82	-	-
MCB1EB_2055	RBRH_03270	Ubiquinone biosynthesis protein COQ7	100	66	100
MCB1EB_2056	fadD1	Outer membrane protein (porin)	100	59	100
MCB1EB_2057	phoU	Acyl-CoA synthetases (AMP-forming)/AMP-acid ligases II	99	69	100
MCB1EB_2058	pstB	Phosphate uptake regulator	100	73	100

MCB1EB_2059	pstA	ABC-type phosphate transport system, ATPase component	100	85	96
MCB1EB_2060	pstC	ABC-type phosphate transport system, permease component	66	77	29
MCB1EB_2061	-	ABC-type phosphate transport system, permease component	100	73	56
MCB1EB_2062	Bcep1808_2620	ABC-type phosphate transport system, periplasmic component	100	73	99
MCB1EB_2063	-	Transposase and inactivated derivatives, IS5 family	99	-	-
MCB1EB_2064	ECDEC2D_3483	Transposase and inactivated derivatives, IS5 family	100	-	-
MCB1EB_2065	RO3G_00984	FOG: Ankyrin repeat	100	-	-
MCB1EB_2066	glmM	FOG: WD40 repeat	100	-	-
MCB1EB_2067	folP	Phosphomannomutase	100	78	100
MCB1EB_2068	ftsH	Dihydropteroate synthase and related enzymes	100	66	96
MCB1EB_2069	rrmJ	ATP-dependent Zn proteases	100	85	98
MCB1EB_2070	-	23S rRNA methylase	100	77	99
MCB1EB_2071	greA	Predicted RNA-binding protein containing KH domain, possibly ribosomal protein	100	53	100
MCB1EB_2072	carB	Transcription elongation factor	100	80	99
MCB1EB_2073	carA	Carbamoylphosphate synthase large subunit (split gene in MJ)	100	-	-
MCB1EB_2074	-	Carbamoylphosphate synthase small subunit	100	76	97
MCB1EB_2075	-	Ribonuclease HI	99	76	96
MCB1EB_2076	PRU_2073	SAM-dependent methyltransferases	100	62	99
MCB1EB_2077	-	Permeases of the major facilitator superfamily	-	-	-
MCB1EB_2078	dnaQ	Soluble lytic murein transglycosylase and related regulatory proteins (some contain LysM/invasin domains)	86	-	77
MCB1EB_2079	Hipma_1707	DNA polymerase III, epsilon subunit and related 3'-5' exonucleases	100	72	98
MCB1EB_2080	trwL	Uncharacterized protein conserved in bacteria	100	-	-
MCB1EB_2081	trwK	Lysine/ornithine N-monooxygenase	100	-	-
MCB1EB_2082	trwJ	Type IV secretory pathway, VirB4 components	100	-	-
MCB1EB_2083	trwI	Hypothetical protein	100	-	-
MCB1EB_2084	trwG	Type IV secretory pathway, VirB6 components	40	-	-
MCB1EB_2085	Q652_00727	Type IV secretory pathway, component VirB8	100	-	-
MCB1EB_2086	-	Type IV secretory pathway, VirB9 components	98	-	-
MCB1EB_2087	trwD	Type IV secretory pathway, VirB10 components	100	-	-
MCB1EB_2088	pyrC	Type IV secretory pathway, VirB11 components, and related ATPases involved in archaeal flagella biosynthesis	100	-	-
MCB1EB_2089	-	Dihydroorotase	100	77	95
MCB1EB_2090	-	ABC-type branched-chain amino acid transport systems, ATPase component	95	76	99
MCB1EB_2091	-	ABC-type branched-chain amino acid transport systems, ATPase component	100	81	97
MCB1EB_2092	-	ABC-type branched-chain amino acid transport system, permease component	96	77	100
MCB1EB_2093	ispH	Branched-chain amino acid ABC-type transport system, permease components	100	80	100
MCB1EB_2094	fkpB	Penicillin tolerance protein	99	76	96
MCB1EB_2095	-	FKBP-type peptidyl-prolyl cis-trans isomerases 2	100	76	100
MCB1EB_2096	-	DNA repair proteins	100	56	95
MCB1EB_2097	rpmG	Ribosomal protein L28	100	86	99
MCB1EB_2098	nadB	Ribosomal protein L33	100	80	100
MCB1EB_2099	-	Aspartate oxidase	100	77	98
MCB1EB_2100	rfbD	Predicted glycosyltransferases	100	-	-
MCB1EB_2101	-	dTDP-4-dehydrorhamnose reductase	100	22	99
MCB1EB_2102	-	dTDP-4-dehydrorhamnose 3,5-epimerase and related enzymes	100	63	100
MCB1EB_2103	-	dTDP-glucose pyrophosphorylase	100	80	100
MCB1EB_2104	-	dTDP-D-glucose 4,6-dehydratase	100	81	-
MCB1EB_2105	nadC	Glycosyltransferases involved in cell wall biogenesis	100	-	-
MCB1EB_2106	nadA	Nicotinate-nucleotide pyrophosphorylase	100	79	98
MCB1EB_2107	-	Quinolinate synthase	100	70	98
MCB1EB_2108	-	Fatty-acid desaturase	100	78	100
MCB1EB_2109	coaD	Ferredoxin	100	75	99
MCB1EB_2110	-	Phosphopantetheine adenylyltransferase	100	75	89
MCB1EB_2111	C266_05194	N6-adenine-specific methylase	100	84	88
MCB1EB_2112	U875_21020	Predicted Zn-dependent peptidases	100	-	-
MCB1EB_2113	ftsY	Predicted Zn-dependent peptidases	95	-	-
MCB1EB_2114	-	Signal recognition particle GTPase	100	-	-
MCB1EB_2115	RF_1288	2-keto-4-pentenoate hydratase/2-oxohepta-3-ene-1,7-dioic acid hydratase (catechol pathway)	100	61	100
MCB1EB_2116	SMUL_0469	Hypothetical protein	100	-	-
MCB1EB_2117	hspC	Predicted transcriptional regulators	100	-	-
MCB1EB_2118	rpoH	Molecular chaperone (small heat shock protein)	100	-	-
MCB1EB_2119	BN480_00009	DNA-directed RNA polymerase, sigma subunit (sigma70/sigma32)	100	59	92
MCB1EB_2120	clpA	Hypothetical protein	100	-	-
MCB1EB_2121	clpS	ATPases with chaperone activity, ATP-binding subunit	100	78	100
MCB1EB_2122	-	Uncharacterized conserved protein	100	85	98
MCB1EB_2123	-	N-acetylglucosamine-6-phosphate deacetylase	100	-	-
MCB1EB_2124	cyoE	Uncharacterized protein SCO1/SenC/PrrC, involved in biogenesis of respiratory and photosynthetic systems	78	-	100
MCB1EB_2125	ctaA	Polyprenyltransferase (cytochrome oxidase assembly factor)	100	69	98
MCB1EB_2126	Bphyt_0552	Uncharacterized protein required for cytochrome oxidase assembly	100	74	97
MCB1EB_2127	-	Hypothetical protein	100	64	89
MCB1EB_2128	HMPREF9123_2316	Uncharacterized conserved protein	100	59	98
MCB1EB_2129	coxC	Hypothetical protein	-	-	-
MCB1EB_2130	-	Heme/copper-type cytochrome/quinol oxidase, subunit 3	100	66	100
MCB1EB_2131	coxB	Heme/copper-type cytochrome/quinol oxidases, subunit 1	100	77	100
MCB1EB_2132	bioC	Heme/copper-type cytochrome/quinol oxidases, subunit 2	100	85	91
MCB1EB_2133	CAGGBEG34_180067	SAM-dependent methyltransferases	100	71	98
MCB1EB_2134	yibK	Predicted amidophosphoribosyltransferases	78	-	-
MCB1EB_2135	gpsA	Predicted rRNA methylase (SpoU class)	100	56	100
MCB1EB_2136	secB	Glycerol-3-phosphate dehydrogenase	100	67	98
MCB1EB_2137	grxC	Preprotein translocase subunit SecB	100	70	78
MCB1EB_2138	-	Glutaredoxin and related proteins	100	69	95
MCB1EB_2139	gpmA	Rhodanese-related sulfurtransferase	87	-	67
MCB1EB_2140	-	Phosphoglycerate mutase 1	100	75	92
MCB1EB_2141	thiF	Periplasmic protease	100	-	-
MCB1EB_2142	-	Dinucleotide-utilizing enzymes involved in molybdopterin and thiamine biosynthesis family 2	100	65	99
MCB1EB_2143	icd	Cold shock proteins	100	-	-
MCB1EB_2144	Naga_100008g11	Isocitrate dehydrogenases	100	87	100
MCB1EB_2145	-	Hypothetical protein	100	-	-
MCB1EB_2146	sodB	Uncharacterized conserved protein	-	-	-
MCB1EB_2147	xseA	Superoxide dismutase	100	83	99
MCB1EB_2148	lpxK	Exonuclease VII, large subunit	100	60	97
MCB1EB_2149	-	Tetraacyldisaccharide-1-P 4'-kinase	100	-	-
MCB1EB_2150	kdsB	Uncharacterized conserved protein	100	67	96
MCB1EB_2151	adk	CMP-2-keto-3-deoxyoctulosonic acid synthetase	100	63	90
MCB1EB_2152	mviN	Adenylate kinase and related kinases	100	72	100
MCB1EB_2153	rpsN	Uncharacterized membrane protein, putative virulence factor	100	73	100
MCB1EB_2154	-	Ribosomal protein S20	100	81	96

MCB1EB_2155	argF	Hypothetical protein	100	-	-
MCB1EB_2156	-	Ornithine carbamoyltransferase	100	81	100
MCB1EB_2157	murB	Argininosuccinate synthase	100	29	85
MCB1EB_2158	-	UDP-N-acetylmuramate dehydrogenase	100	57	99
MCB1EB_2159	h16_A0588	Uncharacterized protein conserved in bacteria	100	78	100
MCB1EB_2160	WQE_06227	Predicted membrane protein	100	66	98
MCB1EB_2161	ppa	Acyl-CoA synthetases (AMP-forming)/AMP-acid ligases II	100	-	-
MCB1EB_2162	xerD	Inorganic pyrophosphatase	100	82	100
MCB1EB_2163	ogt	Site-specific recombinase XerD	100	73	95
MCB1EB_2164	HMPREF9718_00701	Methylated DNA-protein cysteine methyltransferase	100	63	90
MCB1EB_2165	-	Hypothetical protein	100	-	-
MCB1EB_2166	bgla_1g32700	Uncharacterized Fe-S protein	100	68	96
MCB1EB_2167	amiA	Predicted ATPase or kinase	100	59	95
MCB1EB_2168	dedA	N-acetylmuramoyl-L-alanine amidase	99	62	100
MCB1EB_2169	mutL	Uncharacterized membrane-associated protein	100	69	100
MCB1EB_2170	miaA	DNA mismatch repair enzyme (predicted ATPase)	100	65	-
MCB1EB_2171	purM	tRNA delta(2)-isopentenylpyrophosphate transferase	100	59	-
MCB1EB_2172	-	Phosphoribosylaminoimidazole (AIR) synthetase	100	75	99
MCB1EB_2173	-	ATPase involved in DNA replication initiation	100	58	94
MCB1EB_2174	pcnB	Phosphoserine phosphatase	100	67	100
MCB1EB_2175	panB	tRNA nucleotidyltransferase/poly(A) polymerase	100	71	94
MCB1EB_2176	-	Ketopantoate hydroxymethyltransferase	100	-	98
MCB1EB_2177	-	DNA-binding protein H-NS	100	-	52
MCB1EB_2178	WQE_15091	ABC-type branched-chain amino acid transport systems, periplasmic component	100	72	99
MCB1EB_2179	-	Hypothetical protein	85	-	87
MCB1EB_2180	-	Metal-dependent hydrolase	100	-	95
MCB1EB_2181	CAGGBEG34_220056	Flavodoxin reductases (ferredoxin-NADPH reductases) family 1	100	67	93
MCB1EB_2182	-	Flagellar biogenesis protein	99	80	-
MCB1EB_2183	-	Di- and tricarboxylate transporters	100	-	-
MCB1EB_2184	appB	Cytochrome bd-type quinol oxidase, subunit 1	100	69	100
MCB1EB_2185	yrohd0001_33870	Cytochrome bd-type quinol oxidase, subunit 2	100	63	100
MCB1EB_2186	-	Hypothetical protein	100	-	-
MCB1EB_2187	WQE_14941	Protein involved in biosynthesis of mitomycin antibiotics/polyketide fumonisin	100	63	98
MCB1EB_2188	BH160DRAFT_0871	Formamidopyrimidine-DNA glycosylase	100	-	-
MCB1EB_2189	WQE_29953	Predicted N-acetylglucosamine kinase	100	-	-
MCB1EB_2190	serA	DNA-binding protein H-NS	100	-	-
MCB1EB_2191	YN1551_2013	Phosphoglycerate dehydrogenase and related dehydrogenases	100	76	95
MCB1EB_2192	leuD	FOG: Ankyrin repeat	100	-	-
MCB1EB_2193	leuC	3-isopropylmalate dehydratase small subunit	100	75	100
MCB1EB_2194	ptsI	3-isopropylmalate dehydratase large subunit	100	81	100
MCB1EB_2195	pstH	Phosphoenolpyruvate-protein kinase (PTS system EI component in bacteria)	100	75	100
MCB1EB_2196	-	Phosphotransferase system, HPr-related proteins	100	74	99
MCB1EB_2197	-	Phosphotransferase system, mannose/fructose-specific component IIA	100	66	90
MCB1EB_2198	-	Nitrogen regulatory protein PII	100	87	100
MCB1EB_2199	-	Uncharacterized protein conserved in bacteria	100	59	99
MCB1EB_2200	-	Predicted ATPase with chaperone activity	100	65	99
MCB1EB_2201	-	Thiol-disulfide isomerase and thioredoxins	99	-	-
MCB1EB_2202	-	Uncharacterized conserved protein	-	-	-
MCB1EB_2203	-	small molecule metabolism	100	84	-
MCB1EB_2204	metHa	Hypothetical protein	100	66	100
MCB1EB_2205	metHb	Methionine synthase I (cobalamin-dependent), methyltransferase domain	100	82	100
MCB1EB_2206	-	Methionine synthase I, cobalamin-binding domain	100	75	99
MCB1EB_2207	-	Carbonic anhydrase	100	-	98
MCB1EB_2208	BgramDRAFT_1585	ABC-type dipeptide transport system, periplasmic component	100	64	100
MCB1EB_2209	K788_4134	Thiol-disulfide isomerase and thioredoxins	100	54	91
MCB1EB_2210	argS	Cell division protein	100	46	97
MCB1EB_2211	WQE_03647	Arginyl-tRNA synthetase	100	70	100
MCB1EB_2212	N234_15805	Hypothetical protein	100	59	100
MCB1EB_2213	-	Transposase and inactivated derivatives	-	-	-
MCB1EB_2214	Nit79A3_0051	Hypothetical protein	100	67	98
MCB1EB_2215	-	Aspartokinases	-	-	-
MCB1EB_2216	tccC3	Transposase and inactivated derivatives, TnpA family	-	-	-
MCB1EB_2217	tccC3	Rhs family protein	-	-	-
MCB1EB_2218	tccC3	Rhs family protein	-	-	-
MCB1EB_2219	-	Rhs family protein	-	-	-
MCB1EB_2220	PCH70_41490	Hypothetical protein	-	46	-
MCB1EB_2221	tcaA2	Glyceraldehyde-3-phosphate dehydrogenase/erythrose-4-phosphate dehydrogenase	-	-	-
MCB1EB_2222	LYNGBM3L_24300	Hypothetical protein	-	29	-
MCB1EB_2223	-	Predicted NTPase (NACHT family)	66-91	-	-
MCB1EB_2224	PMI28_00004	Growth inhibitor	-	34-37	-
MCB1EB_2225	RPL7	Growth regulator	-	-	-
MCB1EB_2226	-	Small-conductance mechanosensitive channel	-	-	-
MCB1EB_2227	Halhy_2265	Transposase and inactivated derivatives, TnpA family	-	-	-
MCB1EB_2228	NH44784_027271	Predicted NTPase (NACHT family)	66-91	-	-
MCB1EB_2229	CRE_18979	Spermidine/putrescine-binding periplasmic protein	-	-	-
MCB1EB_2230	L810_8411	Undecaprenyl pyrophosphate synthase	-	-	-
MCB1EB_2231	RSc2474	Transcriptional regulator	98	31	87
MCB1EB_2232	MGMO_44c00340	Hypothetical protein	-	-	-
MCB1EB_2233	SKA53_10894	Hypothetical protein	-	-	-
MCB1EB_2234	-	Hypothetical protein	-	-	-
MCB1EB_2235	-	Superfamily II helicase and inactivated derivatives	-	-	-
MCB1EB_2236	YO5_17850	Predicted transcriptional regulator	-	-	-
MCB1EB_2237		Hypothetical protein	-	-	-
MCB1EB_2238	trmE	Integrase	-	-	-
MCB1EB_2239	phrB	Predicted GTPase	100	74	100
MCB1EB_2240	LEP1GSC044_1389	Deoxyribodipyrimidine photolyase	100	38	96
MCB1EB_2241	-	FOG: Ankyrin repeat	-	-	-
MCB1EB_2242	-	Transposase and inactivated derivatives, IS5 family	-	82	-
MCB1EB_2243	-	FOG: Ankyrin repeat	-	-	-
MCB1EB_2244	-	Transposase and inactivated derivatives, IS5 family	-	82	-
MCB1EB_2245	-	ADP-heptose synthase, bifunctional sugar kinase/adenylyltransferase	100	72	98
MCB1EB_2246	BC1001_0218	Putative transcriptional regulator, homolog of Bvg accessory factor	100	51	96
MCB1EB_2247	dppB	Biotin-(acetyl-CoA carboxylase) ligase	100	51	98
MCB1EB_2248	-	ABC-type dipeptide/oligopeptide/nickel transport systems, permease components	100	77	100
MCB1EB_2249	-	ABC-type dipeptide/oligopeptide/nickel transport systems, permease components	100	76	97
MCB1EB_2250	dppF	ABC-type dipeptide/oligopeptide/nickel transport system, ATPase component	94	72	96
MCB1EB_2251	OR16_03807	ABC-type oligopeptide transport system, ATPase component	94	75	99
MCB1EB_2252	OR16_03807	Spermidine/putrescine-binding periplasmic protein	98	-	-
MCB1EB_2253	OR16_03807	Spermidine/putrescine-binding periplasmic protein	88	-	-
MCB1EB_2254	OR16_12515	Spermidine/putrescine-binding periplasmic protein	92-99	-	-

MCB1EB_2255	Bphy_5667	Transcriptional regulator	100	84	91
MCB1EB_2256	-	Na+/citrate symporter	-	-	-
MCB1EB_2257	-	Beta-lactamase class D	-	-	-
MCB1EB_2258	-	Predicted PP-loop superfamily ATPase	100	74	98
MCB1EB_2259	-	Organic radical activating enzymes	100	73	99
MCB1EB_2260	mdrB	6-pyruvoyl-tetrahydropterin synthase	100	72	98
MCB1EB_2261	mrdA	Bacterial cell division membrane protein	100	73	98
MCB1EB_2262	mreD	Cell division protein FtsI/penicillin-binding protein 2	100	74	97
MCB1EB_2263	mreC	Cell shape-determining protein	100	75	100
MCB1EB_2264		Cell shape-determining protein	100	66	80
MCB1EB_2265	gatC	Actin-like ATPase involved in cell morphogenesis	100	-	
MCB1EB_2266	gatA	Asp-tRNAAsn/Glu-tRNAGln amidotransferase C subunit	100	65	100
MCB1EB_2267	gatB	Asp-tRNAAsn/Glu-tRNAGln amidotransferase A subunit and related amidases	100	75	100
MCB1EB_2268	-	Asp-tRNAAsn/Glu-tRNAGln amidotransferase B subunit (PET112 homolog)	100	73	100
MCB1EB_2269	BCh11DRAFT_04361	Exonuclease III	100	76	100
MCB1EB_2270	-	Zn-dependent protease with chaperone function	100	38	93
MCB1EB_2271	metW	Permeases of the major facilitator superfamily	100	70	98
MCB1EB_2272	metX	SAM-dependent methyltransferases	100	74	96
MCB1EB_2273	argB	Homoserine acetyltransferase	100	-	-
MCB1EB_2274	hslU	Acetylglutamate kinase	100	74	100
MCB1EB_2275	clpQ	ATP-dependent protease HslVU (ClpYQ), ATPase subunit	99	77	100
MCB1EB_2276	dksA	ATP-dependent protease HslVU (ClpYQ), peptidase subunit	100	84	86
MCB1EB_2277	B1M_03060	DnaK suppressor protein	100	85	100
MCB1EB_2278	-	Cytochrome c551/c552	100	-	-
MCB1EB_2279	-	Predicted periplasmic or secreted lipoprotein	100	57	88
MCB1EB_2280	CAGGBEG34_180034	Phosphoheptose isomerase	100	52	100
MCB1EB_2281	-	Predicted endonuclease distantly related to archaeal Holliday junction resolvase	100	-	
MCB1EB_2282	LLDT2_10835	Predicted methyltransferases	100	65	100
MCB1EB_2283	CARN4_2025	Putative glycosyl/glycerophosphate transferases involved in teichoic acid biosynthesis TagF/TagB/EpsJ/RodC	93	-	-
MCB1EB_2284	-	Protein containing a metal-binding domain shared with formylmethanofuran dehydrogenase subunit E	36	-	-
MCB1EB_2285	-	Transposase and inactivated derivatives	100	-	95
MCB1EB_2286	B3C1_06283	Transposase and inactivated derivatives	100	82-84	100
MCB1EB_2287	Acife_0758	Phosphate uptake regulator	-	-	-
MCB1EB_2288	Acife_0757	Hypothetical protein	-	-	-
MCB1EB_2289	-	Hypothetical protein	-	-	-
MCB1EB_2290	lipA	18 : Unknown function	-	-	-
MCB1EB_2291	lipB	Lipoate synthase	100	86	-
MCB1EB_2292	-	Lipoate-protein ligase B	99	-	-
MCB1EB_2293	-	Uncharacterized conserved protein	100	54	100
MCB1EB_2294	-	Branched-chain amino acid aminotransferase/4-amino-4-deoxychorismate lyase	100	-	-
MCB1EB_2295	-	D-alanyl-D-alanine carboxypeptidase	100	68	92
MCB1EB_2296	-	Predicted hydrolase of the alpha/beta superfamily	100	70	100
MCB1EB_2297	-	Ferredoxin	-	-	-
MCB1EB_2298	-	Response regulator containing CheY-like receiver, AAA-type ATPase, and DNA-binding domains	-	69	-
MCB1EB_2299	-	Signal transduction histidine kinase involved in nitrogen fixation and metabolism regulation	-	71	-
MCB1EB_2300	-	Hypothetical protein	100	71	88
MCB1EB_2301	fmt	tRNA and rRNA cytosine-C5-methylases	100	59	100
MCB1EB_2302	htpX	Methionyl-tRNA formyltransferase	100	67	100
MCB1EB_2303	C266_09527	Zn-dependent protease with chaperone function	100	81	99
MCB1EB_2304	defl	4-amino-4-deoxy-L-arabinose transferase and related glycosyltransferases of PMT family	96	35	93
MCB1EB_2305	smf	N-formylmethionyl-tRNA deformylase	100	77	99
MCB1EB_2306	-	Predicted Rossmann fold nucleotide-binding protein involved in DNA uptake	100	-	-
MCB1EB_2307	topB	Thiol-disulfide isomerase and thioredoxins	100	59	96
MCB1EB_2308	-	Topoisomerase IA	100	82	100
MCB1EB_2309	-	Site-specific recombinases, DNA invertase Pin homologs	-	-	-
MCB1EB_2310	leu20	Uncharacterized conserved protein	-	-	-
MCB1EB_2311	A176_03014	Citrate lyase beta subunit	-	-	-
MCB1EB_2312	X636_23345	Citrate lyase beta subunit	-	-	-
MCB1EB_2313	CAGGBEG34_190029	Na+/citrate symporter	100	-	-
MCB1EB_2314	-	Predicted transcriptional regulators	-	-	-
MCB1EB_2315	dinJ	Predicted transcriptional regulators	100	-	-
MCB1EB_2316	-	DNA-damage-inducible protein J	-	-	-
MCB1EB_2317	-	Preprotein translocase subunit YidC	100	70	100

Characterized protein

Hypothetical protein

Transposase and inactivated derivatives

Supplemental Table S3. Relative CDSs in prophage of B1-EB^{T} from the bacteriophages infecting bacterial hosts.

Relative CDSs in prophage of B1-EB^{T}	Bacterial host in phylum	Host
	B1-EB^{T}	
PHAGE_Haemop_Aaphi23_NC_004827	Gammaproteobacteria	*Aggregatibacter actinomycetemcomitans*
PHAGE_Haemop_Aaphi23_NC_004827	Gammaproteobacteria	*Aggregatibacter actinomycetemcomitans*
PHAGE_Haemop_Aaphi23_NC_004827	Gammaproteobacteria	*Aggregatibacter actinomycetemcomitans*
PHAGE_Haemop_Aaphi23_NC_004827	Gammaproteobacteria	*Aggregatibacter actinomycetemcomitans*
PHAGE_Haemop_Aaphi23_NC_004827	Gammaproteobacteria	*Aggregatibacter actinomycetemcomitans*
PHAGE_Haemop_Aaphi23_NC_004827	Gammaproteobacteria	*Aggregatibacter actinomycetemcomitans*
PHAGE_Haemop_Aaphi23_NC_004827	Gammaproteobacteria	*Aggregatibacter actinomycetemcomitans*
PHAGE_Haemop_Aaphi23_NC_004827	Gammaproteobacteria	*Aggregatibacter actinomycetemcomitans*
PHAGE_Aggreg_S1249_NC_013597	Gammaproteobacteria	*Aggregatibacter actinomycetemcomitans* D11S-1
PHAGE_Aggreg_S1249_NC_013597	Gammaproteobacteria	*Aggregatibacter actinomycetemcomitans* D11S-1
PHAGE_Aggreg_S1249_NC_013597	Gammaproteobacteria	*Aggregatibacter actinomycetemcomitans* D11S-1
PHAGE_Aggreg_S1249_NC_013597	Gammaproteobacteria	*Aggregatibacter actinomycetemcomitans* D11S-1
PHAGE_Aggreg_S1249_NC_013597	Gammaproteobacteria	*Aggregatibacter actinomycetemcomitans* D11S-1
PHAGE_Aggreg_S1249_NC_013597	Gammaproteobacteria	*Aggregatibacter actinomycetemcomitans* D11S-1
PHAGE_Aggreg_S1249_NC_013597	Gammaproteobacteria	*Aggregatibacter actinomycetemcomitans* D11S-1
PHAGE_Aggreg_S1249_NC_013597	Gammaproteobacteria	*Aggregatibacter actinomycetemcomitans* D11S-1
PHAGE_Aggreg_S1249_NC_013597	Gammaproteobacteria	*Aggregatibacter actinomycetemcomitans* D11S-1
PHAGE_Aggreg_S1249_NC_013597	Gammaproteobacteria	*Aggregatibacter actinomycetemcomitans* D11S-1
PHAGE_Aggreg_S1249_NC_013597	Gammaproteobacteria	*Aggregatibacter actinomycetemcomitans* D11S-1
PHAGE_Aggreg_S1249_NC_013597	Gammaproteobacteria	*Aggregatibacter actinomycetemcomitans* D11S-1
PHAGE_Aggreg_S1249_NC_013597	Gammaproteobacteria	*Aggregatibacter actinomycetemcomitans* D11S-1
PHAGE_Aggreg_S1249_NC_013597	Gammaproteobacteria	*Aggregatibacter actinomycetemcomitans* D11S-1
PHAGE_Aggreg_S1249_NC_013597	Gammaproteobacteria	*Aggregatibacter actinomycetemcomitans* D11S-1
PHAGE_Aggreg_S1249_NC_013597	Gammaproteobacteria	*Aggregatibacter actinomycetemcomitans* D11S-1
PHAGE_Aggreg_S1249_NC_013597	Gammaproteobacteria	*Aggregatibacter actinomycetemcomitans* D11S-1
PHAGE_Aggreg_S1249_NC_013597	Gammaproteobacteria	*Aggregatibacter actinomycetemcomitans* D11S-1
PHAGE_Aggreg_S1249_NC_013597	Gammaproteobacteria	*Aggregatibacter actinomycetemcomitans* D11S-1
PHAGE_Aggreg_S1249_NC_013597	Gammaproteobacteria	*Aggregatibacter actinomycetemcomitans* D11S-1
PHAGE_Aggreg_S1249_NC_013597	Gammaproteobacteria	*Aggregatibacter actinomycetemcomitans* D11S-1
PHAGE_Aggreg_S1249_NC_013597	Gammaproteobacteria	*Aggregatibacter actinomycetemcomitans* D11S-1
PHAGE_Aggreg_S1249_NC_013597	Gammaproteobacteria	*Aggregatibacter actinomycetemcomitans* D11S-1
PHAGE_Aggreg_S1249_NC_013597	Gammaproteobacteria	*Aggregatibacter actinomycetemcomitans* D11S-1
PHAGE_Aggreg_S1249_NC_013597	Gammaproteobacteria	*Aggregatibacter actinomycetemcomitans* D11S-1
PHAGE_Aggreg_S1249_NC_013597	Gammaproteobacteria	*Aggregatibacter actinomycetemcomitans* D11S-1
PHAGE_Aggreg_S1249_NC_013597	Gammaproteobacteria	*Aggregatibacter actinomycetemcomitans* D11S-1
PHAGE_Aggreg_S1249_NC_013597	Gammaproteobacteria	*Aggregatibacter actinomycetemcomitans* D11S-1
PHAGE_Aggreg_S1249_NC_013597	Gammaproteobacteria	*Aggregatibacter actinomycetemcomitans* D11S-1
PHAGE_Aggreg_S1249_NC_013597	Gammaproteobacteria	*Aggregatibacter actinomycetemcomitans* D11S-1
PHAGE_Aggreg_S1249_NC_013597	Gammaproteobacteria	*Aggregatibacter actinomycetemcomitans* D11S-1
PHAGE_Aggreg_S1249_NC_013597	Gammaproteobacteria	*Aggregatibacter actinomycetemcomitans* D11S-1
PHAGE_Aggreg_S1249_NC_013597	Gammaproteobacteria	*Aggregatibacter actinomycetemcomitans* D11S-1
PHAGE_Aggreg_S1249_NC_013597	Gammaproteobacteria	*Aggregatibacter actinomycetemcomitans* D11S-1
PHAGE_Aggreg_S1249_NC_013597	Gammaproteobacteria	*Aggregatibacter actinomycetemcomitans* D11S-1
PHAGE_Aggreg_S1249_NC_013597	Gammaproteobacteria	*Aggregatibacter actinomycetemcomitans* D11S-1
PHAGE_Aggreg_S1249_NC_013597	Gammaproteobacteria	*Aggregatibacter actinomycetemcomitans* D11S-1
PHAGE_Aggreg_S1249_NC_013597	Gammaproteobacteria	*Aggregatibacter actinomycetemcomitans* D11S-1
PHAGE_Aggreg_S1249_NC_013597	Gammaproteobacteria	*Aggregatibacter actinomycetemcomitans* D11S-1
PHAGE_Aggreg_S1249_NC_013597	Gammaproteobacteria	*Aggregatibacter actinomycetemcomitans* D11S-1
PHAGE_Aggreg_S1249_NC_013597	Gammaproteobacteria	*Aggregatibacter actinomycetemcomitans* D11S-1
PHAGE_Aggreg_S1249_NC_013597	Gammaproteobacteria	*Aggregatibacter actinomycetemcomitans* D11S-1
PHAGE_Aggreg_S1249_NC_013597	Gammaproteobacteria	*Aggregatibacter actinomycetemcomitans* D11S-1
PHAGE_Aggreg_S1249_NC_013597	Gammaproteobacteria	*Aggregatibacter actinomycetemcomitans* D11S-1
PHAGE_Aggreg_S1249_NC_013597	Gammaproteobacteria	*Aggregatibacter actinomycetemcomitans* D11S-1
PHAGE_Aggreg_S1249_NC_013597	Gammaproteobacteria	*Aggregatibacter actinomycetemcomitans* D11S-1
PHAGE_Aggreg_S1249_NC_013597	Gammaproteobacteria	*Aggregatibacter actinomycetemcomitans* D11S-1
PHAGE_Aggreg_S1249_NC_013597	Gammaproteobacteria	*Aggregatibacter actinomycetemcomitans* D11S-1
PHAGE_Aggreg_S1249_NC_013597	Gammaproteobacteria	*Aggregatibacter actinomycetemcomitans* D11S-1
PHAGE_Aggreg_S1249_NC_013597	Gammaproteobacteria	*Aggregatibacter actinomycetemcomitans* D11S-1
PHAGE_Aggreg_S1249_NC_013597	Gammaproteobacteria	*Aggregatibacter actinomycetemcomitans* D11S-1
PHAGE_Aggreg_S1249_NC_013597	Gammaproteobacteria	*Aggregatibacter actinomycetemcomitans* D11S-1
PHAGE_Aggreg_S1249_NC_013597	Gammaproteobacteria	*Aggregatibacter actinomycetemcomitans* D11S-1
PHAGE_Aggreg_S1249_NC_013597	Gammaproteobacteria	*Aggregatibacter actinomycetemcomitans* D11S-1
PHAGE_Aggreg_S1249_NC_013597	Gammaproteobacteria	*Aggregatibacter actinomycetemcomitans* D11S-1
PHAGE_Aggreg_S1249_NC_013597	Gammaproteobacteria	*Aggregatibacter actinomycetemcomitans* D11S-1
PHAGE_Aggreg_S1249_NC_013597	Gammaproteobacteria	*Aggregatibacter actinomycetemcomitans* D11S-1
PHAGE_Aggreg_S1249_NC_013597	Gammaproteobacteria	*Aggregatibacter actinomycetemcomitans* D11S-1
PHAGE_Aggreg_S1249_NC_013597	Gammaproteobacteria	*Aggregatibacter actinomycetemcomitans* D11S-1
PHAGE_Aggreg_S1249_NC_013597	Gammaproteobacteria	*Aggregatibacter actinomycetemcomitans* D11S-1
PHAGE_Aggreg_S1249_NC_013597	Gammaproteobacteria	*Aggregatibacter actinomycetemcomitans* D11S-1
PHAGE_Aggreg_S1249_NC_013597	Gammaproteobacteria	*Aggregatibacter actinomycetemcomitans* D11S-1
PHAGE_Aggreg_S1249_NC_013597	Gammaproteobacteria	*Aggregatibacter actinomycetemcomitans* D11S-1
PHAGE_Aggreg_S1249_NC_013597	Gammaproteobacteria	*Aggregatibacter actinomycetemcomitans* D11S-1
PHAGE_Aggreg_S1249_NC_013597	Gammaproteobacteria	*Aggregatibacter actinomycetemcomitans* D11S-1
PHAGE_Aggreg_S1249_NC_013597	Gammaproteobacteria	*Aggregatibacter actinomycetemcomitans* D11S-1
PHAGE_Aggreg_S1249_NC_013597	Gammaproteobacteria	*Aggregatibacter actinomycetemcomitans* D11S-1
PHAGE_Aggreg_S1249_NC_013597	Gammaproteobacteria	*Aggregatibacter actinomycetemcomitans* D11S-1
PHAGE_Aggreg_S1249_NC_013597	Gammaproteobacteria	*Aggregatibacter actinomycetemcomitans* D11S-1
PHAGE_Aggreg_S1249_NC_013597	Gammaproteobacteria	*Aggregatibacter actinomycetemcomitans* D11S-1
PHAGE_Aggreg_S1249_NC_013597	Gammaproteobacteria	*Aggregatibacter actinomycetemcomitans* D11S-1
PHAGE_Aggreg_S1249_NC_013597	Gammaproteobacteria	*Aggregatibacter actinomycetemcomitans* D11S-1
PHAGE_Aggreg_S1249_NC_013597	Gammaproteobacteria	*Aggregatibacter actinomycetemcomitans* D11S-1
PHAGE_Aggreg_S1249_NC_013597	Gammaproteobacteria	*Aggregatibacter actinomycetemcomitans* D11S-1
PHAGE_Aggreg_S1249_NC_013597	Gammaproteobacteria	*Aggregatibacter actinomycetemcomitans* D11S-1
PHAGE_Aggreg_S1249_NC_013597	Gammaproteobacteria	*Aggregatibacter actinomycetemcomitans* D11S-1
PHAGE_Aggreg_S1249_NC_013597	Gammaproteobacteria	*Aggregatibacter actinomycetemcomitans* D11S-1
PHAGE_Aggreg_S1249_NC_013597	Gammaproteobacteria	*Aggregatibacter actinomycetemcomitans* D11S-1
PHAGE_Aggreg_S1249_NC_013597	Gammaproteobacteria	*Aggregatibacter actinomycetemcomitans* D11S-1
PHAGE_Aggreg_S1249_NC_013597	Gammaproteobacteria	*Aggregatibacter actinomycetemcomitans* D11S-1
PHAGE_Azospi_Cd_NC_010355	Alphaproteobacteria	*Azospirillum brasilense* Cd

PHAGE_Azospi_Cd_NC_010355	Alphaproteobacteria	*Azospirillum brasilense* Cd
PHAGE_Bordet_BPP_1_NC_005357	Betaproteobacteria	*Bordetella bronchiseptica*
PHAGE_Bordet_BPP_1_NC_005357	Betaproteobacteria	*Bordetella bronchiseptica*
PHAGE_Bordet_BPP_1_NC_005357	Betaproteobacteria	*Bordetella bronchiseptica*
PHAGE_Bordet_BPP_1_NC_005357	Betaproteobacteria	*Bordetella bronchiseptica*
PHAGE_Bordet_BPP_1_NC_005357	Betaproteobacteria	*Bordetella bronchiseptica*
PHAGE_Bordet_BPP_1_NC_005357	Betaproteobacteria	*Bordetella bronchiseptica*
PHAGE_Bordet_BPP_1_NC_005357	Betaproteobacteria	*Bordetella bronchiseptica*
PHAGE_Brevib_Jimmer1_NC_029104	Firmicutes	*Brevibacillus laterosporus* (lab_host)
PHAGE_Brucel_BiPBO1_NC_031264	Alphaproteobacteria	*Brucella inopinata strain* BO1
PHAGE_Brucel_BiPBO1_NC_031264	Alphaproteobacteria	*Brucella inopinata strain* BO1
PHAGE_Brucel_BiPBO1_NC_031264	Alphaproteobacteria	*Brucella inopinata strain* BO1
PHAGE_Brucel_BiPBO1_NC_031264	Alphaproteobacteria	*Brucella inopinata strain* BO1
PHAGE_Burkho_KL3_NC_015266	Betaproteobacteria	*Burkholderia ambifaria* strain LMG 17828
PHAGE_Burkho_KS9_NC_013055	Betaproteobacteria	*Burkholderia ambifaria* strain LMG 17828
PHAGE_Burkho_KS9_NC_013055	Betaproteobacteria	*Burkholderia ambifaria* strain LMG 17828
PHAGE_Burkho_KS9_NC_013055	Betaproteobacteria	*Burkholderia ambifaria* strain LMG 17828
PHAGE_Burkho_KS5_NC_015265	Betaproteobacteria	*Burkholderia cenocepacia* strain C6433
PHAGE_Burkho_KS5_NC_015265	Betaproteobacteria	*Burkholderia cenocepacia* strain C6433
PHAGE_Burkho_KS5_NC_015265	Betaproteobacteria	*Burkholderia cenocepacia* strain C6433
PHAGE_Burkho_KS5_NC_015265	Betaproteobacteria	*Burkholderia cenocepacia* strain C6433
PHAGE_Burkho_KS5_NC_015265	Betaproteobacteria	*Burkholderia cenocepacia* strain C6433
PHAGE_Burkho_KS5_NC_015265	Betaproteobacteria	*Burkholderia cenocepacia* strain C6433
PHAGE_Burkho_KS5_NC_015265	Betaproteobacteria	*Burkholderia cenocepacia* strain C6433
PHAGE_Burkho_KS5_NC_015265	Betaproteobacteria	*Burkholderia cenocepacia* strain C6433
PHAGE_Burkho_Bcep176_NC_007497	Betaproteobacteria	*Burkholderia cepacia*
PHAGE_Burkho_Bcep176_NC_007497	Betaproteobacteria	*Burkholderia cepacia*
PHAGE_Burkho_Bcep176_NC_007497	Betaproteobacteria	*Burkholderia cepacia*
PHAGE_Burkho_Bcep176_NC_007497	Betaproteobacteria	*Burkholderia cepacia*
PHAGE_Burkho_Bcep176_NC_007497	Betaproteobacteria	*Burkholderia cepacia*
PHAGE_Burkho_phiE202_NC_009234	Betaproteobacteria	*Burkholderia thailandensis*
PHAGE_Burkho_phiE202_NC_009234	Betaproteobacteria	*Burkholderia thailandensis*
PHAGE_Burkho_phiE255_NC_009237	Betaproteobacteria	*Burkholderia* virus phiE255
PHAGE_Bacter_APSE_2_NC_011551	Gammaproteobacteria	*Candidatus Hamiltonella defensa*
PHAGE_Pseudo_YMC11/07/P54_PAE_BP_NC_030909	Gammaproteobacteria	Carbapenem-resistant *Pseudomonas aeruginosa* YMC11/07/P54
PHAGE_Pseudo_YMC11/07/P54_PAE_BP_NC_030909	Gammaproteobacteria	Carbapenem-resistant *Pseudomonas aeruginosa* YMC11/07/P54
PHAGE_Pseudo_YMC11/07/P54_PAE_BP_NC_030909	Gammaproteobacteria	Carbapenem-resistant *Pseudomonas aeruginosa* YMC11/07/P54
PHAGE_Pseudo_YMC11/07/P54_PAE_BP_NC_030909	Gammaproteobacteria	Carbapenem-resistant *Pseudomonas aeruginosa* YMC11/07/P54
PHAGE_Pseudo_YMC11/07/P54_PAE_BP_NC_030909	Gammaproteobacteria	Carbapenem-resistant *Pseudomonas aeruginosa* YMC11/07/P54
PHAGE_Pseudo_YMC11/07/P54_PAE_BP_NC_030909	Gammaproteobacteria	Carbapenem-resistant *Pseudomonas aeruginosa* YMC11/07/P54
PHAGE_Cellul_phi19:1_NC_021799	Bacteroidetes	*Cellulophaga baltica* #19
PHAGE_Cellul_phi19:1_NC_021799	Bacteroidetes	*Cellulophaga baltica* #19
PHAGE_Cellul_phi19:1_NC_021799	Bacteroidetes	*Cellulophaga baltica* #19
PHAGE_Cellul_phi19:1_NC_021799	Bacteroidetes	*Cellulophaga baltica* #19
PHAGE_Clostr_CDMH1_NC_024144	Firmicutes	*Clostridium*
PHAGE_Clostr_CDMH1_NC_024144	Firmicutes	*Clostridium*
PHAGE_Clostr_CDMH1_NC_024144	Firmicutes	*Clostridium*
PHAGE_Clostr_CDMH1_NC_024144	Firmicutes	*Clostridium*
PHAGE_Clostr_CDMH1_NC_024144	Firmicutes	*Clostridium*
PHAGE_Clostr_CDMH1_NC_024144	Firmicutes	*Clostridium*
PHAGE_Edward_PEi21_NC_021342	Gammaproteobacteria	*Edwardsiella ictaluri*
PHAGE_Edward_MSW_3_NC_020082	Gammaproteobacteria	*Edwardsiella tarda*
PHAGE_Edward_MSW_3_NC_020082	Gammaproteobacteria	*Edwardsiella tarda*
PHAGE_Edward_MSW_3_NC_020082	Gammaproteobacteria	*Edwardsiella tarda*
PHAGE_Edward_MSW_3_NC_020082	Gammaproteobacteria	*Edwardsiella tarda*
PHAGE_Edward_MSW_3_NC_020082	Gammaproteobacteria	*Edwardsiella tarda*
PHAGE_Edward_MSW_3_NC_020082	Gammaproteobacteria	*Edwardsiella tarda*
PHAGE_Edward_MSW_3_NC_020082	Gammaproteobacteria	*Edwardsiella tarda*
PHAGE_Edward_MSW_3_NC_020082	Gammaproteobacteria	*Edwardsiella tarda*
PHAGE_Edward_MSW_3_NC_020082	Gammaproteobacteria	*Edwardsiella tarda*
PHAGE_Edward_MSW_3_NC_020082	Gammaproteobacteria	*Edwardsiella tarda*
PHAGE_Entero_c_1_NC_019706	Gammaproteobacteria	*Escherichia coli* (lab_host)
PHAGE_Entero_c_1_NC_019706	Gammaproteobacteria	*Escherichia coli* (lab_host)
PHAGE_Entero_mEp390_NC_019721	Gammaproteobacteria	*Escherichia coli* (lab_host)
PROPHAGE_Escher_CFT073	Gammaproteobacteria	*Escherichia coli* CFT073
PROPHAGE_Escher_CFT073	Gammaproteobacteria	*Escherichia coli* CFT073
PROPHAGE_Escher_CFT073	Gammaproteobacteria	*Escherichia coli* CFT073
PROPHAGE_Escher_CFT073	Gammaproteobacteria	*Escherichia coli* CFT073
PROPHAGE_Escher_CFT073	Gammaproteobacteria	*Escherichia coli* CFT073
PROPHAGE_Escher_CFT073	Gammaproteobacteria	*Escherichia coli* CFT073
PROPHAGE_Escher_CFT073	Gammaproteobacteria	*Escherichia coli* CFT073
PROPHAGE_Escher_CFT073	Gammaproteobacteria	*Escherichia coli* CFT073
PROPHAGE_Escher_CFT073	Gammaproteobacteria	*Escherichia coli* CFT073
PROPHAGE_Escher_CFT073	Gammaproteobacteria	*Escherichia coli* CFT073
PROPHAGE_Escher_CFT073	Gammaproteobacteria	*Escherichia coli* CFT073
PHAGE_Escher_pro483_NC_028943	Gammaproteobacteria	*Escherichia coli* DE048
PROPHAGE_Escher_EDL933	Gammaproteobacteria	*Escherichia coli* EDL933
PROPHAGE_Escher_EDL933	Gammaproteobacteria	*Escherichia coli* EDL933
PROPHAGE_Escher_EDL933	Gammaproteobacteria	*Escherichia coli* EDL933
PROPHAGE_Escher_EDL933	Gammaproteobacteria	*Escherichia coli* EDL933
PHAGE_Escher_UFV_AREG1_NC_031030	Gammaproteobacteria	*Escherichia coli* O157 (lab_host)
PHAGE_Escher_UFV_AREG1_NC_031030	Gammaproteobacteria	*Escherichia coli* O157 (lab_host)
PHAGE_Escher_UFV_AREG1_NC_031030	Gammaproteobacteria	*Escherichia coli* O157 (lab_host)
PHAGE_Escher_UFV_AREG1_NC_031030	Gammaproteobacteria	*Escherichia coli* O157 (lab_host)
PHAGE_Escher_UFV_AREG1_NC_031030	Gammaproteobacteria	*Escherichia coli* O157 (lab_host)
PHAGE_Stx2_II_NC_004914	Gammaproteobacteria	*Escherichia coli* O157:H7 Morioka V526
PHAGE_Entero_fiAA91_ss_NC_022750	Gammaproteobacteria	*Escherichia coli* O157:H7; Shigella sonnei 866 (lab_host)
PHAGE_Entero_fiAA91_ss_NC_022750	Gammaproteobacteria	*Escherichia coli* O157:H7; Shigella sonnei 866 (lab_host)
PHAGE_Entero_fiAA91_ss_NC_022750	Gammaproteobacteria	*Escherichia coli* O157:H7; Shigella sonnei 866 (lab_host)
PHAGE_Entero_fiAA91_ss_NC_022750	Gammaproteobacteria	*Escherichia coli* O157:H7; Shigella sonnei 866 (lab_host)
PHAGE_Entero_fiAA91_ss_NC_022750	Gammaproteobacteria	*Escherichia coli* O157:H7; Shigella sonnei 866 (lab_host)
PHAGE_Entero_fiAA91_ss_NC_022750	Gammaproteobacteria	*Escherichia coli* O157:H7; Shigella sonnei 866 (lab_host)
PHAGE_Entero_fiAA91_ss_NC_022750	Gammaproteobacteria	*Escherichia coli* O157:H7; Shigella sonnei 866 (lab_host)
PHAGE_Entero_fiAA91_ss_NC_022750	Gammaproteobacteria	*Escherichia coli* O157:H7; Shigella sonnei 866 (lab_host)
PHAGE_Entero_fiAA91_ss_NC_022750	Gammaproteobacteria	*Escherichia coli* O157:H7; Shigella sonnei 866 (lab_host)
PHAGE_Gordon_GMA1_NC_030902	Actinobacteria	*Gordonia malaquae* strain BEN700
PHAGE_Iodoba_phiPLPE_NC_011142	Betaproteobacteria	*Iodobacter sp.* CdM7
PHAGE_Iodoba_phiPLPE_NC_011142	Betaproteobacteria	*Iodobacter sp.* CdM7
PHAGE_Iodoba_phiPLPE_NC_011142	Betaproteobacteria	*Iodobacter sp.* CdM7
PHAGE_Iodoba_phiPLPE_NC_011142	Betaproteobacteria	*Iodobacter sp.* CdM7

PHAGE_Iodoba_phiPLPE_NC_011142	Betaproteobacteria	*Iodobacter sp.* CdM7
PHAGE_Iodoba_phiPLPE_NC_011142	Betaproteobacteria	*Iodobacter sp.* CdM7
PHAGE_Lactob_LfeInf_NC_029058	Firmicutes	*Lactobacillus fermentum*
PHAGE_Mannhe_vB_MhS_1152AP2_NC_028956	Gammaproteobacteria	*Mannheimia haemolytica* 1152A serotype A6
PHAGE_Microc_MaMV_DC_NC_029002	Cyanobacteria	*Microcystis aeruginosa*
PHAGE_Microc_MaMV_DC_NC_029002	Cyanobacteria	*Microcystis aeruginosa*
PHAGE_Pseudo_Pq0_NC_029100	Gammaproteobacteria	*Pseudoalteromonas sp.* Bq0
PHAGE_Pseudo_PaMx11_NC_028770	Gammaproteobacteria	*Pseudomonas aeruginosa*
PHAGE_Pseudo_phi297_NC_016762	Gammaproteobacteria	*Pseudomonas aeruginosa*
PHAGE_Pseudo_JBD44_NC_030929	Gammaproteobacteria	*Pseudomonas aeruginosa* PA14
PHAGE_Pseudo_JBD44_NC_030929	Gammaproteobacteria	*Pseudomonas aeruginosa* PA14
PHAGE_Pseudo_phiPSA1_NC_024365	Gammaproteobacteria	*Pseudomonas syringae*
PHAGE_Pseudo_phiPSA1_NC_024365	Gammaproteobacteria	*Pseudomonas syringae*
PHAGE_Pseudo_phiPSA1_NC_024365	Gammaproteobacteria	*Pseudomonas syringae*
PHAGE_Pseudo_phiPSA1_NC_024365	Gammaproteobacteria	*Pseudomonas syringae*
PHAGE_Pseudo_phiPSA1_NC_024365	Gammaproteobacteria	*Pseudomonas syringae*
PHAGE_Ralsto_RSK1_NC_022915	Betaproteobacteria	*Ralstonia solanacearum*
PHAGE_Ralsto_RSK1_NC_022915	Betaproteobacteria	*Ralstonia solanacearum*
PHAGE_Ralsto_RSK1_NC_022915	Betaproteobacteria	*Ralstonia solanacearum*
PHAGE_Ralsto_RSK1_NC_022915	Betaproteobacteria	*Ralstonia solanacearum*
PROPHAGE_Ralsto_GMI1000	Betaproteobacteria	*Ralstonia solanacearum* GMI1000
PROPHAGE_Ralsto_GMI1000	Betaproteobacteria	*Ralstonia solanacearum* GMI1000
PHAGE_Rhizob_RHEph06_NC_027296	Alphaproteobacteria	*Rhizobium etli* GR56
PHAGE_Rhizob_RHEph06_NC_027296	Alphaproteobacteria	*Rhizobium etli* GR56
PHAGE_Rhizob_RHEph06_NC_027296	Alphaproteobacteria	*Rhizobium etli* GR56
PHAGE_Rhizob_RHEph06_NC_027296	Alphaproteobacteria	*Rhizobium etli* GR56
PHAGE_Rhizob_RHEph06_NC_027296	Alphaproteobacteria	*Rhizobium etli* GR56
PHAGE_Rhizob_vB_RleM_PPF1_NC_025427	Alphaproteobacteria	*Rhizobium leguminosarum* strain F1
PHAGE_Rhodoc_REQ3_NC_016654	Actinobacteria	*Rhodococcus equi*
PHAGE_Salmon_epsilon15_NC_004775	Gammaproteobacteria	*Salmonella enterica*
PHAGE_Salmon_BP63_NC_031250	Gammaproteobacteria	*Salmonella enterica Infantis*
PHAGE_Salmon_BP63_NC_031250	Gammaproteobacteria	*Salmonella enterica Infantis*
PHAGE_Salmon_9NA_NC_025443	Gammaproteobacteria	*Salmonella enterica* serovar *Typhiurium* strain LT2
PHAGE_Salmon_ST64T_NC_004348	Gammaproteobacteria	*Salmonella enterica* subsp. *enterica* serovar *Typhimurium*
PHAGE_Salmon_ST64T_NC_004348	Gammaproteobacteria	*Salmonella enterica* subsp. *enterica* serovar *Typhimurium*
PHAGE_Salmon_SEN1_NC_029003	Gammaproteobacteria	*Salmonella enterica* subsp. salamae 10/99 II
PROPHAGE_Shewan_MR-1	Gammaproteobacteria	*Shewanella oneidensis* MR-1
PROPHAGE_Shewan_MR-1	Gammaproteobacteria	*Shewanella oneidensis* MR-1
PROPHAGE_Shewan_MR-1	Gammaproteobacteria	*Shewanella oneidensis* MR-1
PROPHAGE_Shewan_MR-1	Gammaproteobacteria	*Shewanella oneidensis* MR-1
PROPHAGE_Shewan_MR-1	Gammaproteobacteria	*Shewanella oneidensis* MR-1
PROPHAGE_Shewan_MR-1	Gammaproteobacteria	*Shewanella oneidensis* MR-1
PROPHAGE_Shewan_MR-1	Gammaproteobacteria	*Shewanella oneidensis* MR-1
PHAGE_Entero_SfI_NC_027339	Gammaproteobacteria	*Shigella flexneri* serotype 1a strain 019
PHAGE_Entero_SfI_NC_027339	Gammaproteobacteria	*Shigella flexneri* serotype 1a strain 019
PHAGE_Entero_SfI_NC_027339	Gammaproteobacteria	*Shigella flexneri* serotype 1a strain 019
PHAGE_Shigel_SfIV_NC_022749	Gammaproteobacteria	*Shigella flexneri* serotype 4av
PHAGE_Shigel_SfIV_NC_022749	Gammaproteobacteria	*Shigella flexneri* serotype 4av
PHAGE_Shigel_SfIV_NC_022749	Gammaproteobacteria	*Shigella flexneri* serotype 4av
PHAGE_Entero_Sf101_NC_027398	Gammaproteobacteria	*Shigella flexneri* serotype 7a
PHAGE_Shigel_Stx_NC_029120	Gammaproteobacteria	*Shigella sonnei* 75/02
PHAGE_Sinorh_phiLM21_NC_029046	Alphaproteobacteria	*Sinorhizobium sp.* LM21
PHAGE_Sodali_phiSG1_NC_007902	Gammaproteobacteria	*Sodalis glossinidius* str. 'morsitans'
PHAGE_Sodali_phiSG1_NC_007902	Gammaproteobacteria	*Sodalis glossinidius* str. 'morsitans'
PHAGE_Spirop_1_C74_NC_003793	Tenericutes (Mollicutes)	*Spiroplasma citri*
PHAGE_Strept_phiARI0746_NC_031907	Firmicutes	*Streptococcus pneumoniae* 10B04751
PHAGE_Cronob_phiES15_NC_018454	Gammaproteobacteria	*UV-induced Cronobacter sakazakii* ES15
PHAGE_Cronob_phiES15_NC_018454	Gammaproteobacteria	*UV-induced Cronobacter sakazakii* ES15
PHAGE_Cronob_phiES15_NC_018454	Gammaproteobacteria	*UV-induced Cronobacter sakazakii* ES15
PHAGE_Vibrio_vB_VchM_138_NC_019518	Gammaproteobacteria	*Vibrio cholerae*
PHAGE_Vibrio_vB_VchM_138_NC_019518	Gammaproteobacteria	*Vibrio cholerae*
PHAGE_Vibrio_vB_VchM_138_NC_019518	Gammaproteobacteria	*Vibrio cholerae*
PHAGE_Vibrio_CP_T1_NC_019457	Gammaproteobacteria	*Vibrio cholerae* ElTor
PHAGE_Vibrio_CP_T1_NC_019457	Gammaproteobacteria	*Vibrio cholerae* ElTor
PROPHAGE_Xantho_306	Gammaproteobacteria	*Xanthomonas axonopodis* pv. citri str. 306
PROPHAGE_Xantho_306	Gammaproteobacteria	*Xanthomonas axonopodis* pv. citri str. 306
PROPHAGE_Xantho_306	Gammaproteobacteria	*Xanthomonas axonopodis* pv. citri str. 306
PHAGE_Xylell_Xfas53_NC_013599	Gammaproteobacteria	*Xylella fastidiosa*
PHAGE_Xylell_Xfas53_NC_013599	Gammaproteobacteria	*Xylella fastidiosa*
PHAGE_Xylell_Xfas53_NC_013599	Gammaproteobacteria	*Xylella fastidiosa*
PHAGE_Xylell_Xfas53_NC_013599	Gammaproteobacteria	*Xylella fastidiosa*
PHAGE_Xylell_Xfas53_NC_013599	Gammaproteobacteria	*Xylella fastidiosa*
PHAGE_Xylell_Xfas53_NC_013599	Gammaproteobacteria	*Xylella fastidiosa*
PHAGE_Xylell_Xfas53_NC_013599	Gammaproteobacteria	*Xylella fastidiosa*
PHAGE_Yersin_L_413C_NC_004745	Gammaproteobacteria	*Yersinia pestis*
**Mc-AG77**		
PHAGE_Haemop_Aaphi23_NC_004827	Gammaproteobacteria	*Aggregatibacter actinomycetemcomitans*
PHAGE_Haemop_Aaphi23_NC_004827	Gammaproteobacteria	*Aggregatibacter actinomycetemcomitans*
PHAGE_Aggreg_S1249_NC_013597	Gammaproteobacteria	*Aggregatibacter actinomycetemcomitans* D11S-1
PHAGE_Aggreg_S1249_NC_013597	Gammaproteobacteria	*Aggregatibacter actinomycetemcomitans* D11S-1
PHAGE_Aggreg_S1249_NC_013597	Gammaproteobacteria	*Aggregatibacter actinomycetemcomitans* D11S-1
PHAGE_Aggreg_S1249_NC_013597	Gammaproteobacteria	*Aggregatibacter actinomycetemcomitans* D11S-1
PHAGE_Aggreg_S1249_NC_013597	Gammaproteobacteria	*Aggregatibacter actinomycetemcomitans* D11S-1
PHAGE_Aggreg_S1249_NC_013597	Gammaproteobacteria	*Aggregatibacter actinomycetemcomitans* D11S-1
PHAGE_Aggreg_S1249_NC_013597	Gammaproteobacteria	*Aggregatibacter actinomycetemcomitans* D11S-1
PHAGE_Aggreg_S1249_NC_013597	Gammaproteobacteria	*Aggregatibacter actinomycetemcomitans* D11S-1
PHAGE_Aggreg_S1249_NC_013597	Gammaproteobacteria	*Aggregatibacter actinomycetemcomitans* D11S-1
PHAGE_Aggreg_S1249_NC_013597	Gammaproteobacteria	*Aggregatibacter actinomycetemcomitans* D11S-1
PHAGE_Aggreg_S1249_NC_013597	Gammaproteobacteria	*Aggregatibacter actinomycetemcomitans* D11S-1
PHAGE_Aggreg_S1249_NC_013597	Gammaproteobacteria	*Aggregatibacter actinomycetemcomitans* D11S-1
PHAGE_Aggreg_S1249_NC_013597	Gammaproteobacteria	*Aggregatibacter actinomycetemcomitans* D11S-1
PHAGE_Aggreg_S1249_NC_013597	Gammaproteobacteria	*Aggregatibacter actinomycetemcomitans* D11S-1
PHAGE_Aggreg_S1249_NC_013597	Gammaproteobacteria	*Aggregatibacter actinomycetemcomitans* D11S-1
PHAGE_Aggreg_S1249_NC_013597	Gammaproteobacteria	*Aggregatibacter actinomycetemcomitans* D11S-1
PHAGE_Aggreg_S1249_NC_013597	Gammaproteobacteria	*Aggregatibacter actinomycetemcomitans* D11S-1
PHAGE_Aggreg_S1249_NC_013597	Gammaproteobacteria	*Aggregatibacter actinomycetemcomitans* D11S-1
PHAGE_Aggreg_S1249_NC_013597	Gammaproteobacteria	*Aggregatibacter actinomycetemcomitans* D11S-1
PHAGE_Aggreg_S1249_NC_013597	Gammaproteobacteria	*Aggregatibacter actinomycetemcomitans* D11S-1
PHAGE_Aggreg_S1249_NC_013597	Gammaproteobacteria	*Aggregatibacter actinomycetemcomitans* D11S-1
PHAGE_Aggreg_S1249_NC_013597	Gammaproteobacteria	*Aggregatibacter actinomycetemcomitans* D11S-1

PHAGE_Aggreg_S1249_NC_013597	Gammaproteobacteria	*Aggregatibacter actinomycetemcomitans* D11S-1
PHAGE_Aggreg_S1249_NC_013597	Gammaproteobacteria	*Aggregatibacter actinomycetemcomitans* D11S-1
PHAGE_Aggreg_S1249_NC_013597	Gammaproteobacteria	*Aggregatibacter actinomycetemcomitans* D11S-1
PHAGE_Aggreg_S1249_NC_013597	Gammaproteobacteria	*Aggregatibacter actinomycetemcomitans* D11S-1
PHAGE_Aggreg_S1249_NC_013597	Gammaproteobacteria	*Aggregatibacter actinomycetemcomitans* D11S-1
PHAGE_Aggreg_S1249_NC_013597	Gammaproteobacteria	*Aggregatibacter actinomycetemcomitans* D11S-1
PHAGE_Aggreg_S1249_NC_013597	Gammaproteobacteria	*Aggregatibacter actinomycetemcomitans* D11S-1
PHAGE_Aggreg_S1249_NC_013597	Gammaproteobacteria	*Aggregatibacter actinomycetemcomitans* D11S-1
PHAGE_Aggreg_S1249_NC_013597	Gammaproteobacteria	*Aggregatibacter actinomycetemcomitans* D11S-1
PHAGE_Aggreg_S1249_NC_013597	Gammaproteobacteria	*Aggregatibacter actinomycetemcomitans* D11S-1
PHAGE_Aggreg_S1249_NC_013597	Gammaproteobacteria	*Aggregatibacter actinomycetemcomitans* D11S-1
PHAGE_Aggreg_S1249_NC_013597	Gammaproteobacteria	*Aggregatibacter actinomycetemcomitans* D11S-1
PHAGE_Azospi_Cd_NC_010355	Alphaproteobacteria	*Azospirillum brasilense* Cd
PHAGE_Brevib_Abouo_NC_029029	Firmicutes	*Brevibacillus laterosporus* (lab_host)
PHAGE_Burkho_KL3_NC_015266	Betaproteobacteria	*Burkholderia ambifaria* strain LMG 17828
PHAGE_Burkho_KS9_NC_013055	Betaproteobacteria	*Burkholderia ambifaria* strain LMG 17828
PHAGE_Burkho_KS9_NC_013055	Betaproteobacteria	*Burkholderia ambifaria* strain LMG 17828
PHAGE_Burkho_KS9_NC_013055	Betaproteobacteria	*Burkholderia ambifaria* strain LMG 17828
PHAGE_Burkho_KS9_NC_013055	Betaproteobacteria	*Burkholderia ambifaria* strain LMG 17828
PHAGE_Burkho_KS5_NC_015265	Betaproteobacteria	*Burkholderia cenocepacia* strain C6433
PHAGE_Burkho_KS5_NC_015265	Betaproteobacteria	*Burkholderia cenocepacia* strain C6433
PHAGE_Burkho_KS5_NC_015265	Betaproteobacteria	*Burkholderia cenocepacia* strain C6433
PHAGE_Burkho_KS5_NC_015265	Betaproteobacteria	*Burkholderia cenocepacia* strain C6433
PHAGE_Burkho_KS5_NC_015265	Betaproteobacteria	*Burkholderia cenocepacia* strain C6433
PHAGE_Burkho_KS5_NC_015265	Betaproteobacteria	*Burkholderia cenocepacia* strain C6433
PHAGE_Burkho_Bcep176_NC_007497	Betaproteobacteria	*Burkholderia cepacia*
PHAGE_Burkho_Bcep176_NC_007497	Betaproteobacteria	*Burkholderia cepacia*
PHAGE_Burkho_phiE255_NC_009237	Betaproteobacteria	*Burkholderia* virus phiE255
PHAGE_Pseudo_YMC11/07/P54_PAE_BP_NC_030909	Gammaproteobacteria	carbapenem-resistant *Pseudomonas aeruginosa* YMC11/07/P54
PHAGE_Pseudo_YMC11/07/P54_PAE_BP_NC_030909	Gammaproteobacteria	carbapenem-resistant *Pseudomonas aeruginosa* YMC11/07/P54
PHAGE_Cellul_phi19:1_NC_021799	Bacteriodetes	*Cellulophaga baltica* #19
PHAGE_Cellul_phi19:1_NC_021799	Bacteriodetes	*Cellulophaga baltica* #19
PHAGE_Clostr_CDMH1_NC_024144	Firmicutes	*Clostridium*
PHAGE_Clostr_CDMH1_NC_024144	Firmicutes	*Clostridium*
PHAGE_Clostr_CDMH1_NC_024144	Firmicutes	*Clostridium*
PHAGE_Clostr_CDMH1_NC_024144	Firmicutes	*Clostridium*
PHAGE_Edward_PEi21_NC_021342	Gammaproteobacteria	*Edwardsiella ictaluri*
PHAGE_Edward_MSW_3_NC_020082	Gammaproteobacteria	*Edwardsiella tarda*
PHAGE_Edward_MSW_3_NC_020082	Gammaproteobacteria	*Edwardsiella tarda*
PHAGE_Edward_MSW_3_NC_020082	Gammaproteobacteria	*Edwardsiella tarda*
PHAGE_Edward_MSW_3_NC_020082	Gammaproteobacteria	*Edwardsiella tarda*
PHAGE_Entero_BP_4795_NC_004813	Gammaproteobacteria	*Escherichia coli*
PHAGE_Entero_BP_4795_NC_004813	Gammaproteobacteria	*Escherichia coli*
PHAGE_Salmon_HK620_NC_002730	Gammaproteobacteria	*Escherichia coli*
PHAGE_Entero_mEp390_NC_019721	Gammaproteobacteria	*Escherichia coli* (lab_host)
PROPHAGE_Escher_CFT073	Gammaproteobacteria	*Escherichia coli* CFT073
PHAGE_Escher_pro483_NC_028943	Gammaproteobacteria	*Escherichia coli* DE048
PHAGE_Escher_pro483_NC_028943	Gammaproteobacteria	*Escherichia coli* DE048
PHAGE_Escher_UFV_AREG1_NC_031030	Gammaproteobacteria	*Escherichia coli O157* (lab_host)
PHAGE_Escher_UFV_AREG1_NC_031030	Gammaproteobacteria	*Escherichia coli O157* (lab_host)
PHAGE_Escher_UFV_AREG1_NC_031030	Gammaproteobacteria	*Escherichia coli O157* (lab_host)
PHAGE_Escher_UFV_AREG1_NC_031030	Gammaproteobacteria	*Escherichia coli O157* (lab_host)
PHAGE_Entero_fiAA91_ss_NC_022750	Gammaproteobacteria	*Escherichia coli* O157:H7
PHAGE_Entero_fiAA91_ss_NC_022750	Gammaproteobacteria	*Escherichia coli* O157:H7
PHAGE_Iodoba_phiPLPE_NC_011142	Betaproteobacteria	*Iodobacter sp.* CdM7
PHAGE_Iodoba_phiPLPE_NC_011142	Betaproteobacteria	*Iodobacter sp.* CdM7
PHAGE_Lactob_LfeInf_NC_029058	Firmicutes	*Lactobacillus fermentum*
PHAGE_Lister_LP_101_NC_024387	Firmicutes	*Listeria monocytogenes*
PHAGE_Mannhe_vB_MhS_1152AP2_NC_028956	Gammaproteobacteria	*Mannheimia haemolytica* 1152A serotype A6
PHAGE_Mannhe_vB_MhS_1152AP2_NC_028956	Gammaproteobacteria	*Mannheimia haemolytica* 1152A serotype A6
PHAGE_Mannhe_vB_MhS_1152AP2_NC_028956	Gammaproteobacteria	*Mannheimia haemolytica* 1152A serotype A6
PHAGE_Mannhe_vB_MhS_587AP2_NC_028743	Gammaproteobacteria	*Mannheimia haemolytica* 587A serotype A2
PHAGE_Microc_MaMV_DC_NC_029002	Cyanobacteria	*Microcystis aeruginosa*
PHAGE_Microc_MaMV_DC_NC_029002	Cyanobacteria	*Microcystis aeruginosa*
PHAGE_Microc_MaMV_DC_NC_029002	Cyanobacteria	*Microcystis aeruginosa*
PHAGE_Microc_MaMV_DC_NC_029002	Cyanobacteria	*Microcystis aeruginosa*
PHAGE_Mycoba_Enkosi_NC_028936	Actinobacteria	*Mycobacterium smegmatis* mc2 155 (lab_host)
PHAGE_Ralsto_RSA1_NC_009382	Betaproteobacteria	NC_023586
PHAGE_Pseudo_F116_NC_006552	Gammaproteobacteria	*Pseudomonas aeruginosa*
PHAGE_Pseudo_MD8_NC_031091	Gammaproteobacteria	*Pseudomonas aeruginosa*
PHAGE_Pseudo_MD8_NC_031091	Gammaproteobacteria	*Pseudomonas aeruginosa*
PHAGE_Pseudo_phi297_NC_016762	Gammaproteobacteria	*Pseudomonas aeruginosa*
PHAGE_Pseudo_PMG1_NC_016765	Gammaproteobacteria	*Pseudomonas aeruginosa*
PHAGE_Pseudo_vB_PaeS_PM105_NC_028667	Gammaproteobacteria	*Pseudomonas aeruginosa*
PHAGE_Pseudo_vB_PaeS_PM105_NC_028667	Gammaproteobacteria	*Pseudomonas aeruginosa*
PROPHAGE_Pseudo_KT2440	Gammaproteobacteria	*Pseudomonas putida* KT2440
PROPHAGE_Pseudo_KT2440	Gammaproteobacteria	*Pseudomonas putida* KT2440
PHAGE_Pseudo_phiPSA1_NC_024365	Gammaproteobacteria	*Pseudomonas syringae*
PHAGE_Ralsto_NP_2014_NC_023586	Betaproteobacteria	*Ralstonia*
PHAGE_Ralsto_RSK1_NC_022915	Betaproteobacteria	*Ralstonia solanacearum*
PROPHAGE_Ralsto_GMI1000	Betaproteobacteria	*Ralstonia solanacearum* GMI1000
PROPHAGE_Ralsto_GMI1000	Betaproteobacteria	*Ralstonia solanacearum* GMI1000
PHAGE_Rhizob_RHEph06_NC_027296	Alphaproteobacteria	*Rhizobium etli* GR56
PHAGE_Rhizob_RHEph06_NC_027296	Alphaproteobacteria	*Rhizobium etli* GR56
PHAGE_Salmon_SJ46_NC_031129	Gammaproteobacteria	*Salmonella enterica* subsp. *enterica* serovar IndianaJ46
PHAGE_Salmon_ST64T_NC_004348	Gammaproteobacteria	*Salmonella enterica* subsp. *enterica* serovar *Typhimurium*
PHAGE_Salmon_SEN1_NC_029003	Gammaproteobacteria	*Salmonella enterica* subsp. *salamae* 10/99 II
PROPHAGE_Shewan_MR-1	Gammaproteobacteria	*Shewanella oneidensis* MR-1
PROPHAGE_Shewan_MR-1	Gammaproteobacteria	*Shewanella oneidensis* MR-1
PROPHAGE_Shewan_MR-1	Gammaproteobacteria	*Shewanella oneidensis* MR-1
PHAGE_Entero_SfI_NC_027339	Gammaproteobacteria	*Shigella flexneri serotype* 1a strain 019
PHAGE_Shigel_SfIV_NC_022749	Gammaproteobacteria	*Shigella flexneri* serotype 4av
PHAGE_Shigel_SfIV_NC_022749	Gammaproteobacteria	*Shigella flexneri* serotype 4av
PHAGE_Shigel_SfIV_NC_022749	Gammaproteobacteria	*Shigella flexneri* serotype 4av
PHAGE_Shigel_SfIV_NC_022749	Gammaproteobacteria	*Shigella flexneri* serotype 4av
PHAGE_Shigel_SHBML_50_1_NC_031085	Gammaproteobacteria	*Shigella sonnei*
PHAGE_Cronob_phiES15_NC_018454	Gammaproteobacteria	*UV-induced Cronobacter sakazakii* ES15
PHAGE_Cronob_phiES15_NC_018454	Gammaproteobacteria	*UV-induced Cronobacter sakazakii* ES15
PHAGE_Vibrio_vB_VchM_138_NC_019518	Gammaproteobacteria	*Vibrio cholerae*
PHAGE_Vibrio_vB_VchM_138_NC_019518	Gammaproteobacteria	*Vibrio cholerae*

PROPHAGE_Xantho_33913	Gammaproteobacteria	*Xanthomonas campestris pv. campestris* ATCC 33913
PROPHAGE_Xantho_33913	Gammaproteobacteria	*Xanthomonas campestris pv. campestris* ATCC 33913
PHAGE_Xylell_Xfas53_NC_013599	Gammaproteobacteria	*Xylella fastidiosa*
PHAGE_Xylell_Xfas53_NC_013599	Gammaproteobacteria	*Xylella fastidiosa*
PHAGE_Xylell_Xfas53_NC_013599	Gammaproteobacteria	*Xylella fastidiosa*
PHAGE_Xylell_Xfas53_NC_013599	Gammaproteobacteria	*Xylella fastidiosa*
PHAGE_Xylell_Xfas53_NC_013599	Gammaproteobacteria	*Xylella fastidiosa*
PHAGE_Xylell_Xfas53_NC_013599	Gammaproteobacteria	*Xylella fastidiosa*
PHAGE_Xylell_Xfas53_NC_013599	Gammaproteobacteria	*Xylella fastidiosa*
	**HKI454^{T}**	
PHAGE_Acinet_vB_AbaS_TRS1_NC_031098	Gammaproteobacteria	*Acinetobacter baumannii* A118
PHAGE_Acinet_Bphi_B1251_NC_019541	Gammaproteobacteria	*Acinetobacter baumannii* YMC/09/02/B1251
PHAGE_Aeromo_phiAS5_NC_014636	Gammaproteobacteria	*Aeromonas salmonicida*
PHAGE_Aeromo_phiAS5_NC_014636	Gammaproteobacteria	*Aeromonas salmonicida*
PHAGE_Bacill_BCD7_NC_019515	Firmicutes	*Bacillus cereus*
PHAGE_Bacill_SP_15_NC_031245	Firmicutes	*Bacillus subtilis* W-23-S(r); *Bacillus licheniformis* ATCC 9945a (lab_host)
PHAGE_Bacill_BMBtpLA_NC_028748	Firmicutes	*Bacillus thuringiensis* serovar *tenebrionis* strain YBT-1765
PHAGE_Burkho_BcepF1_NC_009015	Betaproteobacteria	*Burkholderia ambifaria*
PHAGE_Burkho_KL3_NC_015266	Betaproteobacteria	*Burkholderia ambifaria* strain LMG 17828
PHAGE_Burkho_KL3_NC_015266	Betaproteobacteria	*Burkholderia ambifaria* strain LMG 17828
PHAGE_Burkho_Bcep22_NC_005262	Betaproteobacteria	*Burkholderia cenocepacia*
PHAGE_Burkho_KS5_NC_015265	Betaproteobacteria	*Burkholderia cenocepacia* strain C6433
PHAGE_Burkho_BcepB1A_NC_005886	Betaproteobacteria	*Burkholderia cepacia complex*
PHAGE_Burkho_BcepB1A_NC_005886	Betaproteobacteria	*Burkholderia cepacia complex*
PHAGE_Burkho_BcepB1A_NC_005886	Betaproteobacteria	*Burkholderia cepacia complex*
PHAGE_Burkho_BcepB1A_NC_005886	Betaproteobacteria	*Burkholderia cepacia complex*
PHAGE_Burkho_BcepB1A_NC_005886	Betaproteobacteria	*Burkholderia cepacia complex*
PHAGE_Burkho_BcepB1A_NC_005886	Betaproteobacteria	*Burkholderia cepacia complex*
PHAGE_Burkho_BcepB1A_NC_005886	Betaproteobacteria	*Burkholderia cepacia complex*
PHAGE_Burkho_BcepB1A_NC_005886	Betaproteobacteria	*Burkholderia cepacia complex*
PHAGE_Burkho_BcepB1A_NC_005886	Betaproteobacteria	*Burkholderia cepacia complex*
PHAGE_Burkho_BcepB1A_NC_005886	Betaproteobacteria	*Burkholderia cepacia complex*
PHAGE_Burkho_BcepB1A_NC_005886	Betaproteobacteria	*Burkholderia cepacia complex*
PHAGE_Burkho_BcepB1A_NC_005886	Betaproteobacteria	*Burkholderia cepacia complex*
PHAGE_Burkho_BcepB1A_NC_005886	Betaproteobacteria	*Burkholderia cepacia complex*
PHAGE_Burkho_BcepB1A_NC_005886	Betaproteobacteria	*Burkholderia cepacia complex*
PHAGE_Burkho_BcepB1A_NC_005886	Betaproteobacteria	*Burkholderia cepacia complex*
PHAGE_Burkho_BcepB1A_NC_005886	Betaproteobacteria	*Burkholderia cepacia complex*
PHAGE_Burkho_BcepB1A_NC_005886	Betaproteobacteria	*Burkholderia cepacia complex*
PHAGE_Burkho_ST79_NC_021343	Betaproteobacteria	*Burkholderia pseudomallei*
PHAGE_Burkho_KS9_NC_013055	Betaproteobacteria	*Burkholderia pyrrocinia* strain LMG 21824
PHAGE_Burkho_phiE125_NC_003309	Betaproteobacteria	*Burkholderia thailandensis*
PHAGE_Burkho_phiE202_NC_009234	Betaproteobacteria	*Burkholderia thailandensis*
PHAGE_Burkho_phiE202_NC_009234	Betaproteobacteria	*Burkholderia thailandensis*
PHAGE_Burkho_phiE202_NC_009234	Betaproteobacteria	*Burkholderia thailandensis*
PHAGE_Burkho_phiE202_NC_009234	Betaproteobacteria	*Burkholderia thailandensis*
PHAGE_Burkho_phiE202_NC_009234	Betaproteobacteria	*Burkholderia thailandensis*
PHAGE_Burkho_phiE202_NC_009234	Betaproteobacteria	*Burkholderia thailandensis*
PHAGE_Burkho_phiE202_NC_009234	Betaproteobacteria	*Burkholderia thailandensis*
PHAGE_Burkho_phiE202_NC_009234	Betaproteobacteria	*Burkholderia thailandensis*
PHAGE_Burkho_phiE202_NC_009234	Betaproteobacteria	*Burkholderia thailandensis*
PHAGE_Burkho_phiE202_NC_009234	Betaproteobacteria	*Burkholderia thailandensis*
PHAGE_Burkho_phiE202_NC_009234	Betaproteobacteria	*Burkholderia thailandensis*
PHAGE_Burkho_phiE202_NC_009234	Betaproteobacteria	*Burkholderia thailandensis*
PHAGE_Burkho_phiE202_NC_009234	Betaproteobacteria	*Burkholderia thailandensis*
PHAGE_Burkho_phiE202_NC_009234	Betaproteobacteria	*Burkholderia thailandensis*
PHAGE_Burkho_phiE202_NC_009234	Betaproteobacteria	*Burkholderia thailandensis*
PHAGE_Burkho_phiE202_NC_009234	Betaproteobacteria	*Burkholderia thailandensis*
PHAGE_Burkho_phiE202_NC_009234	Betaproteobacteria	*Burkholderia thailandensis*
PHAGE_Burkho_phiE202_NC_009234	Betaproteobacteria	*Burkholderia thailandensis*
PHAGE_Burkho_phiE202_NC_009234	Betaproteobacteria	*Burkholderia thailandensis*
PHAGE_Burkho_phiE202_NC_009234	Betaproteobacteria	*Burkholderia thailandensis*
PHAGE_Burkho_phiE202_NC_009234	Betaproteobacteria	*Burkholderia thailandensis*
PHAGE_Burkho_phiE202_NC_009234	Betaproteobacteria	*Burkholderia thailandensis*
PHAGE_Burkho_phiE202_NC_009234	Betaproteobacteria	*Burkholderia thailandensis*
PHAGE_Burkho_phiE202_NC_009234	Betaproteobacteria	*Burkholderia thailandensis*
PHAGE_Clostr_phiCT19406A_NC_030950	Firmicutes	*Clostridium tetani* ATCC 19406
PHAGE_Edward_MSW_3_NC_020082	Gammaproteobacteria	*Edwardsiella tarda*
PHAGE_Escher_172_1_NC_028903	Gammaproteobacteria	*Escherichia coli* 172
PHAGE_Escher_ECML_117_NC_025441	Gammaproteobacteria	*Escherichia coli* O157:H7
PHAGE_Pseudo_NH_4_NC_019451	Gammaproteobacteria	*Pseudomonas aeruginosa*
PHAGE_Pseudo_MP42_NC_018274	Gammaproteobacteria	*Pseudomonas aeruginosa* PA14
PHAGE_Pseudo_PaBG_NC_022096	Gammaproteobacteria	*Pseudomonas aeruginosa* PAO1
PROPHAGE_Pseudo_KT2440	Gammaproteobacteria	*Pseudomonas putida* KT2440
PHAGE_Ralsto_RSA1_NC_009382	Betaproteobacteria	*Ralstonia solanacearum*
PHAGE_Ralsto_RSJ2_NC_028988	Betaproteobacteria	*Ralstonia solanacearum*
PHAGE_Ralsto_RSJ2_NC_028988	Betaproteobacteria	*Ralstonia solanacearum*
PHAGE_Ralsto_RSS0_NC_019548	Betaproteobacteria	*Ralstonia solanacearum*
PROPHAGE_Ralsto_GMI1000	Betaproteobacteria	*Ralstonia solanacearum* GMI1000
PROPHAGE_Ralsto_GMI1000	Betaproteobacteria	*Ralstonia solanacearum* GMI1000
PROPHAGE_Ralsto_GMI1000	Betaproteobacteria	*Ralstonia solanacearum* GMI1000
PROPHAGE_Ralsto_GMI1000	Betaproteobacteria	*Ralstonia solanacearum* GMI1000
PHAGE_Salmon_SEN34_NC_028699	Gammaproteobacteria	*Salmonella enterica* subsp. *diarizonae* 519/03 IIIb
PHAGE_Salmon_SSU5_NC_018843	Gammaproteobacteria	*Salmonella enterica* subsp. *enterica* serovar *Typhimurium strain* LT2
PHAGE_Salmon_SEN1_NC_029003	Gammaproteobacteria	*Salmonella enterica* subsp. *salamae* 10/99 II
PHAGE_Salmon_Fels_2_NC_010463	Gammaproteobacteria	*Salmonella typhimurium* LT2
PROPHAGE_Xantho_306	Gammaproteobacteria	*Xanthomonas axonopodis* pv. citri str. 306
PROPHAGE_Xantho_306	Gammaproteobacteria	*Xanthomonas axonopodis* pv. citri str. 306
PROPHAGE_Xantho_306	Gammaproteobacteria	*Xanthomonas axonopodis* pv. citri str. 306

Supplemental Table S4. Efflux pumps obtained from *M. cysteinexigens* B1-EB^T, Mc-AG77, and *B. rhizoxinica* HKI 454^T genomes.

B1-EB^T		Mc-AG77*	HKI454^T	
**Gene**	**Annotation**	**Gene**	**Gene**	**Annotation**
-	Membrane-fusion protein	ctg1_396	RBRH_01297	Acriflavin resistance periplasmic protein
-	Cation/multidrug efflux pump	ctg1_395	RBRH_01296	Acriflavin resistance plasma membrane protein
-	Outer membrane protein	ctg1_394	RBRH_01295	Type I secretion outer membrane protein
-	Uncharacterized lipoprotein	ctg1_1413	RBRH_03796	Acriflavin resistance periplasmic protein
-	Membrane-fusion protein	-	RBRH_03795	Acriflavin resistance plasma membrane protein
-	Cation/multidrug efflux pump	ctg1_1411, ctg1_1412	RBRH_03794	Acriflavin resistance plasma membrane protein
-	ABC-type multidrug transport system, ATPase and permease components	ctg1_402	RBRH_02428	Multidrug resistance ABC transporter ATP-binding and permease protein
-	Predicted NTPase (NACHT family)	ctg1_1945	RBRH_03778	Multidrug resistance ABC transporter ATP-binding and permease protein
-	Permeases of the major facilitator superfamily	ctg1_683	RBRH_02122	Transporter, MFS superfamily

* Genes listed in Mc-AG77 are predicted from the homologuous sequence of B1-EB^T genome

Supplemental Table S5. Comparison of the Sec pathway exported proteins from *M. cysteinexigens* B1-EB^T, Mc-AG77, and *B. rhizoxinica* HKI 454^T genomes.

B1-EB^T		Mc-AG77*	HKI454^T	
**Gene**	**Annotation**	**Gene**	**Gene**	**Annotation**
-	Type IIA topoisomerase (DNA gyrase/topo II, topoisomerase IV), A subunit	ctg1_1372	RBRH_00055	Outer membrane protein
gph	2-polyprenyl-3-methyl-5-hydroxy-6-metoxy-1,4-benzoquinol methylase	ctg1_1370	RBRH_00057	Phosphoglycolate phosphatase
RPSI07_mp1112	3-methyladenine DNA glycosylase	ctg1_17, ctg1_199, ctg1_2116	RBRH_00100	Hemagglutinin-like protein
RSp1073	Thymidylate kinase	ctg1_17, ctg1_199, ctg1_2116	RBRH_00100	Hemagglutinin-like protein
-	Uncharacterized conserved protein	ctg1_1306	RBRH_00169	Transcriptional regulator, GntR family
-	Metal-dependent hydrolase	ctg1_988	RBRH_00365	Ferredoxin--NADP reductase
RSc2452	ABC-type dipeptide transport system, periplasmic component	ctg1_772, ctg1_1149	RBRH_00366	Endonuclease
gspE	Type II secretory pathway, ATPase PulE/Tfp pilus assembly pathway, ATPase PilB	ctg1_810	RBRH_00781	General secretion pathway protein E
gspD	Type II secretory pathway, component PulD	ctg1_811	RBRH_00782	General secretion pathway protein
-	ABC-type branched-chain amino acid transport systems, periplasmic component	ctg1_822	RBRH_00840	Leucine-, isoleucine-, valine-, threonine-, and alanine-binding protein
-	FKBP-type peptidyl-prolyl cis-trans isomerases 2	ctg1_1073	RBRH_00922	DNA repair protein radC
lpxC	Uncharacterized conserved protein	-	RBRH_00949	UDP-3-O-[3-hydroxymyristoyl] N-acetylglucosamine deacetylase
-	Uncharacterized protein conserved in bacteria	ctg1_1974	RBRH_00982	Hypothetical exported protein
-	Uncharacterized protein related to capsule biosynthesis enzymes	ctg1_1976	RBRH_00984	Putative periplasmic protein
argB	Homoserine acetyltransferase	ctg1_890	RBRH_00991	Acetylglutamate kinase
-	Zn-dependent protease with chaperone function	ctg1_893	RBRH_00996	AmpG protein
spoT	Guanosine polyphosphate pyrophosphohydrolases/synthetases	ctg1_442	RBRH_01008	GTP pyrophosphokinase / Guanosine- 3',5'-bis(Diphosphate) 3'-pyrophosphohydrolase
-	ABC-type phosphate transport system, permease component	ctg1_1112	RBRH_01097	Phosphate-binding protein
folP	Phosphomannomutase	ctg1_1106	RBRH_01099	Dihydropteroate synthase
-	Signal transduction histidine kinase involved in nitrogen fixation and metabolism regulation	ctg1_870	RBRH_01265	Hypothetical protein
-	D-alanyl-D-alanine carboxypeptidase (penicillin-binding protein 4)	ctg1_1214	RBRH_01300	Hypothetical protein
BURMUCGD1_0587	Uncharacterized protein conserved in bacteria	ctg1_1216	RBRH_01305	Esterase
purC	Phosphoribosylaminoimidazole carboxylase (NCAIR synthetase)	ctg1_1206	RBRH_01308	Phosphoribosylaminoimidazole carboxylase carboxyltransferase subunit
-	Cytochrome c553	ctg1_323	RBRH_01337	Cytochrome c-554(548)
-	Cytochrome c553	ctg1_324	RBRH_01338	Cytochrome c-554(548)
pyrB	Aspartate carbamoyltransferase, catalytic chain	ctg1_414	RBRH_01477	Aspartate carbamoyltransferase
-	Outer membrane protein and related peptidoglycan-associated (lipo)proteins	ctg1_1342	RBRH_01525	Tol system periplasmic component YbgF
tolB	Hypothetical protein	ctg1_1344	RBRH_01528	TolB protein
glyA	Predicted transcriptional regulator, consists of a Zn-ribbon and ATP-cone domains	ctg1_1349	RBRH_01534	Serine hydroxymethyltransferase
CAGGBEG34_200100	Uncharacterized protein conserved in bacteria	ctg1_405	RBRH_01545	Hypothetical exported protein
-	Intracellular septation protein A	ctg1_1785	RBRH_01601	Peptide methionine sulfoxide reductase msrB
miaA	DNA mismatch repair enzyme (predicted ATPase)	ctg1_998	RBRH_01633	tRNA delta(2)-Isopentenylpyrophosphate transferase
CAGGBEG34_290002	Uncharacterized paraquat-inducible protein A	ctg1_1184	RBRH_01670	Paraquat-inducible protein B
-	ABC-type metal ion transport system, permease component	ctg1_91	RBRH_01730	ABC transporter substrate-binding protein
engC	FOG: TPR repeat	ctg1_78	RBRH_01746	GTPase
fumC	Fumarase	ctg1_827	RBRH_01757	Aldose 1-dehydrogenase
-	Nucleotidyltransferase/DNA polymerase involved in DNA repair	-	RBRH_01768	Unnamed protein product
-	Ribosomal protein L21	ctg1_1257	RBRH_01990	Cytochrome c4
gltJ	ABC-type amino acid transport system, permease component	ctg1_506	RBRH_02078	Glutamate/aspartate transport system permease protein gltJ
-	ABC-type amino acid transport/signal transduction systems, periplasmic component/domain	ctg1_507	RBRH_02079	Glutamate/aspartate-binding protein
gdhA	Glutamate dehydrogenase/leucine dehydrogenase	ctg1_508	RBRH_02080	Glutamate dehydrogenase
BC1002_0370	Uncharacterized protein conserved in bacteria	ctg1_136	RBRH_02101	Hypothetical protein
queF	Enzyme related to GTP cyclohydrolase I	ctg1_142	RBRH_02109	Queuosine biosynthesis protein QueF
-	FOG: TPR repeat	ctg1_196	RBRH_02146	Tetratricopeptide repeat family protein
prsA	Phosphoribosylpyrophosphate synthetase	ctg1_193	RBRH_02149	Ribose-phosphate pyrophosphokinase
prsA	Phosphoribosylpyrophosphate synthetase	ctg1_193	RBRH_02149	Ribose-phosphate pyrophosphokinase
-	ABC-type metal ion transport system, periplasmic component	ctg1_91	RBRH_02176	ABC transporter substrate-binding protein
-	Heme/copper-type cytochrome/quinol oxidase, subunit 3	ctg1_1038	RBRH_02186	Cytochrome c oxidase polypeptide I
coxB	Heme/copper-type cytochrome/quinol oxidases, subunit 1	ctg1_1037	RBRH_02187	Cytochrome c oxidase polypeptide II
-	Cytochrome c1	ctg1_544	RBRH_02274	Cytochrome c1
-	Trypsin-like serine proteases, typically periplasmic, contain C-terminal PDZ domain	ctg1_548	RBRH_02279	Endopeptidase degP
BURK_006002	ABC-type transport system involved in resistance to organic solvents, auxiliary component	ctg1_567	RBRH_02300	Toluene transport system Ttg2D protein
PMI06_006043	Outer membrane protein W	ctg1_2340	RBRH_02318	Outer membrane protein
BamMEX5DRAFT_2889	Thiol:disulfide interchange protein	ctg1_591	RBRH_02340	Thiol:disulfide interchange protein DsbD
Bcep18194_A3475	Superfamily I DNA and RNA helicases	ctg1_2141	RBRH_02341	Periplasmic divalent cation tolerance protein CutA
trpD	Anthranilate phosphoribosyltransferase	ctg1_527	RBRH_02386	Anthranilate phosphoribosyltransferase
msbA	Lipid A core - O-antigen ligase and related enzymes	ctg1_2172	RBRH_02470	Phospholipid-lipopolysaccharide ABC transporter
hemF	Nicotinic acid mononucleotide adenylyltransferase	ctg1_2168	RBRH_02486	Coproporphyrinogen III oxidase
ilvB	Acetolactate synthase, small (regulatory) subunit	ctg1_1965	RBRH_02508	Acetolactate synthase large subunit
rpsO	Polyribonucleotide nucleotidyltransferase (polynucleotide phosphorylase)	ctg1_1435	RBRH_02518	SSU ribosomal protein S15P
-	Na+/H+ antiporter NhaD and related arsenite permeases	ctg1_479	RBRH_02593	Hypothetical membrane spanning protein
-	Periplasmic serine proteases (ClpP class)	ctg1_309	RBRH_02608	Signal peptide peptidase sppA
-	Trypsin-like serine proteases, typically periplasmic, contain C-terminal PDZ domain	ctg1_292	RBRH_02624	Endopeptidase degP
-	ABC-type dipeptide/oligopeptide/nickel transport systems, permease components	ctg1_937	RBRH_02731	Dipeptide transport system permease protein dppC
-	ABC-type dipeptide/oligopeptide/nickel transport systems, permease components	ctg1_936	RBRH_02732	Dipeptide transport ATP-binding protein dppD
-	Hypothetical protein	ctg1_35	RBRH_02803	Potassium-transporting ATPase A chain
-	Uncharacterized protein conserved in bacteria	ctg1_35	RBRH_02803	Potassium-transporting ATPase A chain
-	Uncharacterized protein conserved in bacteria	ctg1_35	RBRH_02803	Potassium-transporting ATPase A chain
-	K+-transporting ATPase, A chain	-	RBRH_02840	Outer membrane porin protein 32 precursor
-	ABC-type amino acid transport/signal transduction systems, periplasmic component/domain	ctg1_700	RBRH_02850	Histidine-binding protein
-	Hypothetical protein	ctg1_400	RBRH_02876	34 kDa membrane antigen precursor
-	Cell wall-associated hydrolases (invasion-associated proteins)	ctg1_1804	RBRH_02890	Oligopeptide transport ATP-binding protein OppD/ Oligopeptide transport ATP-binding protein OppF
-	ABC-type Fe^{3+} transport system, periplasmic component	ctg1_1805	RBRH_02891	NlpC/P60 family protein

aroC	Large exoproteins involved in heme utilization or adhesion	ctg1_1905	RBRH_02955	Chorismate synthase
BgramDRAFT_1585	ABC-type dipeptide transport system, periplasmic component	ctg1_960	RBRH_03115	Thiol:disulfide interchange protein dsbA
K788_4134	Thiol-disulfide isomerase and thioredoxins	ctg1_959	RBRH_03116	Cell division protein ftsN
K788_8069	Aspartate 1-decarboxylase	ctg1_1923	RBRH_03137	Cobalamin-binding protein
-	Uncharacterized protein conserved in bacteria	ctg1_716	RBRH_03189	Surface protein
-	Phosphatidylglycerophosphatase A and related proteins	ctg1_720	RBRH_03197	Phosphatidylglycerophosphatase A
mpl	Exoribonuclease R	ctg1_1296	RBRH_03203	UDP-N-acetylmuramate:L-alanyl-gamma-D-glutamyl-meso-diaminopimelate ligase
-	Sugar kinases, ribokinase family	ctg1_1271	RBRH_03214	Outer membrane lipoprotein
nrdB	Hypothetical protein	ctg1_1269	RBRH_03217	Ribonucleoside-diphosphate reductase beta chain
RBRH_03270	Ubiquinone biosynthesis protein COQ7	ctg1_1118	RBRH_03270	Outer membrane porin protein 32 precursor
mviN	Adenylate kinase and related kinases	ctg1_1016	RBRH_03318	Virulence factor mviN
h16_A0588	Uncharacterized protein conserved in bacteria	ctg1_1009	RBRH_03325	Hypothetical membrane spanning protein
xerD	Inorganic pyrophosphatase	ctg1_1006	RBRH_03335	Integrase/recombinase (XerD/RipX family)
-	Outer membrane protein/protective antigen OMA87	ctg1_60	RBRH_03566	Outer membrane protein Omp
glnD	NAD-dependent DNA ligase (contains BRCT domain type II)	ctg1_126	RBRH_03580	[protein-PII] uridylyltransferase
arnT	4-amino-4-deoxy-L-arabinose transferase and related glycosyltransferases of PMT family	ctg1_461	RBRH_03677	Glucosyltransferase
BUPH_06600	Transcriptional regulator	ctg1_1663	RBRH_03701	Unnamed protein product
purA	ATP phosphoribosyltransferase involved in histidine biosynthesis	ctg1_174	RBRH_03721	Adenylosuccinate synthetase
BH160DRAFT_6132	Uncharacterized protein conserved in bacteria	ctg1_181	RBRH_03730	Hypothetical protein
ispG	Enzyme involved in the deoxyxylulose pathway of isoprenoid biosynthesis	ctg1_183	RBRH_03732	1-hydroxy-2-methyl-2-(E)-butenyl 4-diphosphate synthase
-	Membrane proteins related to metalloendopeptidases	ctg1_424	RBRH_03742	Peptidoglycan-specific endopeptidase, M23 family
-	Cation/multidrug efflux pump	ctg1_1411, ctg1_1412	RBRH_03794	Acriflavin resistance plasma membrane protein
mdtB	Membrane-fusion protein	-	RBRH_03795	Acriflavin resistance plasma membrane protein
BYI23_A017700	ABC-type transport system, involved in lipoprotein release, permease component	ctg1_1465	RBRH_03837	Regulatory protein
phrB	Predicted GTPase	ctg1_951	RBRH_03918	Deoxyribodipyrimidine photolyase
ppiB	FOG: TPR repeat	ctg1_2008	RBRH_03963	Peptidyl-prolyl cis-trans isomerase
-	Predicted NTPase (NACHT family)	ctg1_2003	RBRH_03966	RRNA methylase, SpoU family
mutS	Uncharacterized conserved small protein	ctg1_1873	RBRH_03971	DNA mismatch repair protein mutS

* Genes listed in Mc-AG77 are predicted from the homologuous sequence of B1-EB^{T} genome

Supplemental Table S6. Type III secretion system components derived from *M. cysteinexigens* B1-EB^T, Mc-AG77, and *B. rhizoxinica* HKI 454^T genomes.

B1-EB^T		Mc-AG77*	HKI454^T	
**Gene**	**Annotation**	**Gene**	**Gene**	**Annotation**
-	Cell wall-associated hydrolases (invasion-associated proteins)	ctg1_1804	RBRH_02890	Oligopeptide transport ATP-binding protein OppD/ Oligopeptide transport ATP-binding protein OppF
-	Uncharacterized conserved protein	ctg1_1306	RBRH_00169	Transcriptional regulator, GntR family
glnD	NAD-dependent DNA ligase (contains BRCT domain type II)	ctg1_1306	RBRH_03580	[protein-PII] uridylyltransferase
-	Gamma-aminobutyrate permease and related permeases	ctg1_1172	RBRH_00422	Lysine 2,3-aminomutase
-	Predicted NTPase (NACHT family)	ctg1_2003	RBRH_03966	RRNA methylase, SpoU family
gspE	Type II secretory pathway, ATPase PulE/Tfp pilus assembly pathway, ATPase PilB	ctg1_810	RBRH_00781	General secretion pathway protein E
purA	ATP phosphoribosyltransferase involved in histidine biosynthesis	ctg1_174	RBRH_03721	Adenylosuccinate synthetase
arnT	4-amino-4-deoxy-L-arabinose transferase and related glycosyltransferases of PMT family	ctg1_461	RBRH_03677	Glucosyltransferase
fumC	Fumarase	ctg1_827	RBRH_01757	Fumarate hydratase
-	Uncharacterized protein conserved in bacteria	ctg1_716	RBRH_03189	surface protein
BC1002_0370	Uncharacterized protein conserved in bacteria	ctg1_136	RBRH_02101	Hypothetical protein
-	Nucleotidyltransferase/DNA polymerase involved in DNA repair	-	RBRH_01356	DNA polymerase III alpha subunit
miaA	DNA mismatch repair enzyme (predicted ATPase)	ctg1_998	RBRH_01633	tRNA delta(2)-Isopentenylpyrophosphate transferase
aroC	Large exoproteins involved in heme utilization or adhesion	ctg1_1905	RBRH_02955	Chorismate synthase
xerD	Inorganic pyrophosphatase	ctg1_1006	RBRH_03335	Integrase/recombinase (XerD/RipX family)
-	K+-transporting ATPase, A chain	ctg1_34	RBRH_02804	Potassium-transporting ATPase B chain
gdhA	Glutamate dehydrogenase/leucine dehydrogenase	ctg1_508	RBRH_02080	Glutamate dehydrogenase
gph	2-polyprenyl-3-methyl-5-hydroxy-6-metoxy-1,4-benzoquinol methylase	ctg1_147	RBRH_00057	type III secretion inner membrane protein SctR
queF	Enzyme related to GTP cyclohydrolase I	ctg1_142	RBRH_02109	Queuosine biosynthesis protein QueF
ilvB	Acetolactate synthase, small (regulatory) subunit	ctg1_1965	RBRH_02508	Acetolactate synthase large subunit
-	FKBP-type peptidyl-prolyl cis-trans isomerases 2	ctg1_1073	RBRH_00922	DNA repair protein radC
-	Metal-dependent hydrolase	ctg1_988	RBRH_00365	Ferredoxin--NADP reductase
folP	Phosphomannomutase	ctg1_1106	RBRH_01099	Dihydropteroate synthase
-	Zn-dependent protease with chaperone function	ctg1_893	RBRH_00996	AmpG protein
-	ABC-type dipeptide/oligopeptide/nickel transport systems, permease components	ctg1_936	RBRH_02732	Dipeptide transport ATP-binding protein dppD
hemF	Nicotinic acid mononucleotide adenylyltransferase	ctg1_2168	RBRH_02486	Coproporphyrinogen III oxidase
ppiB	FOG: TPR repeat	ctg1_2008	RBRH_03963	Peptidyl-prolyl cis-trans isomerase
-	Phosphatidylglycerophosphatase A and related proteins	ctg1_720	RBRH_03197	Phosphatidylglycerophosphatase A
pyrB	Aspartate carbamoyltransferase, catalytic chain	ctg1_414	RBRH_01477	Aspartate carbamoyltransferase
-	Periplasmic serine proteases (ClpP class)	ctg1_309	RBRH_02608	Signal peptide peptidase sppA
msbA	Lipid A core - O-antigen ligase and related enzymes	ctg1_2172	RBRH_02470	Phospholipid-lipopolysaccharide ABC transporter
argB	Homoserine acetyltransferase	ctg1_890	RBRH_00991	Acetylglutamate kinase
-	Membrane proteins related to metalloendopeptidases	ctg1_424	RBRH_03742	Peptidoglycan-specific endopeptidase, M23 family
trpD	Anthranilate phosphoribosyltransferase	ctg1_527	RBRH_02386	Anthranilate phosphoribosyltransferase
mutS	Uncharacterized conserved small protein	ctg1_1873	RBRH_03971	DNA mismatch repair protein mutS
phrB	Predicted GTPase	ctg1_951	RBRH_03918	Deoxyribodipyrimidine photolyase
ispG	Enzyme involved in the deoxyxylulose pathway of isoprenoid biosynthesis	ctg1_183	RBRH_03732	1-hydroxy-2-methyl-2-(E)-butenyl 4-diphosphate synthase
gltJ	ABC-type amino acid transport system, permease component	ctg1_506	RBRH_02078	Glutamate/aspartate transport system permease protein gltJ
dxs	Geranylgeranyl pyrophosphate synthase	ctg1_2092	RBRH_03430	1-Deoxy-D-xylulose 5-phosphate synthase
BURMUCGD1_0587	Uncharacterized protein conserved in bacteria	ctg1_1216	RBRH_01305	Esterase
sms	Alanine racemase	ctg1_2152	RBRH_00325	DNA repair protein RadA
BH160DRAFT_6132	Uncharacterized protein conserved in bacteria	ctg1_181	RBRH_03730	Hypothetical protein
engC	FOG: TPR repeat	ctg1_78	RBRH_01746	GTPase
-	ABC-type dipeptide/oligopeptide/nickel transport systems, permease components	ctg1_937	RBRH_02731	Dipeptide transport system permease protein dppC
prsA	Phosphoribosylpyrophosphate synthetase	ctg1_193	RBRH_02149	Ribose-phosphate pyrophosphokinase
RPSI07_mp1112	3-methyladenine DNA glycosylase	ctg1_17, ctg1_199, ctg1_2116	RBRH_00100	Hemagglutinin-like protein
CAGGBEG34_290002	Uncharacterized paraquat-inducible protein A	ctg1_1184	RBRH_01670	Paraquat-inducible protein B
rpsO	Polyribonucleotide nucleotidyltransferase (polynucleotide phosphorylase)	ctg1_1435	RBRH_02518	SSU ribosomal protein S15P
nrdB	Hypothetical protein	ctg1_1269	RBRH_03217	Ribonucleoside-diphosphate reductase beta chain
-	Intracellular septation protein A	ctg1_1785	RBRH_01601	Peptide methionine sulfoxide reductase msrB
purC	Phosphoribosylaminoimidazole carboxylase (NCAIR synthetase)	ctg1_1206	RBRH_01308	Phosphoribosylaminoimidazole carboxylase carboxyltransferase subunit
-	Heme/copper-type cytochrome/quinol oxidase, subunit 3	ctg1_1038	RBRH_02186	Cytochrome c oxidase polypeptide I
glyA	Predicted transcriptional regulator, consists of a Zn-ribbon and ATP-cone domains	ctg1_1349	RBRH_01534	Serine hydroxymethyltransferase
RSp1073	Thymidylate kinase	ctg1_17, ctg1_199, ctg1_2116	RBRH_00100	Hemagglutinin-like protein

* Genes listed in Mc-AG77 are predicted from the homologous sequence of B1-EB^T genome

Supplemental Table S7. Comparison of metabolic potentials, nutrients uptake and defense responses in B1-EB^T, Mc-AG77 and HKI 454^T.

Attributes	B1-EB^T	Mc-AG77	HKI454^T
**Primary metabolism**			
Gluconeogenesis	–	–	RBRH_00086, RBRH_01311, RBRH_01407, RBRH_03119
Ethanol assimilation	–	–	RBRH_01957, RBRH_02691, RBRH_03108, RBRH_03107, RBRH_00946, RBRH_00316, RBRH_00393
Import of organic acids	–	–	RBRH_00074, RBRH_00570, RBRH_00171, RBRH_00170, RBRH_02513, RBRH_01475, RBRH_02105, RBRH_00014, RBRH_02435, RBRH_03529, RBRH_00215
Dipeptide and oligopeptide import system	MCB1EB_2025, MCB1EB_2209, MCB1EB_2249, MCB1EB_2250, MCB1EB_2251, MCB1EB_1684, MCB1EB_1241	ctg1_1149, ctg1_960, ctg1_937, ctg1_936, ctg1_935, ctg1_1449, ctg1_1800	RBRH_03113, RBRH_02733, RBRH_02732, RBRH_02731, RBRH_02730, RBRH_02729, RBRH_02888, RBRH_02889, RBRH_02890, RBRH_02887
**Cofactor biosynthesis**			
Biosynthesis of pyridoxin, heme, flavin, biotin and thiamine	MCB1EB_152, MCB1EB_467, MCB1EB_519, MCB1EB_565, MCB1EB_654, MCB1EB_719, MCB1EB_1949, MCB1EB_949, MCB1EB_112, MCB1EB_1169, MCB1EB_1172, MCB1EB_1922, MCB1EB_2131, MCB1EB_2132, MCB1EB_2133, MCB1EB_1169, MCB1EB_149, MCB1EB_151, MCB1EB_48, MCB1EB_1901, MCB1EB_1902, MCB1EB_2248, MCB1EB_993, MCB1EB_995, MCB1EB_1115, MCB1EB_1357, MCB1EB_1394, MCB1EB_2142,	ctg1_705, ctg1_467, ctg1_415, ctg1_375, ctg1_278, ctg1_197, ctg1_2155, ctg1_1242, ctg1_1229, ctg1_981, ctg1_951, ctg1_729, ctg1_17, ctg1_199, ctg1_2116, ctg1_1954, ctg1_1953, ctg1_1256, ctg1_1039, ctg1_1038, ctg1_1037, ctg1_2098, ctg1_2096, ctg1_1965, ctg1_1706, ctg1_1668, ctg1_1027, ctg1_708, ctg1_706, ctg1_1825, ctg1_818, ctg1_817, ctg1_1276, ctg1_1275, ctg1_1264, ctg1_982, ctg1_939	RBRH_02443, RBRH_01081, RBRH_02335, RBRH_02334, RBRH_02178, RBRH_02179, RBRH_00935, RBRH_03796, RBRH_03795, RBRH_03794, RBRH_01414, RBRH_01296, RBRH_01297, RBRH_03209, RBRH_03208, RBRH_02661, RBRH_03392, RBRH_04087, RBRH_02226, RBRH_03100, RBRH_03099, RBRH_03196, **pBRH01-** RBRH_03434, RBRH_02982, RBRH_02981, RBRH_02980, RBRH_00528, RBRH_02772, RBRH_03038
**Membrane transport**			
Putative cobalamin transport system	MCB1EB_1332, MCB1EB_1334, MCB1EB_2205	ctg1_1734, ctg1_1732, ctg1_964	RBRH_01656, RBRH_03137, RBRH_01135, RBRH_01971,
Fe^{2+}, Mg^{2+}, Co^{2+}, Zn^{2+}, K^{+} uptake system	MCB1EB_271, MCB1EB_1926, MCB1EB_216, MCB1EB_351, MCB1EB_1926, MCB1EB_541, MCB1EB_543, MCB1EB_761, MCB1EB_2006, MCB1EB_550, MCB1EB_2006, MCB1EB_743, MCB1EB_891, MCB1EB_892, MCB1EB_893, MCB1EB_895, MCB1EB_1641, MCB1EB_1953,	ctg1_581, ctg1_1251, ctg1_642, ctg1_523, ctg1_1251, ctg1_398, ctg1_400, ctg1_149, ctg1_1164, ctg1_390, ctg1_1164, ctg1_173, ctg1_35, ctg1_34, ctg1_33, ctg1_31, ctg1_1834, ctg1_1225	RBRH_03162, RBRH_02008, RBRH_02210, RBRH_03237, RBRH_02878, RBRH_02719, RBRH_01747, RBRH_02688, RBRH_01133, RBRH_01325, RBRH_02015, RBRH_03110, RBRH_03075, RBRH_03050, RBRH_03420, RBRH_00997, RBRH_00785, RBRH_03719, RBRH_02130, **pBRH01-** RBRH_00461, RBRH_02819, RBRH_02805, RBRH_02804, RBRH_02803
Na^{+}/Ca^{2+} antiporter	MCB1EB_455, MCB1EB_1707,	ctg1_479, ctg1_1426	RBRH_00006, RBRH_00005, RBRH_02842, RBRH_01432
Na^{+}/citrate symporter	MCB1EB_449, MCB1EB_476, MCB1EB_921, MCB1EB_981, MCB1EB_1145, MCB1EB_1275, MCB1EB_1417, MCB1EB_1529, MCB1EB_1583, MCB1EB_1878, MCB1EB_2020, MCB1EB_2313	ctg1_1584, ctg1_1647, ctg1_1817, ctg1_457, ctg1_930, ctg1_1933, ctg1_8, ctg1_1300, ctg1_2108, ctg1_858	RBRH_03087, RBRH_01674, RBRH_00074, **pBRH01-** RBRH_00570
NRPS/PKS system for rhizoxin biosynthesis	–	–	RBRH_02642, RBRH_04173, RBRH_02641, RBRH_03984, RBRH_04313, RBRH_03867, RBRH_01506, RBRH_04125, RBRH_00209, RBRH_04061, rhiE, rhiD, rhiC, rhiB, rhiA, **pBRH01-** RBRH_04314, RBRH_02787, RBRH_04279, RBRH_01504, RBRH_00423, RBRH_00429, RBRH_04227, RBRH_00452, RBRH_00484, RBRH_00572, RBRH_00578, RBRH_00622, RBRH_04248, RBRH_00623, RBRH_00274, RBRH_00260, RBRH_00213, RBRH_01792, RBRH_04314, RBRH_02787, RBRH_04279
Lantibibiotic biosynthesis	–	–	**pBRH01-** RBRH_00226
Chitinolytic enzymes and chitin-binding proteins	–	–	RBRH_01547, RBRH_02666
**Amino acid metabolism**			
Branched-chain amino acid	MCB1EB_30, MCB1EB_44, MCB1EB_1194, MCB1EB_1971, MCB1EB_2090, MCB1EB_2091, MCB1EB_2092, MCB1EB_2093, MCB1EB_2178, MCB1EB_2294	ctg1_822, ctg1_837, ctg1_1876, ctg1_1202, ctg1_1079, ctg1_1078, ctg1_1077, ctg1_1076, ctg1_991, ctg1_876	RBRH_00809, RBRH_00810, RBRH_04065, RBRH_00813, RBRH_00915, RBRH_00914, RBRH_00913, RBRH_00912, RBRH_01064, RBRH_01063, RBRH_01062, RBRH_02655, RBRH_02654, RBRH_02653, RBRH_02652, RBRH_01316, RBRH_01317, RBRH_01318, **pBRH01**-RBRH_00502, RBRH_00503
Aromatic amino acid	MCB1EB_291, MCB1EB_474, MCB1EB_487, MCB1EB_1007, MCB1EB_1038	ctg1_560, ctg1_459, ctg1_447, ctg1_2084, ctg1_2047	RBRH_02874, **pBRH01**-RBRH_00490, RBRH_00491
Histidine	MCB1EB_154, MCB1EB_155, MCB1EB_607, MCB1EB_741, MCB1EB_815, MCB1EB_895, MCB1EB_1032, MCB1EB_1188, MCB1EB_1387	ctg1_703, ctg1_702, ctg1_330, ctg1_175, ctg1_99, ctg1_31, ctg1_2053, ctg1_1887, ctg1_1676	RBRH_03722, RBRH_03800, RBRH_01405, RBRH_04111, RBRH_01406, RBRH_01301, RBRH_00797, RBRH_02293, RBRH_02292, RBRH_02850, RBRH_02858, RBRH_03731, **pBRH01-** RBRH_02789, RBRH_00406
Glutamate/ aspartate	MCB1EB_91, MCB1EB_153, MCB1EB_165, MCB1EB_207, MCB1EB_367, MCB1EB_1216, MCB1EB_1857, MCB1EB_1916, MCB1EB_2044, MCB1EB_2274, MCB1EB_360, MCB1EB_474, MCB1EB_487, MCB1EB_507, MCB1EB_520, MCB1EB_849, MCB1EB_91, MCB1EB_1038, MCB1EB_1070, MCB1EB_1155, MCB1EB_1776, MCB1EB_1863, MCB1EB_2099	ctg1_748, ctg1_704, ctg1_692, ctg1_651, ctg1_508, ctg1_1854, ctg1_1322, ctg1_1261, ctg1_1129, ctg1_890, ctg1_515, ctg1_459, ctg1_447, ctg1_425, ctg1_414, ctg1_69, ctg1_2047, ctg1_2016, ctg1_1924, ctg1_1362, ctg1_1316, ctg1_1070	RBRH_03931, RBRH_00189, RBRH_01412, RBRH_02075, RBRH_02077, RBRH_02078, RBRH_02079, RBRH_02080, RBRH_00991, RBRH_00010, RBRH_00003, RBRH_03261, RBRH_03258, RBRH_03246, RBRH_03230, RBRH_01650, RBRH_02313, RBRH_02312,

Glycine	MCB1EB_934, MCB1EB_996, MCB1EB_1251, MCB1EB_1258, MCB1EB_1680, MCB1EB_1789, MCB1EB_1948, MCB1EB_1949, MCB1EB_1950, MCB1EB_2027	ctg1_2176, ctg1_2095, ctg1_1784, ctg1_1782, ctg1_1453, ctg1_1349, ctg1_1229, ctg1_1228, ctg1_1227, ctg1_1146	RBRH_00844, RBRH_00845, RBRH_02308, RBRH_02487, **pBRH01-** RBRH_02814, RBRH_02813, RBRH_02811, RBRH_03481
Citrate	MCB1EB_1110, MCB1EB_1864, MCB1EB_1865, MCB1EB_2144, MCB1EB_2313	ctg1_1970, ctg1_1314, ctg1_1315, ctg1_1025, ctg1_858	RBRH_03108, RBRH_03107, RBRH_01674, RBRH_00946, RBRH_00074, RBRH_00175, RBRH_00164, **pBRH01**-RBRH_00392, RBRH_00393, RBRH_00570, RBRH_00316, RBRH_00306, RBRH_02824,
Efflux systems for Arg, His, Lys	MCB1EB_458, MCB1EB_546, MCB1EB_1195, MCB1EB_1721, MCB1EB_1722	ctg1_476, ctg1_395, ctg1_1875, ctg1_1411, ctg1_1412	RBRH_03883, RBRH_02157, RBRH_03122, RBRH_00102, RBRH_02499, **pBRH01-** RBRH_04222, RBRH_00422, RBRH_03457
Efflux systems for cysteine	–	–	RBRH_03826, RBRH_02205, RBRH_02239, RBRH_01971, RBRH_00010, RBRH_00003, RBRH_00938, RBRH_01725, RBRH_01073, **pBRH01-** RBRH_03440, RBRH_02988
**Polar lipids**			
Phosphatidylethanolamine	MCB1EB_470, MCB1EB_1043, MCB1EB_1111, MCB1EB_1112, MCB1EB_2025	ctg1_463, ctg1_2042, ctg1_1969, ctg1_1968, ctg1_1149	–
Phosphatidylglycerol	MCB1EB_470, MCB1EB_662, MCB1EB_1043, MCB1EB_2025	ctg1_463, ctg1_271, ctg1_2042, ctg1_1149	–
Aminophospholipid	MCB1EB_635, MCB1EB_1957	ctg1_300, ctg1_1216	–
**Fatty acid synthesis**			
Acetyl-CoA carboxylase components	MCB1EB_1077, MCB1EB_1858, MCB1EB_2247	ctg1_2012, ctg1_1321, ctg1_939	RBRH_03208
Peripheral enzymes (ACP synthase and Biotin ligase)	MCB1EB_655, MCB1EB_2247	ctg1_277, ctg1_939	**pBRH01-** RBRH_02776
Glycerophospholipid metabolism	MCB1EB_1970, MCB1EB_2140, MCB1EB_2191, MCB1EB_1090,	ctg1_1158, ctg1_1029, ctg1_978, ctg1_1992	RBRH_01314, RBRH_02197, RBRH_01278, RBRH_02322, **pBRH01-** RBRH_03529
**Lipopolysaccharide gene cluster (LPSs)**			
Lipopolysaccharide (LPS)	–	–	RBRH_02470, waaC, RBRH_01946, **pBRH01-** RBRH_00221
O-antigen	–	–	waaL, RBRH_01327,
Inner membrane ABC transporter, MsbA	MCB1EB_929	ctg1_2172	–
Transport complex (LptA, LptAB, LptC, LptD, LptE, LptG)	–	–	RBRH_01445, RBRH_00521, RBRH_03824,
**Expo/ capsular polysaccharide (EPS) biosynthesis**			
Antigen flippase and polymerase	–	–	RBRH_03897, wzx
Trans-envelope transport complex (Wza/Wzb/Wzc)	–	–	RBRH_00520, RBRH_00521, RBRH_00522
Glycosyltransferase gene	MCB1EB_466, MCB1EB_472, MCB1EB_540, MCB1EB_926, MCB1EB_927, MCB1EB_1270, MCB1EB_1781, MCB1EB_2100, MCB1EB_2105, MCB1EB_2304	ctg1_468, ctg1_461, ctg1_401, ctg1_2175, ctg1_2174, ctg1_1772, ctg1_1357, ctg1_1069, ctg1_1064, ctg1_866	wbxA, wbiF, RBRH_01468, RBRH_03053, RBRH_02249, RBRH_00363, RBRH_01942, RBRH_02426, RBRH_01558, RBRH_01575, RBRH_03887, RBRH_03896, **pBRH01-** RBRH_02977, RBRH_00433, RBRH_00435

Fig. S1A-1
Sharmin, D., et al

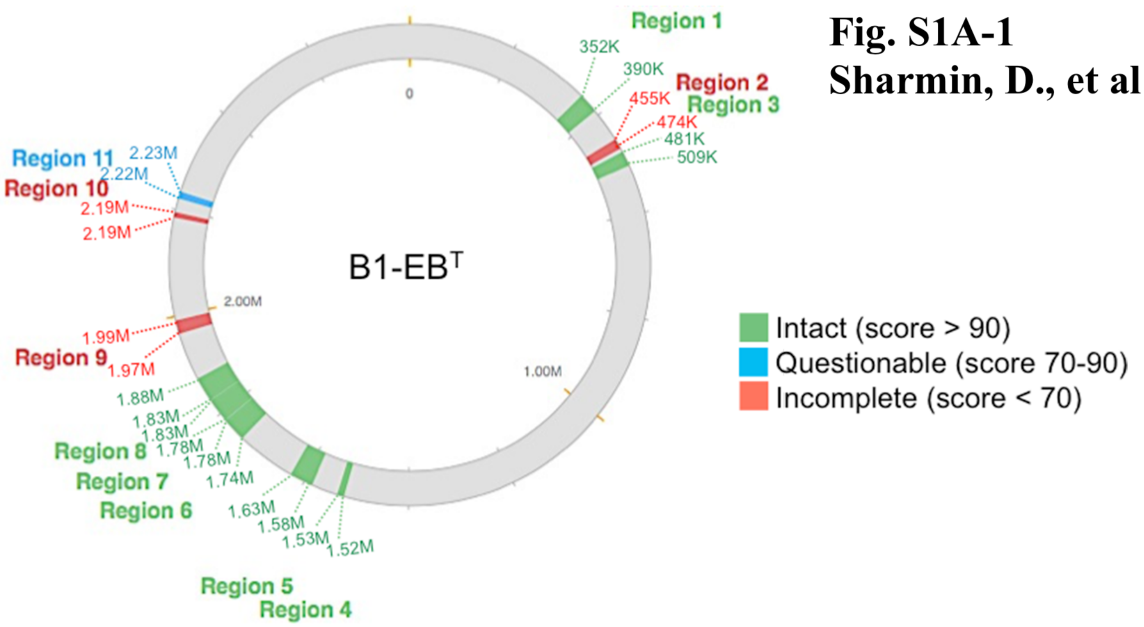

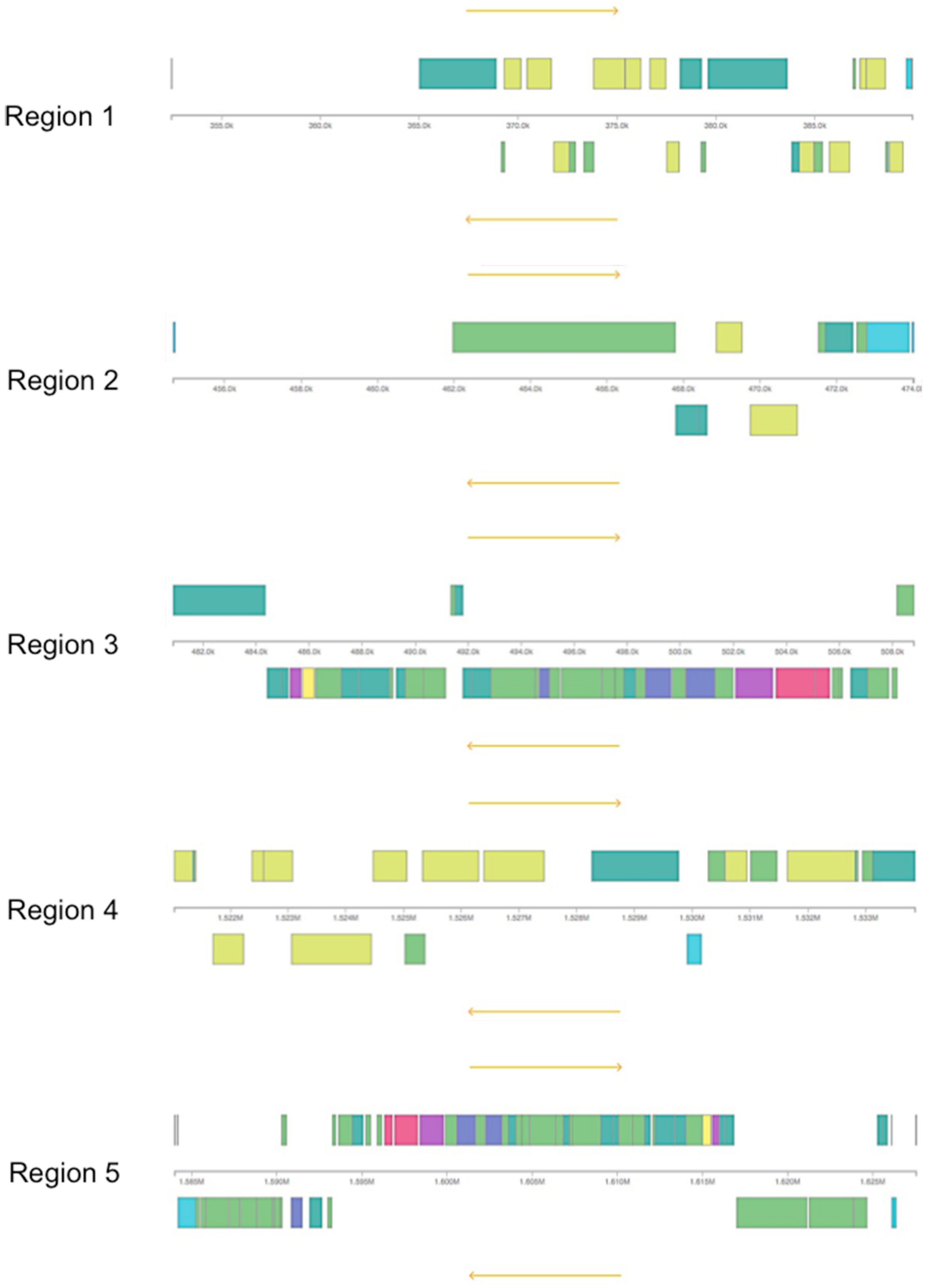

Fig. S1A-2
Sharmin, D., et al

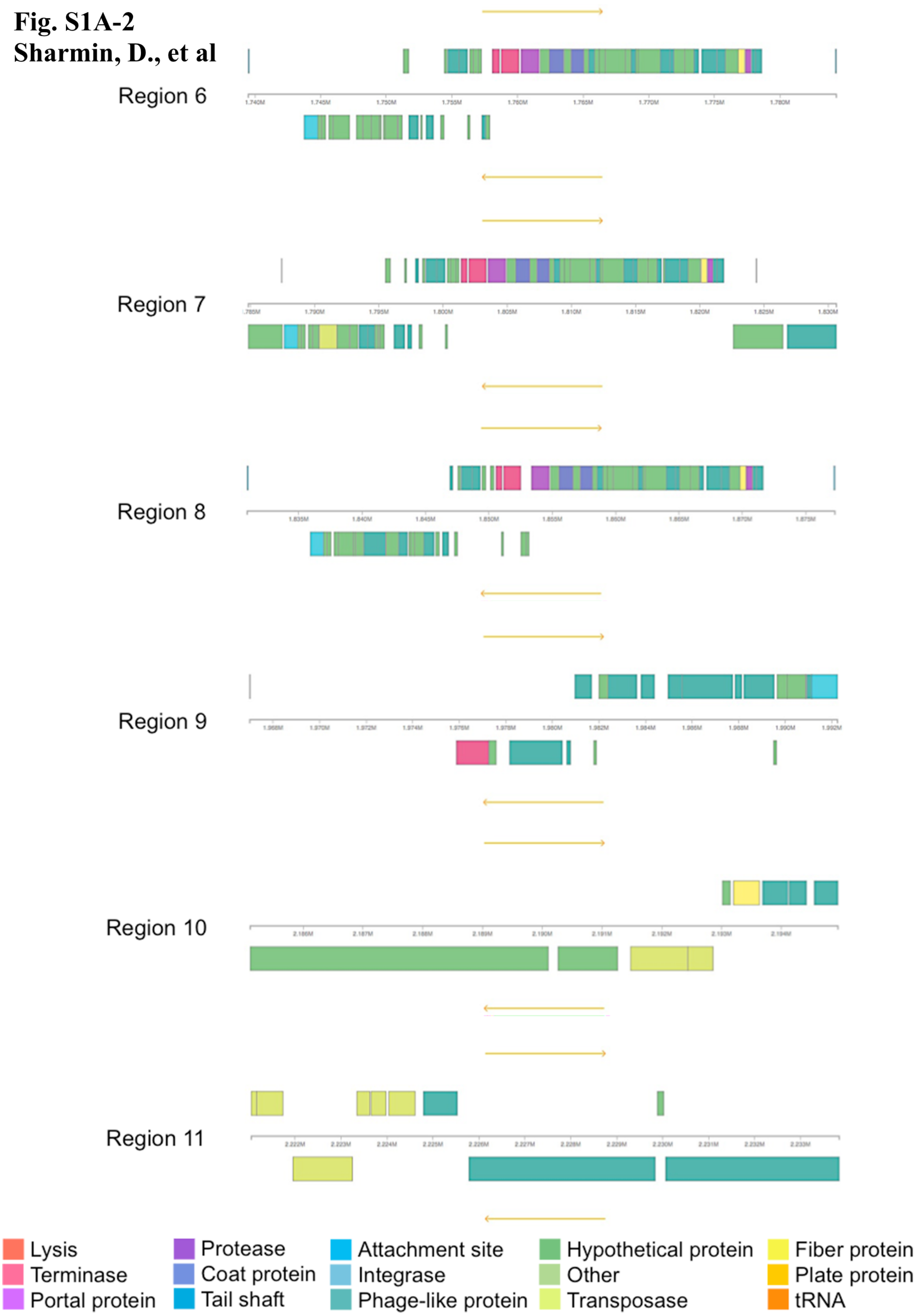

Fig. S1B-1
Sharmin, D., et al

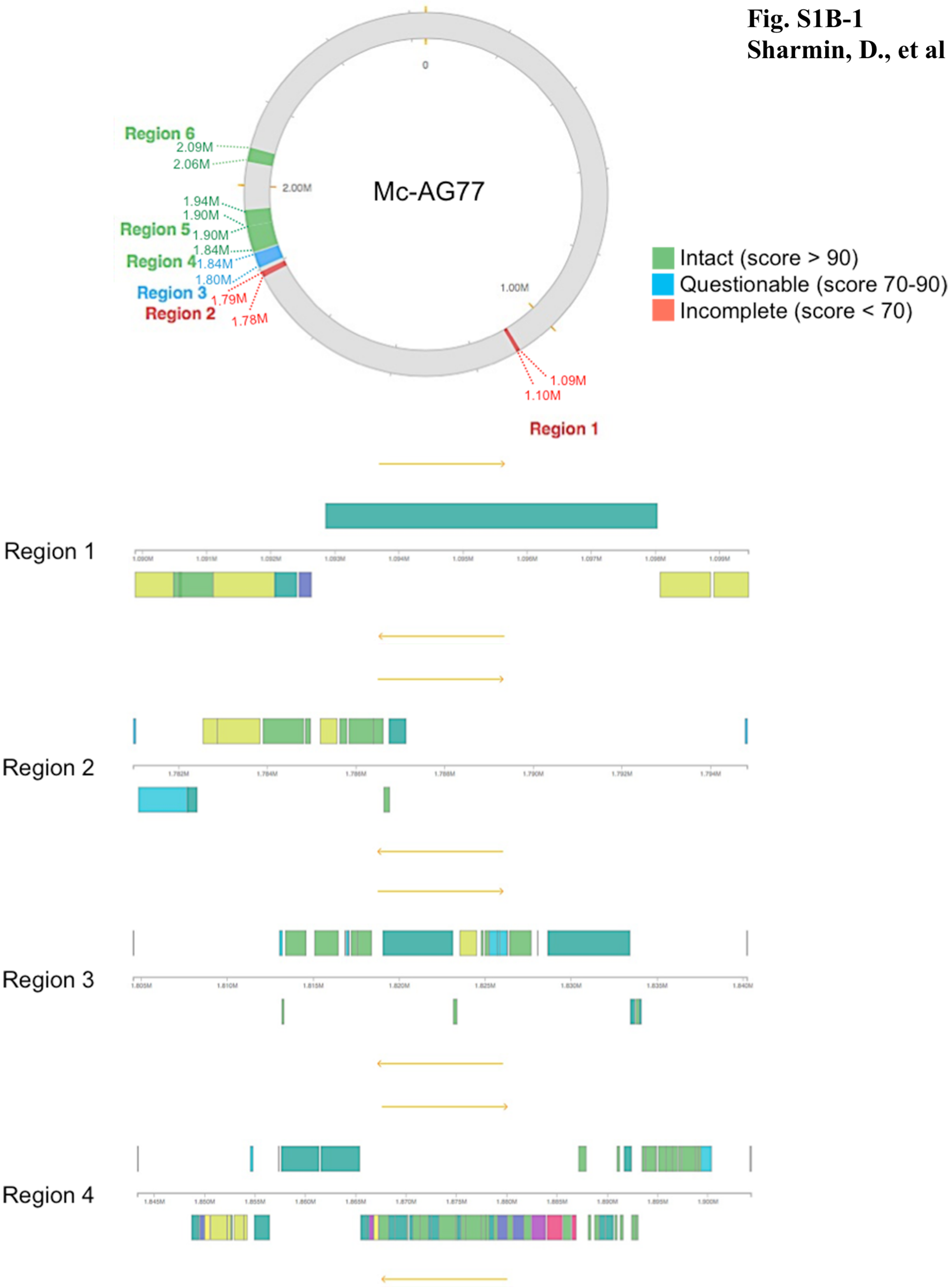

Fig. S1B-2
Sharmin, D., et al

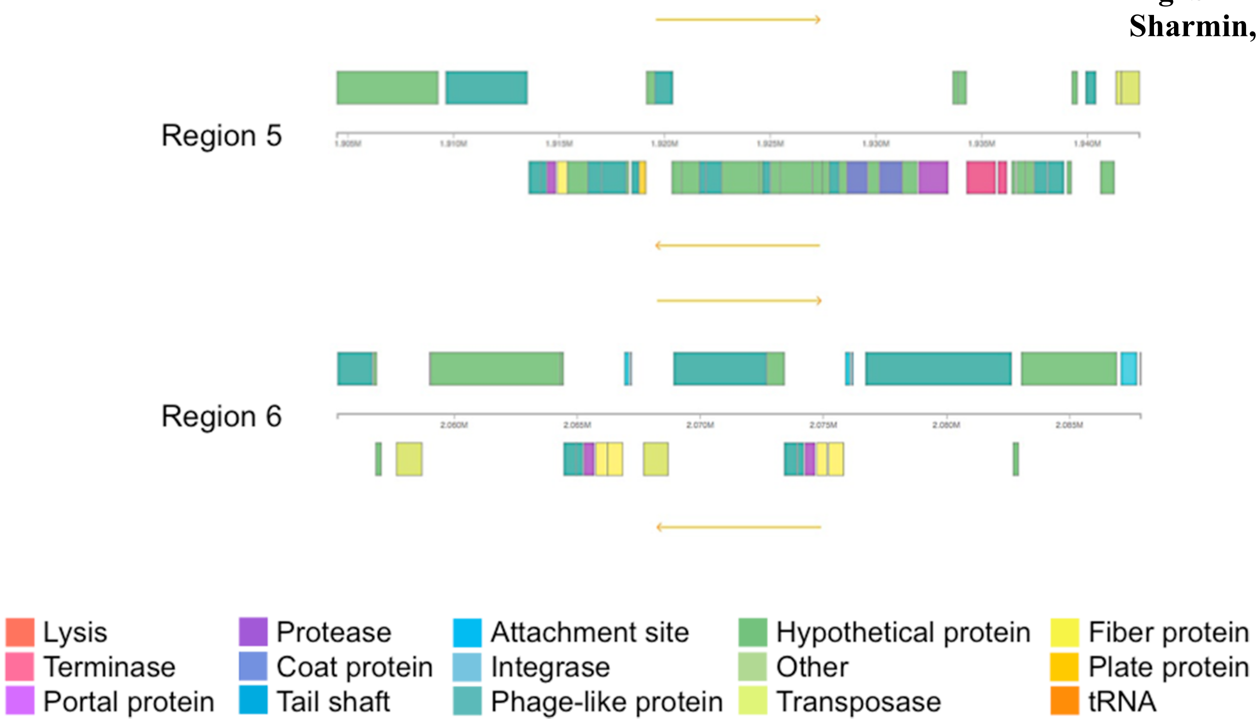

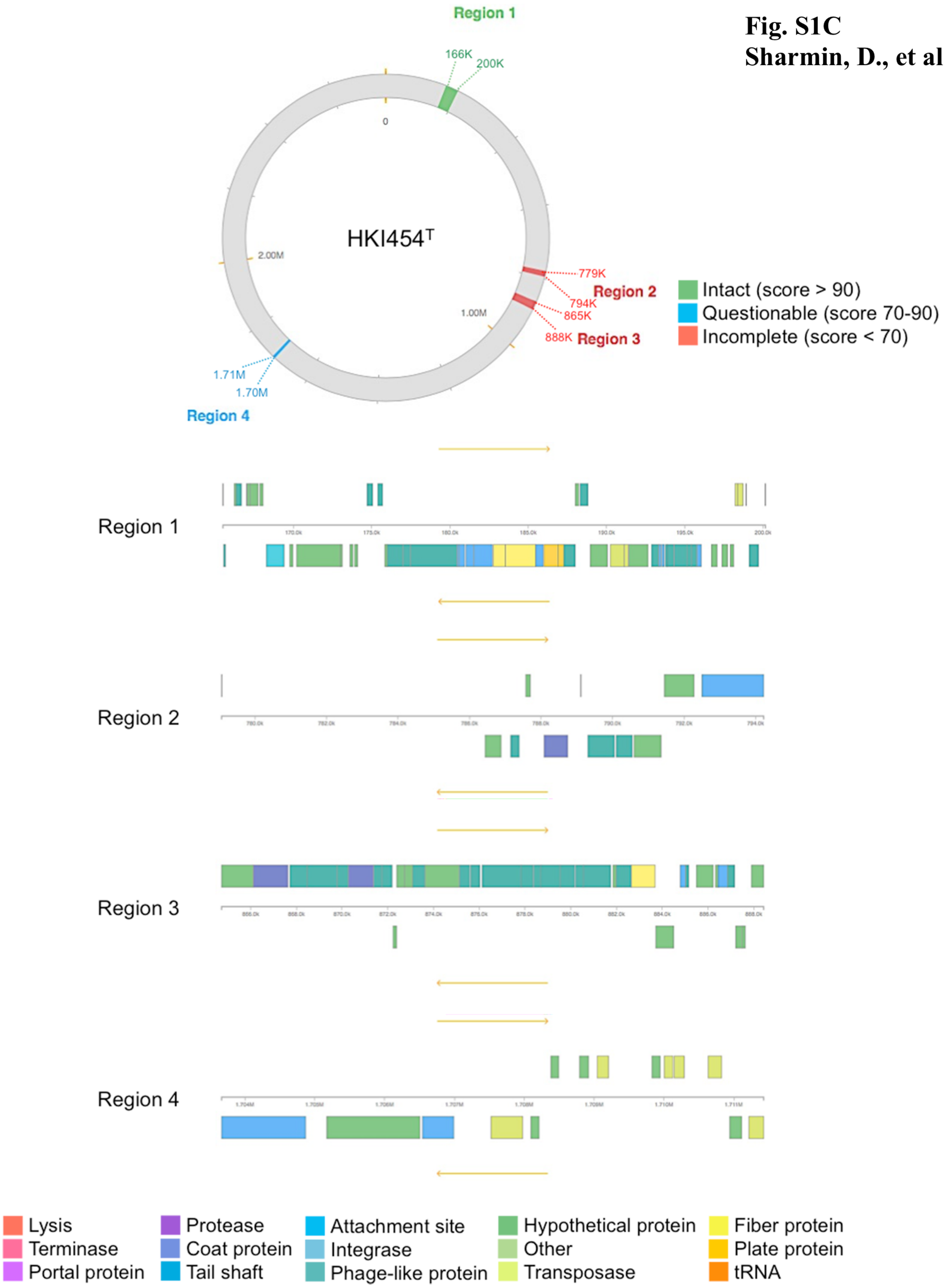

Supplemental Fig. S1. Prophage sequences within the genomes of (A) B1-EB^T (B) Mc-AG77 and (C) HKI 454^T chromosome. The details are showed in each region. Position-based identification of gene functions is shown in colors. Scale bar is in K/M bp.

Fig. S2
Sharmin, D., et al

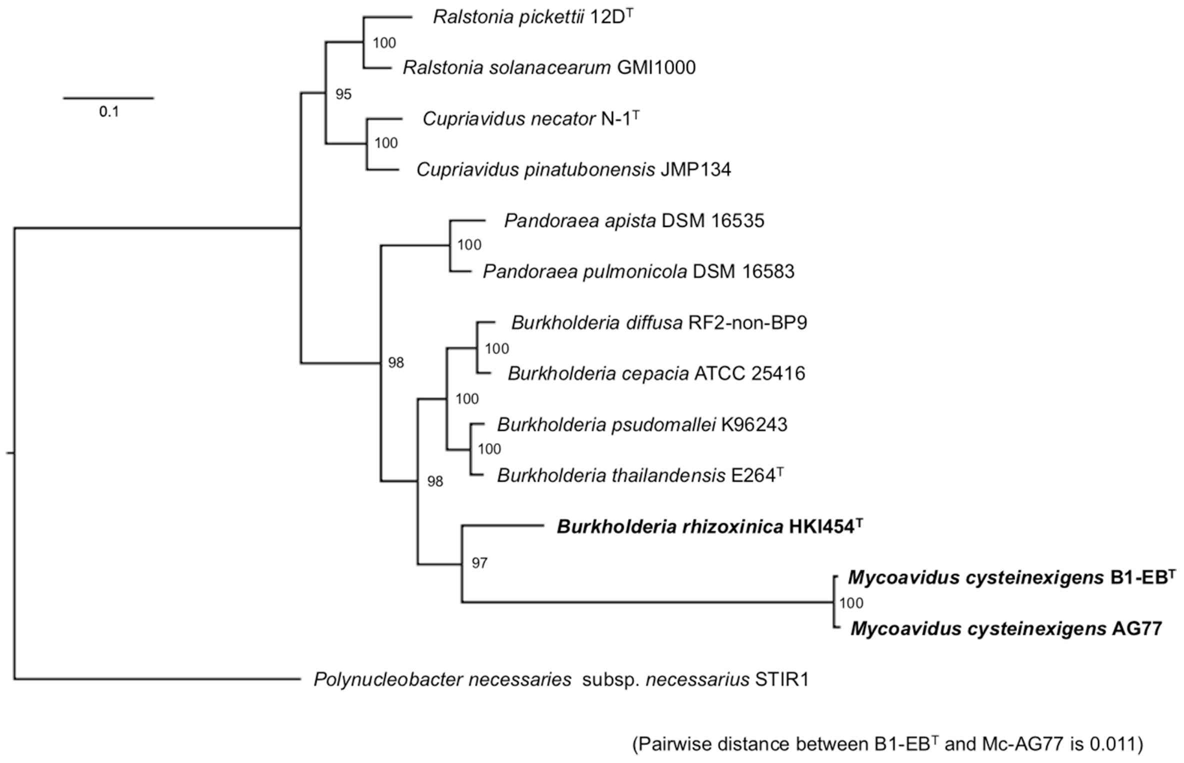

Supplemental Fig. S2. Randomized axelerated maximum likelihood (RAxML) tree based on concatenated sequences (12,492 positions in total) of 16S ribosomal RNA and the five housekeeping genes (*atpD*, *gyrB*, *lepA*, *recA*, and *rpoB*) indicating the relative placement of the three endofungal bacteria (bold) and other genera in the family *Burkholderiaceae* and *Ralstoniaceae*. The horizontal lines show genetic distances, which are supported by values estimated with 100 bootstrap replicates. The scale bar indicates the number of substitutions per nucleotide position.
